# Discovery of a Dual IDO1/TDO Inhibitor That Modulates Enzymatic Activity and IDO1/SHP‑2 Signaling

**DOI:** 10.1021/acs.jmedchem.6c00759

**Published:** 2026-07-22

**Authors:** Sarah Jane Rezzi, Andrea Butticè, Salvatore Villani, Gaia Lesca, Sofia Rossini, Eleonora Panfili, Daniele Mazzoletti, Elia Bari, Davide Violante, Marta Serafini, Alberto Massarotti, Ciriana Orabona, Maria Teresa Pallotta, Silvio Aprile, Silvia Fallarini, Erika Del Grosso, Rita Maria Concetta Di Martino, Riccardo Miggiano, Tracey Pirali

**Affiliations:** † Department of Pharmaceutical Sciences, 60253 Università Degli Studi Del Piemonte Orientale, Novara 28100, Italy; ‡ Department of Medicine and Surgery, 60250 University of Perugia, Perugia 06132, Italy

## Abstract

Indoleamine 2,3-dioxygenase 1 (IDO1) inhibitors have shown limited clinical translation, partly because IDO1 blockade can be bypassed by tryptophan 2,3-dioxygenase (TDO) and may stabilize nonenzymatic IDO1 signaling states that promote tumor progression. Here, we report compound **66**, an *ortho*-benzamidourea derivative obtained through the optimization of VS-15. Compound **66** displayed improved metabolic stability, balanced IDO1/TDO inhibition, and spectroscopic behavior consistent with *apo*-enzyme targeting. In tumor cells, **66** reduced kynurenine-pathway output and attenuated the IDO1/SHP-2 interaction, counteracting IDO1-driven noncatalytic signaling. This multimodal profile was associated with the inhibition of cancer cell proliferation and migration. Compound **66** therefore represents a chemical probe for dissecting enzymatic and signaling functions within the IDO1/TDO axis and supports small-molecule modulation of both catalytic and noncatalytic immune-metabolic pathways as an alternative to purely catalytic IDO1 inhibition or complete IDO1 degradation.

## Graphical abstract

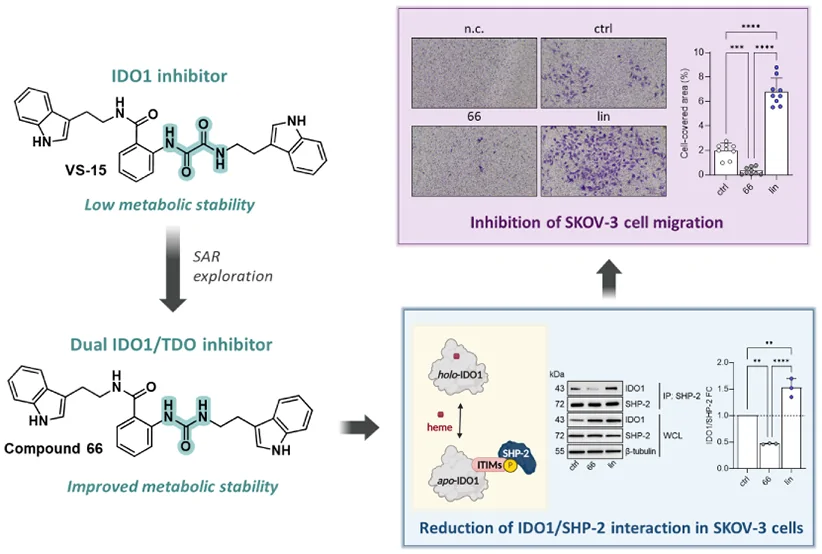

## Introduction

Indoleamine 2,3-dioxygenase 1 (IDO1) is a key target in cancer immunotherapy and is expressed by both tumor cells and immune cells within the tumor microenvironment (TME).^1^ Initially regarded as a simple metabolic enzyme, IDO1 is now recognized as a multifunctional protein whose dual enzymatic and nonenzymatic activities may contribute to the limited clinical efficacy of catalytic inhibitors developed to date.

At the molecular level, IDO1 exists in a dynamic equilibrium between two conformational states: a catalytically active *holo*-form, in which the heme cofactor is bound, and a heme-free *apo*-form.^2,3^ These two forms support distinct biological functions: *holo*-IDO1 acts as an intracellular enzyme, whereas *apo*-IDO1 functions as a signaling protein.^4^

*Holo*-IDO1 catalyzes the oxidative degradation of l-tryptophan into immunosuppressive kynurenine metabolites through an active site located in the large domain of the protein.^5^ Accumulation of these metabolites contributes to immune tolerance by promoting regulatory T-cell (Treg) differentiation, suppressing effector T-cell and natural killer (NK) cell activity, and inducing tolerogenic dendritic cells (DCs).^1^

In contrast, *apo*-IDO1 mediates nonenzymatic signaling functions.^5^ The small domain of IDO1 contains two immunoreceptor tyrosine-based inhibitory motifs (ITIMs) that can be phosphorylated by Src family kinases, enabling recruitment of the tyrosine phosphatases SHP-1 and SHP-2.^6^ The downstream consequences of ITIM-dependent signaling are cell-type dependent. In DCs, particularly in a transforming growth factor beta (TGF-β)-rich microenvironment, ITIM phosphorylation promotes SHP-1/2 recruitment,^6,7^ resulting in the retention of IDO1 within early endosomes (EEs). This endosomal confinement accounts for its apparent cytosolic sequestration and defines the subcellular compartment associated with its signaling activity.^4,8^ This pathway reinforces a tolerogenic DC phenotype through a positive feedback loop that sustains IDO1 expression. By contrast, in tumor cells, engagement of the IDO1/SHP-2 axis activates proliferative and migratory signaling pathways, ultimately promoting tumor growth and invasion.^9–11^

Despite strong preclinical validation, most catalytic IDO1 inhibitors have failed to demonstrate clinical efficacy.^12–14^ Notably, the selective *holo*-IDO1 inhibitor epacadostat did not improve clinical outcomes when combined with pembrolizumab in a Phase III trial in melanoma patients.^15^ Several hypotheses have been proposed to explain these disappointing results, including compensatory upregulation of tryptophan 2,3-dioxygenase (TDO) following IDO1 inhibition.^16–18^ In this context, dual IDO1/TDO inhibitors have emerged as a potential strategy to overcome the limitations of selective IDO1 inhibition (Figure 1A).^16,17,19–21^ However, only a limited number of such compounds have been reported to date, and many display weak or unbalanced dual activity, with limited evidence of simultaneous target engagement in cellular systems. Among these dual IDO1/TDO inhibitors, SHR9146 has progressed to Phase I clinical evaluation, both as monotherapy and in combination regimens (Figure 1A, NCT03208959 and NCT03491631), while its close analog IDO1/TDO-IN-1 has shown efficacy in preclinical models.^22^ In addition, the dual inhibitor G-4 demonstrated immunomodulatory potential by significantly attenuating LPS-induced proinflammatory cytokine production in mouse primary macrophages.^18^

**Figure 1:**
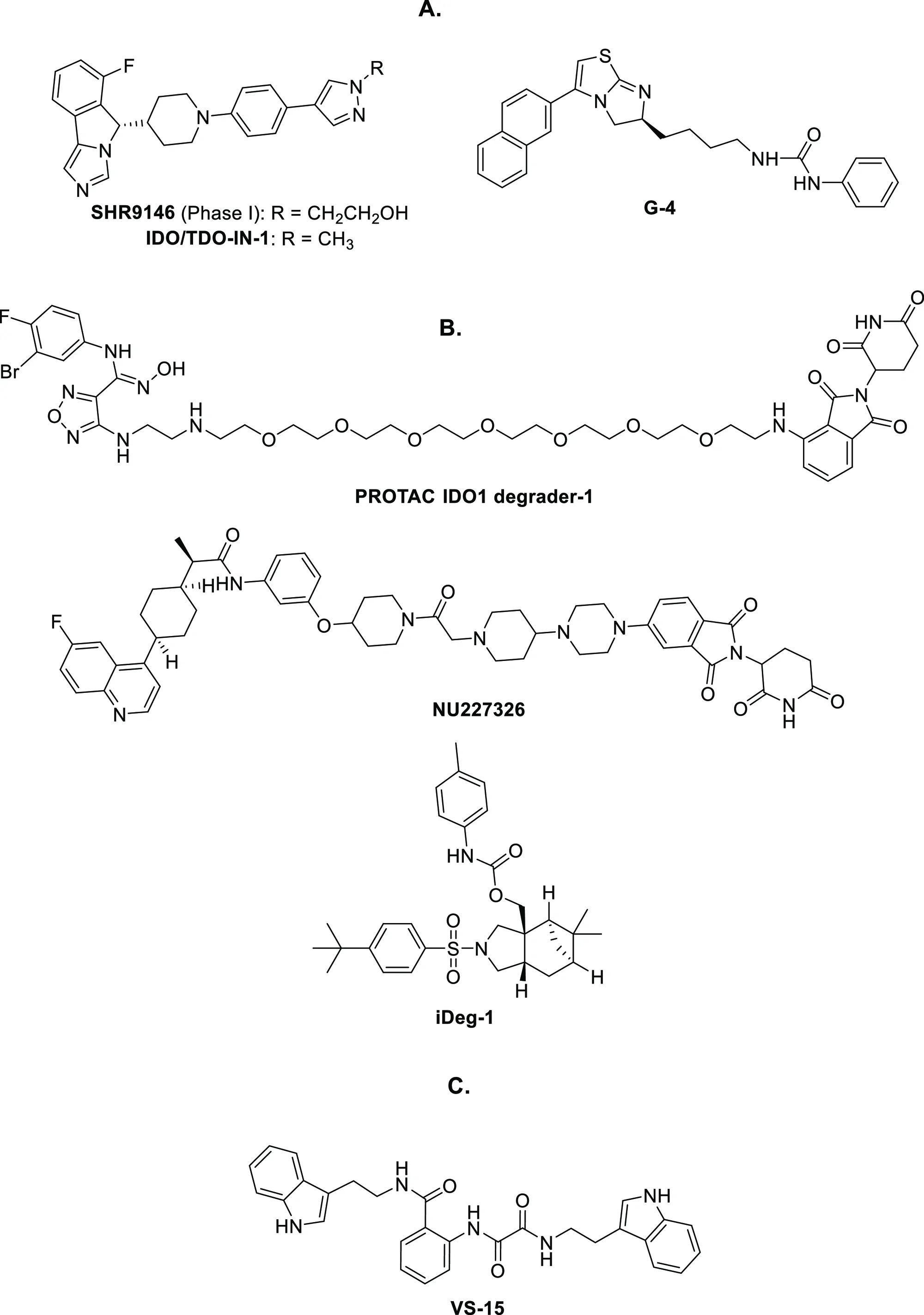
Representative chemical structures of A) dual IDO1/TDO inhibitors, B) PROTACs and iDegs targeting IDO1, and C) VS-15.

An additional emerging explanation for the clinical failure of catalytic IDO1 inhibitors is that, irrespective of their inhibitory mechanism, they may stabilize nonenzymatic IDO1 conformations, thereby favoring SHP-2 interaction and activation of intracellular signaling pathways that support, rather than suppress, tumor cell proliferation and migration.^10,23–25^ These observations suggest that selective inhibition of IDO1 enzymatic activity may be insufficient, or even detrimental, in tumor cells where signaling-competent IDO1 predominates. To address both enzymatic and nonenzymatic functions of IDO1, targeted protein degradation has recently been explored as an alternative strategy. Epacadostat- and linrodostat-based PROTACs^26–28^ (Figure 1B), as well as pseudonatural products derived from (−)-myrtanol^29^ (Figure 1B), have been described. While effective in eliminating all IDO1-associated functions, degraders may not always represent the optimal approach to selectively interrogate IDO1 biology or to disentangle catalytic from signaling contributions. As a complementary strategy, small molecules capable of directly interfering with IDO1 signaling, particularly by modulating IDO1/SHP interactions, could selectively suppress pro-tumorigenic IDO1 functions while retaining control over enzymatic activity. Through a structure-based virtual screening (SBVS) campaign, we previously identified VS-15 (Figure 1C; IC_50_ = 480 nM in A375 cells), a small-molecule ligand that selectively binds the *apo*-form of IDO1, thereby inhibiting enzymatic activity. Importantly, VS-15 also disrupts IDO1 signaling by impairing its interaction with SHP-2, leading to downregulation of IDO1 expression. However, the poor metabolic stability of VS-15, primarily attributed to its oxalyl amide moiety, limited its further development. In addition, the lack of activity against TDO does not address compensatory TDO overexpression.^30^

In the present study, we aimed to improve the metabolic stability of VS-15 while preserving its unique multimodal biological profile and introducing TDO inhibitory activity. To this end, we conducted a medicinal chemistry optimization campaign that led to the identification of a novel compound featuring an unprecedented *ortho*-benzamidourea scaffold. This compound exhibits balanced dual inhibitory activity against IDO1 and TDO and interferes with IDO1 signaling by modulating SHP-2-mediated pathways. By simultaneously targeting enzymatic activity and nonenzymatic signaling, the compound reverses the intrinsic IDO1-driven pro-tumorigenic phenotype in tumor cells, supporting an alternative strategy for targeting IDO1 biology in cancer immunotherapy.

## Results and Discussion

### Metabolic Profiling of VS-15

VS-15 is chemically stable under a range of experimental conditions; however, it exhibits poor metabolic stability, with less than 5% of parent compound remaining after 60 min of incubation in mouse liver microsomes (MLM).^30^ To identify the metabolic liabilities responsible for its rapid clearance, the biotransformation of VS-15 was investigated by LC–HRMS/MS.

Metabolic profiling revealed that the primary pathway involves enzymatic hydrolysis of the oxalyl amide moiety, leading to the formation of metabolite M1 as the predominant species (Figure 2). This transformation was identified as the main contributor to the low metabolic stability of VS-15. Based on the structure of M1, hydrolysis was hypothesized to generate amido-oxoacetic acid intermediate and tryptamine (1). Since tryptamine is a known IDO1 inhibitor that could potentially interfere with downstream biological assays,^31^ its formation was specifically evaluated. No ions attributable to tryptamine (1) or its known metabolites^32^ were detected under the experimental conditions employed (Table S1, Supporting Information).

**Figure 2:**
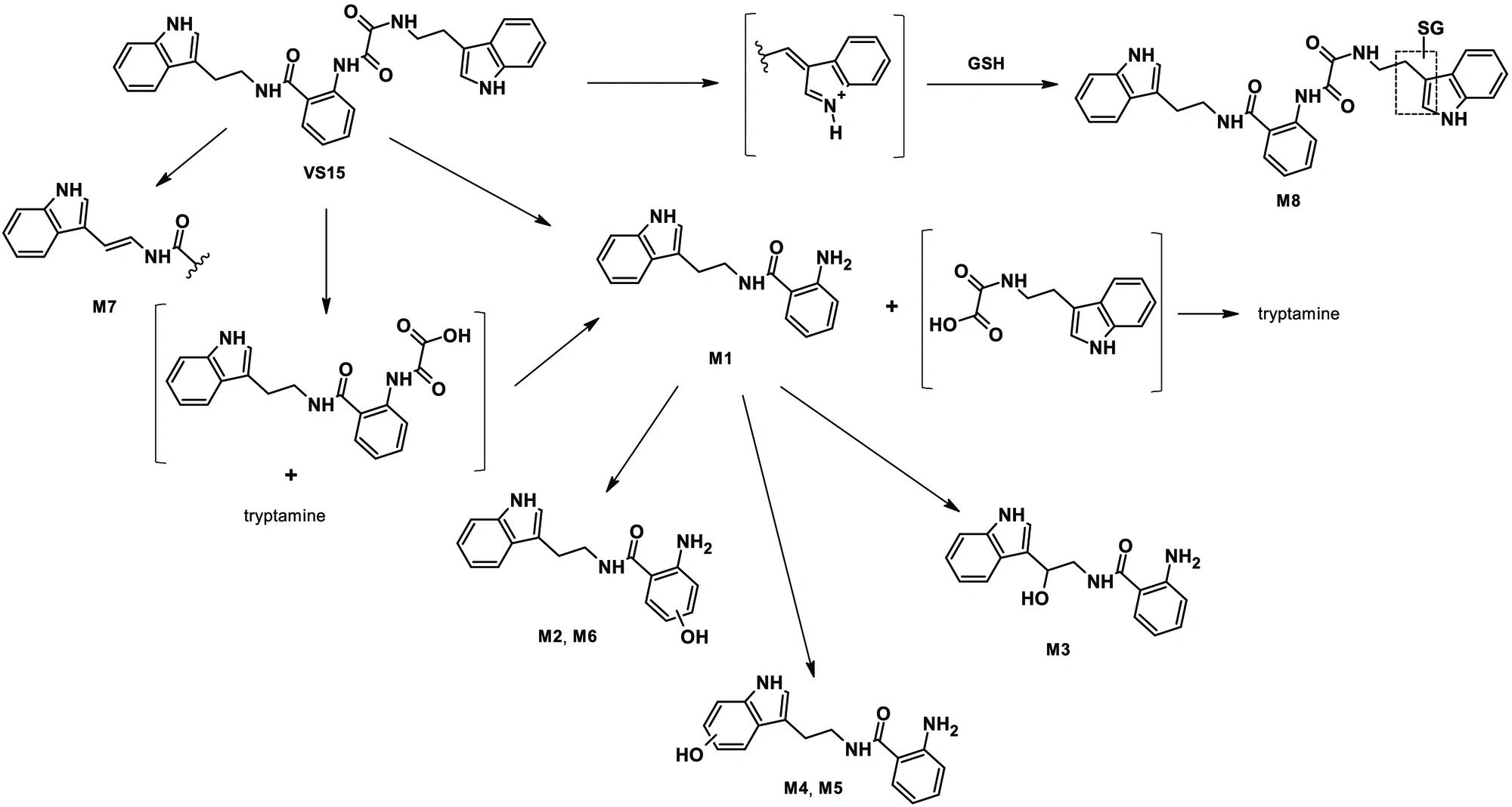
Proposed *in vitro* phase I metabolism pathways of VS-15.

Upon activation of the microsomal mixed-function oxidase system with NADPH, additional metabolites were identified, arising from hydroxylation of M1 (metabolites M2–M6) and substrate dehydrogenation (M7) (Figure 2; Figure S1 and Table S1, Supporting Information). Furthermore, inclusion of reduced glutathione (GSH) in the incubation mixture led to the detection of metabolite M8, consistent with trapping of a reactive iminium intermediate.^33,34^

### Synthesis of VS-15 Analogues

The identification of the oxalyl amide moiety of VS-15 as a key metabolic soft spot provided the rationale for its structural modification in subsequent optimization efforts. The synthetic strategy focused on replacing this motif while preserving the structural elements required for *apo*-IDO1 binding. According to the docking pose in *apo*-IDO1, VS-15 occupies pockets A and D of the IDO1 active site, forming key hydrogen bonds with Ala264 and Ser167 in pocket A, and with Ser267 in pocket D. The tryptamine substructure of VS-15, predicted to reside in pocket A, along with the central phenyl ring, also engages a π–π interaction with Phe163 (Figure 3).^30^

**Figure 3:**
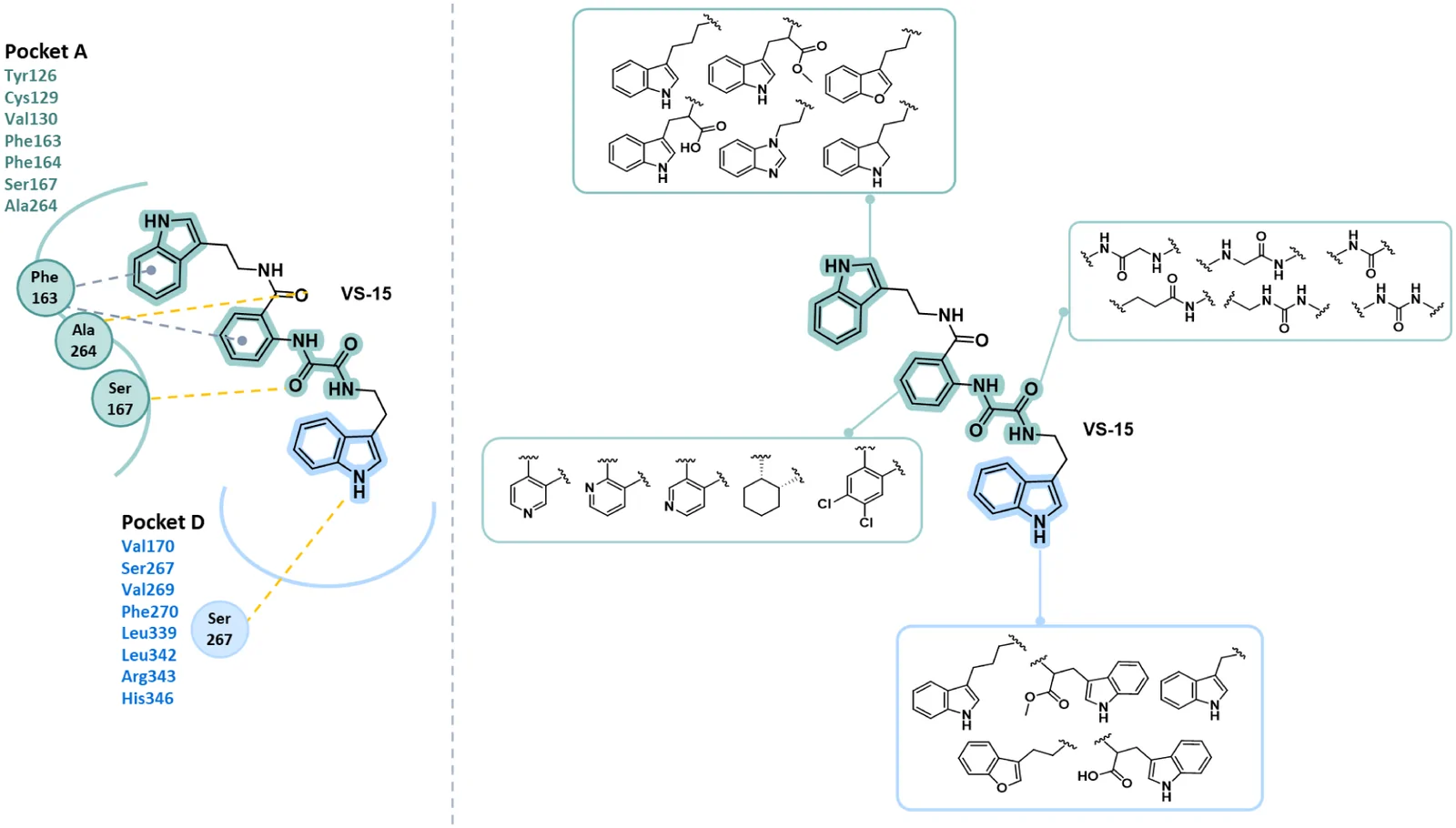
Left: Schematic representation of predicted interactions of VS-15 within the active site of *apo*-IDO1. Hydrogen bonds are represented by yellow dotted lines, and π–π stacking interactions are shown as gray dotted lines. Right: structure–activity relationship (SAR) exploration performed around compound VS-15.

### Modification of the Indole Moiety Located in Pocket A

The tryptamine substructure of VS-15, predicted to occupy pocket A, was modified through (i) chain extension, (ii) insertion of polar functionalities (−COOMe and −COOH), and (iii) ring equivalence (Scheme 1). To achieve this, aminolysis of commercially available ethyl ester **6** with tryptamine (**1**) in toluene under reflux afforded intermediate **7**, which was subsequently coupled with 3-(1*H*-indol-3-yl)­propan-1-amine (**2**), methyl tryptophanate (**3**), and 2-(benzofuran-3-yl)­ethan-1-amine (**4**) to afford amides **12–14**, respectively. Hydrolysis of the methyl ester function of **13** led to **15**.

**Scheme 1:**
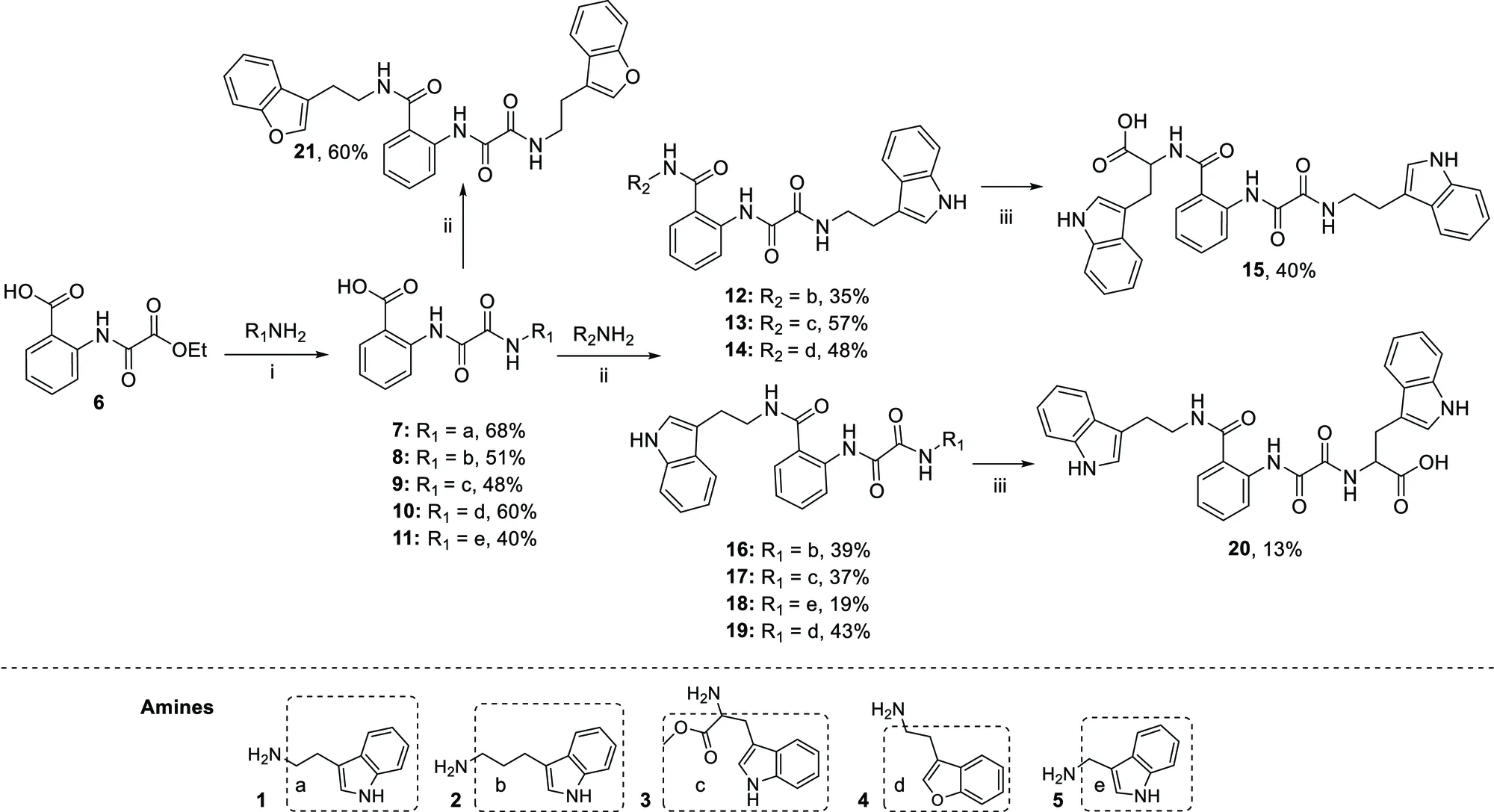
Multistep Synthesis of Compounds **12–21** ^a^

a Reagents conditions: i) TEA, toluene, reflux, overnight; ii) EDCI, HOBt, TEA, dry DCM, rt, N_2_, 18 h; iv) NaOH, THF/water (1:1), rt, overnight.

### Modification of the Indole Moiety Located in Pocket D

Following a similar approach to that applied to the tryptamine moiety predicted to engage pocket A (i.e., chain extension/contraction, insertion of polar functionalities and ring equivalence), we next targeted the second tryptamine substructure of VS-15, which is predicted to occupy pocket D (Scheme 1). Aminolysis of ethyl ester **6** with amines **2–4** and (1*H*-indol-3-yl)­methanamine (**5**) yielded compounds **8–11**. A subsequent coupling of **8–11** with tryptamine (**1**) afforded the corresponding amides **16–19**. Finally, hydrolysis of the methyl ester group of **17** gave **20**.

### Substitution of Both Indole Rings

To investigate the role of the indole -NH- moiety, we also designed compound **21** by replacing both tryptamine substructures of VS-15 with 2-(benzofuran-3-yl)­ethyl groups (Scheme 1). The synthesis of this derivative involved an amide coupling reaction between **10** and amine **4**.

### Modification of the Oxalyl Amide Moiety and the Central Phenyl Ring

The labile oxalyl moiety of VS-15 was substituted with isosteric substructures lacking adjacent carbonyl groups, with the aim of improving metabolic stability and modulating the conformational flexibility of the molecule. The initial strategy involved the removal of a carbonyl group, ultimately leading to the synthesis of derivatives **26** and **29**, featuring an acetamido functionality (Scheme 2). Compound **26** was prepared from amide intermediate **23**, which was acylated with 2-bromoacetyl chloride (**24**), followed by a nucleophilic substitution with tryptamine (**1**). Conversely, to prepare **29**, a nucleophilic substitution of 2-bromoacetic acid (**27**) with **23** afforded carboxylic acid **28**, which was finally coupled with tryptamine (**1**). The key intermediate **23** itself was prepared by coupling **22** with **1**.

**Scheme 2:**
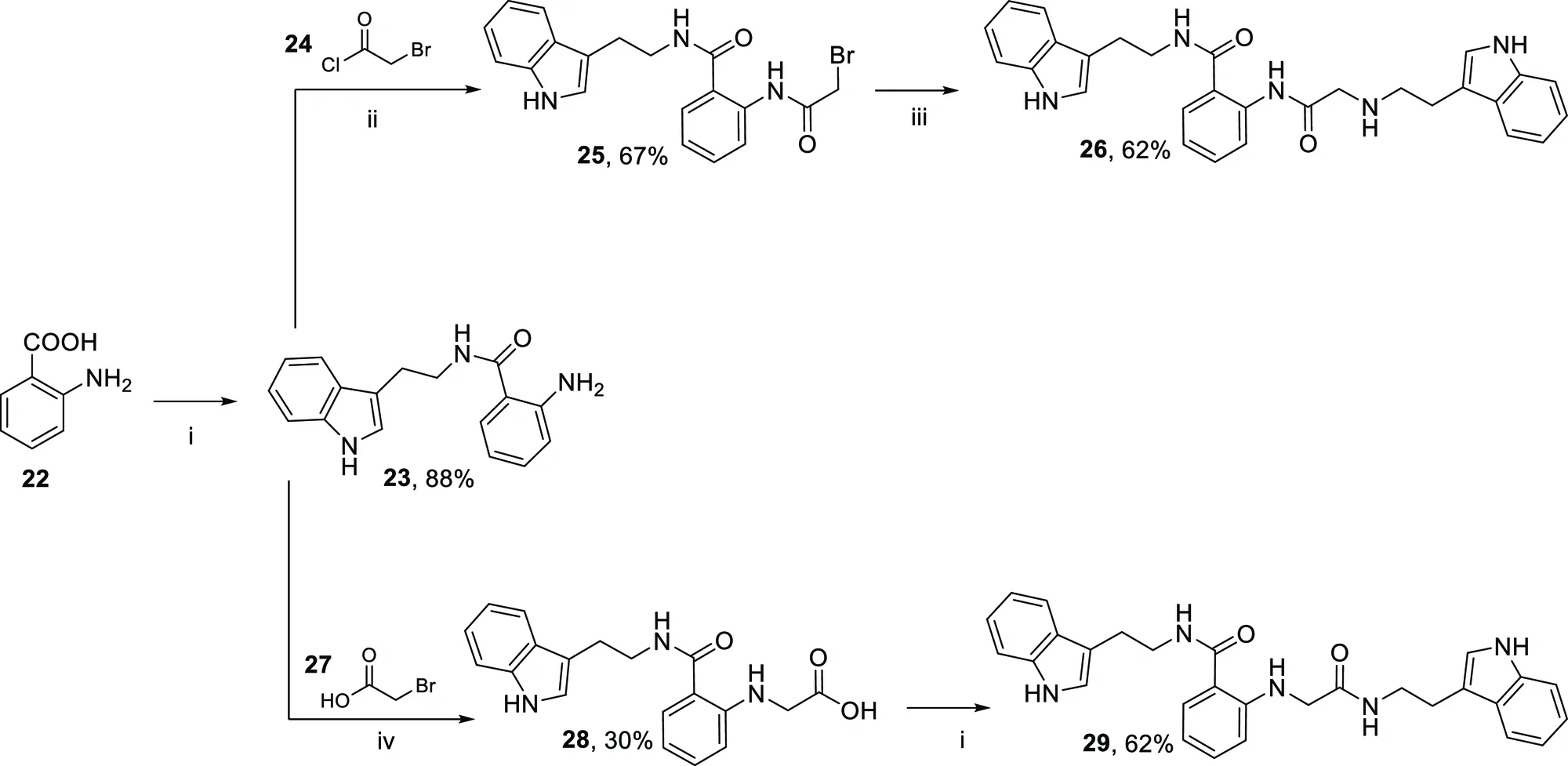
Multistep Synthesis of **26** and **29** ^a^

a Reagents and conditions: (i) **1**, EDCI, HOBt, TEA, dry DCM, rt, N_2_, 18 h; (ii) TEA, dry DCM, 0 °C, N_2_, 4 h; (iii) K_2_CO_3_, CH_3_CN, 90 °C, 18 h; (iv) DMAP, TEA, EtOH, reflux, 8 days.

Similarly, systematic removal of each amide moiety within the oxalyl amide scaffold was explored, while either retaining or reducing the length of the spacer between the central phenyl ring and the terminal indole predicted to occupy pocket D, as illustrated in Schemes 3 and 4. By coupling commercially available carboxylic acid **30** and aniline **31**, we prepared amide **32**, whose ethyl ester function was hydrolyzed under basic conditions. Finally, an amide coupling reaction between the obtained carboxylic acid **33** and tryptamine (**1**) yielded diamide derivative **34** (Scheme 3).

**Scheme 3:**
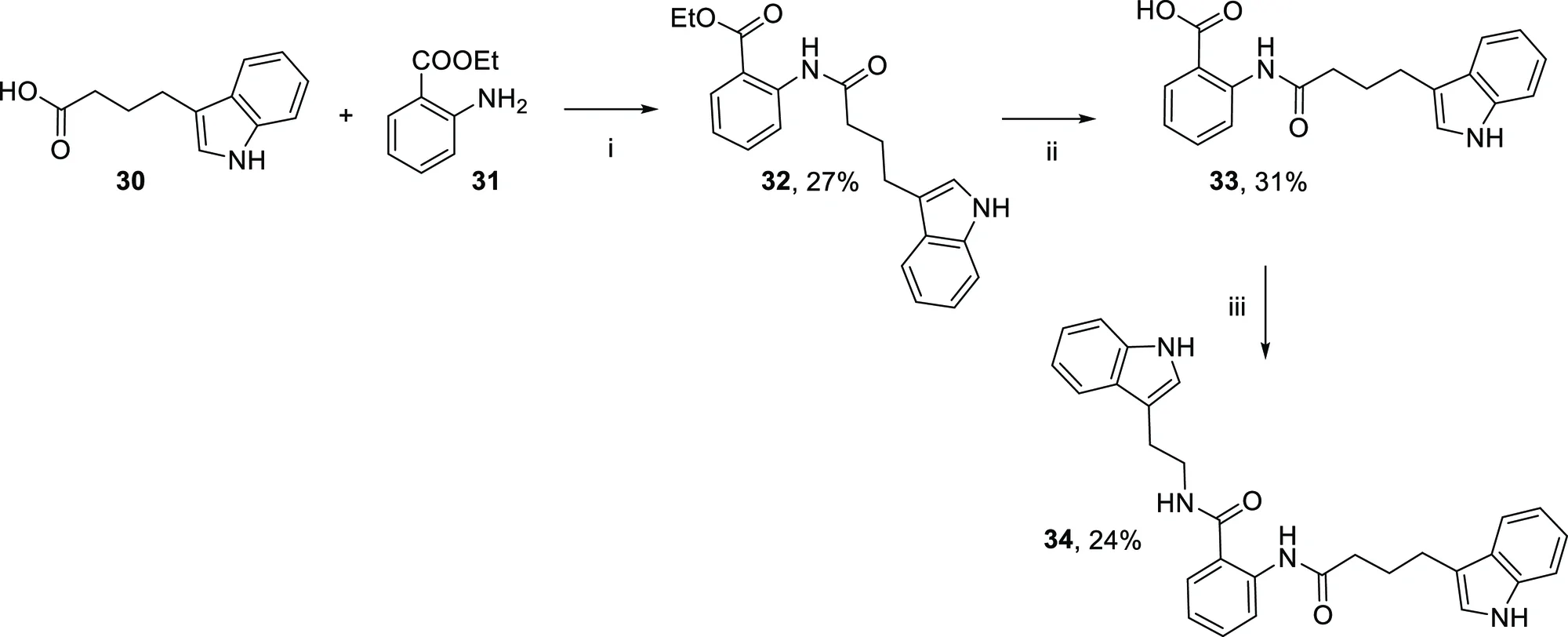
Synthesis of Diamide Derivative **34** ^a^

a Reagents conditions: (i) EDCI, DMAP, TEA, DIPEA, dry DCM, rt, N_2_, 48 h; (ii) NaOH, THF/water (1:1), rt, overnight; (iii) **1**, EDCI, HOBt, TEA, dry DCM, rt, N_2_, 18 h.

**Scheme 4:**
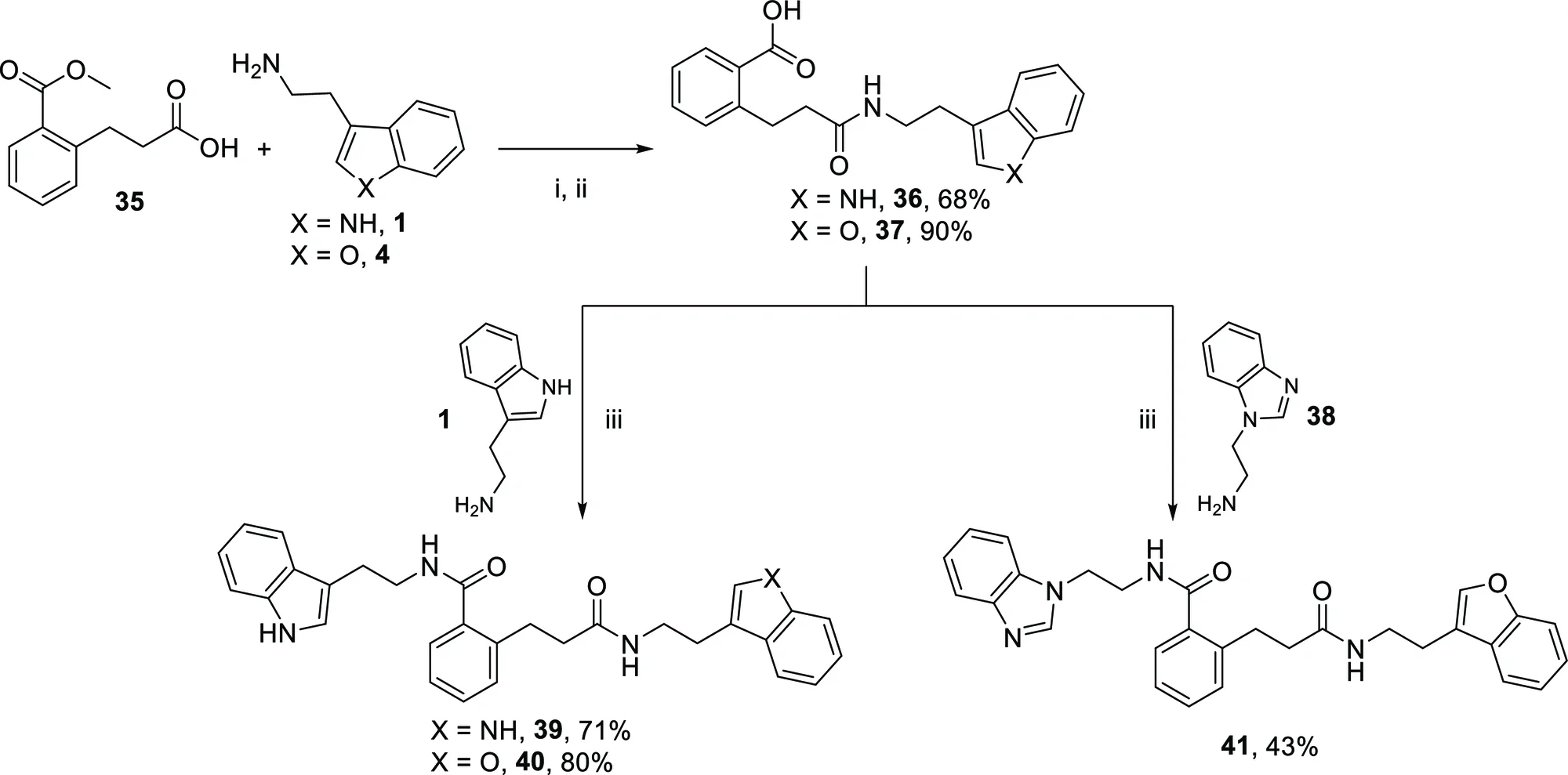
Preparation of Diamide Compounds **39–41** ^a^

a Reagents conditions: (i) TEA, EDCI, DIPEA, DMAP, dry DCM, rt, N_2_, 48 h, 58–76%; (ii) NaOH, THF/water (1:1), rt, overnight; (iii) EDCI, HOBt, TEA, dry DCM, rt, N_2_, 18 h.

A similar synthetic route was used to prepare diamide derivatives **39–41** (Scheme 4). In compounds **40** and **41**, the tryptamine substructure, predicted to occupy pocket D, was replaced by a (2-(benzofuran-3-yl)­ethyl)­amino moiety. Furthermore, the tryptamine moiety, predicted to reside within pocked A, was retained in compound **40**, while it was replaced by using 2-(1*H*-benzo­[*d*]­imidazol-1-yl)­ethan-1-amine (**38**) in derivative **41**. Amide coupling reactions of 3-(2-(methoxycarbonyl)­phenyl)­propanoic acid (**35**) with amines **1** and **4**, followed by methyl ester hydrolysis under basic conditions, afforded the corresponding carboxylic acids **36** and **37**, respectively. A subsequent amide coupling reaction between **36** and tryptamine (**1**) gave compound **39**. Conversely, amide coupling reactions of **37** with **1** and **38** afforded **40** and **41**, respectively.

Finally, to further expand the chemical space around VS-15, we replaced the oxalyl amide moiety with a urea group and modified the central phenyl ring, leading to the synthesis of derivatives **60–67** (Scheme 5). In compound **65**, the tryptamine substructure of VS-15, predicted to occupy pocket A, was further modified by saturating the indole ring to give the corresponding indoline analogue. In pursuit of a more environmentally sustainable synthetic route, we employed phenyl 4,5-dichloro-6-oxopyridazine-1­(6*H*)-carboxylate (**59**) as a safer and viable alternative to triphosgene for the one-pot synthesis of the desired ureas.^35^ This method involved the reaction of **59**, prepared by reacting 4,5-dichloropyridazin-3­(2*H*)-one (**58**) with phenyl chloroformate (**57**) in the presence of triethylamine, with tryptamine (**1**) and selected anilines/amines **23**, **50–56**. Notably, this method avoids the formation of chlorine gas or chlorinated byproducts, offering a safer and less toxic alternative to phosgene-based procedures.

**Scheme 5:**
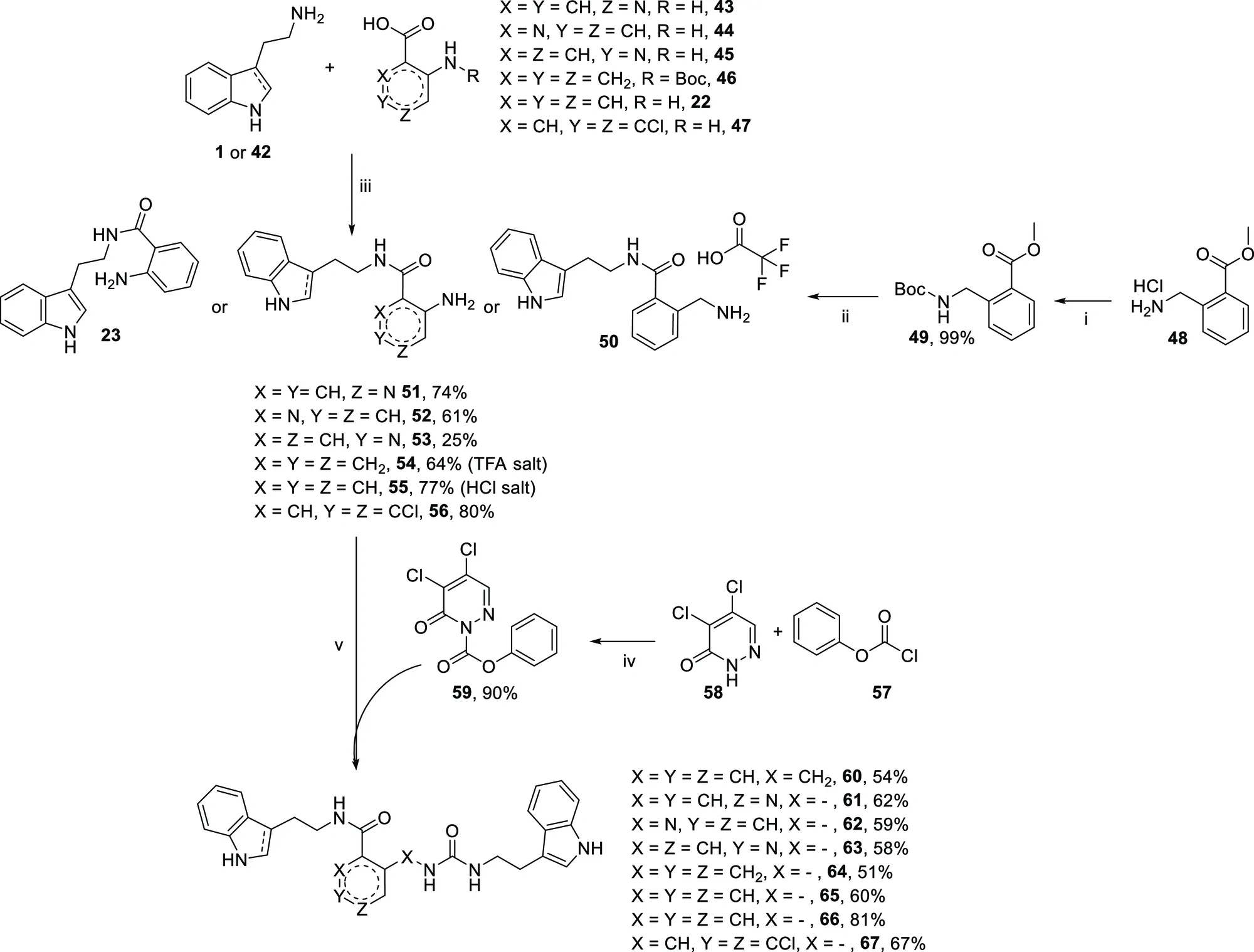
Preparation of Urea Derivatives **60–67** ^a^

a Reagent conditions: (i) Boc_2_O, TEA, dry THF, rt, N_2_, 18 h; (ii) 1. **1**, Cs_2_CO_3_, H_2_O, 100 °C, 4 h, 24%; 2. CF_3_COOH, DCM, 0 °C to rt, 2 h, 40%; (iii) 1. EDCI, HOBt, TEA, dry DCM, rt, 18 h; 2. CF_3_COOH, DCM, 0 °C to rt, 2 h and 30 min, 64% (for **54**); (iv) TEA, dry DCM, 5 °C, N_2_, 20 min; (v) **1**, DMAP, dry THF, rt, N_2_, 7 h.

Anilines **51–56** were, in turn, prepared via an amide coupling reaction between **1** or 2-(indolin-3-yl)­ethan-1-amine (**42**) and commercially available carboxylic acids **22** and **43–47**. To synthesize the trifluoroacetic acid salt **50**, amine **48** was initially protected using di-*tert*-butyl dicarbonate to afford **49**, which then underwent aminolysis with **1**. Final deprotection of the Boc protecting group using trifluoroacetic acid in DCM yielded **50**.

### Identification of Compound **66**

The resulting compound set was subsequently evaluated for cell viability, IDO inhibitory activity and metabolic stability. Molecules were screened in a cell-based assay using A375, a human melanoma-derived cell line. A375 was selected as the cellular model due to its high expression levels of IDO1 upon treatment with recombinant human IFNγ, as confirmed in our previous work *via* RT-PCR^36^ and by measuring *L*-Kyn. In our experimental setting, A375 cells were either left untreated or treated with 10 μM of each compound for 48 h, and *L*-Kyn levels were subsequently measured by HPLC. Epacadostat and linrodostat were used as positive controls (Table 1). Importantly, with the exception of compound **12**, cell viability remained at or above 86% in all cases, indicating that the observed reduction in *L*-Kyn levels was not due to direct compound cytotoxicity.

**Table 1:**
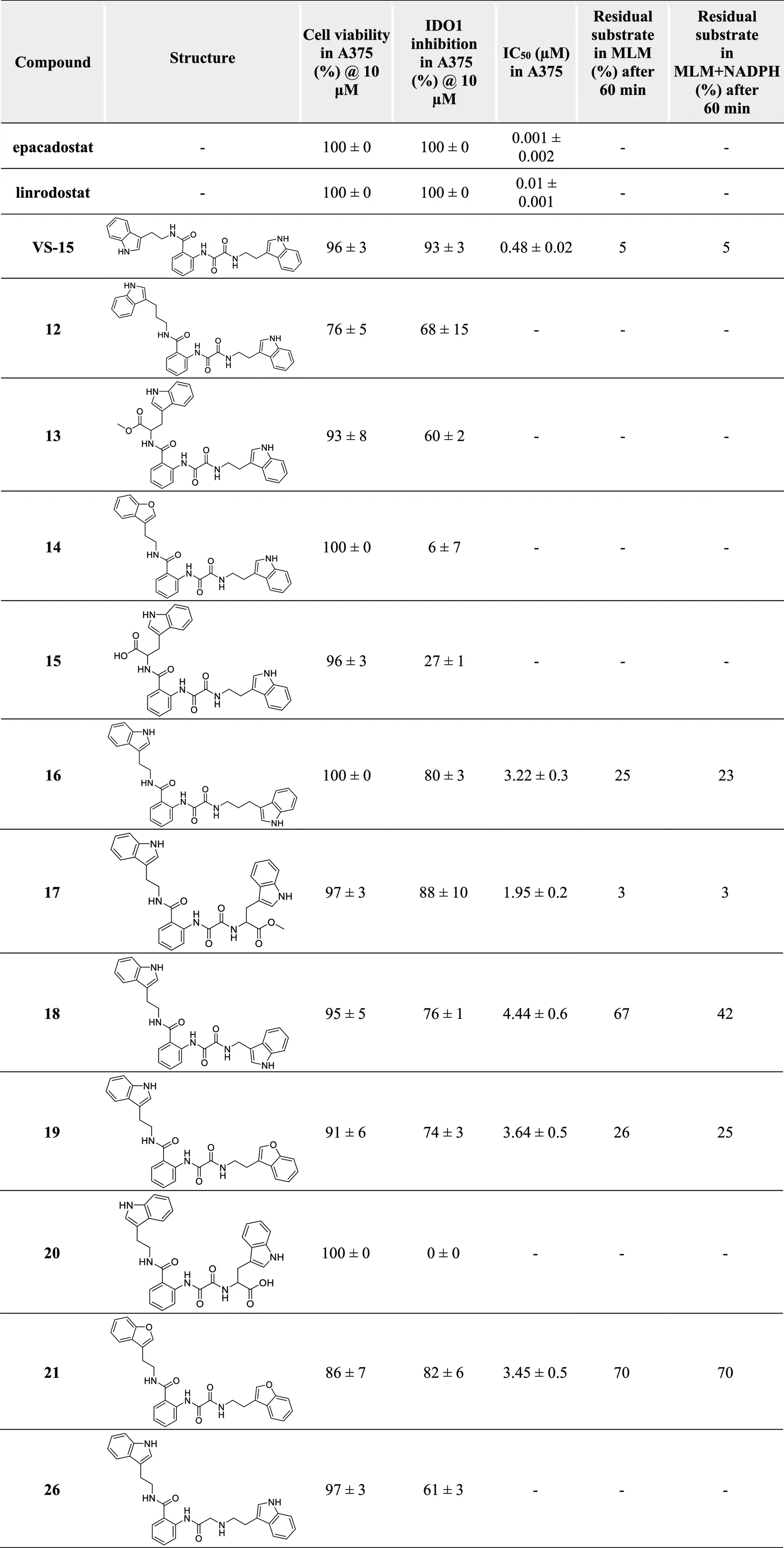

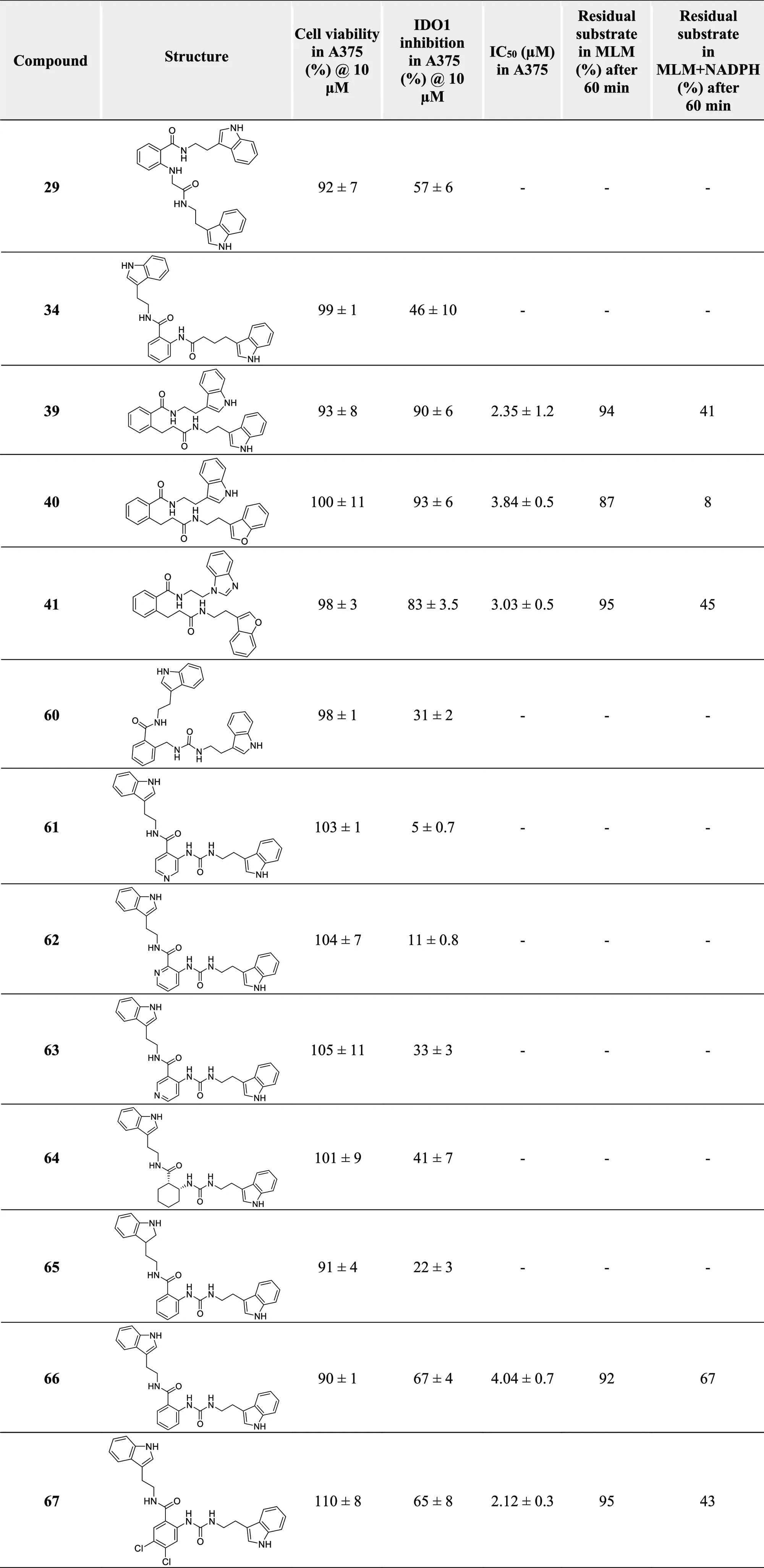
Cellular Viability, IDO1 Inhibitory Activity in A375 Cells, and Metabolic Stability Data for Selected Compounds

Compounds showing cell viability ≥80% and inhibition ≥65% were selected for determination of IC_50_ values and for investigation of metabolic stability. The latter was assessed by quantifying residual substrate after 60 min in MLM, both in the absence and in the presence of NADPH (Table 1). Selected compounds exhibited inhibitory activity in the low micromolar range, with IC_50_ values ranging from 1.95 to 4.44 μM, and higher metabolic stability compared to VS-15, except for compound **17**.

Based on our findings, the following key conclusions can be drawn:

(i) The indole ring in pocket A is essential for IDO1 inhibition. Replacing this moiety with a benzofuran (**14**, 6 ± 7%) lead to a dramatic loss of inhibitory activity compared to VS-15. Elongation of the carbon chain (**12**, 68 ± 15%) results in reduced inhibitory potency, and notably, was associated with an undesirable impact on cell viability. Similarly, the presence of a methyl ester group (**13**, 60 ± 2%) causes a decrease in inhibitory activity, whereas the inclusion of a polar and ionizable functional group (−COOH) renders compounds inactive, likely due to membrane impermeability (**15**, 27 ± 1%).
(ii) Substitution of the indole ring in pocket D with a benzofuran moiety (**19**, 74 ± 3%) or modification of the alkyl chain length (**16**, 80 ± 3%, and **18**, 76 ± 1%) or the incorporation of methyl ester functionality (**17**, 88 ± 10%) was well tolerated, suggesting that the hydrogen bond donor capability of the indole −NH– is not essential for the inhibitory activity. Conversely, the incorporation of a carboxylic function caused a loss in activity (**20**, total loss in activity), probably still due to a detrimental effect on cell permeability. However, none of these modifications resulted in a significant improvement in metabolic stability.
(iii) The replacement of both tryptamine substructures, as in compound **21**, slightly reduces the activity compared to VS-15 but improves metabolic stability, both toward hydrolysis and oxidation (70% of residual substrate in MLM in the absence and presence of NADPH).
(iv) Removal of the carbonyl moiety involved in a hydrogen bond interaction with Ser167 reduces activity (**29**, 57 ± 6%), while, unexpectedly, eliminating the aromatic amine group yields a slightly less active compound (**39**, 90 ± 6%) with greater metabolic stability (94% and 41% residual substrates in MLM in the absence and presence of NAPDH, respectively, vs 5% for VS-15).
(v) Removal of the other carbonyl group (**26**, 61 ± 10%) and amide function (**34**, 46 ± 10%) resulted in reduced activity, suggesting that this functional group may contribute to conformational rigidity and proper orientation of the molecule within the IDO1 active site. Simultaneous substitutions of the indole ring with a benzofuran in pocket D and removal of the aromatic amide moiety are well tolerated (**40**, 93 ± 6%), though oxidation during metabolism assays occurs (8% residual substrate in MLM in the presence of NADPH). Similarly, simultaneous substitutions of the indole ring with a benzimidazole in pocket A, a benzofuran in pocket D, and removal of the aromatic amide group are well tolerated (**41**, 83 ± 4%), also showing increased metabolic stability (95% and 45% residual substrates in MLM in the absence and presence of NADPH, respectively, vs 5% for VS-15).
(vi) Introduction of a urea group in the side chain reduces activity (**66**, 67 ± 4%), but results in a significant enhancement of metabolic stability. Further exploration of compound **66** revealed that adding a methylene unit between the central phenyl ring and the urea functionality decreases activity (**60**, 31 ± 2%), and replacing the indole ring in pocket A with an indoline was unsuccessful (**65**, 22 ± 3%). Decoration of the central phenyl ring with two chlorine atoms (**67**, 65 ± 8%) is well tolerated, while replacing it with a pyridine or a cyclohexyl moiety decreases potency (**61**, 5 ± 0.7%; **62**, 11 ± 0.8%; **63**, 33 ± 3%; and **64**, 41 ± 7%).

Among the explored modifications, replacement of the oxalyl amide with a benzamidourea linker emerged as the most effective strategy to improve metabolic stability while maintaining biological activity. Although compound **66** did not reach the nanomolar potency of clinically advanced catalytic IDO1 inhibitors, it was selected for further characterization because it provided the best overall balance between inhibitory activity and improved metabolic stability. It demonstrated improved resistance to microsomal biotransformation compared to VS-15, particularly showing high stability toward hydrolysis (92% residual substrate in MLM without NADPH). As shown in Scheme 6 and Table S2 of Supporting Information, compound **66** underwent both aliphatic and aromatic oxidation upon activation of the mixed-function oxidase system (67% residual substrate in MLM with NADPH; see Figure S2 of Supporting Information for full spectral data). Notably, bioactivation of the indole moiety to a reactive iminium intermediate was ruled out, as no corresponding GSH adduct was detected. Finally, compound **66** showed poor to negligible *in vitro* CYP inhibition in both human and rodent systems, even at high micromolar concentrations (Figure S3, Supporting Information).

**Scheme 6:**
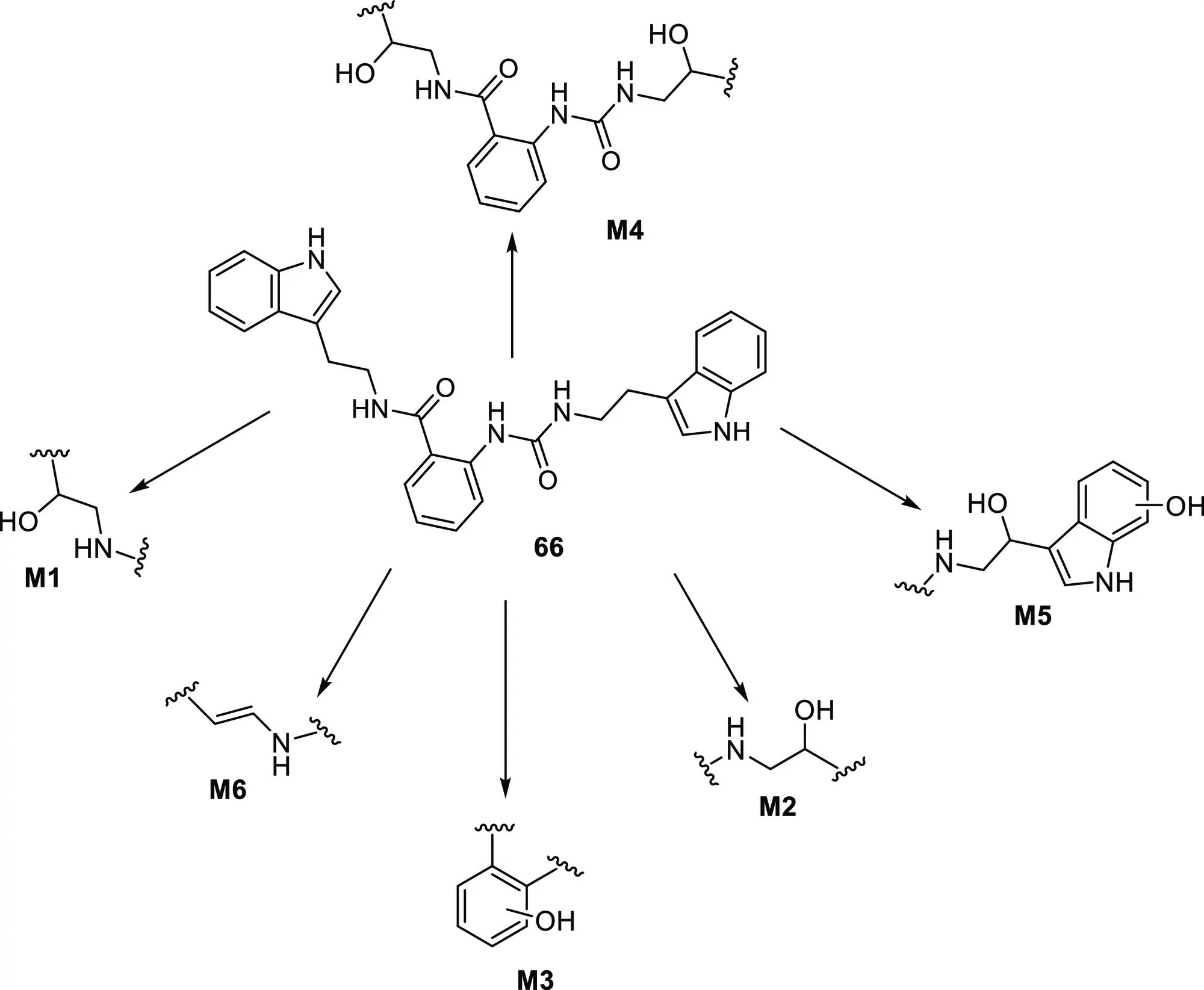
Proposed *In Vitro* Phase I Metabolism Pathway of Compound **66**

### Targeted Metabolomic Profiling and IDO1/TDO Inhibition

Using our previously established LC–MS/MS method for quantitative profiling of the kynurenine pathway,^37^ we performed a targeted metabolomics analysis to assess whether the inhibitory activity of compound **66** translated into coordinated modulation of tryptophan catabolism at the cellular level. The analysis was conducted across a concentration range of compound **66** in three human cancer cell lines selected for their distinct expression patterns of tryptophan-catabolizing enzymes: A375 melanoma cells, which express IDO1 only upon IFNγ stimulation and lack TDO expression,^37^ HepG2 hepatocellular carcinoma and U87 glioblastoma cells, which constitutively express TDO and express IDO only upon IFNγ induction (Figures S4 and S5, Supporting Information). In addition to tryptophan (Trp) and kynurenine (Kyn), downstream metabolites of the kynurenine pathway, including xanthurenic acid (XA), kynurenic acid (KA), 3-hydroxyanthranilic acid (3OHAA), and anthranilic acid (AA), were quantified to evaluate broader pathway modulation under both basal and IFNγ-induced conditions. Heatmaps summarizing metabolite profiles for each cell line are shown in Figure 4, together with dose–response curves of the Kyn/Trp ratio, while principal component analyses (PCAs) and fold changes are reported in the (Supporting Information Figures S6 and S7).

**Figure 4:**
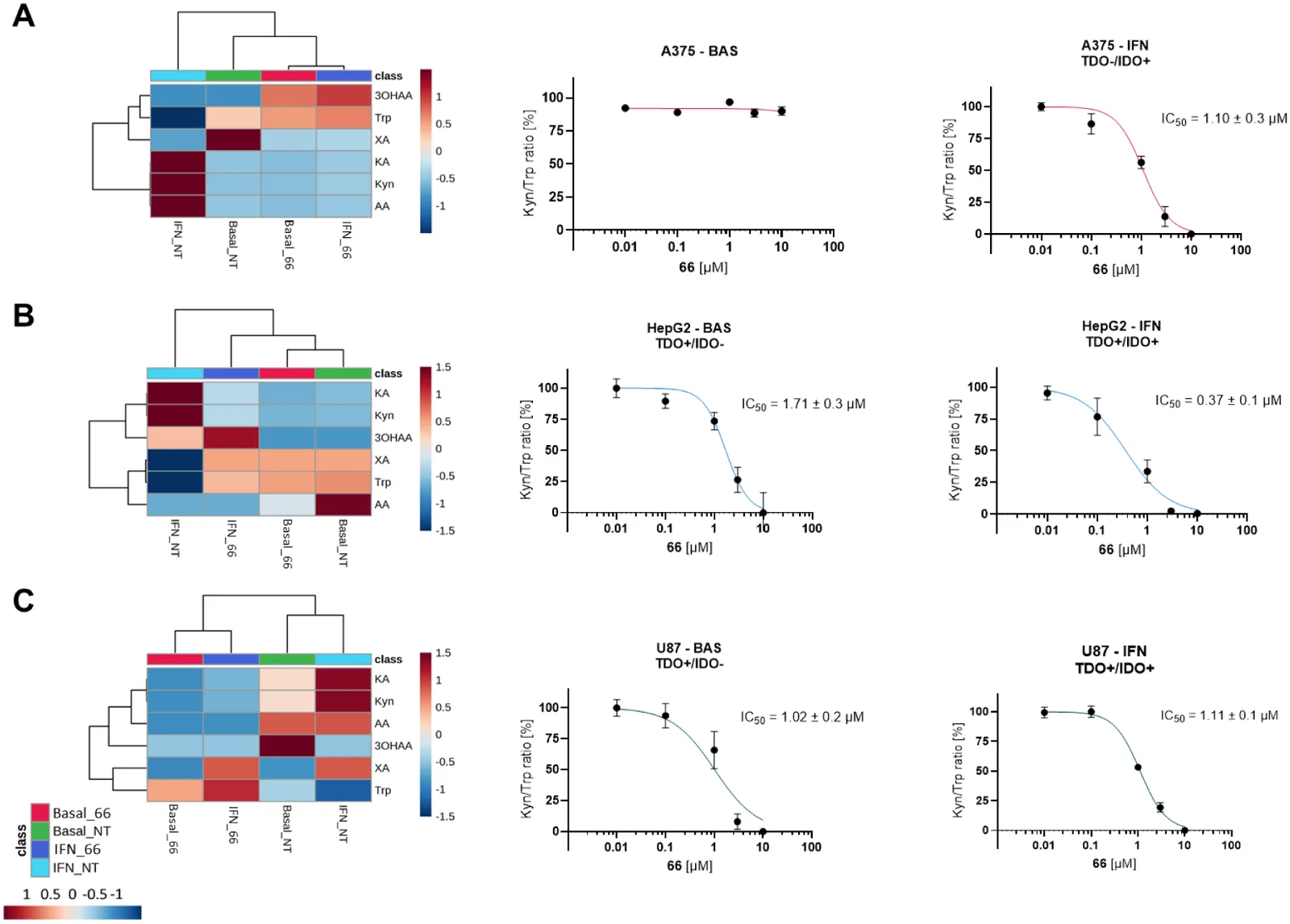
Targeted metabolomic analysis and concentration–response curves of compound **66** in (A) A375, (B) HepG2, and (C) U87 cells. For each cell line, the heatmap shows the mean levels of Kyn, Trp, KA, 3OHAA, and AA across the different experimental conditions, grouped by treatment class, while the corresponding concentration–response curve shows the inhibitory activity of compound **66** under basal and IFNγ-stimulated conditions. IC_50_ plots were generated using the “[Inhibitor] *vs* normalized response” function in GraphPad Prism v. 10.2.3. The *x*-axes represent the logarithm base 10 of the inhibitor concentrations, and the *y*-axes depict the inhibition percentage, calculated as Kyn/Trp ratios and normalized by setting the maximal and basal controls (100% and 0% CTRLs) corresponding to IFNγ treatment and basal conditions, respectively. Error bars represent the Standard Errors of the Mean (SEM). IC_50_ values, calculated by GraphPad Prism v. 10.2.3, are shown alongside the corresponding dose-inhibition curves. Results are the mean ± SD of four independent experiments, each performed in triplicate.

In A375 cells, in which basal Kyn production is negligible and no constitutive TDO-dependent activity was detected under our experimental conditions,^37^ treatment with compound **66** did not reduce Kyn levels under basal conditions (Figure 4A), consistent with the minimal fold changes observed between treated and control samples (Figure S7, Supporting Information). This model therefore provides a clear functional separation between basal conditions, in which no detectable dioxygenase-dependent Kyn production was observed, and IFNγ-induced conditions driven selectively by IDO1. Upon IFNγ stimulation, compound **66** induced a marked reduction in Kyn levels accompanied by accumulation of Trp, consistent with effective inhibition of induced IDO1 activity. The calculated IC_50_ value against IFNγ-induced IDO1 was 1.10 μM (Figure 4A and Table 2), slightly lower than the value previously determined under different experimental conditions (Table 1). In this setting, AA and KA displayed moderate decreases in parallel with Kyn reduction, whereas XA and 3OHAA showed mild increases, indicative of broader modulation of the kynurenine pathway downstream of IDO1 inhibition. In HepG2 cells, which are known to constitutively express TDO,^21,38^ compound **66** significantly reduced Kyn levels in the absence of IFNγ stimulation (Figure 4B), consistent with inhibition of TDO-dependent tryptophan catabolism under basal conditions. In HepG2 cells, Kyn levels decreased with a log_2_ fold change of −0.98, accompanied by pronounced reductions in KA (−1.47) and AA (−1.83) (Figures S6 and S7, Supporting Information). These coordinated changes indicate that, in TDO-driven cellular contexts, inhibition by compound **66** propagates beyond Kyn formation and affects downstream branches of the pathway, likely as a consequence of reduced substrate flux. In U87 cells,^39,40^ compound **66** also reduced basal Kyn levels (Figure 4C). To clarify the enzymatic source contributing to basal Kyn production in this model, we evaluated both IDO1 and TDO protein expression under our experimental conditions. Western blot analysis showed detectable TDO protein in U87 cells, using SW-48 and A375 cells as positive and negative controls, respectively, whereas IDO1 was not detectable under basal conditions and was only induced following IFNγ stimulation (Figures S4 and S5, Supporting Information). Consistently, the selective TDO inhibitor 680C91 inhibited basal Kyn production in U87 cells (Figure S8, Supporting Information). These data indicate that the reduction of basal Kyn levels by compound **66** in U87 cells is most consistent with inhibition of TDO-dependent tryptophan catabolism. In U87 cells, Kyn reduction (log_2_ fold change −0.63) was accompanied by a marked increase in Trp levels (log_2_ fold change +1.89), whereas downstream metabolite changes were less pronounced, reflecting differences in pathway regulation between the two cell lines (Figures S6 and S7, Supporting Information).

**Table 2:** Activity of Compound **66** on Different Cell Lines

Cell line	IC_50_ (μM) IFNγ (−)	IC_50_ (μM) IFNγ (+)
**A375**	-	1.10 ± 0.3
**HepG2**	1.71 ± 0.3	0.37 ± 0.1
**U87**	1.02 ± 0.2^a^	1.11 ± 0.1^b^
**P1.hIDO1**	2.90 ± 0.3	-
**P1.mIDO1**	1.47 ± 0.02	-
**P1.hTDO**	0.42 ± 0.3	-
**P1.mTDO**	13.11 ± 2.2	-

a 680C91. IC_50_ = 0.275 ± 0.033 μM.

b IDO/TDO-IN-1. IC_50_ = 0.260 ± 0.132 μM (see Figure S8, Supporting Information).

Upon IFNγ induction, which enhances IDO1 expression in both HepG2 and U87 cells (Figure S4, Supporting Information), compound **66** maintained its inhibitory activity. In HepG2 cells, the IC_50_ value decreased from 1.71 μM under basal conditions to 0.37 μM following IFNγ treatment (Figure 4B and Table 2), consistent with enhanced apparent activity under IDO1-induced conditions. In contrast, U87 cells displayed comparable IC_50_ values under basal and IFNγ-induced conditions (1.02 vs 1.11 μM; Figure 4C and Table 2), suggesting that compound **66** retains similar potency irrespective of IDO1 induction. Collectively, these results highlight a cell-type-dependent balance between IDO1 and TDO inhibition by compound **66**.

To further validate dual IDO1/TDO inhibition independently of cell-specific regulatory mechanisms, the activity of compound **66** was evaluated in a controlled cellular system expressing individual dioxygenases. The P1.HTR murine mastocytoma cell line, which lacks endogenous IDO1 and TDO expression, was stably transfected with constructs encoding either human or murine IDO1 (P1.hIDO1, P1.mIDO1) or TDO (P1.hTDO, P1.mTDO). In this system, compound **66** inhibited both IDO1 and TDO in a dose-dependent manner, yielding IC_50_ values ranging from 0.42 μM to 13.11 μM (Table 2 and Figure S9, Supporting Information). Notably, inhibition was particularly balanced against the human enzymes, further supporting the classification of compound **66** as a dual IDO1/TDO inhibitor.

Together, these metabolomic and cellular data support coordinated and context-dependent inhibition of the kynurenine pathway by compound **66**, consistent with functional dual IDO1/TDO engagement across complementary biochemical and cellular models.

### Apo-IDO1 and TDO Inhibition

To define the mode of IDO1 inhibition exerted by compound **66**, we employed a combination of spectroscopic, enzymatic, and cell-based assays specifically designed to discriminate between *holo*- and *apo*-IDO1 targeting. *Holo*-IDO1 contains a heme prosthetic group coordinated to its iron center, which gives rise to a characteristic absorption peak at approximately 404 nm, the Soret band. To evaluate whether compound **66** can interact with and displace the heme cofactor, thus acting as an *apo*-IDO1 inhibitor, we employed a spectrophotometric assay in the presence of the *holo*-recombinant protein (Figure S10, Supporting Information), a well-established readout of heme displacement.^24^ Following a 15 min preincubation at 37 °C, the absorbance at 404 nm was continuously recorded for 120 min after compound addition. A time-dependent decrease in Soret absorbance was observed for **66** and linrodostat, indicative of heme displacement and consistent with their *apo*-inhibitory mechanism, while it was absent for the *holo*-inhibitor epacadostat (Figure 5A and Figure S11, Supporting Information).

**Figure 5:**
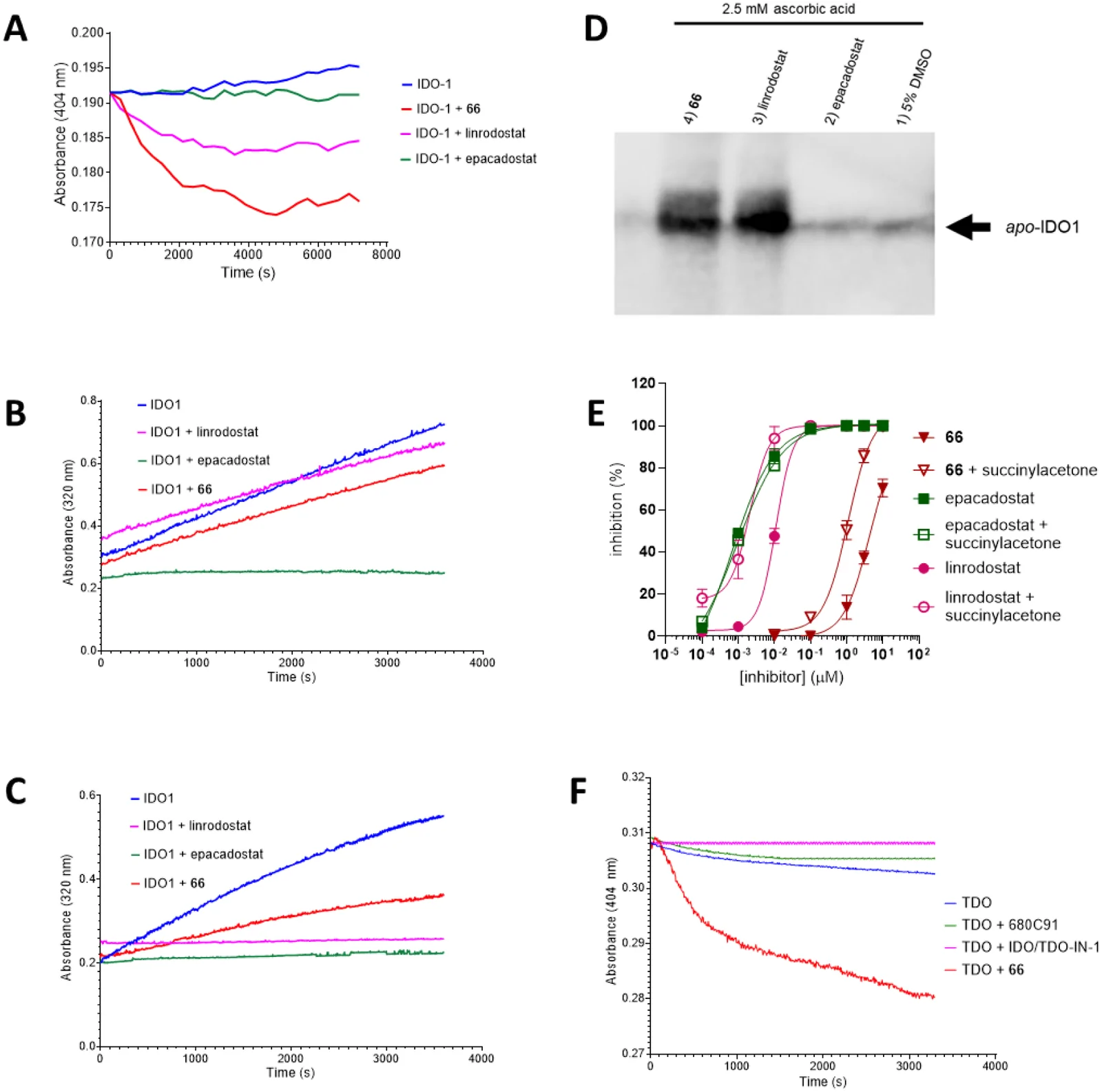
Biochemical and cell-based characterization of compound **66** against IDO1 and TDO. A) Biochemical spectrophotometric assay of IDO1. Tracking the loss of absorbance at the IDO1 Soret peak (λ max = 404 nm) over time, in the absence (blue) and presence of compound **66** (red), linrodostat (magenta) and epacadostat (green) at 50 μM (*n* = 2; each concentration tested in technical triplicate). B) and C) Biochemical enzymatic assay of recombinant IDO1. IDO1 activity was monitored in the presence of 25 μM linrodostat, epacadostat, or compound **66** at 25 °C (B) and 37 °C (C); (*n* = 2; each concentration tested in technical triplicate). D) Biochemical hemin–agarose assay for *apo*-IDO1 detection in supernatants following incubation with hemin–agarose resin and inhibitors. Western blot performed using a monoclonal anti-IDO1 antibody (clone EPR20374) shows the amount of *apo*-IDO1 released into the final supernatant after treatment with 1) 10 mM ascorbic acid +5% DMSO; 2) 10 mM ascorbic acid +5% DMSO + 1 mM epacadostat; 3) 10 mM ascorbic acid +5% DMSO + 1 mM linrodostat; 4) 10 mM ascorbic acid +5% DMSO + 1 mM compound **66**. E) Cellular-based IDO1 inhibition assay. Cellular activity of epacadostat, linrodostat and compound **66** in the presence and absence of succinylacetone. Results are the mean ± SD of 3 independent experiments, each performed in triplicate. F) Biochemical spectrophotometric assay of TDO. Tracking the loss of absorbance at the TDO Soret peak (λ max = 404 nm) over time, in the absence (blue) and presence of 680C91 (green), IDO/TDO-IN-1 (magenta), and compound **66** (red) at 25 μM at 37 °C (*n* = 2; each concentration tested in technical triplicate).

To further validate the *apo*-inhibition mechanism of compound **66**, we employed a biochemical assay designed to distinguish between *holo*- and *apo-*IDO1 inhibition under permissive versus forced conditions (Figures 5B and 5C, and Figure S12, Supporting Information). The assay monitors the enzymatic conversion of Trp to *N*-formylkynurenine (NFK) by spectrophotometric detection at 320 nm (ε = 3100 M^–1^ cm^–1^), a standard approach for assessing IDO1 catalytic activity. It is based on previously reported protocols for identifying *apo*-IDO1 inhibitors, notably those described by Ortiz-Meoz et al., who demonstrated that extended preincubation times and physiological temperatures promote the conversion of *holo*-IDO1 to its *apo*-form, thereby enabling selective *apo*-binders to act effectively.^24^ To favor binding to the *holo*-form of the enzyme, the assay was first performed at 25 °C without preincubation of the inhibitor prior to substrate addition. Under these conditions, compound **66** and linrodostat failed to inhibit the enzyme, while epacadostat completely suppressed enzymatic activity (Figure 5B). In contrast, when the assay was carried out under forced conditions (i.e., at 37 °C with a prolonged 120 min preincubation period before substrate addition), both **66** and linrodostat were able to inhibit IDO1 activity at 25 μM, similarly to epacadostat (Figure 5C). This differential behavior under permissive versus forced conditions provides functional evidence that compound **66** does not directly inhibit *holo*-IDO1 but requires conversion to the *apo*-form to exert activity. The IC_50_ of **66** was also determined under these conditions, affording a value of 26.54 μM (Figure S13, Supporting Information). The apparent IC_50_ determined under forced conditions reflects the kinetics of heme displacement rather than intrinsic binding affinity and is therefore not directly comparable to IC_50_ values obtained for *holo*-IDO1 inhibitors. The *apo*-inhibitor linrodostat and the dual *holo*-IDO1/TDO inhibitor IDO/TDO-IN-1 were included as reference compounds (Figures S13 and S15, Supporting Information). These results are consistent with a mechanism in which compound **66** preferentially binds to and stabilizes the *apo*-conformation of IDO1, requiring time and elevated temperature to displace the heme cofactor and exert its inhibitory effect.

Finally, a hemin–agarose pull-down assay further corroborated the *apo*-inhibition mechanism of compound **66**. The results of hemin-agarose affinity chromatography followed by SDS-PAGE and immunoblot analysis of the final supernatants with an IDO1–specific antibody showed that compound **66** or linrodostat (1 mM) prevented IDO1 from binding to immobilized hemin, resulting in the recovery of the enzyme in the supernatant, consistent with stabilization of the *apo* form. By contrast, no comparable effect was observed upon treatment with epacadostat (Figure 5D).

To assess whether the *apo*-selective inhibition observed in biochemical assays translated to a cellular context, we next evaluated the activity of compound **66** in IDO1-expressing cells. To recreate the optimal environment for *apo*-IDO inhibitors in a cell-based assay, we treated cells with succinylacetone, a δ-aminolevulinic acid dehydratase inhibitor that blocks the first enzyme in the heme biosynthesis pathway. This treatment depletes cellular heme supplies, ensuring that IDO1 exists predominantly in its *apo*-form, thereby shifting the *holo*/*apo* balance toward the *apo*-form. A375 cells were stimulated with IFNγ in the presence or absence of succinylacetone and treated with increasing concentrations (0.01–10 μM) of **66** for 48 h. The presence of succinylacetone significantly reduced Kyn levels across all **66** concentrations tested, inducing a leftward shift in the concentration–response curve for **66**. Consequently, the calculated IC_50_ shifted from 4.04 ± 0.7 μM under normal conditions to 1.08 ± 0.1 μM in the presence of succinylacetone (Figure 5E). As a positive control, linrodostat was also tested under both conditions and a similar leftward shift in the concentration–response curve was observed. In contrast, no change was noted when epacadostat was tested. These findings indicate that **66** exhibits behavior consistent with linrodostat and support an *apo*-IDO1 targeting profile.

Like IDO1, *holo*-TDO harbors a heme prosthetic group that gives rise to the characteristic Soret absorption band at 404 nm. To assess whether compound **66** could inhibit TDO via an *apo*-binding mechanism, a spectrophotometric assay was performed by monitoring changes in absorbance at the Soret maximum. Upon incubation of recombinant human *holo*-TDO with **66** at 25 μM and 37 °C for 15 min, a notable reduction in the 404 nm peak was observed (Figure 5F). This decrease suggests that **66** interferes with heme binding, either by displacing the prosthetic group or by preventing its association, consistent with a mode of action involving *apo*-TDO inhibition. Under these conditions, an apparent IC_50_ value of 15.63 μM was determined for compound **66**. As with IDO1, this value is not directly comparable to the IC_50_ values of selective *holo*-TDO inhibitor 680C91 ^41^ and the dual *holo*-IDO1/TDO inhibitor IDO/TDO-IN-1, used as reference compounds (Figures S14 and S16, Supporting Information), but rather reflects an inhibitory mechanism involving heme displacement.

### Docking Pose of **66** in Apo-IDO1 and Apo-TDO

Although IDO1 and TDO catalyze the same oxidative cleavage of Trp, their active sites show distinct architectures, especially in their *apo* forms, where the absence of the heme cofactor alters both geometry and polarity. In *apo*-IDO1, the active site is more solvent-accessible and features well-defined hydrophobic and polar pockets, whereas *apo*-TDO retains a more compact and hydrophobic environment shaped by highly conserved aromatic residues. These differences are crucial in determining ligand orientation and binding affinity, particularly for compounds like **66** that may act as dual inhibitors.

To provide a structural rationale for the dual *apo*-IDO1/TDO inhibitory profile of compound **66**, docking studies were performed. The FRED software was used, and potential water-mediated interactions were further investigated using SZMAP. In the docking pose within *apo*-IDO1 (Figure 6A), compound **66** is positioned across pocket A and pocket D. The indole group adjacent to the urea moiety is oriented toward pocket A, forming π-contacts with Phe163. The urea nitrogen atoms engage in water-bridged hydrogen bonding with His346, as highlighted by SZMAP analysis. The central aromatic ring is placed near Ala264, at the entrance of pocket A, while the second indole ring penetrates deep into a hydrophobic subpocket of pocket D, formed by Phe270, Leu342, and Val269. Additionally, the indole nitrogen forms a hydrogen bond with the backbone carbonyl of Val170. Notably, the overall orientation of **66** is opposite to that observed for VS-15 (Figure 6C), as reported previously,^30^ suggesting a different anchoring strategy in the *apo*-conformation and highlighting how subtle linker modification alters binding orientation and flexibility within the *apo* pocket.

**Figure 6:**
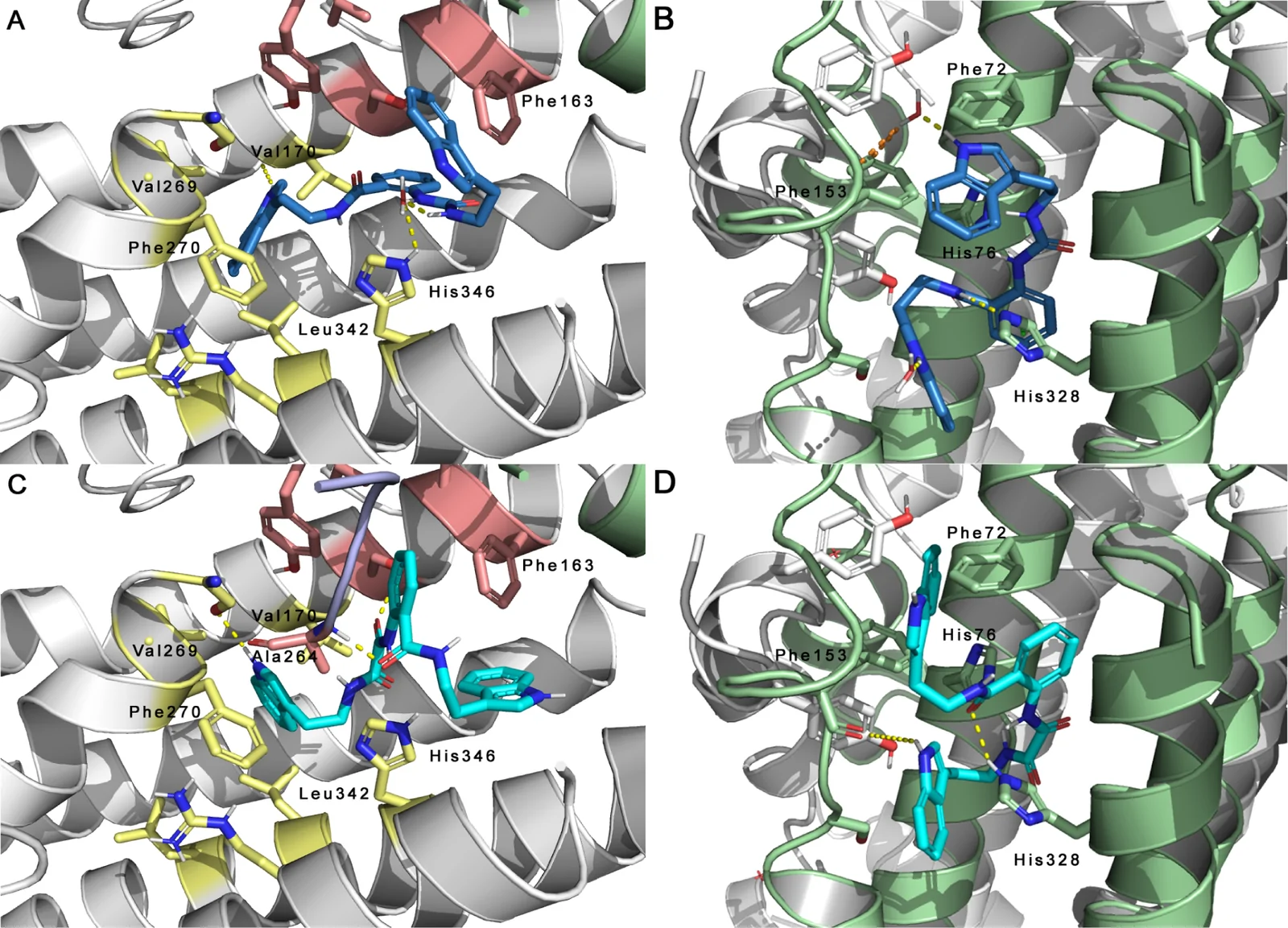
Docking poses of compounds **66** and VS-15. Amino acids discussed in the main text are shown and labeled. (A) Binding pose of **66** within the active site of *apo*-IDO1 (PDB ID: 7B1O); Ala264 has been omitted for clarity, as it occluded the view of the ligand. (B) Binding pose of **66** within the active site of *apo*-TDO (PDB ID: 8QV7). (C) Binding pose of VS-15 within the active site of *apo*-IDO1 (PDB ID: 7B1O). (D) Binding pose of VS-15 within the active site of *apo*-TDO (PDB ID: 8QV7). In *apo*-IDO1 (A, C), residues are colored according to their location within binding subpockets: salmon for pocket A, green for pocket B, and yellow for pocket D (pocket C is not visible in this orientation). In *apo*-TDO (B, D), which functions as a tetramer, the subunit contributing most significantly to the binding pocket is highlighted in light green, while the remaining subunits are shown in grayscale.

Conversely, in *apo*-TDO (Figure 6B), compound **66** adopts a distinct binding mode. Due to the tetrameric nature of TDO, the binding pocket is formed at the interface of multiple subunits rather than being confined to a single one. The first indole group occupies a hydrophobic cleft defined by Phe153 and Phe72, while the urea moiety orients toward His76, stabilizing the central aromatic ring near His328. This heterocyclic system engages in both π–π stacking interactions and a hydrogen bond via the nitrogen. The second indole group is partially solvent-exposed, yet remains in proximity to His328, potentially contributing to binding stability through secondary contacts (Gln154, Ser155, Phe158, and Trp324). Despite the different architectures of *apo*-IDO1 and *apo*-TDO, compound **66** is able to engage both targets through distinct but energetically favorable binding modes, providing a structural basis for its balanced dual inhibitory activity.

Despite only subtle 2D structural changes, VS-15 evolved into **66**, a dual *apo*-IDO1/TDO inhibitor. To unravel the structural basis of this transformation, we examined the binding modes of the ligands, their intrinsic 3D shapes, and the architectures of the corresponding *apo* pockets of both IDO1 and TDO. A 3D alignment in vacuo confirmed that VS-15 and **66** are highly similar and share an almost perfectly overlapping terminal moiety. However, replacement of the oxalyl amide in VS-15 with the benzamidourea linker in **66** removes one carbonyl group, disrupts the pseudosymmetry of the scaffold, and relaxes the conformational constraint imposed by the original oxalyl unit. As a result, both ligands can adopt an alternative binding mode characterized by an approximately 180° flipped orientation in the *apo* forms of IDO1 and TDO, enabling the recovery of optimal hydrophobic and hydrogen-bonding interactions (Figure 6A and 6B versus [Fig fig6]C and 6D). Thus, a seemingly modest chemical modification exerts a disproportionate effect on the three-dimensional conformational space accessible to the ligands within the *apo* binding pockets.

Consistent with the experimental evidence of *apo*-enzyme targeting, docking was performed using *apo*-IDO1 and *apo*-TDO structures, in which the absence of the heme cofactor exposes distinct binding cavities. An FPocket-based shape analysis^42,43^ revealed that the *apo*-TDO cavity is more compact and nearly spherical (NPR1 ≈ 0.65, NPR2 ≈ 0.87; Figure S17, Supporting Information), whereas the *apo*-IDO1 cavity is more elongated (NPR1 ≈ 0.40, NPR2 ≈ 0.86; Figure S17, Supporting Information); a similar trend was observed for the *holo* forms (*holo*-TDO: NPR1 ≈ 0.90, NPR2 ≈ 0.96; *holo*-IDO1: NPR1 ≈ 0.58, NPR2 ≈ 0.86; Figure S17, Supporting Information). In this context, the slightly increased flexibility and altered mass distribution of **66** relative to the more rigid VS-15 are expected to facilitate a flipped binding orientation that better complements the more spherical *apo*-TDO pocket, while still supporting productive interactions within the elongated *apo*-IDO1 cavity.

### Inhibition of Pro-Tumorigenic IDO1 Signaling in SKOV-3 Cells

Stabilization of a nonenzymatic, signaling-competent conformation of IDO1 by catalytic inhibitors, including linrodostat, has recently been demonstrated in the human ovarian cancer cell line SKOV-3, as well as in other human cancer cell lines that endogenously express IDO1.^10,11^ On this basis, SKOV-3 cells were selected as a suitable model to investigate the functional behavior of compound **66**, as these cells predominantly contain IDO1 in its *apo*-conformation.^2^

As expected, compound **66** significantly reduced Kyn release in SKOV-3 cells after 16 h of treatment, indicating effective inhibition of IDO1 catalytic activity. The extent of Kyn reduction was comparable to that observed with the catalytic inhibitor linrodostat (Figure 7A).

**Figure 7:**
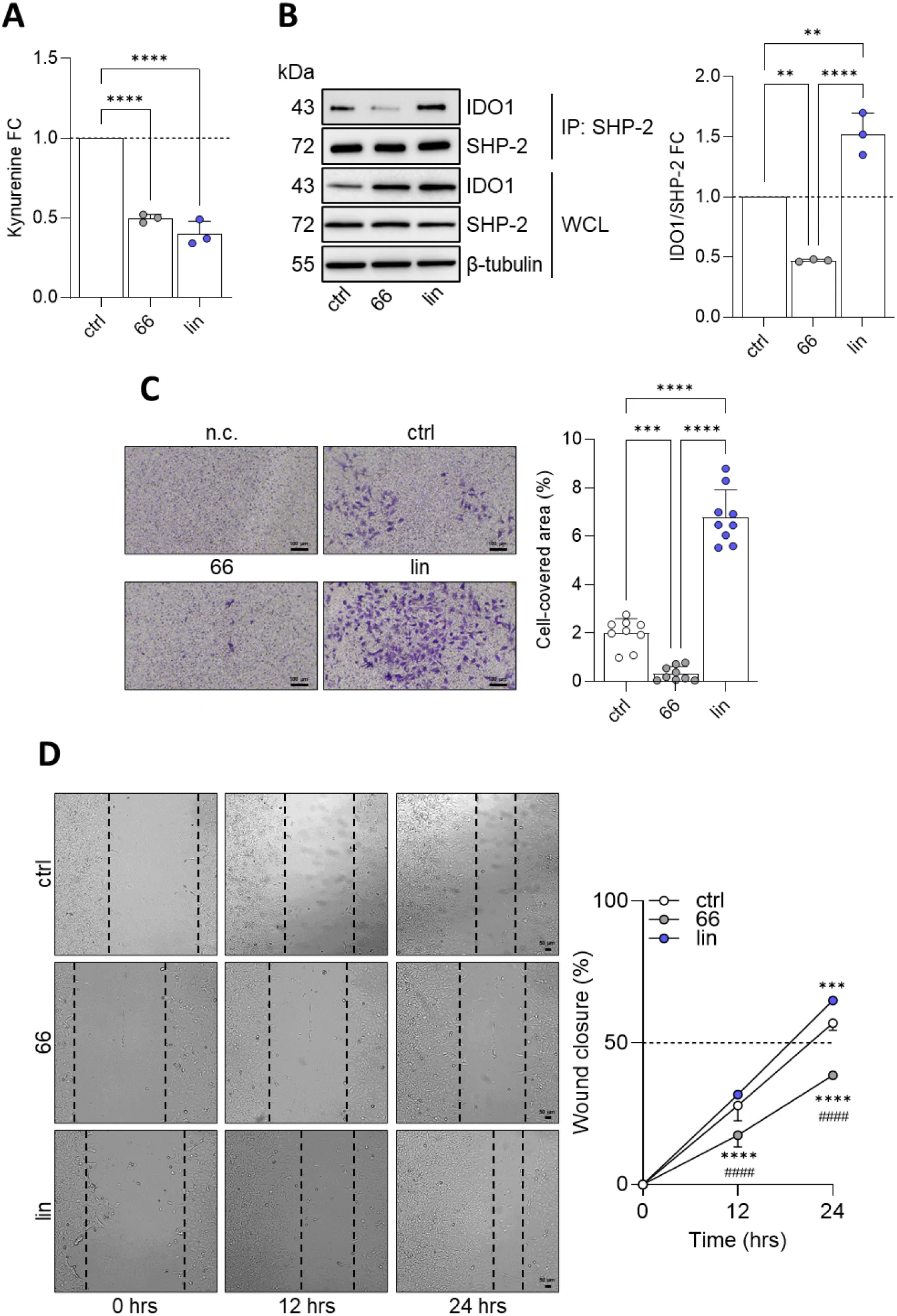
(A) Kynurenine released by SKOV-3 treated with compound **66** (10 μM) or linrodostat (lin, 1 μM) for 16 h. Vehicle-treated cells were used as control (ctrl). Results are shown as kynurenine fold change (FC) of treated versus ctrl cells (dotted line, 1-fold). (B) SHP-2 immunoprecipitation (IP) followed by SHP-2 and IDO1 immunoblot analysis performed in SKOV-3 cells treated as in (A). Whole cell lysate (WCL) was used as control of input protein expression, whereas β-tubulin expression was used as normalizer. In the left panel, one representative immunoblot of three is shown. In the right panel, the quantitative data from densitometric analysis (IDO1/SHP-2 ratio calculated in IP samples) are reported as FC of treated versus ctrl cells (dotted line, 1-fold). (C) Transwell migration assay performed in SKOV-3 cells exposed to compound **66** (10 μM) or lin (1 μM) in serum-free medium and cultured over complete medium (10% FCS) for 24 h. Vehicle-treated cells were used as ctrl, while vehicle-treated cells cultured over serum-free medium were included as a negative ctrl (n.c.). For each condition, a representative field of view is shown (10× magnification; scale bar: 100 μm) and cell migration is reported as percentage (%) of cell-covered area, calculated in each field of view. (D) Over time analysis of the wound closure (black dotted lines) performed in SKOV-3 cells in the presence of compound **66** (10 μM), lin (1 μM), or vehicle alone (ctrl). For each reported time point, one representative image is shown (10× magnification; scale bar: 50 μm) and data are reported as wound closure percentage (%) of respective time 0 (time 0 = 0%; dotted line, 50%). Data in (A, B, C, D) are mean ± SD of three independent experiments and were analyzed by one-way (A, B, C) or two-way (D) ANOVA followed by posthoc Bonferroni’s test. In (D), statistical analysis of treated versus ctrl cells is indicated by *, whereas comparison between **66**- and lin-treated cells is indicated by ^#^. **P < 0.01, ***P < 0.001, ****P < 0.0001, ^####^ *P* < 0.0001.

Beyond enzymatic inhibition, catalytic IDO1 inhibitors have been shown to promote stabilization of a nonenzymatic IDO1 conformation that is competent for pro-tumorigenic signaling, including activation of SHP-2–dependent pathways.^11^ SHP-2 is a well-established oncogenic tyrosine phosphatase that functions as a central signaling node downstream of multiple growth factor receptors and is critically involved in regulating cancer cell proliferation and migration.^44–46^ Engagement of SHP-2 by signaling-competent IDO1 therefore represents an early and functionally relevant molecular event in tumor cells.^10^ To assess whether compound **66** modulates this signaling axis, we evaluated its effect on the interaction between IDO1 and SHP-2 in SKOV-3 cells.

Notably, treatment with compound **66** reduced the association between IDO1 and SHP-2 in SKOV-3 cells compared with both vehicle-treated and linrodostat-treated controls (Figure 7B). In P1.HTR cells stably expressing mouse IDO1, compound **66** similarly reduced IDO1/SHP-2 coimmunoprecipitation and the phosphatase activity associated with immunoprecipitated IDO1. Subcellular fractionation further showed reduced levels of both IDO1 and SHP-2 in the Rab5-positive EE fraction and a corresponding increase in the cytosolic fraction after treatment with compound **66**.^8^ (Figure S18, Supporting Information). These findings are consistent with attenuation of IDO1-associated signaling and altered subcellular distribution of both IDO1 and SHP-2 upon treatment with compound **66**.

Given that SHP-2 engagement by IDO1 represents an early molecular event driving SKOV-3 cell migration,^10^ we next examined the functional consequences of disrupting this interaction. Using both transwell migration and wound-closure assays, treatment with compound **66** significantly reduced the percentage of migrated SKOV-3 cells compared to vehicle-treated and linrodostat-treated controls (Figures 7C and 7D). These effects were fully consistent with the observed inhibition of IDO1/SHP-2 complex formation.

Overall, these results indicate that compound **66**, by attenuating the IDO1/SHP-2 signaling axis, counteracts the pro-tumorigenic effects associated with stabilization of signaling-competent IDO1 conformations by conventional catalytic inhibitors.

## Conclusions

Recent advances have revealed the dual nature of IDO1, which exists in a dynamic equilibrium between an enzymatically active, heme-bound *holo*-form and a heme-free *apo*-form that exerts noncatalytic signaling functions implicated in immune modulation and tumor progression. While most IDO1 inhibitors developed to date selectively target the *holo*-enzyme through coordination of the heme–iron complex, increasing evidence supports the therapeutic relevance of *apo*-IDO1, particularly in tumor contexts where this form predominates and contributes to immune escape through protein–protein interactions with signaling mediators such as SHP phosphatases.

In this framework, we previously identified VS-15 as a small molecule capable of selectively modulating *apo*-IDO1 by disrupting its nonenzymatic interactions. However, its poor metabolic stability, primarily associated with the oxalyl amide moiety, limited its translational potential. To overcome this limitation, we undertook a SAR campaign aimed at improving metabolic stability while preserving *apo*-IDO1 selectivity.

This effort led to the identification of compound **66**, featuring a novel *ortho*-benzamide urea scaffold, as a metabolically stable analogue retaining low micromolar potency. Among a focused library of 24 newly synthesized compounds, **66** emerged as the most promising candidate, displaying balanced dual inhibition of IDO1 and TDO in cell-based assays (IC_50_ = 2.90 μM for P1.hIDO1; IC_50_ = 0.47 μM for P1.hTDO) and selectivity for the *apo*-form of both enzymes.

The dual inhibitory profile of compound **66** was further validated by targeted cellular metabolomics. In A375 cells, which lack basal expression of IDO1 and TDO, **66** was inactive under basal conditions but effectively inhibited IFNγ-induced IDO1 (IC_50_ = 1.10 μM). Conversely, in HepG2 and U87 cells, where TDO is constitutively expressed, **66** reduced kynurenine levels under basal conditions (log_2_ fold change −0.98 and −0.63, respectively), consistent with primary TDO inhibition. Cellular IC_50_ values of 1.71 μM (HepG2) and 1.02 μM (U87) further decreased to 0.37 μM in IFNγ-treated HepG2 cells, indicating enhanced inhibitory potency in the presence of both IDO1 and TDO. Collectively, these data support a cell-type-dependent dual inhibitory action of compound **66** on immunosuppressive tryptophan catabolism and its potential as a broad-spectrum modulator of the kynurenine pathway. Notably, compound **66** also reduced levels of KA, a kynurenine pathway metabolite known to contribute to the establishment of an immunosuppressive TME.^47^

Mechanistic studies further clarified the mode of action of compound **66**. Spectrophotometric Soret assays revealed a progressive reduction of the characteristic 404 nm absorption peak of both IDO1 and TDO upon incubation with **66**, consistent with heme displacement and *apo*-enzyme binding, as previously reported for linrodostat and the first described *apo*-TDO inhibitor.^48^ This mechanism was corroborated by enzymatic assays under forced conditions, in which inhibition required prolonged preincubation, confirming the inability of compound **66** to directly bind the *holo*-form.

Molecular docking studies provided a structural rationale for *apo*-enzyme targeting and dual activity. In *apo*-IDO1, compound **66** spans pockets A and D, engaging a deep hydrophobic cleft through key interactions with residues including Phe163, His346, Val170, and Phe270. In *apo*-TDO, the compound adopts a distinct binding mode compatible with the different architecture of the pocket, supporting its cross-reactivity toward both targets. Comparative analysis with the previously reported VS-15 indicated that subtle modification of the central linker alters the balance between conformational rigidity and flexibility within the *apo* pockets, rationalizing the transition from selective *apo*-IDO1 inhibition to a dual *apo*-IDO1/TDO profile.

Importantly, compound **66** also suppressed the intrinsic pro-tumorigenic signaling function of IDO1 in SKOV-3 cells, as evidenced by reduced engagement of the SHP-2 oncogene and consequent inhibition of tumor cell migration. This tumor-intrinsic IDO1 signaling pathway has recently been shown to be activated by clinically advanced catalytic IDO1 inhibitors, including epacadostat, navoximod, and linrodostat, thereby representing an adverse consequence of selective enzymatic inhibition.^11^ In addition, compound **66** attenuated the interaction between SHP-2 and murine IDO1 in P1.HTR cells and altered the subcellular distribution of both IDO1 and SHP-2, further supporting attenuation of IDO1-associated signaling activity.

On a mechanistic level, two nonmutually exclusive hypotheses may explain how compound **66** interferes with IDO1/SHP-2 signaling. First, recent biophysical and computational studies have demonstrated that ligand binding to the catalytic cleft of IDO1 can encode distinct conformational and dynamic states that propagate to distal regions of the protein, including surfaces involved in the recruitment of signaling partners.^49^ In this framework, binding of **66** to the heme-free *apo*-form may stabilize a specific conformational ensemble of IDO1 that is less permissive to SHP-2 association by reshaping the geometry and/or electrostatic properties of the protein–protein interaction interface. Second, structural modeling and molecular dynamics analyses of phosphorylated *apo*-IDO1 have suggested that engagement of SHP-2 involves insertion of its C-terminal region into the heme-binding pocket, an interaction that is favored only in specific open-pocket conformations.^50^ Although based on computational predictions, these observations raise the possibility that occupation of the *apo* catalytic pocket by **66** may destabilize or sterically interfere with this mode of SHP-2 binding. Together, these hypotheses provide a plausible structural framework for the selective reduction of IDO1/SHP-2 signaling observed for compound **66** and illustrate how *apo*-IDO1 ligands may differentially modulate enzymatic and nonenzymatic functions of the protein.

The dual modality of IDO1 inhibition exhibited by compound **66**, encompassing *apo*-enzyme targeting, interference with enzymatic activity, and suppression of tumor-specific signaling, defines a differentiated pharmacological profile compared with conventional single-mode inhibitors. Together with its activity across distinct cellular contexts, these features establish compound **66** as a robust chemical probe and support its further use in preclinical studies aimed at dissecting and modulating the kynurenine pathway in cancer. In this context, recent plasma metabolomic studies in ovarian cancer patients have reported systemic dysregulation of the kynurenine pathway, underscoring the clinical relevance of strategies aimed at controlling pathway output rather than isolated enzymatic steps.^37^

These findings should be interpreted in light of some remaining mechanistic and translational aspects that warrant further investigation. Compound **66** currently displays micromolar potency; nevertheless, the combined biochemical, spectroscopic, cellular, and computational data support a dual IDO1/TDO profile consistent with *apo*-enzyme targeting and attenuation of IDO1/SHP-2 signaling. Future studies aimed at obtaining orthogonal structural or biophysical validation of *apo-*enzyme engagement, defining the functional contribution of TDO inhibition in cellular systems, genetically validating SHP-2-dependent downstream signaling, assessing dendritic-cell immunomodulation, and evaluating activity in in vivo tumor models will further define the scope of this approach.

Altogether, this work introduces compound **66** as a metabolically improved *ortho*-benzamidourea derivative with a functional dual IDO1/TDO inhibitory profile and the ability to attenuate IDO1-associated noncatalytic signaling in cancer-relevant cellular models. These findings support small-molecule modulation of the IDO1/TDO–kynurenine axis as a complementary strategy to purely catalytic IDO1 inhibition or complete IDO1 degradation, and provide a chemical probe for further dissecting the interplay between enzymatic and signaling functions of IDO1 in tumor biology.

## Experimental Section

### Chemistry

#### General Information

Reagents were purchased from Sigma-Aldrich (MO, USA), TCI Europe (Zwijndrecht, Belgium), and BLD-Pharm (Hamburg, Germany). Epacadostat was purchased from Selleckchem (Huston, TX, USA), and linrodostat was purchased from D.B.A. ITALIA S.R.L. (Milan, Italy).

Commercially available reagents and solvents were used as purchased without further purification. When needed, solvents were distilled and stored on molecular sieves. Column chromatography was performed through puriFlash XS520 Plus. Thin layer chromatography (TLC) was carried out on 5 × 20 cm aluminum plates with a layer thickness of 0.25 cm, coated with silica gel (Merck Kieselgel 60, 230–400 mesh-ASTM). When necessary, TLC plates were visualized with aqueous KMnO_4_ or with Ehrlich solution. Purification was performed using flash or gravimetric chromatography on silica gel (Merck Kieselgel 60, 230–400 mesh-ASTM or 70–230 mesh-ASTM) or using puriFlash XS520 Plus. Melting points were determined in an open glass capillary with a Stuart scientific SMP3 apparatus. All the target compounds were checked by IR (FT-IR Bruker Alpha II), ^1^H NMR and ^13^C NMR (Bruker Avance Neo 400 MHz), mass spectrometry (Thermo Finningan LCQ-deca XP-plus) equipped with an ESI source and an ion trap detector, and HRMS (Thermo Fisher Q-Exactive Plus) equipped with an Orbitrap (ion trap) mass analyzer. All intermediate compounds were checked by ^1^H NMR and mass spectrometry. Chemical shifts are reported in parts per million (ppm). The chemical shifts were referenced with respect to the nondeuterated residual solvent signal (DMSO-*d*_6_: 2.50 ppm for ^1^H and 39.5 ppm for ^13^C; acetone-*d*_6_: 2.05 ppm for ^1^H and 29.84 ppm for ^13^C; CDCl_3_: 7.26 ppm for ^1^H and 77.16 ppm for ^13^C). Data are reported as follows: chemical shift (δ), multiplicity (s = singlet, d = doublet, t = triplet, q = quartet, br s = broad singlet, dd = doublet of doublets, dt = doublet of triplets, td = triplet of doublets, tt = triplet of triplets, qt = quartet of triplets, sxt = sextet, m = multiplet), coupling constants (*J*, Hz) and number of protons. The purity of selected compounds was determined by HPLC using a Shimadzu HPLC system LC-10AD series equipped with a Synergi Polar (150 × 4.6 mm, 4 μm d_*p*_) (Phenomenex) and using 0.2% formic acid in water HPLC grade and 0.2% formic acid in acetonitrile HPLC grade as eluents. The purity of all tested compounds is 95% or higher.

**General procedure A:** aminolysis reaction for the synthesis of **7–11**.

A solution of **6** (900 mg, 0.40 mmol), TEA (1.1 mL, 0.80 mmol), the corresponding amine (0.50 mmol), and toluene (31 mL) was stirred at reflux overnight. Then, the mixture was diluted with EtOAc and washed with a saturated NH_4_Cl solution. The organic layer was dried over sodium sulfate, filtered, evaporated under reduced pressure, and purified through flash chromatography.

**General procedure B:** amide coupling reaction for the synthesis of compounds **12–14**, **16–19**, **21**, **23**, **29**, **34**, **39**-**41**, **51–53**, **55**, **56**, and **71**.

A solution of the suitable carboxylic acid (0.30 mmol), the proper amine (0.36 mmol), EDCI (0.36 mmol), HOBt (0.36 mmol), and TEA (0.66 mmol) in dry DCM (2.56 mL) was stirred at room temperature, under a nitrogen atmosphere for 18 h. The mixture was diluted with water and extracted with DCM (2x). The organic layer was dried over sodium sulfate, filtered, evaporated, and purified through flash chromatography.

**General procedure C:** ester hydrolysis for the synthesis of **15**, **20**, **33**, **36**, and **37**.

NaOH (0.36 mmol) and water (1 mL) were added to a solution of the methyl/ethyl ester compound (0.18 mmol) in THF (1 mL). The reaction mixture was stirred at room temperature overnight. Subsequently, a 1 M HCl solution was added to adjust the pH to 4. The product was extracted with EtOAc, and the organic layer was dried over sodium sulfate, filtered, evaporated in vacuo, and purified through flash chromatography.

**General procedure D:** amide coupling reaction for the preparation of **32**, **68**, and **69**.

A solution of the appropriate acid (1.47 mmol) and amine (1.47 mmol), EDCI (2.95 mmol), DIPEA (4.43 mmol), and DMAP (0.15 mmol) in dry DCM (6 mL) was stirred under a nitrogen atmosphere at room temperature for 48 h. The mixture was diluted with water and extracted with DCM (2x). The organic layer was dried over sodium sulfate, filtered, evaporated, and purified through flash chromatography.

**General procedure E:** Boc cleavage for the synthesis of compounds **50** and **54**.

To a solution of the protected amine (0.25 mmol) in DCM (1.67 mL), CF_3_COOH (6 mmol) was added dropwise at 0 °C. The reaction was stirred at 0 °C for 30 min and then at room temperature for 2 additional hours. The reaction was purified through flash chromatography without any previous workup.

**General procedure F:** one-pot synthesis of urea compounds **60–67**.

A solution of **59** (0.17 mmol) in dry THF, the appropriate amine intermediate (0.088 mmol), and DMAP (0.17 mmol) was stirred for 7 h at room temperature. Then **1** (0.17 mmol) was added, and the mixture was refluxed overnight. After the solvent was evaporated, the crude material was purified through flash chromatography.

**2-(2-((2-(1** ***H*** **-Indol-3-yl)­ethyl)­amino)-2-oxoacetamido)­benzoic acid (7)**

Prepared according to General procedure A using **1**; purification by flash column chromatography (EtOAc/MeOH 9:1) afforded **7** as a brown solid (yield: 68%). ^1^H NMR (400 MHz, acetone-*d*_6_): δ 12.89 (br s, 1H), 10.01 (br s, 1H), 8.71 (d, *J* = 8.0 Hz, 1H), 8.40 (br s, 1H), 8.19 (d, *J* = 7.6 Hz, 1H), 7.64 (d, *J* = 7.8 Hz, 1H), 7.60 (s, 1H), 7.38 (d, *J* = 8.1 Hz, 1H), 7.21 (s, 2H), 7.09 (t, *J* = 7.3 Hz, 1H), 7.02 (t, *J* = 7.4 Hz, 1H), 3.67 (q, *J* = 6.8 Hz, 2H), 3.07 (t, *J* = 7.2 Hz, 2H). LRMS (ESI) *m*/*z* (M-H)^−^ calcd for C_19_H_16_N_3_O_4_ 350 found 350.

**2-(2-((3-(1** ***H*** **-Indol-3-yl)­propyl)­amino)-2-oxoacetamido)­benzoic acid (8)**

Prepared according to General procedure A using **2**; purification by flash column chromatographic (PE/EtOAc 3:7) afforded **8** as a brown solid (yield: 51%). ^1^H NMR (400 MHz, methanol-*d*_4_): δ 8.71 (d, *J* = 8.3 Hz, 1H), 8.14 (d, *J* = 7.4 Hz, 1H), 7.61–7.51 (m, 2H), 7.32 (d, *J* = 8.3 Hz, 1H), 7.22 (t, *J* = 7.4 Hz, 1H), 7.09–7.06 (m, 2H), 7.00 (t, *J* = 7.4 Hz, 1H), 3.41 (t, *J* = 7.0 Hz, 2H), 2.84 (t, *J* = 7.0 Hz, 2H), 2.03 (quint, *J* = 7.0 Hz, 2H). HRMS (ESI) *m*/*z* calcd for C_20_H_18_N_3_O_4_ (M-H)^−^ 364.1303, found 364.1303.

**2-(2-((3-(1** ***H*** **-Indol-3-yl)-1-methoxy-1-oxopropan-2-yl)­amino)-2-oxoacetamido)­benzoic acid (9)**

Preparation according to General procedure A using **3**; purification by flash column chromatography using (PE/EtOAc 3:7 as eluent) afforded **9** as a yellow solid (yield: 48%). ^1^H NMR (400 MHz, acetone-*d*_6_): δ 12.64 (s, 1H), 10.13 (s, 1H), 8.72 (dd, *Js* = 8.5, 1.1 Hz, 1H), 8.16 (dd, *Js* = 8.0, 1.7 Hz, 1H), 7.67 (ddd, *Js* = 8.6, 7.3, 1.7 Hz, 1H), 7.59 (dq, *Js* = 7.8, 0.9 Hz, 1H), 7.38 (dt, *Js* = 8.0, 0.9 Hz, 1H), 7.31–7.24 (m, 2H), 7.10 (ddd, *Js* = 8.1, 7.0, 1.3 Hz, 1H), 7.03 (ddd, *Js* = 8.1, 7.0, 1.1 Hz, 1H), 4.86 (dt, *Js* = 8.3, 6.2 Hz, 1H), 3.71 (s, 3H). HRMS (ESI) *m*/*z* calcd for C_21_H_18_N_3_O_6_ (M-H)^−^ 408.1201, found 408.1200.

**2-(2-(((1** ***H*** **-Indol-3-yl)­methyl)­amino)-2-oxoacetamido)­benzoic acid (10)**

Prepared according to General procedure A using **4**. The crude product was used in the next step without further purification as a brown solid (yield: 60%). ^1^H NMR (400 MHz, acetone-*d*_6_): δ 10.14 (br s, 1H), 8.60 (d, *J* = 8.2 Hz, 1H), 8.34 (d, *J* = 7.6 Hz, 1H), 7.44–7.29 (m, 3H), 7.09 (t, *J* = 7.6 Hz, 1H), 7.11–6.92 (m, 3H), 4.21 (s, 2H). HRMS (ESI) *m*/*z* (M-H)^−^ calcd for C_18_H_14_N_3_O_4_ 236.0990, found 236.0564.

**2-(2-((2-(Benzofuran-3-yl)­ethyl)­amino)-2-oxoacetamido)­benzoic acid (11)**

Prepared according to General procedure A using **5**; purification by flash chromatographic column (EtOAc) afforded **11** as a brown solid (yield: 40%). ^1^H NMR (400 MHz, methanol-*d*_4_): δ 8.60 (d, *J* = 8.2 Hz, 1H), 8.08 (dd, *J* = 7.8, 1.4 Hz, 1H), 7.67 (d, *J* = 7.4 Hz, 1H), 7.62 (s, 1H), 7.45 (td, *Js* = 8.5, 2.8 Hz, 2H), 7.30–7.20 (m, 4H), 7.14 (t, *J* = 7.9 Hz, 1H), 3.64 (t, *J* = 7.3 Hz, 2H), 2.99 (t, *J* = 7.3 Hz, 2H). LRMS (ESI) *m*/*z* calcd for C_19_H_15_N_2_O_5_ (M-H)^−^ 351, found 351.

***N*** ^**1**^ **-(2-(1** ***H*** **-Indol-3-yl)­ethyl)-** ***N*** ^**2**^ **-(2-((3-(1** *H* **-indol-3yl)­propyl)­carbamoyl)­phenyl)­oxalamide (12)**

Prepared according to General procedure B using **2** and **7**; purification by flash column chromatography (EtOAc) afforded **12** as a white solid (50.8 mg, 0.10 mmol, yield: 35%). Mp 184–185 °C; ^1^H NMR (400 MHz, acetone-*d*_6_): δ 12.81 (br s, 1H), 10.08 (br s, 1H), 10.00 (br s, 1H), 8.66 (d, *J* = 8.3 Hz, 1H), 8.40 (br s,1H), 8.05 (br s, 1H), 7.75 (d, *J* = 7.8 Hz, 2H), 7.69 (d, *J* = 7.8 Hz, 1H), 7.60 (d, *J* = 8.0 Hz, 1H), 7.52 (t, *J* = 7.8 Hz, 1H), 7.42–7.39 (m, 2H), 7.19–7.00 (m, 6H), 3.70 (q, *J* = 7.5 Hz, 2H), 3.55 (q, *J* = 6.6 Hz, 2H), 3.10 (t, *J* = 7.5 Hz, 2H), 2.90 (t, *J* = 6.6 Hz, 2H), 2.07 (quint, *J* = 7.5 Hz, 2H). ^13^C NMR (101 MHz, acetone-*d*_6_): δ 168.0, 159.8, 158.7, 138.0 (2C), 136.9, 136.8, 131.7, 127.7, 127.6, 123.6, 122.6, 122.4, 122.1, 122.0, 121.3, 121.1, 120.4, 118.6, 118.4 (2C), 114.7, 112.0, 111.3, 111.2, 40.2, 39.5, 29.8, 25.0, 22.4. IR (neat): ṽ = 3395, 2920, 1672, 1637, 1543, 1509, 1450, 1298, 1225, 744, 580, 500 cm^–1^. HRMS (ESI) *m*/*z* (M+H)^+^ calcd for C_30_H_30_N_5_O_3_ 508.2343, found 508.2346.

**Methyl (2-(2-((2-(1** ***H*** **-indol-3-yl)­ethyl)­amino)-2-oxoacetamido)­benzoyl)­tryptophanate (13)**

Prepared according to General procedure B using **3** and **7**; purification by flash column chromatography (PE/EtOAc 4:6) afforded **13** as a yellow solid (178.7 mg, 0.32 mmol, yield: 57%). Mp 152–153 °C; ^1^H NMR (400 MHz, acetone-*d*_6_): δ 12.51 (br s, 1H), 10.05 (d, *J* = 20.8 Hz, 2H), 8.62 (d, *J* = 8.3 Hz, 1H), 8.34 (br s, 1H), 8.01 (d, *J* = 7.3 Hz, 1H), 7.74–7.61 (m, 3H), 7.51 (t, *J* = 7.9 Hz, 1H), 7.38 (t, *J* = 7.5 Hz, 2H), 7.31 (s, 1H), 7.23 (s, 1H), 7.16–7.07 (m, 3H), 7.06–6.99 (m, 2H), 5.03 (td, *Js* = 7.8, 5.4 Hz, 1H), 3.71 (s, 3H), 3.67 (q, *J* = 7.0 Hz, 2H), 3.53–3.36 (m, 2H), 3.08 (t, *J* = 7.4 Hz, 2H). ^13^C NMR (101 MHz, acetone-*d*_6_): δ 172.8, 168.9, 160.6, 159.6, 138.9, 137.8, 137.6, 133.1, 128.8, 128.6, 128.5, 124.4, 124.4, 123.5, 122.4, 122.3, 122.2, 121.3, 119.7, 119.5, 119.3, 119.1, 112.9, 112.3, 112.2, 110.9, 54.6, 52.5, 41.1, 27.9, 25.9. IR (neat): ṽ = 3416, 3359, 2921, 1678, 1507, 1441, 1365, 1215, 1184, 733, 496, 425 cm^–1^. HRMS (ESI) *m*/*z* (M+H)^+^ calcd for C_31_H_30_N_5_O_5_ 552.2241, found 552.2242.

***N*** ^**1**^ **-(2-(1** ***H*** **-Indol-3-yl)­ethyl)-** ***N*** ^**2**^ **-(2-((2-(benzofuran-3-yl)­ethyl)­carbamoyl)­phenyl)­oxalamide (14)**

Prepared according to General procedure B using **4** and **7**; purification by column chromatography using PE/EtOAc 7:3 as eluent afforded **14** as a yellow solid (76.9 mg, 0.15 mmol, 48%). Mp 180–181 °C; ^1^H NMR (400 MHz, DMSO-*d*_6_): δ 10.80 (br s, 1H), 9.09 (br s, 1H), 8.92 (br s, 1H), 8.54 (d, *J* = 8.1 Hz, 1H), 7.87 (s, 1H), 7.75–7.69 (m, 2H), 7.62–7.53 (m, 3H), 7.36–7.23 (m, 5H), 7.08 (t, *J* = 7.1 Hz, 1H), 7.00 (t, *J* = 7.6 Hz, 1H), 3.63 (t, *J* = 6.0 Hz, 2H), 3.51 (t, *J* = 7.2 Hz, 2H), 3.00–2.93 (m, 4H). ^13^C NMR (101 MHz, DMSO-*d*_6_): δ 168.1, 159.8, 158.7, 142.2, 138.1, 136.9, 131.7, 128.2, 127.6, 124.2, 123.5, 122.5, 122.3, 122.2, 121.3, 120.5, 119.7, 118.6 (2C), 118.4 (2C), 117.8, 112.1, 111.3, 111.1, 40.2, 39.3, 25.0, 23.2. IR (neat): ṽ = 3321, 1680, 1595, 1519, 1448, 1088, 740, 496, 420 cm^–1^. HRMS (ESI) *m*/*z* (M+H)^+^ calcd for C_29_H_27_N_4_O_4_ 495.2027, found 495.2027.

**(2-(2-((2-(1** ***H*** **-Indol-3-yl)­ethyl)­amino)-2-oxoacetamido)­benzoyl)­tryptophan (15)**

Prepared according to General procedure C, starting from **13**; purification by column chromatography using EtOAc/MeOH 8:2 as eluent afforded **15** as a yellow solid (38.4 mg, 0.071 mmol, yield: 40%). Mp 182–183 °C; ^1^H NMR (400 MHz, methanol-*d*_4_): δ 8.35 (d, *J* = 8.6 Hz, 1H), 7.50–7.46 (m, 2H), 7.35–7.31 (m, 3H), 7.22 (d, *J* = 8.0 Hz, 1H), 7.16 (d, *J* = 8.0 Hz, 1H), 7.03 (s, 1H), 6.99–6.88 (m, 5H), 6.79 (t, *J* = 7.3 Hz, 1H), 4.77–4.74 (m, 1H), 3.51 (t, *J* = 7.4 Hz, 1H), 3.43 (dd, *Js* = 14.7, 5.0 Hz, 1H), 3.26–3.24 (m, 1H), 2.93 (t, *J* = 7.4 Hz, 2H). 175.9, 168.4, 160.2, 158.0, 136.8, 136.7, 136.5, 131.5, 127.8, 127.6, 127.2, 123.7, 123.0, 122.5, 122.1, 121.0, 120.9, 120.4, 118.3 (2C), 118.1, 117.9, 111.4, 110.8 (2C), 110.2, 55.0, 40.3, 24.6, 19.4. IR (neat): ṽ = 3384, 2921, 1670, 1584, 1508, 1447, 1224, 1092, 738, 422 cm^–1^. HRMS (ESI) *m*/*z* calculated for C_30_H_28_N_5_O_5_ [M + H]^+^ 538.2085, found 538.2087.

***N*** ^**1**^ **-(2-((2-(1** ***H*** **-Indol-3-yl)­ethyl)­carbamoyl)­phenyl)-** *N* ^**2**^ **-(3-(1** *H* **-indol-3-yl)­propyl)­oxalamide (16)**

Prepared according to General procedure B using **1** and **8**; purification by chromatographic column using PE/EtOAc 3:7 as eluent afforded **16** as a white solid (51.7 mg, 0.102 mmol, yield: 39%). Mp 107–108 °C; ^1^H NMR (400 MHz, chloroform-*d*) δ 12.70 (s, 1H), 8.59 (d, *J* = 8.4 Hz, 1H), 8.08 (s, 1H), 7.99 (s, 1H), 7.61 (dd, *Js* = 12.9, 7.9 Hz, 1H), 7.54–7.50 (m, 1H), 7.49–7.44 (m, 2H), 7.40–7.34 (m, 1H), 7.29 (d, *J* = 1.5 Hz, 1H), 7.24–7.16 (m, 2H), 7.16–7.09 (m, 3H), 7.09–7.03 (m, 2H), 6.25 (s, 2H), 3.84 (q, *J* = 6.3 Hz, 2H), 3.47 (q, *J* = 6.7 Hz, 2H), 3.11 (t, *J* = 6.6 Hz, 2H), 2.85 (t, *J* = 7.3 Hz, 2H), 2.03 (p, *J* = 7.3 Hz, 2H). ^13^C NMR (101 MHz, acetone-*d*_6_) δ 168.9, 160.8, 159.6, 138.9, 137.8, 137.7, 132.6, 128.6, 128.6, 128.5, 124.4, 123.5, 123.3, 122.9, 122.1, 122.0, 121.3, 119.4, 119.3, 119.23 (2C), 115.4, 113.3, 112.2, 112.1, 41.4, 40.3, 30.6, 25.9, 23.2. IR (neat): ṽ = 3308, 2961, 1674, 1513, 1442, 1258, 1086, 1017, 794 cm^–1^. HRMS (ESI) *m*/*z* calcd for C_30_H_30_N_5_O_3_ [M + H]^+^ 508.2343, found 508.2345.

**Methyl (2-((2-((2-(1** ***H*** **-Indol-3-yl)­ethyl)­carbamoyl)­phenyl)­amino)-2-oxoacetyl)­tryptophanate (17)**

Prepared according to General procedure B using **1** and **9**; purification by column chromatography using PE/EtOAc 3:7 as eluent afforded **17** as an off-white solid (27.6 mg, 0.050 mmol, yield: 37%). Mp 104–105 °C; ^1^H NMR (400 MHz, acetone-*d*_6_) δ 12.81 (s, 1H), 10.06 (s, 1H), 10.04 (s, 1H), 8.61 (dt, *J*s = 8.4, 1.2 Hz, 1H), 8.26 (d, *J* = 8.2 Hz, 1H), 8.09 (s, 1H), 7.74 (dd, *J*s = 7.9, 1.5 Hz, 1H), 7.66–7.58 (m, 2H), 7.56–7.47 (m, 1H), 7.40–7.35 (m, 2H), 7.26 (dd, *J*s = 17.0, 2.3 Hz, 2H), 7.19–7.13 (m, 1H), 7.13–7.06 (m, 2H), 7.06–6.97 (m, 2H), 4.88 (dt, *J*s = 9.0, 6.1 Hz, 1H), 3.77–3.72 (m, 2H), 3.71 (s, 3H), 3.45 (d, *J* = 6.1 Hz, 2H), 3.10 (t, *J* = 7.3 Hz, 2H). ^13^C NMR (101 MHz, acetone-*d*_6_) δ 172.1, 168.9, 160.4, 158.9, 138.8, 137.7, 137.6, 132.7, 128.6 (2C), 128.5, 124.6, 124.6, 123.5, 123.2, 122.3, 122.1, 121.4, 119.8, 119.5, 119.3, 119.1, 113.3, 112.3, 112.2, 110.2, 54.4, 52.7, 41.3, 27.9, 25.9. IR (neat): ṽ = 3369, 2922, 2851, 1737, 1673, 1504, 1448, 1220, 1094, 739, 481, 424 cm^–1^. HRMS (ESI) *m*/*z* calcd for C_31_H_28_N_5_O_5_ [M-H]^−^ 550.2096, found 550.2095.

***N*** ^**1**^ **-(2-((2-(1** ***H*** **-Indol-3-yl)­ethyl)­carbamoyl)­phenyl)-** *N* ^**2**^ **-((1** *H* **-indol-3-yl)­methyl)­oxalamide (18)**

Prepared according to General procedure B, using **1** and **11**; purification by chromatographic column using PE/EtOAc 7.5:2.5 as eluent afforded **18** as a yellow solid (37.8 mg, 0.08 mmol, 19%). Mp 98–99 °C; ^1^H NMR (400 MHz, acetone-*d*_6_) δ 10.10–9.90 (m, 3H), 9.74 (s, 1H), 8.23 (s, 1H), 8.14 (d, *J* = 5.1 Hz, 1H), 7.69–7.57 (m, 2H), 7.44 (d, *J* = 5.1 Hz, 1H), 7.42–7.33 (m, 2H), 7.21 (dd, *J*s = 5.3, 2.2 Hz, 2H), 7.09 (dtd, *J*s = 7.9, 6.7, 1.3 Hz, 2H), 7.01 (ddt, *J*s = 8.0, 7.0, 1.1 Hz, 2H), 3.69 (td, *J*s = 7.2, 5.7 Hz, 2H), 3.58 (td, *J*s = 7.4, 5.7 Hz, 2H), 3.08 (t, *J* = 7.2 Hz, 2H), 3.03 (t, *J* = 7.4 Hz, 2H). ^13^C NMR (101 MHz, acetone-*d*_6_) δ 168.9, 160.4, 159.6, 138.8, 137.6, 137.5, 132.6, 129.5, 128.6, 128.3, 127.7, 125.0, 124.4, 123.3, 122.4, 122.1, 121.3, 119.9, 119.6, 119.4, 119.3, 113.2, 112.8, 112.2, 112.1, 41.2, 35.5, 25.9. IR (neat): ṽ = 3369, 2921, 2852, 1671, 1506, 1448, 1260, 1012, 799, 739, 534, 422 cm^–1^. HRMS (ESI) *m*/*z* calcd for C_28_H_26_N_5_O_3_ [M + H]^+^ 480.2030, found 480.2029.

***N*** ^**1**^ **-(2-((2-(1** ***H*** **-Indol-3-yl)­ethyl)­carbamoyl)­phenyl)-** *N* ^**2**^ **-(2-(benzofuran-3-yl)­ethyl)­oxalamide (19)**

Prepared according to General procedure B using **1** and **10**; purification by column chromatography using PE/EtOAc 6:4 as eluent afforded **19** as a brown solid (48.2 mg, 0.097 mmol, yield: 43%). Mp 189–190 °C; ^1^H NMR (400 MHz, acetone-*d*_6_) δ 12.79 (s, 1H), 10.01 (s, 1H), 8.64 (d, *J* = 8.4 Hz, 1H), 8.48 (s, 1H), 8.08 (s, 1H), 7.79–7.71 (m, 3H), 7.65 (d, *J* = 7.8 Hz, 1H), 7.55–7.45 (m, 2H), 7.38 (d, *J* = 8.1 Hz, 1H), 7.34–7.23 (m, 3H), 7.12 (dt, *Js* = 24.3, 7.7 Hz, 2H), 7.01 (t, *J* = 7.4 Hz, 1H), 3.74 (dq, *Js* = 13.9, 6.9 Hz, 4H), 3.12 (t, *J* = 7.3 Hz, 2H), 3.07 (t, *J* = 7.3 Hz, 2H). ^13^C NMR (101 MHz, acetone-*d*_6_) δ 168.9, 160.92 159.5, 156.2, 143.1, 138.9, 137.7, 132.6, 129.0, 128.6, 128.6, 125.2, 124.4, 123.5, 123.3, 123.3, 122.1, 121.4, 120.7, 119.4, 119.3 118.5, 113.3, 112.2, 112.1, 41.4, 40.0, 25.9, 24.1. IR (neat): ṽ = 3299, 2921, 2850, 1670, 1632, 1504, 1446, 1181, 1092, 737, 628, 546, 425 cm^–1^. HRMS (ESI) *m*/*z* (M+H)^+^ calcd for C_29_H_27_N_4_O_4_ 495.2027, found 495.2023.

**(2-((2-((2-(1** ***H*** **-Indol-3-yl)­ethyl)­carbamoyl)­phenyl)­amino)-2-oxoacetyl)­tryptophan (20)**

Prepared according to General procedure C starting from **17**; purification by column chromatography using EtOAc/MeOH 8:2 as eluent afforded **20** as a white solid (9.0 mg, 0.016 mmol, yield: 13%). Mp 184–185 °C; ^1^H NMR (400 MHz, methanol-*d*_4_): δ 8.47 (d, *J* = 8.2 Hz, 1H), 7.65–7.55 (m, 3H), 7.47 (t, *J* = 7.3 Hz, 1H), 7.34–7.29 (m, 2H), 7.20–6.96 (m, 7H), 4.71 (t, *J* = 5.6 Hz, 1H), 3.69 (t, *J* = 7.3 Hz, 2H), 3.42–3.39 (m, 2H), 3.09 (t, *J* = 7.2 Hz, 2H). ^13^C NMR (101 MHz, methanol-*d*_4_): δ 168.9, 162.0, 159.5, 157.9, 146.2, 136.8, 136.5, 134.3, 131.4, 127.7, 127.4, 126.0, 123.7, 123.3, 122.8, 122.1, 121.3, 120.8, 120.4, 118.3, 118.2, 118.1, 117.9, 111.9, 110.8, 109.6, 40.6, 29.3, 27.1, 24.6. IR (neat): ṽ = 3350, 2922, 2852, 1671, 1508, 1449, 1259, 1012, 795, 739, 492 cm^–1^. HRMS (ESI) *m*/*z* (M+H)^+^ calcd for C_30_H_28_N_5_O_5_ 538.2085, found 538.2090.

***N*** ^***1***^ **-(2-(Benzofuran-3-yl)­ethyl)-** ***N*** ^**2**^ **-(2-((2-(benzofuran-3-yl)­ethyl)­carbamoyl)­phenyl)­oxalamide (21)**

Prepared according to General procedure B using **4** and **10**; purification by column chromatography using PE/EtOAc 8:2 as eluent afforded **21** as a yellow solid (90.0 mg, 0.182 mmol, yield: 60%). Mp 154–156 °C; ^1^H NMR (400 MHz, acetone-*d*_6_): δ 12.72 (s, 1H), 8.64 (dd, *Js* = 8.4, 1.2 Hz, 1H), 8.49 (s, 1H), 8.19 (s, 1H), 7.81–7.66 (m, 5H), 7.56–7.43 (m, 3H), 7.37–7.21 (m, 4H), 7.16 (td, *Js* = 7.6, 1.2 Hz, 1H), 3.79 (td, *Js* = 7.0, 5.8 Hz, 2H), 3.76–3.69 (m, 2H), 3.18–2.99 (m, 4H). ^13^C NMR (101 MHz, chloroform-*d*) δ 168.3, 160.2, 158.4, 155.6 (2C), 142.1, 142.0, 138.1, 132.7, 127.8 (2C), 126.8, 124.7, 124.6, 124.1, 122.8, 122.7, 121.7, 121.3, 119.6 (2C), 117.4, 116.9, 111.8 (2C), 39.5, 39.4, 23.9. IR (neat): ṽ = 3060, 2962, 1676, 1620, 1509, 1259, 1080, 1019, 794, 738 cm^–1^. HRMS (ESI) *m*/*z* (M+H)^+^ calcd for C_29_H_26_N_3_O_5_ 496.1867, found 496.1864.

***N*** **-(2-(1** ***H*** **-Indol-3-yl)­ethyl)-2-aminobenzamide (23)**

Prepared according to General procedure B using **1** and **22**; purification by column chromatography using PE/EtOAc 6:4 as eluent afforded **23** as a brown solid (yield: 88%). ^1^H NMR (400 MHz, acetone-*d*_6_): δ 9.98 (br s, 1H), 7.68 (d, *J* = 7.8 Hz, 1H), 7.57 (br s, 1H), 7.47 (d, *J* = 7.9 Hz, 1H), 7.40 (d, *J* = 7.9 Hz, 1H), 7.22 (s, 1H), 7.16–7.09 (m, 2H), 7.04 (t, *J* = 7.9 Hz, 1H), 6.76 (d, *J* = 7.8 Hz, 1H), 6.52 (t, *J* = 7.8 Hz, 1H), 6.24 (br s, 2H), 3.70 (q, *J* = 7.2 Hz, 2H), 3.09 (t, *J* = 7.2 Hz, 2H). LRMS (ESI) *m*/*z* (M+H)^+^ calcd for C_17_H_18_N_3_O 280, found 280.

***N*** **-(2-(1** ***H***-**Indol-3-yl)­ethyl)-2-(2-bromoacetamido)­benzamide (25)**

To a solution of **23** (400 mg, 1.4 mmol, 1 equiv) in dry DCM (8 mL) and TEA (439 μL, 3.15 mmol, 2.2 equiv), **24** (310 μL, 3.72 mmol, 2.6 equiv) was added dropwise at 0 °C under a nitrogen atmosphere. After 4 h, the reaction was diluted with water and extracted with DCM (2x). The organic layer was dried over sodium sulfate, filtered, and concentrated under *vacuum*. Purification by column chromatography using PE/EtOAc 6:4 as eluent afforded **25** as a yellow solid (yield: 67%). ^1^H NMR (400 MHz, acetone-*d*_6_) δ 12.06 (s, 1H), 10.01 (s, 1H), 8.56 (dd, *Js* = 8.4, 0.9 Hz, 1H), 8.15 (s, 1H), 7.72 (dd, *J* = 7.9, 1.3 Hz, 1H), 7.65 (d, *J* = 7.8 Hz, 1H), 7.51–7.45 (m, 1H), 7.39 (d, *J* = 8.1 Hz, 1H), 7.22 (d, *J* = 2.2 Hz, 1H), 7.15–7.07 (m, 2H), 7.01 (td, *Js* = 7.5, 7.1, 1.0 Hz, 1H), 4.12 (s, 2H), 3.74 (q, 2H), 3.11 (t, *J* = 7.2 Hz, 2H). LRMS (ESI) *m*/*z* (M+Na)^+^ calcd for C_19_H_18_BrN_3_NaO_2_ 422 found 422.

***N*** **-(2-(1** ***H*** **-Indol-3-yl)­ethyl)-2-(2-((2-(1** *H* **-indol-3-yl)­ethyl)­amino)­acetamido)­benzamide (26)**

To a solution of **25** (150 mg, 0.4 mmol, 1 equiv) in AcCN (2.8 mL), **1** (120 mg, 0.7 mmol) and K_2_CO_3_ (99.8 mg, 0.7 mmol) were added. The mixture was heated at 90 °C for 18 h. The solvent was evaporated, and the resulting residue was diluted with water and extracted with EtOAc (2x). The organic layer was dried over sodium sulfate, filtered, and concentrated under reduced pressure. Purification by column chromatography (PE/EtOAc 5:5 as eluent) afforded **26** as a pink solid (111.6 mg, 0.233 mmol, yield: 62%). Mp: 61–62 °C; ^1^H NMR (400 MHz, acetone-*d*_6_): δ 12.05 (br s, 1H), 9.96 (br s, 1H), 8.72 (d, *J* = 8.4 Hz, 1H), 7.96 (br s, 1H), 7.71–7.64 (m, 3H), 7.44 (t, *J* = 8.4 Hz, 1H), 7.39–7.36 (m, 2H), 7.25 (s, 1H), 7.19 (s, 1H), 7.10–7.04 (m, 3H), 7.00–6.96 (m, 2H), 3.79 (q, *J* = 7.1 Hz, 2H), 3.39 (s, 2H), 3.18–3.13 (m, 4H), 3.00 (t, *J* = 7.1 Hz, 2H). ^13^C NMR (101 MHz, acetone-*d*_6_): δ 171.2, 168.4, 138.9, 136.8, 131.3, 129.6, 127.8, 127.7, 127.5, 122.8, 122.6, 122.5, 122.3, 121.2, 121.1, 120.5, 118.7, 118.6, 118.5 (2C), 113.1, 112.5, 111.3, 111.2, 53.5, 50.7, 40.5, 25.6, 25.4. IR (neat): ṽ = 3291, 2920, 1636, 1580, 1509, 1444, 1284, 1092, 734, 485, 423 cm^–1^. HRMS (ESI) *m*/*z* (M+H)^+^ calcd for C_29_H_30_N_5_O_2_ 480.2394, found 480.2392.

**(2-((2-(1** ***H*** **-Indol-3-yl)­ethyl)­carbamoyl)­phenyl)­glycine (28)**

To a suspension of **23** (100 mg, 0.36 mmol) and **27** (0.38 mL, 0.54 mmol) in EtOH (5 mL), TEA (0.15 mL, 1.07 mmol) and DMAP (13.12 mg, 0.11 mmol) were added. The mixture was refluxed overnight. After 8 days, the volatile was evaporated under *vacuum*; the crude material was then diluted with DCM and washed with a 3N solution of HCl. The organic phase was dried over sodium sulfate, filtered, and evaporated under reduced pressure. Purification by column chromatography (PE/EtOAc 5:5 as eluent) afforded **28** as a dark yellow solid (yield: 30%). ^1^H NMR (400 MHz, methanol-*d*_4_): δ 7.65 (d, *J* = 7.8 Hz, 1H), 7.45–7.37 (m, 2H), 7.25 (t, *J* = 7.8 Hz, 1H), 7.20–7.09 (m, 2H), 7.00 (t, *J* = 7.8 Hz, 1H), 6.62–6.59 (m, 2H), 3.92 (s, 2H), 3.38 (t, *J* = 7.3 Hz, 2H), 3.11 (t, *J* = 7.3 Hz, 2H). LRMS (ESI) *m*/*z* (M+Na)^+^ calcd for C_19_H_19_N_3_O_3_Na 360, found 360.

***N*** **-(2-(1** ***H*** **-Indol-3-yl)­ethyl)-2-((2-((2-(1** *H* **-indol-3-yl)­ethyl)­amino)-2-oxoethyl)­amino)­benzamide (29)**

Prepared according to General procedure B using **1** and **28**; purification by column chromatography using PE/EtOAc 4:6 as eluent, yielded **29** as a brown solid (30.9 mg, yield: 62%). Mp: 81–82 °C; ^1^H NMR (400 MHz, acetone-*d*_6_): δ 7.96 (s, 1H), 7.63 (d, *J* = 7.9 Hz, 1H), 7.51 (d, *J* = 7.9 Hz, 1H), 7.39–7.30 (m, 3H), 7.20 (t, *J* = 7.0 Hz, 1H), 7.12–7.05 (m, 3H), 7.02–6.96 (m, 2H), 6.87 (s, 1H), 6.66 (t, *J* = 7.3 Hz, 1H), 6.42 (d, *J* = 7.9 Hz, 1H), 3.71 (s, 2H), 3.67 (t, *J* = 7.3 Hz, 2H), 3.50 (t, *J* = 7.1 Hz, 2H), 3.07 (t, *J* = 7.3 Hz, 2H), 2.89 (t, *J* = 7.1 Hz, 2H). ^13^C NMR (101 MHz, acetone-*d*_6_): δ 169.8, 162.5, 136.9, 136.8, 132.9, 132.2, 128.5, 127.9, 127.7, 127.6, 122.6, 122.5, 121.2, 118.7, 118.6, 118.5, 116.7, 115.4, 112.8, 112.6, 112.5, 112.3, 111.6, 111.3, 58.3, 52.6, 47.3, 40.2, 25.4. IR (neat): ṽ = 3273, 2922, 2852, 1629, 1510, 1454, 1259, 1011, 798, 738, 474 cm^–1^. HRMS (ESI) *m*/*z* (M+H)^+^ calcd for C_29_H_30_N_5_O_2_ 480.2394, found 480.2392.

**Ethyl 2-(4-(1** ***H*** **-Indol-3-yl)­butanamido)­benzoate (32)**

Prepared according to General procedure D starting from **30** and **31**; the crude material was purified by column chromatography using PE/EtOAc 8:2 as eluent, affording **32** as a yellow solid (yield: 27%). ^1^H NMR (400 MHz, acetone-*d*_6_) δ 11.02 (s, 1H), 9.96 (s, 1H), 8.80–8.71 (m, 1H), 8.05 (dd, *Js* = 8.0, 1.6 Hz, 1H), 7.65–7.53 (m, 2H), 7.37 (d, *J* = 8.1 Hz, 1H), 7.18 (s, 1H), 7.15–7.06 (m, 2H), 7.03–6.97 (m, 1H), 4.38 (q, *J* = 7.1 Hz, 2H), 2.88 (t, 2H), 2.53 (t, *J* = 7.4 Hz, 2H), 2.14 (p, *J* = 7.5 Hz, 2H), 1.38 (t, *J* = 7.1 Hz, 3H). HRMS (ESI) *m*/*z* (M+H)^+^ calcd for C_21_H_23_N_2_O_3_ 351.1703, found 351.1704.

**2-(4-(1** ***H*** **-Indol-3-yl)­butanamido)­benzoic acid (33)**

Prepared according to General procedure C starting from **32**; the crude product was isolated as a yellow solid (yield: 31%) and was pure enough to be used without further purification. ^1^H NMR (400 MHz, acetone *d*_6_): δ 9.85 (br s, 1H), 8.71 (d, *J* = 8.1 Hz, 1H), 8.28 (br s, 1H), 7.51 (d, *J* = 8.1 Hz, 1H), 7.33–7.31 (m, 2H), 7.06–6.95 (m, 5H), 2.65 (t, *J* = 7.4 Hz, 2H), 2.41–2.36 (m, 4H). HRMS (ESI) *m*/*z* (M+H)^+^ calcd for C_19_H_19_N_2_O_3_ 323.1390, found 323.1392.

**2-(4-(1** ***H*** **-Indol-3-yl)­butanamido)-** *N* **-(2-(1** *H* **-indol-3-yl)­ethyl)­benzamide (34)**

Prepared according to General procedure B starting from **33** and **1**; purification by column chromatography using PE/EtOAc 7:3 as eluent afforded **34** as a yellow solid (12.2 mg, 0.026 mmol, yield: 24%). Mp: 188–189 °C; ^1^H NMR (400 MHz, methanol-*d*_4_): δ 8.29 (d, *J* = 8.2 Hz, 1H), 7.61–7.51 (m, 3H), 7.45 (t, *J* = 7.8 Hz, 1H), 7.34–7.31 (m, 2H), 7.14–7.05 (m, 5H), 7.00–6.95 (m, 2H), 3.68 (t, *J* = 7.2 Hz, 2H), 3.07 (t, *J* = 7.2 Hz, 2H), 2.86 (t, *J* = 7.4 Hz, 2H), 2.44 (t, *J* = 7.4 Hz, 2H), 2.12 (q, *J* = 7.4 Hz, 2H). ^13^C NMR (101 MHz, methanol-*d*_4_): δ 172.9, 169.4, 137.9, 136.8, 134.1, 131.3, 127.4, 126.1, 125.9, 123.6, 122.8, 122.1, 121.6, 121.3, 120.9, 120.0, 118.4, 118.1, 117.1, 114.0, 113.8, 111.9, 110.9, 110.5, 40.5, 37.1, 29.3, 26.0, 24.2. IR (neat): ṽ = 3305, 2919, 1512, 1443, 1260, 1091, 1010, 799, 737, 422 cm^–1^. HRMS (ESI) *m*/*z* (M+H)^+^ calcd for C_29_H_29_N_4_O_2_ 465.2285, found 465.2287.

**Methyl 2-(3-((2-(1** ***H*** **-indol-3-yl)­ethyl)­amino)-3-oxopropyl)­benzoate (68)**

Prepared according to General procedure D using **35** and **1**; purification by column chromatography using PE/EtOAc 5:5 as eluent afforded **68** as a pink oil (yield: 58%). ^1^H NMR (400 MHz, methanol-*d*_4_): δ 7.83 (d, *J* = 6.8 Hz, 1H), 7.54 (d, *J* = 7.9 Hz, 1H), 7.37–7.33 (m, 2H), 7.23–7.19 (m, 2H), 7.09 (t, *J* = 7.9 Hz, 1H), 7.01 (t, *J* = 7.9 Hz, 1H), 6.98 (s, 1H), 3.81 (s, 3H), 3.44 (t, *J* = 7.3 Hz, 2H), 3.20 (t, *J* = 7.5 Hz, 2H), 2.87 (t, *J* = 7.3 Hz, 2H), 2.45 (t, *J* = 7.5 Hz, 2H). LRMS (ESI) *m*/*z* (M+Na)^+^ calcd for C_21_H_22_N_2_NaO_3_ 373, found 373.

**Methyl 2-(3-((2-(benzofuran-3-yl)­ethyl)­amino)-3-oxopropyl)­benzoate (69)**

Prepared according to General procedure D using **35** and **4**; purification by column chromatography using PE/EtOAc 7:3 as eluent afforded **69** as a brown oil (yield: 76%). ^1^H NMR (400 MHz, chloroform-*d*) δ 7.90 (dd, *Js* = 7.8, 1.5 Hz, 1H), 7.55 (ddd, *Js* = 7.7, 1.5, 0.7 Hz, 1H), 7.46 (dt, *Js* = 8.2, 0.9 Hz, 1H), 7.44–7.38 (m, 2H), 7.33–7.27 (m, 3H), 7.26–7.20 (m, 1H), 5.97 (s, 1H), 3.85 (s, 3H), 3.58 (td, *Js* = 6.8, 5.9 Hz, 2H), 3.28–3.15 (m, 2H), 2.86 (td, *Js* = 6.8, 1.1 Hz, 2H), 2.53–2.42 (m, 2H). LRMS (ESI) *m*/*z* (M+H)^+^ calcd for C_21_H_22_NO_4_ 352.15, found 352.18.

**2-(3-((2-(1** ***H*** **-Indol-3-yl)­ethyl)­amino)-3-oxopropyl)­benzoic acid (36)**

Prepared according to General procedure C starting from **68**; the crude product was isolated as a brown solid (yield: 68%) and was pure enough to be used without further purification. ^1^H NMR (400 MHz, methanol-*d*_4_): δ 7.92 (d, *J* = 6.3 Hz, 1H), 7.58 (d, *J* = 7.1 Hz, 1H), 7.43 (t, *J* = 6.3 Hz, 1H), 7.39–7.30 (m, 3H), 7.11 (t, *J* = 7.1 Hz, 1H), 7.02–6.99 (m, 2H), 3.45 (t, *J* = 7.2 Hz, 2H), 3.25 (t, *J* = 7.8 Hz, 2H), 2.88 (t, *J* = 7.2 Hz, 2H), 2.52 (t, *J* = 7.8 Hz, 2H). HRMS (ESI) *m*/*z* (M+H)^+^ calcd for C_20_H_21_N_2_O_3_ 337.1547, found 337.1543.

**2-(3-((2-(Benzofuran-3-yl)­ethyl)­amino)-3-oxopropyl)­benzoic acid (37)**

Prepared according to General procedure C starting from **69**; the crude product was isolated as a yellow solid (yield: 90%) and was pure enough to be used without further purification. ^1^H NMR (400 MHz, chloroform-*d*): δ 7.98 (d, *J* = 9.4 Hz, 1H), 7.52 (d, *J* = 7.4 Hz, 1H), 7.49–7.42 (m, 2H), 7.35 (s, 1H), 7.33–7.26 (m, 3H), 7.23 (t, *J* = 7.4 Hz, 1H), 6.00 (br s, 1H), 3.56 (q, *J* = 6.6 Hz, 2H), 3.29 (t, *J* = 6.6 Hz, 2H), 2.84 (t, *J* = 7.0 Hz, 2H), 2.59 (t, *J* = 7.0 Hz, 2H). HRMS (ESI) *m*/*z* (M-H)^−^ calcd for C_20_H_18_NO_4_ 336.1241, found 336.1243.

***N*** **-(2-(1** ***H*** **-Indol-3-yl)­ethyl)-2-(3-((2-(1** ***H*** **-indol-3-yl)­ethyl)­amino)-3-oxopropyl)­benzamide (39)**

Prepared according to General procedure B using **36** and **1**; purification by column chromatography using PE/EtOAc 4:6 as eluent, yielded **39** as a light brown solid (91.0 mg, 0.190 mmol, yield: 71%). Mp: 98–99 °C; ^1^H NMR (400 MHz, acetone-*d*_6_) δ 10.07 (d, *J* = 24.8 Hz, 2H), 7.96 (t, *J* = 5.8 Hz, 1H), 7.66 (d, *J* = 7.9 Hz, 1H), 7.57 (d, *J* = 7.8 Hz, 1H), 7.40–7.32 (m, 3H), 7.27 (tt, *Js* = 13.0, 3.1 Hz, 4H), 7.17 (td, *J* = 7.1, 2.0 Hz, 1H), 7.11–7.05 (m, 3H), 7.04–6.97 (m, 2H), 3.74 (q, *J* = 7.2 Hz, 2H), 3.44 (td, *J* = 7.4, 5.7 Hz, 2H), 3.11 (t, *J* = 7.3 Hz, 2H), 2.85 (t, *J* = 7.4 Hz, 2H), 2.53 (t, *J* = 7.5 Hz, 2H). ^13^C NMR (101 MHz, acetone-*d*_6_) δ 172.8, 170.5, 140.0, 138.3, 137.7 (2C), 130.5, 130.2, 128.6, 128.5, 128.3, 126.7, 123.5, 123.3, 122.1, 122.0, 119.4 (3C), 119.3, 113.4, 113.3, 112.2, 112.1, 41.0, 40.7, 38.1, 29.4, 26.2, 26.2. IR (neat): ṽ = 3254, 1629, 1521, 1454, 1226, 1095, 739, 423 cm^–1^. HRMS (ESI) *m*/*z* (M+H)^+^ calcd for C_30_H_31_N_4_O_2_ 479.2442, found 479.2441.

***N*** **-(2-(1** ***H*** **-Indol-3-yl)­ethyl)-2-(3-((2-(benzofuran-3-yl)­ethyl)­amino)-3-oxopropyl)­benzamide (40)**

Prepared according to General procedure B using **37** and **1**; purification by column chromatography using PE/EtOAc 3:7 as eluent afforded **40** as a light brown solid (273.5 mg, 0.570 mmol, yield: 80%). Mp: 69–71 °C; ^1^H NMR (400 MHz, chloroform-*d*) δ 8.13 (s, 1H), 7.61 (d, *J* = 7.9 Hz, 1H), 7.51 (dd, *Js* = 7.6, 1.3 Hz, 1H), 7.47–7.43 (m, 1H), 7.34 (d, *J* = 8.1 Hz, 1H), 7.29 (dt, *Js* = 7.4, 1.7 Hz, 2H), 7.25–7.22 (m, 2H), 7.20 (dd, *Js* = 7.4, 1.5 Hz, 3H), 7.18–7.08 (m, 2H), 7.05 (d, *J* = 2.3 Hz, 1H), 6.36 (t, *J* = 5.9 Hz, 1H), 6.27 (t, *J* = 5.6 Hz, 1H), 3.78 (q, *J* = 6.5 Hz, 2H), 3.46 (q, *J* = 6.6 Hz, 2H), 3.07 (t, *J* = 6.6 Hz, 2H), 2.94 (t, *J* = 7.4 Hz, 2H), 2.74 (t, *J* = 6.6 Hz, 2H), 2.49 (t, *J* = 7.4 Hz, 2H). ^13^C NMR (101 MHz, chloroform-*d*) δ 172.6, 170.4, 155.4, 141.8, 138.7, 136.6, 136.2, 130.1, 129.9, 128.0, 127.4, 127.2, 126.5, 124.4, 122.5, 122.4, 122.2, 119.6, 119.4, 118.7, 117.4, 112.6, 111.5, 111.5, 40.1, 38.7, 37.8, 28.6, 25.3, 23.7. IR (neat): ṽ = 3394, 3270, 2924, 1630, 1521, 1451, 1433, 1090, 739 cm^–1^. HRMS (ESI) *m*/*z* (M+H)^+^ calcd for C_30_H_30_N_3_O_3_ 480.2282, found 480.2282.

***N*** **-(2-(1** ***H*** **-Benzo­[** ***d*** **]­imidazol-1-yl)­ethyl)-2-(3-((2-(benzofuran-3-yl)­ethyl)­amino)-3-oxopropyl)­benzamide (41)**

Prepared according to General procedure B using **37** and **38**; purification by column chromatography using EtOAc/MeOH 9:1 as eluent afforded **41** as a yellow solid (42.9 mg, 0.089 mmol, yield: 43%). ^1^H NMR (400 MHz, chloroform-*d*) δ 7.86–7.78 (m, 2H), 7.68 (d, *J* = 7.8 Hz, 1H), 7.48 (ddd, *Js* = 14.5, 8.1, 1.8 Hz, 3H), 7.29 (tt, *Js* = 5.9, 2.3 Hz, 4H), 7.24 (d, *J* = 7.6 Hz, 3H), 7.18–7.11 (m, 2H), 6.50 (t, *J* = 5.9 Hz, 1H), 4.41 (t, *J* = 5.9 Hz, 2H), 3.83 (q, *J* = 5.9 Hz, 2H), 3.45 (q, *J* = 6.5 Hz, 2H), 2.86 (t, *J* = 7.1 Hz, 2H), 2.75 (t, *J* = 6.7 Hz, 2H), 2.48 (t, *J* = 7.1 Hz, 2H). ^13^C NMR (101 MHz, chloroform-*d*) δ 172.6, 170.7, 155.4, 143.6, 143.2, 141.9, 138.4, 135.7, 133.8, 130.4, 129.6, 128.0 (2C), 126.5, 124.5, 123.3, 122.6, 122.5, 120.2, 119.6, 117.4, 111.6, 109.8, 44.1, 39.6, 38.8, 37.4, 28.0, 23.7. IR (neat): ṽ = 3260, 3066, 2884, 1656, 1636, 1560, 1540, 1431, 741, 705 cm^–1^. HRMS (ESI) *m*/*z* calculated for C_29_H_29_N_4_O_3_ [M + H]^+^ 481.2234, found 481.2235.

**Methyl 2-(((** ***tert*** **-butoxycarbonyl)­amino)­methyl)­benzoate (49)**

Methyl 2-(aminomethyl)­benzoate hydrochloride (**48**, 200 mg, 0.99 mmol) was dissolved in dry THF (4 mL) at room temperature under a nitrogen atmosphere. TEA (0.27 mL, 1.98 mmol) and di-*tert*-butyl dicarbonate (216.45 mg, 0.99 mmol) were then added sequentially. The resulting mixture was stirred overnight. Upon completion, the volatile was removed under reduced pressure, and the resulting residue was washed with a saturated aqueous NH_4_Cl solution and extracted with DCM (3x). The separated organic layer was dried over sodium sulfate, filtered, and evaporated under reduced pressure. The crude material was purified by column chromatography using PE/EtOAc 8:2 as eluent, giving **49** as a light-colored oil (yield: 99%). ^1^H NMR (400 MHz, CDCl_3_): δ 7.99 (d, *J* = 7.7 Hz, 1H), 7.53–7.49 (m, 2H), 7.38–7.34 (m, 1H), 5.59 (br s, 1H), 4.53 (d, *J* = 6.2 Hz, 2H), 3.93 (s, 3H), 1.44 (s, 9H). LRMS (ESI) *m*/*z* (M+Na)^+^ calcd for C_14_H_19_NO_4_Na 288, found 288.

***tert*** **-Butyl 2-((2-(1** ***H*** **-indol-3-yl)­ethyl)­carbamoyl)­benzylcarbamate (70)**

To a solution of Cs_2_CO_3_ (1005 mg, 3.22 mmol) in MeOH (5.37 mL), **49** (285 mg, 1.07 mmol) and **1** (206.5 mg, 1.29 mmol) were added sequentially. The mixture was stirred for 4 h at reflux. Then, the reaction was cooled at room temperature and evaporated under *vacuum*. Then the reaction was extracted with EtOAc (3×) and washed with water. The organic layer was dried over sodium sulfate, filtered, and concentrated under reduced pressure. The crude material was purified by column chromatography using PE/EtOAc 6:4 as eluent, affording **70** as a yellow solid (yield: 24%). ^1^H NMR (400 MHz, chloroform-*d*): δ 8.52 (br s, 1H), 7.64 (d, *J* = 7.3 Hz, 1H), 7.41 (d, *J* = 7.7 Hz, 1H), 7.37–7.34 (m, 2H), 7.27 (d, *J* = 7.7 Hz, 1H), 7.24–7.18 (m, 2H), 7.16–7.11 (m, 1H), 7.05 (s, 1H), 6.44 (br s, 1H), 5.81 (br s, 1H), 4.30 (d, *J* = 6.2 Hz, 2H), 3.78 (q, *J* = 6.5 Hz, 2H), 3.10 (t, *J* = 6.5 Hz, 2H), 1.44 (s, 9H). LRMS (ESI) *m*/*z* (M+Na)^+^ calcd for C_23_H_27_N_3_NaO_3_ 416, found 416.

***N*** **-(2-(1** ***H*** **-Indol-3-yl)­ethyl)-2-(aminomethyl)­benzamide trifluoroacetic acid (50)**

Prepared according to General procedure E starting from **70**; purification through column chromatography using EtOAc/MeOH 7:3 as eluents afforded **50** as a yellow solid (yield: 40%). ^1^H NMR (400 MHz, methanol-*d*_4_): δ 7.59 (d, *J* = 7.9 Hz, 1H), 7.55–7.42 (m, 4H), 7.35 (d, *J* = 8.1 Hz, 1H), 7.14–7.04 (m, 2H), 6.96 (t, *J* = 7.9 Hz, 1H), 3.91 (s, 2H), 3.69 (t, *J* = 7.1 Hz, 2H), 3.07 (t, *J* = 7.1 Hz, 2H). LRMS (ESI) *m*/*z* (M+H)^+^ calcd for C_18_H_20_N_3_O 294, found 294.

***N*** **-(2-(1** ***H*** **-Indol-3-yl)­ethyl)-3-aminoisonicotinamide (51)**

Prepared according to General procedure B using **1** and **43**; purification by column chromatography using PE/EtOAc 3:7 as eluent afforded **51** as a brown solid (yield: 74%). ^1^H NMR (400 MHz, acetone-*d*_6_) δ 10.03 (s, 1H), 8.19 (s, 1H), 7.93 (s, 1H), 7.77–7.73 (m, 1H), 7.64 (d, *J* = 7.8 Hz, 1H), 7.38 (d, *J* = 8.1 Hz, 1H), 7.30 (d, *J* = 5.1 Hz, 1H), 7.21 (d, *J* = 2.0 Hz, 1H), 7.13–7.06 (m, 1H), 7.05–6.97 (m, 1H), 6.30 (s, 2H), 3.72–3.65 (m, 2H), 3.07 (t, *J* = 7.4 Hz, 2H). HRMS (ESI) *m*/*z* (M+H)^+^ calcd for C_16_H_17_N_4_O 281.1397, found 281.1397.

***N*** **-(2-(1** ***H*** **-Indol-3-yl)­ethyl)-3-aminopicolinamide (52)**

Prepared via General procedure B using **1** and **44**; purification by column chromatography using PE/EtOAc 6:4 as eluent afforded **52** as a brown solid (yield: 61%). ^1^H NMR (400 MHz, acetone-*d*_6_) δ 10.04 (s, 1H), 8.56 (s, 1H), 7.79 (dd, *Js* = 3.5, 2.2 Hz, 1H), 7.74 (d, *J* = 7.9 Hz, 1H), 7.43 (d, *J* = 8.1 Hz, 1H), 7.24 (d, *J* = 2.3 Hz, 1H), 7.19 (dd, *Js* = 2.9, 1.3 Hz, 2H), 7.17–7.12 (m, 1H), 7.10–7.05 (m, 1H), 6.73 (s, 2H), 3.85–3.71 (m, 2H), 3.12 (t, *J* = 7.3 Hz, 2H). HRMS (ESI) *m*/*z* (M+H)^+^ calcd for C_16_H_17_N_4_O 281.1397, found 281.1398.

***N*** **-(2-(1** ***H*** **-Indol-3-yl)­ethyl)-4-aminonicotinamide (53)**

Prepared according to General procedure B using **1** and **45**; purification by column chromatography using PE/EtOAc 7:3 as eluent afforded **53** as a brown solid (yield: 25%). ^1^H NMR (400 MHz, Acetone-*d*_6_) δ 10.02 (s, 1H), 8.53 (s, 1H), 8.03 (d, *J* = 5.8 Hz, 1H), 7.83 (s, 1H), 7.65 (d, *J* = 7.9 Hz, 1H), 7.38 (d, *J* = 8.1 Hz, 1H), 7.21 (d, *J* = 2.2 Hz, 1H), 7.14–7.06 (m, 1H), 7.05–6.98 (m, 1H), 6.94 (s, 2H), 6.65 (d, *J* = 5.8 Hz, 1H), 3.74–3.65 (m, 2H), 3.08 (t, *J* = 7.3 Hz, 2H). HRMS (ESI) *m*/*z* (M+H)^+^ calcd for C_16_H_17_N_4_O 281.1397, found 281.1394.

***tert*** **-Butyl ((1** ***S***,**2** ***R*** **)-2-((2-(1** *H* **-indol-3-yl)­ethyl)­carbamoyl)­cyclohexyl)­carbamate (71)**

Prepared according to General procedure B using **1** and **46**; purification by column chromatography using PE/DCM/EtOAc 7:1:2 as eluent afforded **71** as a brown solid (yield: 99%). ^1^H NMR (400 MHz, chloroform-*d*) δ 8.26 (s, 1H), 7.62–7.56 (m, 1H), 7.37 (d, *J* = 8.1 Hz, 1H), 7.20 (t, *J* = 8.0 Hz, 1H), 7.12 (t, *J* = 7.5 Hz, 1H), 7.03 (s, 1H), 5.81 (s, 1H), 3.79 (s, 1H), 3.63–3.51 (m, 2H), 2.96 (t, *J* = 6.6 Hz, 2H), 2.44 (q, *J* = 5.1 Hz, 1H), 1.97 (q, *Js* = 10.1, 8.3 Hz, 1H), 1.68–1.61 (m, 2H), 1.60–1.49 (m, 4H), 1.43 (s, 9H), 0.97–0.85 (m, 2H). HRMS (ESI) *m*/*z* (M+H)^+^ calcd for C_22_H_32_N_3_O_3_ 386.2438, found 386.2439.

**(1** *S*,**2** ***R*** **)-** ***N*** **-(2-(1** *H* **-Indol-3-yl)­ethyl)-2-aminocyclohexane-1-carboxamide trifluoroacetic acid (54)**

Prepared according to General procedure E starting from **71**; precipitation with cold diethyl ether afforded **54** as a light-yellow solid (yield: 64%). ^1^H NMR (400 MHz, acetone-*d*_6_) δ 10.04 (s, 1H), 8.44 (s, 1H), 7.61 (d, *J* = 7.8 Hz, 1H), 7.37 (d, *J* = 8.1 Hz, 1H), 7.16 (s, 1H), 7.08 (t, *J* = 7.4 Hz, 1H), 7.00 (t, *J* = 7.4 Hz, 1H), 3.72 (dd, *J*_*s*_ = 8.4, 4.0 Hz, 1H), 3.60–3.47 (m, 2H), 2.91 (td, *J*_*s*_ = 7.1, 2.1 Hz, 2H), 2.34 (d, *J* = 3.7 Hz, 2H), 1.81–1.73 (m, 1H), 1.68–1.60 (m, 1H), 1.57–1.48 (m, 2H), 1.43–1.33 (m, 4H). HRMS (ESI) *m*/*z* (M+H)^+^ calcd for C_17_H_24_N_3_O 286.1914, found 286.1914.

**2-Amino-** ***N*** **-(2-(indolin-3-yl)­ethyl)­benzamide hydrochloric acid (55)**

Prepared according to General procedure B using **42** and **22**; purification by column chromatography using EtOAc as eluent afforded **55** as a brown solid (yield: 77%). After purification, the product was salificated using a 4N solution of HCl in dioxane (1.78 mL for 0.711 mmol of **55**). ^1^H NMR (400 MHz, acetone-*d*_6_) δ 7.61 (s, 1H), 7.51 (d, *J* = 7.9 Hz, 1H), 7.18–7.08 (m, 2H), 6.95 (t, *J* = 7.6 Hz, 1H), 6.76 (d, *J* = 8.2 Hz, 1H), 6.67–6.45 (m, 3H), 6.25 (s, 2H), 4.82 (s, 1H), 3.73 (t, *J* = 8.4 Hz, 1H), 3.57–3.44 (m, 2H), 3.37–3.32 (m, 1H), 3.32–3.24 (m, 1H), 2.19–2.09 (m, 1H), 1.83 (tt, *Js* = 13.7, 7.0 Hz, 1H). HRMS (ESI) *m*/*z* (M+H)^+^ calcd for C_17_H_20_N_3_O 282.1601, found 282.1600.

***N*** **-(2-(1** ***H*** **-Indol-3-yl)­ethyl)-2-amino-4,5-dichlorobenzamide (56)**

Prepared according to General procedure B using **1** and **47**; purification by column chromatography using PE/EtOAc 7:3 as eluent afforded **56** as a brown solid (yield: 80%). ^1^H NMR (400 MHz, acetone-*d*_6_) δ 10.00 (s, 1H), 7.85 (s, 1H), 7.68–7.59 (m, 2H), 7.38 (d, *J* = 8.1 Hz, 1H), 7.20 (d, *J* = 2.2 Hz, 1H), 7.10 (t, *J* = 7.9 Hz, 1H), 7.02 (t, *J* = 7.2 Hz, 1H), 6.98 (s, 1H), 6.55 (s, 2H), 3.72–3.63 (m, 2H), 3.06 (t, *J* = 7.3 Hz, 2H). HRMS (ESI) *m*/*z* (M-H)^−^ calcd for C_17_H_14_Cl_2_N_3_O 346.0519, found 346.0523.

**Phenyl 4,5-dichloro-6-oxopyridazine-1­(6** ***H*** **)-carboxylate (59)**

To a solution of **58** (3.03 mmol) and TEA (3.64 mmol) in dry DCM (30 mL), **57** (3.03 mmol) was added dropwise at 5 °C. The resulting mixture was stirred for 20 min at the same temperature. After evaporating the solvent under reduced pressure, the crude product was extracted with diethyl ether/water (7:2, v/v). The organic layer was dried over sodium sulfate and concentrated under reduced pressure. The resulting residue was recrystallized from THF/*n*-hexane (1:3, v/v) to afford **59** as a white powder (yield: 90%). ^1^H NMR (400 MHz, chloroform-*d*) δ 7.90 (s, 1H), 7.45 (t, *J* = 7.9 Hz, 2H), 7.33 (t, *J* = 7.4 Hz, 3H). HRMS (ESI) *m*/*z* (M+H)^+^ calcd for C_11_H_7_Cl_2_N_2_O_3_ 284.9828, found 284.9826.

***N*** **-(2-(1** ***H*** **-Indol-3-yl)­ethyl)-2-((3-(2-(1** ***H*** **-indol-3-yl)­ethyl)­ureido)­methyl)­benzamide (60)**

Prepared according to General procedure F using **50**; purification by column chromatography using PE/EtOAc 6:4 as eluent afforded **60** as a yellow solid (40.6 mg, 0.085 mmol, yield: 54%). Mp: 95–97 °C; ^1^H NMR (400 MHz, acetone-*d*_6_): δ 10.08 (br s, 1H), 10.03 (br s, 1H), 8.24 (br s, 1H), 7.67 (d, *J* = 7.9 Hz, 1H), 7.60 (d, *J* = 8.7 Hz, 1H), 7.49–7.43 (m, 2H), 7.41–7.33 (m, 3H), 7.27–7.21 (m, 2H), 7.14–7.05 (m, 3H), 7.05–6.96 (m, 2H), 6.26 (br s, 1H), 5.94 (br s, 1H), 4.40 (d, *J* = 6.2 Hz, 2H), 3.74 (q, *J* = 6.5 Hz, 2H), 3.46 (q, *J* = 6.9 Hz, 2H), 3.12 (t, *J* = 6.5 Hz, 2H), 2.92 (t, *J* = 6.9 Hz, 2H). ^13^C NMR (101 MHz, acetone-*d*_6_): δ 169.2, 158.5, 139.0, 136.9, 136.5, 129.7 (2C), 129.5, 127.8, 127.4, 126.6 (2C), 122.4, 121.2 (2C), 121.1, 118.5 (4C), 112.7, 112.6, 111.3, 111.2, 41.7, 40.8, 40.3, 26.2, 25.3. IR (neat): ṽ = 3398, 3270, 3052, 2925, 1628, 1525, 1433, 1261, 1009, 733, 577, 423 cm^–1^. HRMS (ESI) *m*/*z* (M-H)^−^ calcd for C_29_H_28_N_5_O_2_ 478.2248, found 478.2249.

***N*** **-(2-(1** ***H*** **-Indol-3-yl)­ethyl)-3-(3-(2-(1** ***H*** **-indol-3-yl)­ethyl)­ureido)­isonicotinamide (61)**

Prepared according to General procedure F using **51**; purification by column chromatography using EtOAc as eluent afforded the title compound as a brown solid (127.0 mg, 0.273 mmol, yield: 62%). Mp: 131–133 °C; ^1^H NMR (400 MHz, acetone-*d*_6_) δ 10.10–9.90 (m, 3H), 9.74 (s, 1H), 8.23 (s, 1H), 8.14 (d, *J* = 5.1 Hz, 1H), 7.69–7.57 (m, 2H), 7.44 (d, *J* = 5.1 Hz, 1H), 7.42–7.33 (m, 2H), 7.21 (dd, *Js* = 5.3, 2.2 Hz, 2H), 7.09 (dtd, *Js* = 7.9, 6.7, 1.3 Hz, 2H), 7.01 (ddt, *Js* = 8.0, 7.0, 1.1 Hz, 2H), 3.69 (td, *Js* = 7.2, 5.7 Hz, 2H), 3.58 (td, *Js* = 7.4, 5.7 Hz, 2H), 3.08 (t, *J* = 7.2 Hz, 2H), 3.03 (t, *J* = 7.4 Hz, 2H). ^13^C NMR (101 MHz, acetone-*d*_6_) δ 168.1, 155.6, 144.2 (2C), 137.7 (3C), 128.7, 128.5, 126.1, 123.5, 123.4, 122.2, 122.1, 120.9, 119.5, 119.4 (2C), 119.2, 113.5, 113.1, 112.2, 112.1, 41.6, 41.3, 26.8, 25.8. IR (neat): ṽ = 3272, 2921, 2169, 1982, 1593, 1549, 1412, 1218, 1073, 1008, 806, 726, 670, 477, 423 cm^–1^. HRMS (ESI) *m*/*z* (M+H)^+^ calcd for C_27_H_27_N_6_O_2_ 467.2190, found 467.2193.

***N*** **-(2-(1** ***H*** **-Indol-3-yl)­ethyl)-3-(3-(2-(1** ***H*** **-indol-3-yl)­ethyl)­ureido)­picolinamide (62)**

Prepared according to General procedure F using **52**; purification by column chromatography using PE/EtOAc 7:3 as eluent afforded **62** as an off-white solid (96 mg, 0.206 mmol, yield: 59%). Mp: 163–165 °C; ^1^H NMR (400 MHz, Acetone-*d*_6_) δ 11.35 (s, 1H), 10.03 (d, *J* = 16.0 Hz, 2H), 9.14–9.00 (m, 1H), 8.83 (s, 1H), 8.08 (dd, *J* = 4.3, 1.2 Hz, 1H), 7.69 (dd, *J* = 7.7, 5.1 Hz, 2H), 7.47–7.43 (m, 1H), 7.40 (t, *J* = 8.1 Hz, 2H), 7.24 (s, 2H), 7.12 (q, *J* = 7.4 Hz, 2H), 7.04 (td, *J* = 7.4, 2.7 Hz, 2H), 6.89 (s, 1H), 3.75 (q, *J* = 7.0 Hz, 2H), 3.65–3.50 (m, 2H), 3.11 (t, *J* = 7.4 Hz, 2H), 3.06 (t, *J* = 7.5 Hz, 2H). ^13^C NMR (101 MHz, DMSO-*d*_6_) δ 166.9, 154.8, 139.9, 139.5, 136.3, 136.2, 131.9, 127.3, 127.2 (2C), 127.0, 122.6, 122.5, 121.0, 120.9, 118.4, 118.3, 118.2 (2C), 111.9, 111.6, 111.4 (2C), 25.6 (2C), 25.1 (2C). IR (neat): ṽ = 3326, 2916, 2360, 1651, 1506, 1215, 1100, 737, 585, 421 cm^–1^. HRMS (ESI) *m*/*z* (M+H)^+^ calcd for C_27_H_27_N_6_O_2_ 467.2190, found 467.2196.

***N*** **-(2-(1** ***H*** **-Indol-3-yl)­ethyl)-4-(3-(2-(1** ***H*** **-indol-3-yl)­ethyl)­ureido)­nicotinamide (63)**

Prepared according to General procedure F using **53**; purification by column chromatography using PE/EtOAc 3:7 as eluent afforded **63** as an off-white solid (115.6 mg, 0.248 mmol, yield: 58%). Mp: 220–221 °C; ^1^H NMR (400 MHz, DMSO-*d*_6_): δ 10.82 (d, *J* = 7.3 Hz, 2H), 10.32 (br s, 1H), 8.93 (br s, 1H), 8.66 (s, 1H), 8.35 (dd, *J*s = 23.8, 0.8 Hz, 2H), 7.87 (br s, 1H), 7.56 (t, *J* = 6.9 Hz, 2H), 7.37–7.30 (m, 2H), 7.21–7.14 (m, 2H), 7.09–7.03 (m, 2H), 7.01–6.95 (m, 2H), 3.56 (q, *J* = 7.0 Hz, 2H), 3.37 (q, 2H), 2.99 (t, *J* = 7.4 Hz, 2H), 2.87 (t, *J* = 7.4 Hz, 2H). ^13^C NMR (101 MHz, DMSO-*d*_6_): δ 167.0, 154.2, 151.6, 148.9, 147.4, 136.3 (2C), 127.3 (2C), 122.8, 122.7, 121.0, 120.9, 118.3 (2C), 118.2 (2C), 114.7, 112.5, 111.8, 111.7, 111.4 (2C), 40.2, 38.9, 25.5, 24.9. IR (neat): ṽ = 3311, 3055, 2919, 2851, 1646, 1538, 1455, 1253, 1225, 1042, 938, 849, 741, 659, 547, 423 cm^–1^. HRMS (ESI) *m*/*z* (M+H)^+^ calcd for C_27_H_27_N_6_O_2_ 467.2190, found 467.2192.

**(1** ***S***,**2** ***R*** **)-** ***N*** **-(2-(1** ***H*** **-Indol-3-yl)­ethyl)-2-(3-(2-(1** ***H*** **-indol-3-yl)­ethyl)­ureido)­cyclohexane-1-carboxamide (64)**

Prepared according to General procedure F using **54**; purification by column chromatography using PE/EtOAc 4:6 as eluent afforded **64** as a white solid (28.7 mg, 0.061 mmol, yield: 51%). Mp: 135–136 °C. ^1^H NMR (400 MHz, acetone-*d*_6_) δ 10.01 (s, 2H), 7.62–7.56 (m, 2H), 7.39–7.33 (m, 2H), 7.33–7.26 (m, 1H), 7.16 (d, *J* = 2.1 Hz, 1H), 7.12 (d, *J* = 2.2 Hz, 1H), 7.10–7.04 (m, 3H), 7.02–6.95 (m, 2H), 5.82 (t, *J* = 5.8 Hz, 1H), 5.75 (d, *J* = 8.2 Hz, 1H), 4.17–4.04 (m, 1H), 3.57–3.39 (m, 4H), 2.92 (h, *Js* = 8.1, 7.6 Hz, 4H), 2.57 (dt, *Js* = 8.3, 4.0 Hz, 1H), 1.83–1.70 (m, 1H), 1.61 (tdd, *Js* = 19.6, 9.6, 5.7 Hz, 3H), 1.48 (td, *Js* = 8.6, 4.0 Hz, 1H), 1.38 (ddd, *Js* = 16.4, 7.7, 3.7 Hz, 2H). ^13^C NMR (101 MHz, acetone-*d*_6_) δ 174.3, 158.7, 137.7 (2C), 128.7, 128.6, 123.5, 123.3, 122.0 (2C), 119.5, 119.4 (3C), 113.8, 113.5, 112.1, 112.0, 54.9, 48.9, 46.5, 41.5, 40.5, 31.4, 27.2, 26.4, 24.2, 23.3. IR (neat): ṽ = 3301, 2923, 2854, 2360, 1630, 1522, 1454, 1308, 1223, 1094, 1009, 801, 737, 582, 423 cm^–1^. HRMS (ESI) *m*/*z* (M+H)^+^ calcd for C_28_H_34_N_5_O_2_ 472.2707, found 472.2711.

**2-(3-(2-(1** ***H*** **-Indol-3-yl)­ethyl)­ureido)-** ***N*** **-(2-(indolin-3-yl)­ethyl)­benzamide (65)**

Prepared according to General procedure F using **55**; purification by column chromatography using PE/EtOAc 4:6 as eluent afforded **65** as an off-white solid (55.0 mg, 0.118 mmol, yield: 60%). Mp: 125–127 °C; ^1^H NMR (400 MHz, acetone-*d*_6_): δ 10.28 (br s, 1H), 9.99 (br s, 2H), 8.03 (t, *J* = 7.2 Hz, 2H), 7.69–7.62 (m, 2H), 7.38 (d, *J* = 8.0 Hz, 1H), 7.31–7.16 (m, 4H), 7.12–7.05 (m, 2H), 7.02 (t, *J* = 7.3 Hz, 1H), 6.84 (t, *J* = 7.3 Hz, 1H), 6.19 (br s, 1H), 4.20–4.10 (m, 2H), 4.07–3.99 (m, 1H), 3.83–3.75 (m, 1H), 3.64–3.53 (m, 2H), 3.50–3.41 (m, 1H), 3.04 (t, *J* = 7.3 Hz, 2H), 2.24–2.13 (m, 1H), 1.91–1.79 (m, 1H). ^13^C NMR (101 MHz, acetone-*d*_6_): δ 163.2, 155.8, 145.5, 140.6, 137.9, 135.8, 134.6, 128.9, 128.7, 128.3, 124.6, 123.5, 122.2, 122.0, 119.5 (2C), 115.9, 115.8, 115.7, 115.5, 113.8, 112.2, 53.8, 42.0, 39.1, 38.8, 34.4, 27.0. IR (neat): ṽ = 3294, 3054, 2926, 1708, 1645, 1517, 1481, 1447, 1340, 1262, 1093, 1060, 801, 741, 692, 460, 423 cm^–1^. HRMS (ESI) *m*/*z* (M+Na)^+^ calcd for C_28_H_29_N_5_O_2_Na 490.2213, found 490.2218.

***N*** **-(2-(1** ***H*** **-Indol-3-yl)­ethyl)-2-(3-(2-(1** ***H*** **-indol-3-yl)­ethyl)­ureido)­benzamide (66)**

Prepared according to General procedure F using **23**; purification by column chromatography using PE/EtOAc 6:4 as eluent afforded **66** as a white solid (81.2 mg, 0.175 mmol, yield: 81%). Mp: 165–167 °C; ^1^H NMR (400 MHz, acetone-*d*_6_) δ 10.47 (s, 1H), 9.99 (d, *J* = 10.6 Hz, 2H), 8.54 (dd, *Js* = 8.6, 1.2 Hz, 1H), 7.94 (s, 1H), 7.70–7.62 (m, 2H), 7.59 (dd, *Js* = 7.8, 1.6 Hz, 1H), 7.44–7.33 (m, 3H), 7.21 (dd, *Js* = 4.6, 2.4 Hz, 2H), 7.09 (dtd, *Js* = 8.1, 6.8, 1.3 Hz, 2H), 7.06–6.98 (m, 2H), 6.88 (td, *Js* = 7.6, 1.2 Hz, 1H), 6.60 (s, 1H), 3.69 (td, *Js* = 7.4, 5.7 Hz, 2H), 3.60–3.53 (m, 2H), 3.10–3.01 (m, 4H). ^13^C NMR (101 MHz, acetone-*d*_6_) δ 169.9, 156.0, 142.8, 137.7 (2C), 132.4, 128.7, 128.6, 128.1, 123.4, 123.3, 122.1 (2C), 120.8 (2C), 120.4, 119.5, 119.4 (2C), 119.3, 113.6, 113.3, 112.2, 112.1, 41.6, 41.2, 26.9, 26.0. IR (neat): 3418, 3270, 3222, 2922, 2849, 1625, 1550, 1507, 1436, 1272, 735, 488 cm^–1^. HRMS (ESI) *m*/*z* (M+H)^+^ calcd for C_28_H_28_N_5_O_2_ 466.2238, found 466.2235.

***N*** **-(2-(1** ***H*** **-Indol-3-yl)­ethyl)-2-(3-(2-(1** *H* **-indol-3-yl)­ethyl)­ureido)-4,5-dichlorobenzamide (67)**

Prepared via General procedure F using **56**; purification by column chromatography using PE/EtOAc 4:6 as eluent afforded **67** as a white solid (124.0 mg, 0.232 mmol, yield: 67%). Mp: 115–118 °C; ^1^H NMR (400 MHz, acetone-*d*_6_) δ 10.47 (s, 1H), 10.01 (d, *J* = 14.0 Hz, 2H), 8.88 (s, 1H), 8.16 (s, 1H), 7.75 (s, 1H), 7.63 (dd, *Js* = 15.1, 7.8 Hz, 2H), 7.38 (dd, *Js* = 8.0, 6.3 Hz, 2H), 7.21 (s, 2H), 7.09 (d, *J* = 7.3 Hz, 2H), 7.02 (t, *J* = 7.4 Hz, 2H), 6.88 (s, 1H), 3.68 (td, *Js* = 7.2, 5.7 Hz, 2H), 3.61–3.52 (m, 2H), 3.05 (dt, *Js* = 19.6, 7.3 Hz, 4H). ^13^C NMR (101 MHz, acetone-*d*_6_) δ 168.1, 155.6, 142.7, 137.9, 135.7, 129.9, 128.8, 123.6, 123.5, 123.0, 122.3, 122.2 (2C), 121.8, 120.2, 119.6 (2C), 119.5 (2C), 119.4, 113.6, 113.3, 112.3, 112.2, 41.7, 41.6, 26.9, 25.9. IR (neat): ṽ = 3398, 3053, 2921, 2851, 1962, 1637, 1497, 1455, 1258, 1224, 1092, 740, 422 cm^–1^. HRMS (ESI) *m*/*z* calcd for C_28_H_24_Cl_2_N_5_O_2_ (M-H)^−^ 532.1313, found 532.1317.

### Cell Lines and Cell Culture

A375 and SW-48 cells were grown with DMEM supplemented with 10% fetal bovine serum (FBS), 2 mM l-glutamine, 100 U/mL penicillin, and 100 mg/mL streptomycin (GE healthcare, Milan, Italy). U87-MG and HepG2 cell lines were maintained in Eagle’s Minimum Essential Medium (EMEM) completed with 10% FBS, 1 mM glutamine (Gibco, Invitrogen CA, USA), and penicillin/streptomycin (Gibco, Invitrogen CA, USA). The human ovarian adenocarcinoma cell line SKOV-3 was grown in RPMI-1640 medium supplemented with 10% fetal calf serum (FCS), 2 mM l-glutamine, 100 U/mL penicillin, and 100 mg/mL streptomycin (Thermo Fisher Scientific, Walthman, MA, USA). The human cell lines were maintained at 37 °C in a humidified 5% CO_2_ incubator. For all cellular assays, the cell line was used at a passage number not exceeding the 10th.

### Cell Viability

Cell viability was measured by the 3-(4,5-dimethylthiazol-2-yl)-2,5-diphenyl-tetrazolium bromide (MTT) assay. Cells were seeded (5 × 10^3^ cells/well) into a flat-bottom 96-well plate and treated with increasing concentrations (0.01–10 μM) of each tested compound, linrodostat and epacadostat, followed by incubation for 48 h. After that, a solution of 5 mg/mL MTT was added to each well to reach a final concentration of 10% (v/v), and cells were incubated for 30 min. Subsequently, the supernatants were discarded, and the resulting formazan crystals were solubilized in DMSO. Absorbance was measured using a microplate spectrophotometer at a dual wavelength of 570/630 nm. Cell viability was calculated using the formula: Viability (%) = 100*­(x-y)/(z-y), where x is the absorbance of compound-treated cells, y is the absorbance of resting/unstimulated cells, and z is the absorbance of untreated cells. The assay was performed in triplicate for each condition.

### Cell Treatment

A total of 5 × 10^3^ A375, U87, and HepG2 cells were seeded into 96-well plates and incubated for 4 h to allow cell adhesion. Cells were then treated with increasing concentrations (0.01–10 μM) of each compound and simultaneously stimulated with 1000 U/mL recombinant human IFNγ (PeproTech) to induce IDO1 expression. All compounds were initially dissolved in 100% DMSO (Sigma-Aldrich) and then diluted to a final concentration of 0.1% DMSO in the culture medium. An equivalent amount of DMSO was added to untreated cells. After 48 h, the plates were centrifuged at 1200 rpm for 5 min, and the supernatants were collected and stored at −80 °C until analysis. Identical experimental conditions were applied to cells cultured in the absence of IFNγ stimulation.

For determination of IDO1 catalytic activity, SKOV-3 were seeded in 24-well plate at 0.15 × 10^6^ cells/ml and conditioned with **66** (10 μM), linrodostat (1 μM), or vehicle for 16 h at 37 °C. After incubation, cell culture supernatants were analyzed by HPLC, as previously described.^9^

### HPLC Conditions for IDO1 Inhibitors Cell-Based Screening in A375 Cell Line

Detection of Kyn concentrations was performed by using a PerkinElmer, series 200 HPLC instrument (MA, USA). A Kinetex C18 column (250 × 4.6 mm, 5 μm, 100 Å; Phenomenex, USA), maintained at the temperature of 25 °C and pressure of 1800 PSI, was used. A sample volume of 20 μL was injected and eluted by a mobile phase containing 10 mM NaH_2_PO_4_ pH 4.8 (90%) and acetonitrile (10%) (Sigma-Aldrich, MO, USA), with a flow rate of 1 mL/min. Kyn was detected at 330 nm by a UV detector. The software TURBOCHROM 4 was used for evaluating the concentration of Kyn in samples using a calibration curve. The detection limit of the analysis was 0.05 μM.

### Metabolomic Analysis in A375, HepG2, and U87 Cell Lines

Quantitative targeted metabolomics of Kyn catabolites in cell supernatants was performed via LC-MS/MS using the internally validated method previously published by Villani et al.^37^ IC_50_ plots were generated using the “[Inhibitor] vs. response (three parameters)” function in GraphPad Prism software. The *x*-axes represent the logarithm base 10 of the inhibitor concentrations, while the *y*-axes depict the inhibition percentage, calculated as Kyn/Trp ratios and normalized by setting the maximal and basal controls (100% and 0% CTRLs), corresponding to IFNγ treatment and basal conditions, respectively. IC_50_ values were directly calculated by GraphPad Prism. Multivariate analyses were conducted using the MetaboAnalyst web platform, with Kyn concentrations log10-transformed and normalized relative to the IFN group corresponding to maximal Kyn pathway stimulation. The Ward clustering algorithm was applied to differentiate experimental conditions in heatmaps. MetaboAnalyst 6.0 is freely available at https://www.metaboanalyst.ca, with no login required.

### Phase I Metabolic Stability and Profiling

The standard incubation mixture (250 μL final volume) was carried out in a 50 mM Tris (tris­[hydroxymethyl]­aminomethane) buffer (pH 7.4) containing 150 mM KCl, 1.5 mM, 3.3 mM MgCl_2_, 1.3 mM βNADPNa_2_, 3.3 mM glucose 6-phosphate, 0.4 units/mL glucose 6-phosphate dehydrogenase, acetonitrile as cosolvent (1% of total volume), and the substrate (50 μM). After pre-equilibration of the mixture, an appropriate volume of MLM suspension was added to give a final protein concentration of 1.0 mg/mL. The mixture was shaken for 60 min at 37 °C using a horizontal shaking thermostatic bath. Control incubations were carried out without the presence of substrate, NADPH-regenerating system, or microsomes. Each incubation was stopped by the addition of 250 μL of ice-cold acetonitrile, vortexed, and centrifuged at 13,000 rpm for 5 min. The supernatants were analyzed by LC-UV (see Supporting material for instrumental settings) for the determination of substrate residue. Compounds VS-15 and **66** were further analyzed by LC-HRMS equipment for metabolites structural characterization (see Supporting material for full parameter settings). Raw data files were processed using both Xcalibur and Compound Discoverer 3.3 software (Thermo Scientific) using a customized workflow for detection and identification of the expected and unknown metabolites.

### Expression and Purification of *holo*-IDO1

The open reading frame (ORF) of wild-type human IDO1 (hIDO1) was synthesized by Genscript and subcloned into the pET28a plasmid vector for the production of an N-terminal His-tagged protein using *E. coli* BL21 (DE3) (Novagen) as the expression system. *E. coli* cells were grown at 37 °C in 2 × TY medium supplemented with kanamycin (50 μg/mL). When the culture reached an OD600 of 0.5, 5-aminolevulinic acid (5-ALA) was added to a final concentration of 1.5 mM. Upon reaching an OD600 of 0.8–1.0, protein expression was induced with 0.3 mM isopropyl-β-d-thiogalactopyranoside (IPTG), and cultures were incubated for an additional 18 h at 25 °C with shaking. Cells were harvested by centrifugation at 4500 × g at 4 °C and stored at −80 °C. The bacterial pellet obtained from 2 L of culture was thawed and resuspended in Buffer A (50 mM potassium phosphate, 300 mM KCl, 15 mM imidazole, 5% glycerol, 0.1 mM TCEP, 0.1% [v/v] Triton X-100, pH 7.2). The cells were lysed by sonication (15 min total time, 30 s on/60 s off cycles). The lysate was clarified by centrifugation at 17,000 × g for 45 min at 4 °C. The resulting supernatant was incubated with Ni^2+^-nitrilotriacetic acid (Ni-NTA) resin pre-equilibrated with Buffer A for 90 min under gentle agitation. The resin was washed with 10 column volumes of Buffer A supplemented with 40 mM imidazole to remove nonspecifically bound proteins. hIDO1 was then eluted with Buffer A containing 500 mM imidazole. Protein fractions were analyzed by SDS-PAGE, and relevant fractions were pooled and dialyzed against a buffer containing 25 mM potassium phosphate, 25 mM NaCl, 5% glycerol, and 0.1 mM EDTA (pH 6.0). The dialyzed protein was applied to a Mono S column (Cytiva) and eluted with a linear gradient of 25 mM to 1 M NaCl. Protein fractions were analyzed by SDS-PAGE, and those containing hIDO1 at high purity were pooled and concentrated to 5 mL. The sample was loaded onto a HiLoad 16/600 Superdex 200 prep-grade gel filtration column pre-equilibrated with 25 mM MES sodium salt (pH 6.5), 150 mM KCl, and 0.1 mM EDTA. Fractions corresponding to the peak of interest were further analyzed by SDS-PAGE. Highly pure hIDO1-containing aliquots were pooled, concentrated, and stored at −80 °C. The final yield was approximately 10 mg of hIDO1 per liter of bacterial culture.

### Expression and Purification of *holo*-TDO

The details of protein expression and purification are described elsewhere.^51^ Briefly, the human TDO (hTDO) protein, with truncated N- and C-terminal tails and a C-terminal 6X-His tag extension, was overexpressed in *E. coli* BL21 Star (DE3) cells using the pET24b vector purchased by Genscript. Cells were grown at 37 °C in 2xTY medium supplemented with kanamycin (50 μg/mL) and 1.5 mM 5-aminolevulinic acid (5-ALA). When the culture reached an OD600 of 0.6–0.8, protein expression was induced with 0.5 mM isopropyl-β-D-thiogalactopyranoside (IPTG), and the cells were incubated for an additional 18 h at 25 °C under shaking. Cells were harvested by centrifugation at 4,500 g for 10 min at 4 °C and stored at −80 °C. The recombinant protein was purified by Ni-NTA affinity chromatography (Novagen). The bacterial pellet was resuspended in 50 mM phosphate buffer (pH 8) containing 300 mM NaCl, 20 mM imidazole, 5% glycerol, and 10 mM l-tryptophan to stabilize the protein. Cells were disrupted by sonication (total sonication time: 15 min, with 30” ON/60” OFF cycles). The lysate was clarified by centrifugation at 17,000 g for 45 min at 4 °C, and the supernatant was incubated with Ni-NTA resin beads for 90 min under shaking. The resin was washed with 10 column volumes (CVs) of Lysis buffer containing 60 mM imidazole, and TDO was eluted with Lysis buffer containing 300 mM imidazole. Fractions containing high concentrations of TDO were pooled and loaded onto a HiLoad 16/600 Superdex 200 prep grade gel filtration column pre-equilibrated with 300 mM NaCl, 5% glycerol, pH 8. Fractions corresponding to the peak of interest were analyzed by SDS-PAGE. Aliquots containing highly pure TDO were pooled, concentrated, and stored at −80 °C. The final yield was 12 mg of pure TDO per liter of bacterial culture. The *holo* form of TDO was obtained by overnight.

### hIDO1 and hTDO Spectrophotometric Assay

By using the recombinant hIDO1 and hTDO, the cofactor dissociation was followed by measuring the decremental absorbance of the Soret peak at 404 nm for ferric hIDO1 on a Varian Cary 50 Bio UV–visible Spectrophotometer. The assay was performed at 37 °C in a total volume of 200 μL, by testing the inhibitor **66** at 25 μM, in the presence of 50 mM phosphate buffer, pH = 7, 1 mM EDTA, 5% DMSO, and 3 μM hIDO1. TDO (5 μM) was incubated with compound **66** (25 μM) at 37 °C for 10 min in 50 mM Tris-HCl pH 7.5, 1 mM EDTA, and 5% DMSO. Samples were prepared in 1.5 mL tubes and incubated for 15 min at 37 °C. Then, the heme loss was followed for 30 min.

### hIDO1 and hTDO Enzymatic Assay under Normal and Forced Conditions

The activity of recombinant IDO1 and TDO was measured following previously established methods.^24^ Briefly, 1 μM *holo*-IDO1 in 100 mM potassium phosphate buffer (pH 7.2) plus 1 mM CHAPS catalase (Sigma 02071) was added (50 μL) to inhibitors in DMSO carrier (5%) and allowed to incubate for 120 min at the temperature of 37.5 °C. After incubation, a substrate mix containing 4 mM d-tryptophan (for IDO1) or 0.5 mM l-tryptophan (for TDO), 20 mM l-ascorbic acid, and 1 μM methylene blue in 100 mM potassium phosphate buffer (pH 7.2), with 5% DMSO and 1 mM CHAPS, was added to the reaction (50 μL). The absorbance at 320 nm was measured for 30 min on a Tecan Spark microplate reader. The enzymatic characterization of the protein was performed at 25 and 37 °C with a preincubation step, and the two Michaelis–Menten constants were calculated and compared. Through this method, the residual activity was calculated and, where applicable, IC_50_ values were generated (Prism GraphPad). As a control, the inhibitor response was monitored at 25 °C without preincubation. Results are average and standard deviation of N = 2 experiments.

### Hemin–Agarose Pull-Down Assay

*Apo*-IDO1 was purified following the procedure previously described.^49^ Hemin–agarose resin (Sigma-Aldrich, H5533) was used to assess the interaction between *apo*-IDO1 and inhibitors. The resin (100 μL slurry) was transferred into a 1.5 mL microcentrifuge tube, allowed to settle by gravity, and washed three times with the *apo*-IDO1 SEC buffer (25 mM Tris-HCl pH 8.0, 500 mM NaCl, 10 mM MgCl_2_). Recombinant *apo*-IDO1 (5 μg) was incubated with the equilibrated resin in a final volume of 100 μL for 30 min at room temperature with gentle agitation. After incubation, the beads were allowed to settle and the initial flow-through was collected. An aliquot of 8 μL of the resin was directly analyzed by SDS–PAGE to verify IDO-1 binding. The IDO-1–bound resin was divided into four equal portions and incubated for 2 h at 37 °C with 100 μL of the following solutions: a) 10 mM ascorbic acid +5% DMSO + 1 mM compound **66**; b) 10 mM ascorbic acid +5% DMSO + 1 mM linrodostat; c) 10 mM ascorbic acid +5% DMSO + 1 mM epacadostat; d) 10 mM ascorbic acid +5% DMSO. At the end of the incubation, beads were left to settle and 20 μL of the final supernatant from each condition was collected for SDS–PAGE and immunoblot analysis.

### SDS–PAGE and Immunoblotting

Samples (20 μL) were mixed with 4× Laemmli buffer, heated at 95 °C for 5 min, and resolved on 12% SDS–polyacrylamide gels. Proteins were transferred onto PVDF membranes using a semidry blotting system. Membranes were blocked for 1 h in 5% (w/v) nonfat dry milk in TBS-T (20 mM Tris-HCl pH 7.5, 150 mM NaCl, 0.1% Tween-20) and incubated overnight at 4 °C with a rabbit monoclonal anti-IDO1 antibody (clone EPR20374, Abcam ab211017) diluted 1:1000 in blocking buffer. After washing, membranes were incubated with HRP-conjugated antirabbit secondary antibody for 1 h at room temperature. Signals were detected by enhanced chemiluminescence and imaged using a digital acquisition system.

### Succinyl Acetone Cell-Based Assay

A375 cells were treated with media containing 1 mM tryptophan, 50 μM succinylacetone, and 1000 U/mL of human IFNγ (PeproTech) to induce IDO1 synthesis, and either vehicle (DMSO) or increasing concentrations (0.01–30 μM) of compound **66**, epacadostat, or linrodostat for 48 h. Supernatants were collected, deproteinized by 20% (v/v) aqueous CCl_3_COOH, centrifuged at 13,000 rpm for 10 min, and the amounts of l-Kyn quantified with HPLC. Twenty μL of supernatants was injected into an HPLC system (Waters 1225 pump with Dual Absorbance Detector Water 2487), equipped with an analytical column C-18 Kinetex (150 × 4.6 mm, 5 m length). The mobile phase (50 mM potassium dihydrogen phosphate, 10% v/v acetonitrile; pH 4.8) was delivered at a flow-rate of 1 mL/min at room temperature, and the absorbance was measured at 330 nm. Amounts of l-Kyn in A375 cell media were quantified on the basis of a calibration curve obtained using the same HPLC-UV experimental setting.

In each experiment, the corresponding controls were included: IFNγ treatment alone for the standard assay, and IFNγ plus succinylacetone treatment for the succinylacetone.

### Docking *apo*-IDO1/TDO

The structure of compound **66** and VS-15 were processed as follows. An in-house script was used in order to standardize charges, enumerate ionization states, and generate tautomers at physiological pH range QUACPAC software from OpenEye (QUACPAC, version 1.6.3.1; OpenEye, Cadence Molecular Sciences, Santa Fe, NM, http://www.eyesopen.com). The latter operation was followed by a 3D structure optimization and conformers generation using OMEGA2 software^52^ (OMEGA, version 4.1.0.2; OpenEye, Cadence Molecular Sciences, Santa Fe, NM. http://www.eyesopen.com).

The X-ray structures used in this study were: for *apo*-IDO1, entry code 7B1O, chain B,^53^ and for *apo*-TDO, entry code 8QV7.^48^ Water molecules were removed, and all hydrogen atoms and MMFF94 charges were added. Then, the complex was transferred into fred_receptor and prepared for docking with FRED^54^ (FRED, version 4.0.0.2, OpenEye, Cadence Molecular Sciences, Santa Fe, NM, http://www.eyesopen.com). Docked conformations were scored using Chemgauss4.

Thermodynamic properties of water molecules in the binding site were evaluated using the SZMAP software (SZMAP, version 1.7.0.0, OpenEye, Cadence Molecular Sciences, Santa Fe, NM. http://www.eyesopen.com). Water probes were then placed on a regular grid, at each probe location, SZMAP samples multiple orientations of the water molecule. For each sampled orientation, the energy of the system is computed as the sum of several components: van der Waals interactions between the water probe and the surrounding protein and ligand, electrostatic interactions involving the ligand, protein, and the polarizable Poisson–Boltzmann continuum solvent, as well as the desolvation penalties for both the protein and the water probe. The resulting ensemble of energies and orientations is used to build a partition function, from which the probability of each sampled orientation is derived.

### Immunoprecipitation and Immunoblot Analysis

SKOV-3 cells (2.5 × 10^6^ cells/sample) were treated with compound **66** (10 μM), linrodostat (1 μM), or vehicle for 16 h at 37 °C and used for immunoprecipitation experiments, followed by Western blotting analysis, as described.^11^ Briefly, cell lysates were incubated in the presence of the mouse monoclonal anti-SH-PTP2 antibody (clone B-1, Cat #sc-7384, Santa Cruz Biotechnology, Dallas, TX, USA), overnight at 4 °C under rotary agitation. After an additional incubation of 1 h with the Protein G Sepharose 4 Fast Flow (Cytiva, Milano, Italy), the immuno-complexes were used for immunoblot analysis. The following antibodies were used for immunoblot analysis: rabbit mAb antihuman IDO1 antibody (D5J4E, Cat #86630, Cell Signaling Technology, Danvers, MA, USA), mouse mAb anti-SH-PTP2 (clone B-1, Cat #sc-7384, Santa Cruz Biotechnology, Dallas, TX, USA), mouse mAb anti-β-tubulin antibody (clone AA2, Cat# T8328, Sigma-Aldrich, Merck, Darmstadt, Germany).

### Transwell Migration Assay

Transwell migration assay in SKOV-3 cells was performed by using 8 μm polycarbonate membrane Transwell inserts (Corning, NY, USA) in 24-well plate. Briefly, 0.05 × 10^6^ cells in 150 μL of culture medium without FCS were treated with **66** (10 μM), linrodostat (1 μM), or vehicle, and seeded into the upper chamber of the inset, over either complete culture medium (10% FCS as chemoattractant) or culture medium without FCS as a negative control. After 24 h at 37 °C, cells on the lower surface of the insets were stained with 0.1% crystal violet (Sigma-Aldrich, Merck, Darmstadt, Germany) and imaged by using an EVOS M5000 Imaging System (Thermo Fisher Scientific, Waltham, MA, USA) with 10× magnification, as described.^11^ Cell migration was analyzed on raw images by automatic particle analysis (ImageJ Software, NIH), and was reported as the percentage of cell-covered area, calculated in each field of view. For each experiment, three representative fields of vision were reported.

### Scratch Wound Healing Assay

For the scratch wound healing assay, SKOV-3 cells were seeded at 0.3 × 10^6^ cells/ml in 24-well plate and preconditioned with **66** (10 μM), linrodostat (1 μM), or vehicle for 24 h at 37 °C. After incubation, scratch wound healing assay was performed as described,^11^ in the presence of stimuli or vehicle for additional 24 h. Over this incubation, the wound healings were continuously observed and imaged by using a Nikon Eclipse T*i* inverted microscope (Nikon Corporation, Tokyo, Japan) with 10× magnification. The area of the wound healing was determined by using the MRI Wound Healing tool (ImageJ Software, NIH) and the data were reported as percentage of the wound closure with respect to time 0.

### Statistical Analysis

All analyses were performed using Prism version 8.0.1 or 10.2.3 (GraphPad Software). Data usually met normality and were analyzed by two-tailed paired Student’s *t* test when two samples were under comparison or ANOVA followed by posthoc Bonferroni’s test when three or more samples were under comparison. A p-value less than 0.05 was considered significant. Overall results, obtained by at least three replicates per experimental parameter, were shown as mean ± SD.

## Supplementary Material













## Notes

Additional experimental details, LC–HRMS metabolite identification, CYP inhibition data, IDO1 protein-level analysis by Western blot, quantitative targeted metabolomics, *apo*-IDO1 and *apo*-TDO inhibition assays, FPocket-based shape analysis, murine IDO1 signaling experiments, HPLC–UV purity assessment, and ^1^H and ^13^C NMR spectra of selected compounds (PDF)
3D PDB Docking Figure 6A (PDB)
3D PDB Docking Figure 6B (PDB)
3D PDB Docking Figure 6C (PDB)
3D PDB Docking Figure 6D (PDB)
Molecular formula strings (CSV)

## ∥

Department of Drug Science and Technology, Università di Torino, Via Pietro Giuria 9, 10125 Torino, Italy

## Abbreviations

3OHAA: 3-hydroxyanthranilic acid
AA: anthranilic acid
EE: early endosome
HLM: human liver microsomes
IDO1: indoleamine 2,3-dioxygenase 1
ITIMs: tyrosine-based inhibitory motifs
KA: kynurenic acid
Kyn: kynurenine
MLM: mouse liver microsomes
NADPH: nicotinamide adenine dinucleotide phosphate
NFK: *N*-formylkynurenine
NK: natural killer cells
SBVS: structure-based virtual screening
SHP: Src homology 2 domain-containing tyrosine phosphatases
TDO: tryptophan 2,3-dioxygenase
TGF-β: transforming growth factor beta
Trp: tryptophan
XA: xanthurenic acid.
