# Supplementary material for: Discovery of a Dual IDO1/TDO Inhibitor That Modulates Enzymatic Activity and IDO1/SHP‑2 Signaling
Source: J Med Chem. 2026 Jul 22;69(15):18336–63. doi: 10.1021/acs.jmedchem.6c00759 (PMC13492295; doi:10.1021/acs.jmedchem.6c00759)
Supplement: Supplementary file 1 [file jm6c00759_si_001.pdf]

Supporting Information

Discovery of a Dual IDO1/TDO Inhibitor That Modulates Enzymatic Activity and IDO1/SHP-2 Signaling

Sarah Jane Rezzi[1,§], Andrea Butticè[1,§], Salvatore Villani[1], Gaia Lesca[1], Sofia Rossini[2], Eleonora Panfili[2], Daniele Mazzoletti[1], Elia Bari[1], Davide Violante[1], Marta Serafini[1,^], Alberto Massarotti[1], Ciriana Orabona[2], Maria Teresa Pallotta[2], Silvio Aprile[1], Silvia Fallarini[1], Erika Del Grosso[1], Rita Maria Concetta Di Martino[1,*], Riccardo Miggiano[1], Tracey Pirali[1,*]

[1]*Department of Pharmaceutical Sciences, Università degli Studi del Piemonte Orientale, Novara 28100, Italy.*

[2]*Department of Medicine and Surgery, University of Perugia, Perugia 06132, Italy.*

^ Present address: *Department of Drug Science and Technology, Università di Torino, Via Pietro Giuria 9, 10125 Torino, Italy.*

§ These authors contributed equally

* Correspondence should be addressed to:

Dr. Rita Maria Concetta Di Martino, email: rita.dimartino@uniupo.it (https://orcid.org/0000-0003-2287-3331)

Prof. Tracey Pirali, email: tracey.pirali@uniupo.it (https://orcid.org/0000-0003-3936-4787)

Table of Contents

1. **LC-HRMS analysis**……………………………………………………………………S3
2. **CYP inhibition**………………………………………………………………………...S23
3. **IDO1 protein levels by western blot**…………………………………………………S24
4. **TDO protein levels by western blot**…………………………………………………..S24
5. **Quantitative targeted-metabolomics in A375, U87 and HepG2 cell lines**…………S25
6. ***Apo*-IDO1 and *apo*-TDO inhibition**………………………………………………..S26
7. **FPocket-based shape analysis of *apo*-IDO1 and *apo*TDO**…………………………S32
8. **Interference with murine IDO1 signaling**……………………………………………S33
9. **Purity evaluation of selected compounds by HPLC-UV analysis**………………….S34
10. **^{1}H-NMR and ^{13}C-NMR spectra of selected compounds**…..…………...……………S41

LC-HRMS analysis

Instrumentation:	Hybrid quadrupole-orbitrap, Thermo Scientific Q-exactive *Plus*, equipped with a Vanquish UHPLC system.
Column:	Synergi Polar (150 × 2 mm, 4 µm d_p) (Phenomenex).
Mobile Phase	Phase A: 0.1% formic acid in water UHPLC grade.<br>Phase B: 0.1% formic acid in acetonitrile UHPLC grade.
Analysis mode:	Gradient of concentration.

Time (min)	% B
0.0	20
15.5	75
15.7	95
17.0	95
17.5	20
22.0	20

Flow rate:	0.250 mL/min
Column temperature:	40 °C
Sample temperature:	15 °C
Injected volume:	5 µL

The operating conditions of the HESI were as follows:

Parameter	Value
sheath gas flow rate (N_2)	45 Auxiliary Units
auxiliary gas flow rate	10 Auxiliary Units
sweep gas flow rate	1 Auxiliary Units
spray voltage	3.50 kV
capillary temperature	300 °C
auxiliary gas heater temperature	350 °C

The detector acquisition parameters were the following:

**+ Full scan**	**Value**
Microscan	1
Resolution	70,000
AGC target	1e6

+PRM (MS^2)	Value
Microscan	1
Resolution	35,000
AGC target	1e5
Maximum IT	120 ms
Loop count	1
MSX count	1
Isolation wind.	1.5 *m/z*
Isolation offset	0.0 *m/z*
NCE	20, 40, 60

+ ddMS^2	Value
Microscan	1
Resolution	17,500
AGC target	1e5
Maximum IT	60 ms
Loop count	4
MSX count	1
Isolation wind.	4.0 *m/z*
Isolation offset	0.0 *m/z*
NCE	20, 40, 60

	Retention time (min)	Theoretical $[M+H]^+$	Measured $[M+H]^+$	Δ Da ppm
**VS-15**	11.40	494.21867	494.21820	0.95
**M1**	7.81	280.14444	280.14413	1.11
**M2**	3.58	296.13935	296.13892	1.45
**M3**	4.38	296.13935	296.13898	1.25
**M4**	4.97	296.13935	296.13889	1.55
**M5**	5.71	296.13935	296.13889	1.55
**M6**	6.11	296.13935	296.13895	1.35
**M7**	9.64	492.20302	492.20278	0.49
**M8**	6.51	799.28682	799.28595	1.09

Table S1. Phase I metabolites of compound VS-15 formed upon incubation with MLM and detected by LC-HRMS analysis.

A. Compound VS-15

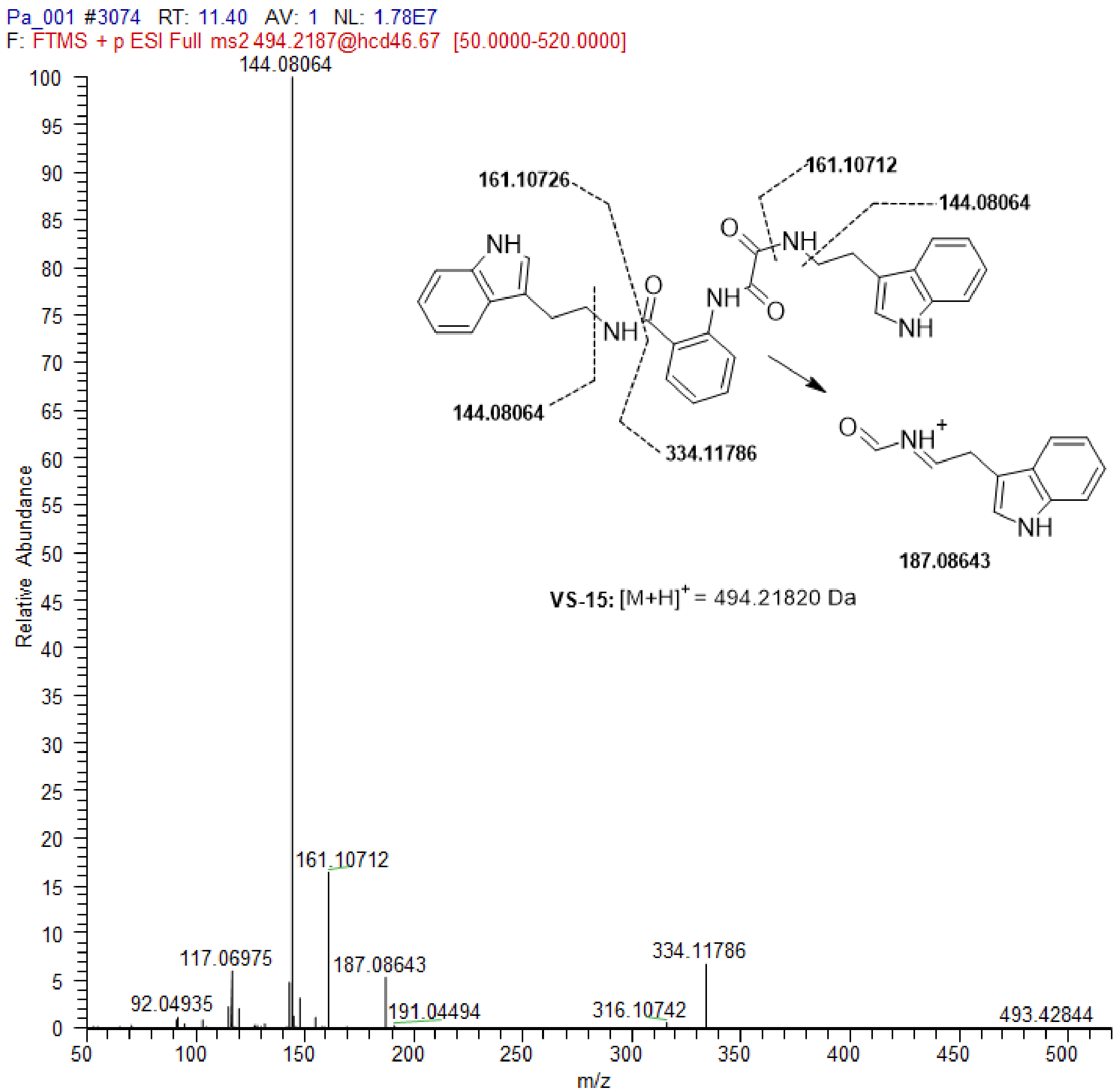

B. Metabolite **M1**

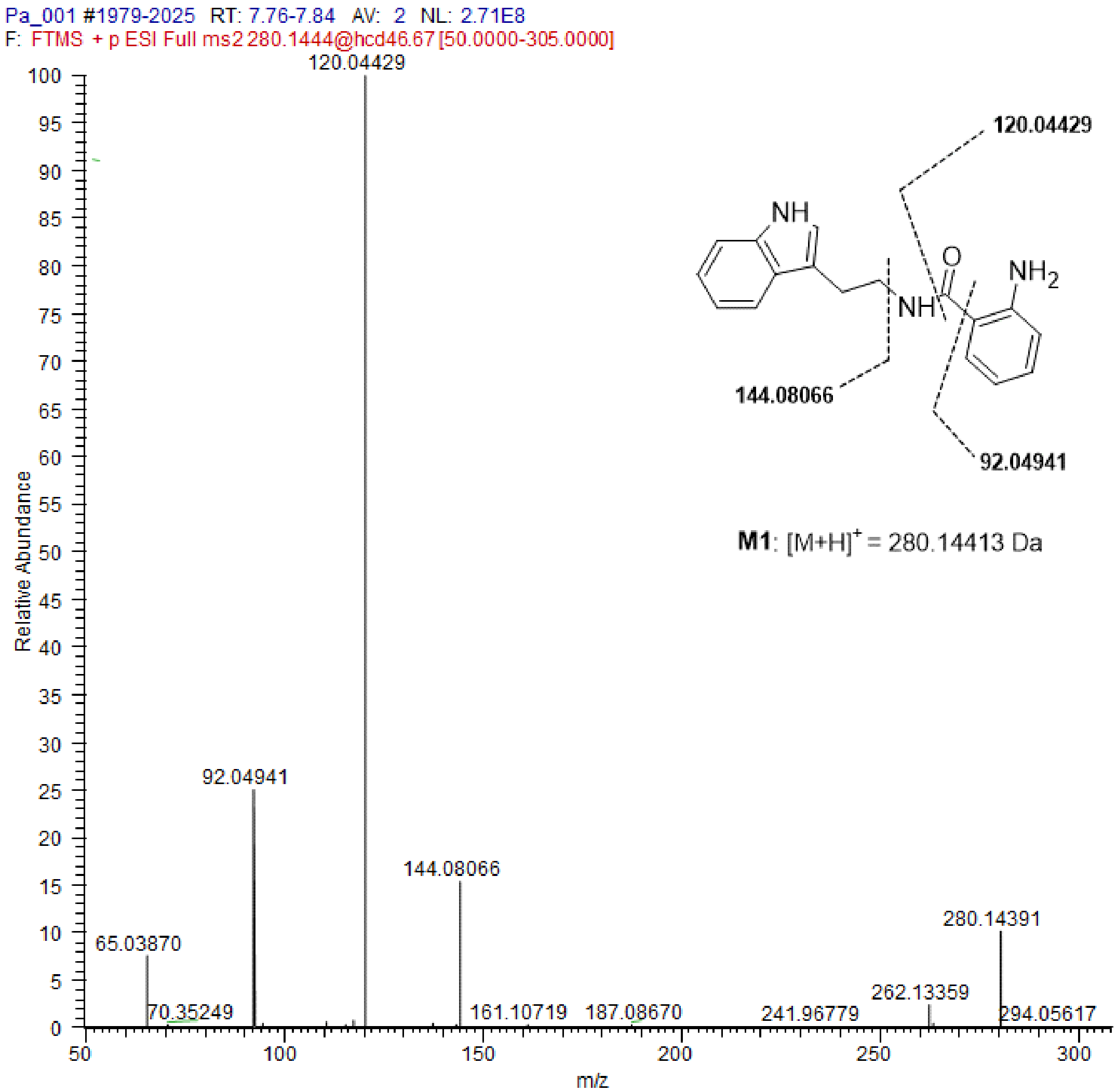

C. Metabolite **M2**

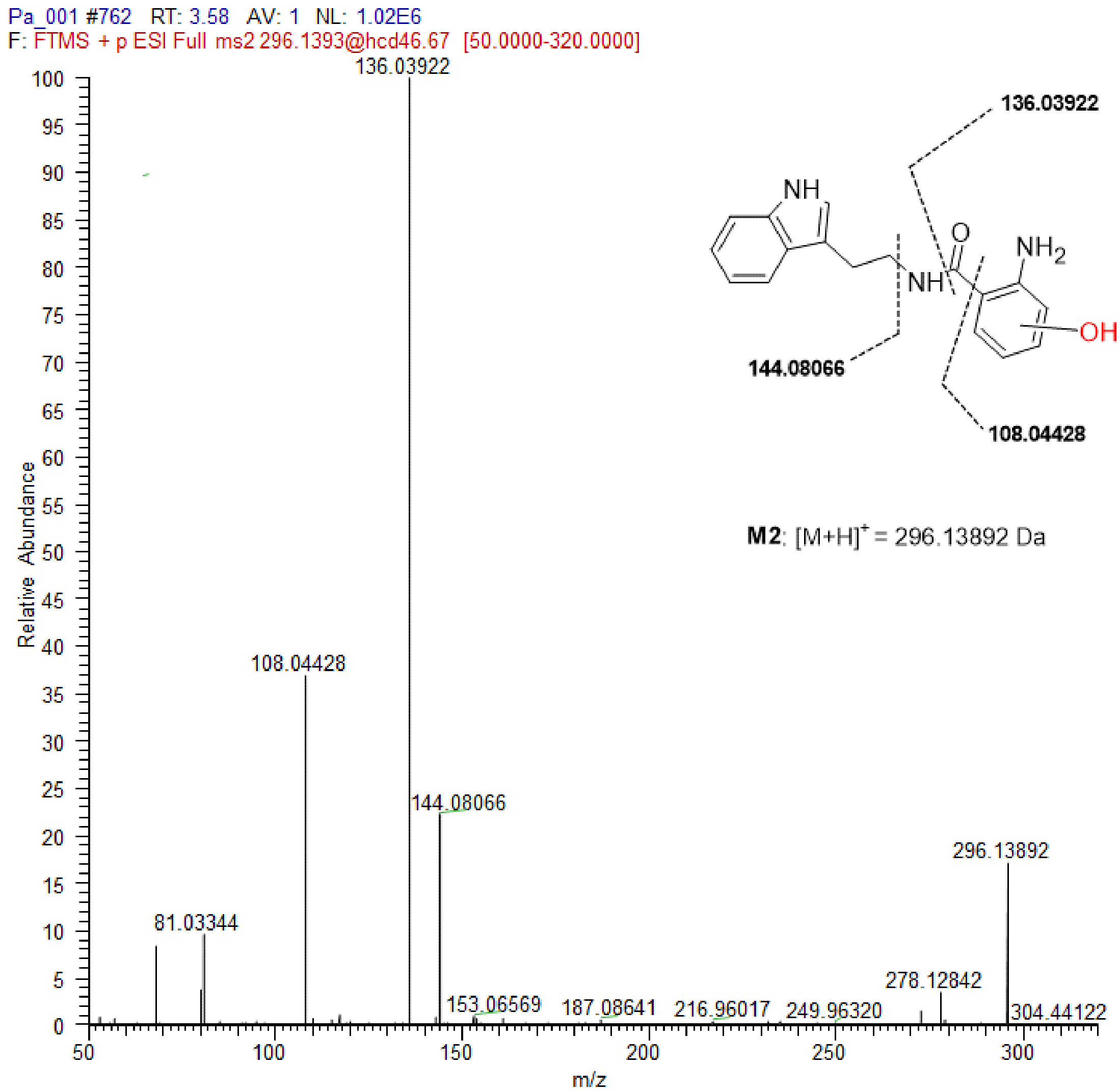

D. Metabolite **M3**

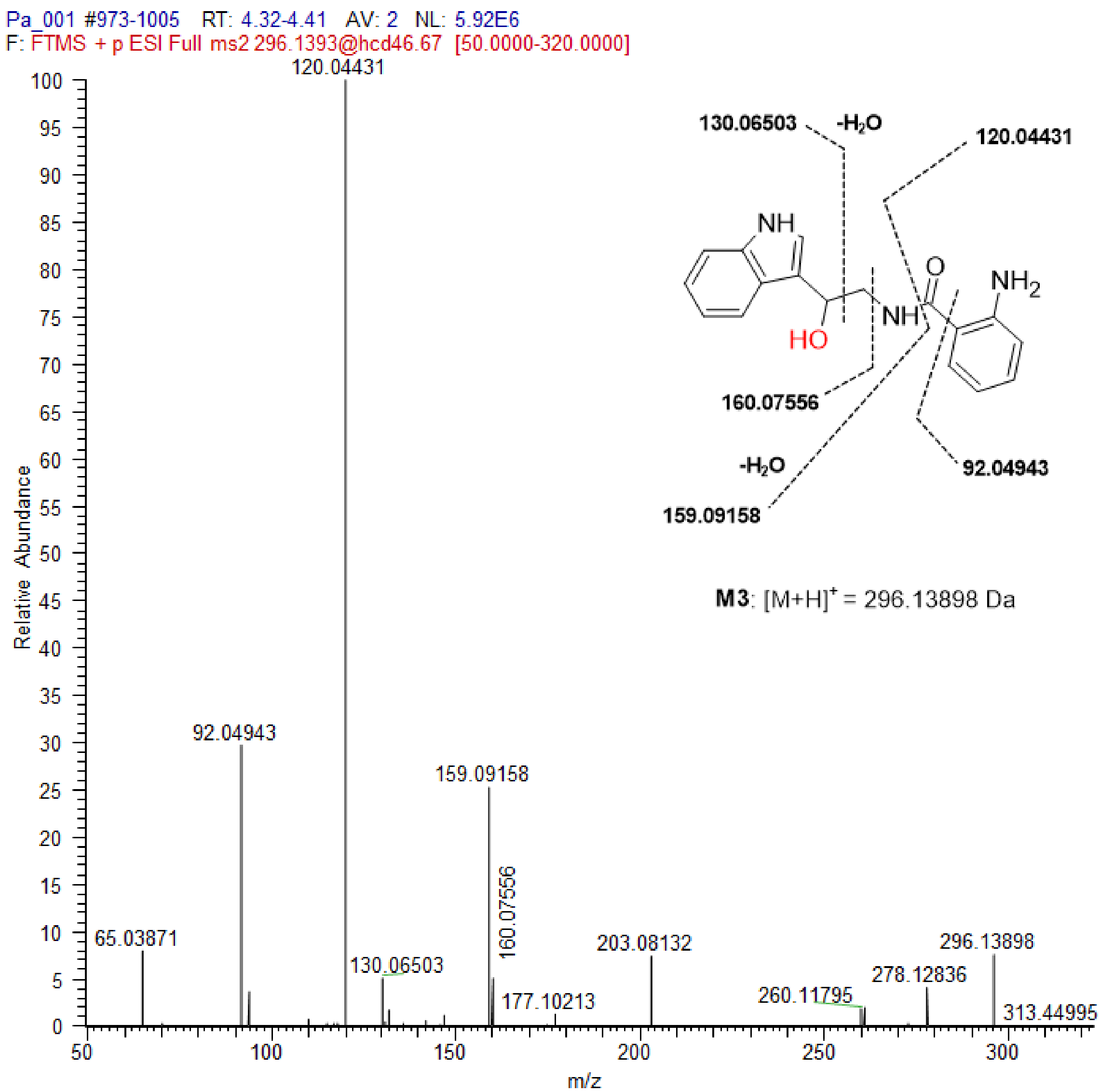

E. Metabolite **M4**

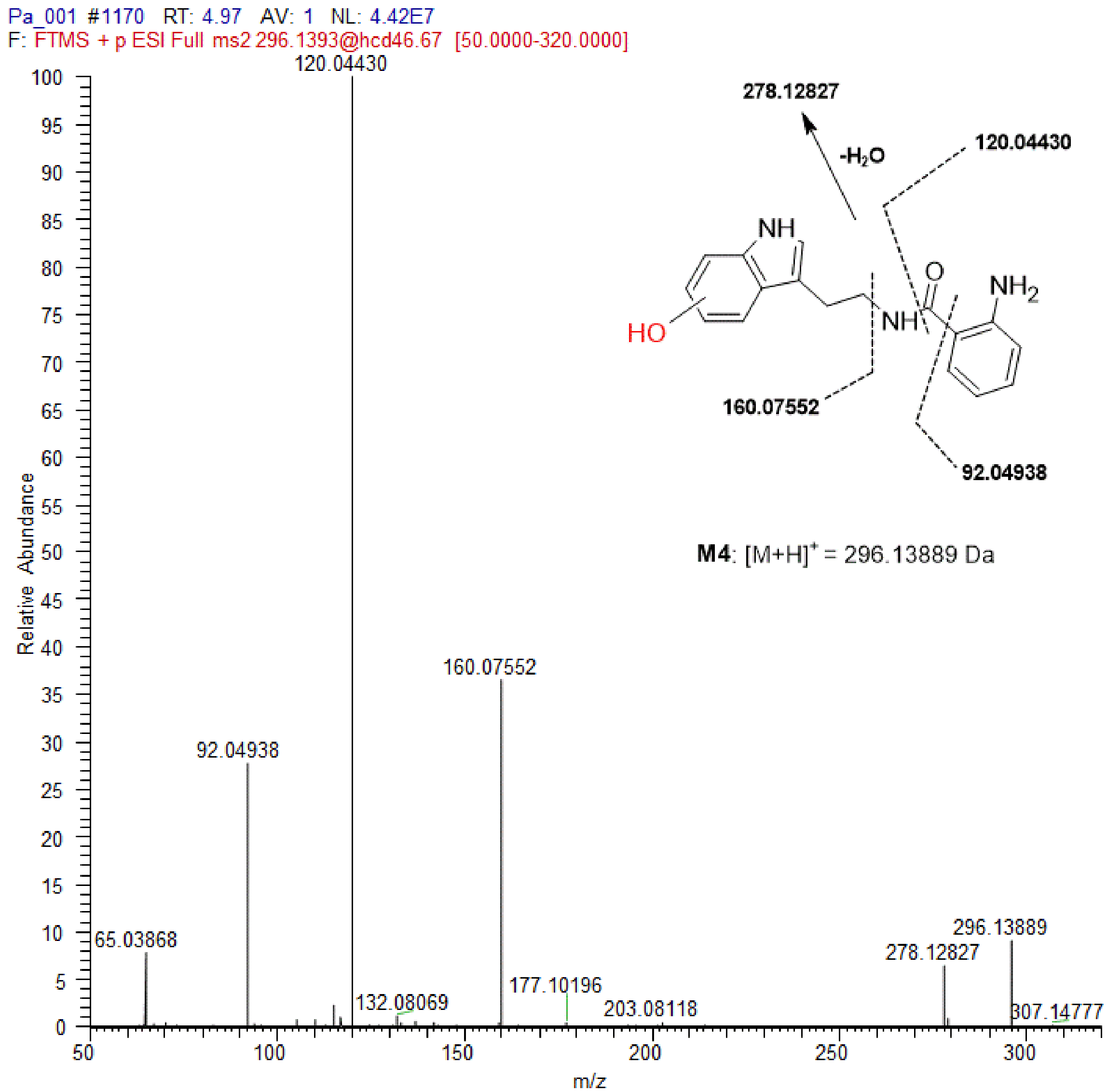

F. Metabolite **M5**

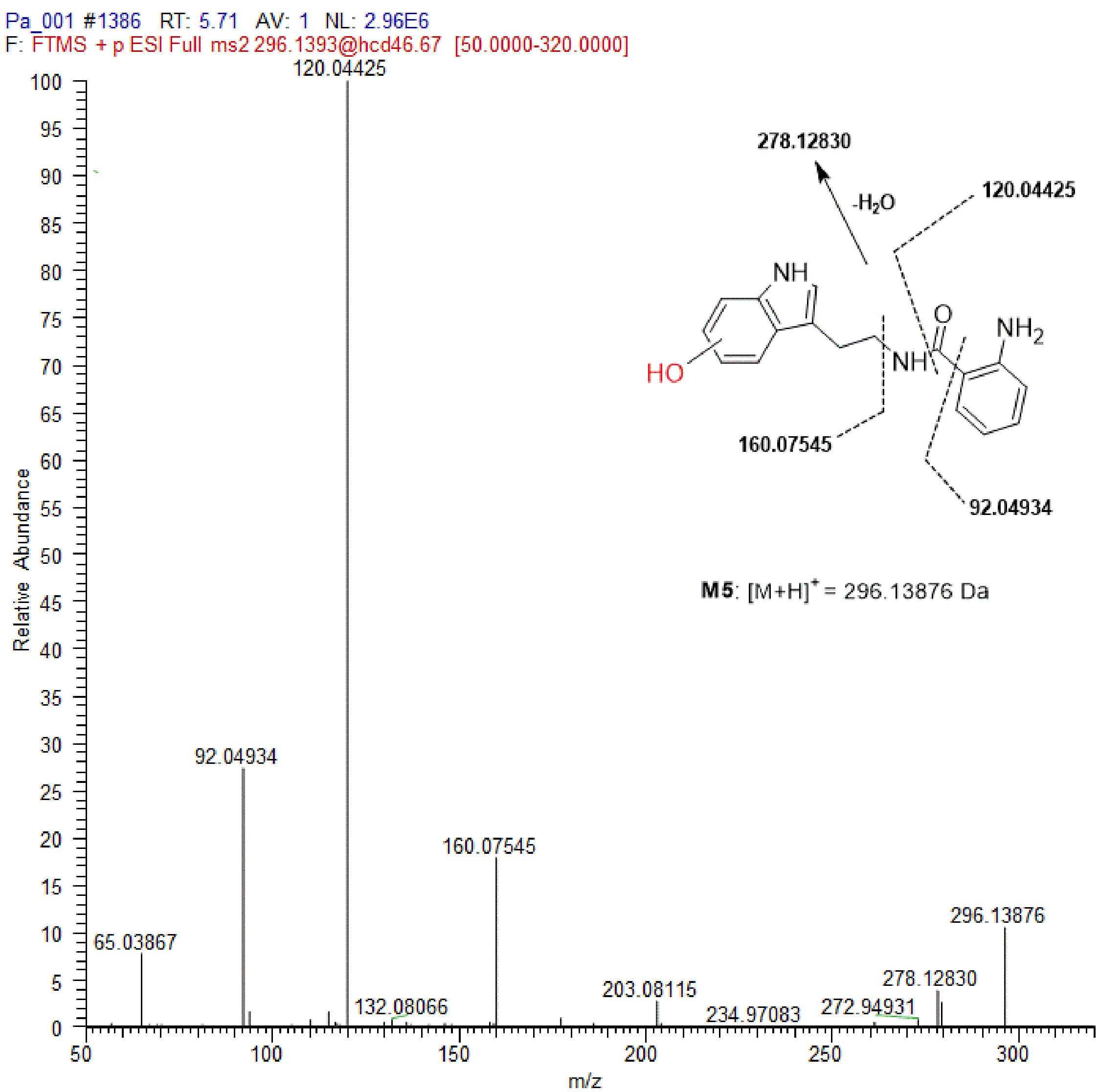

G. Metabolite **M6**

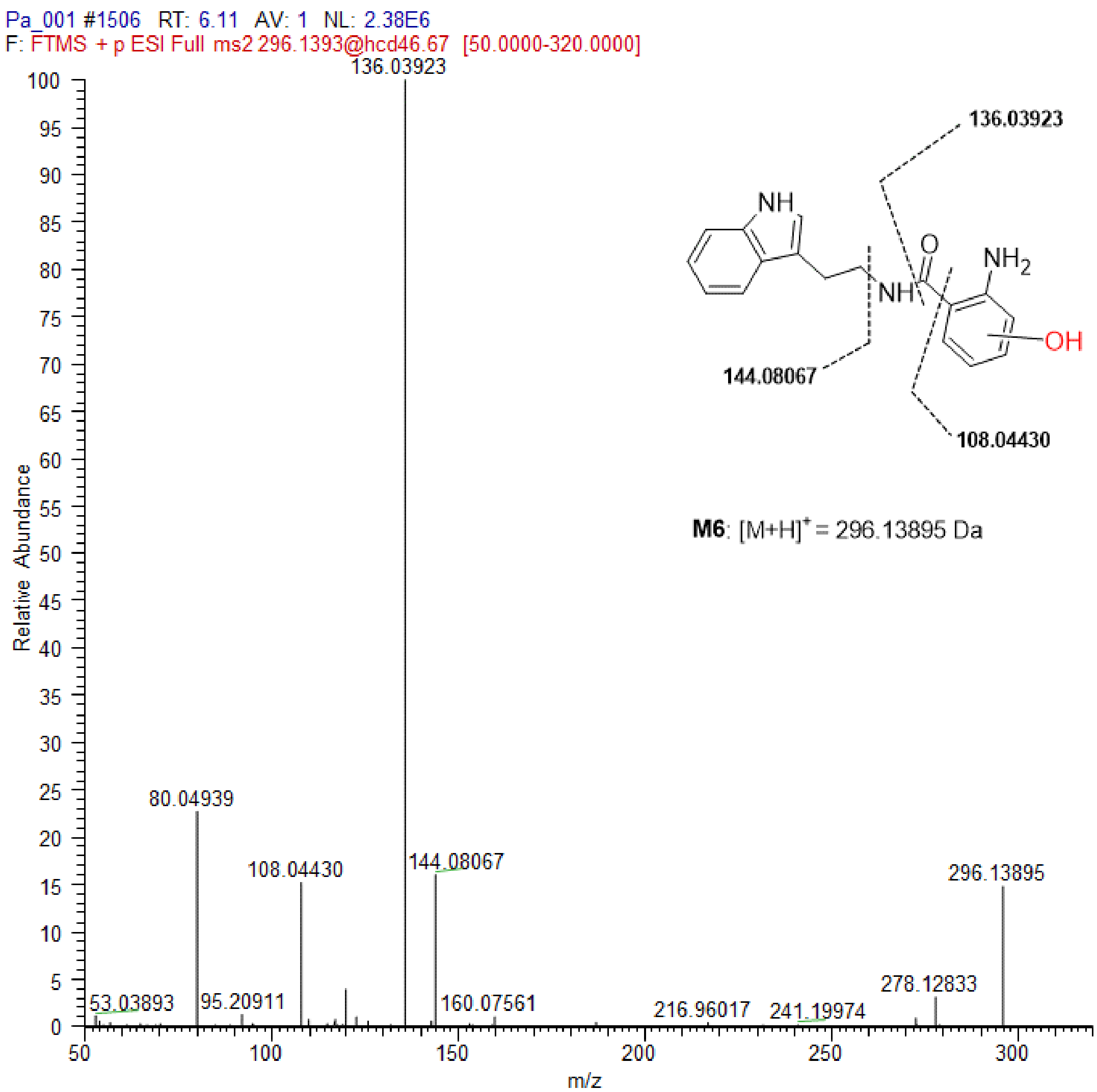

H. Metabolite **M7**

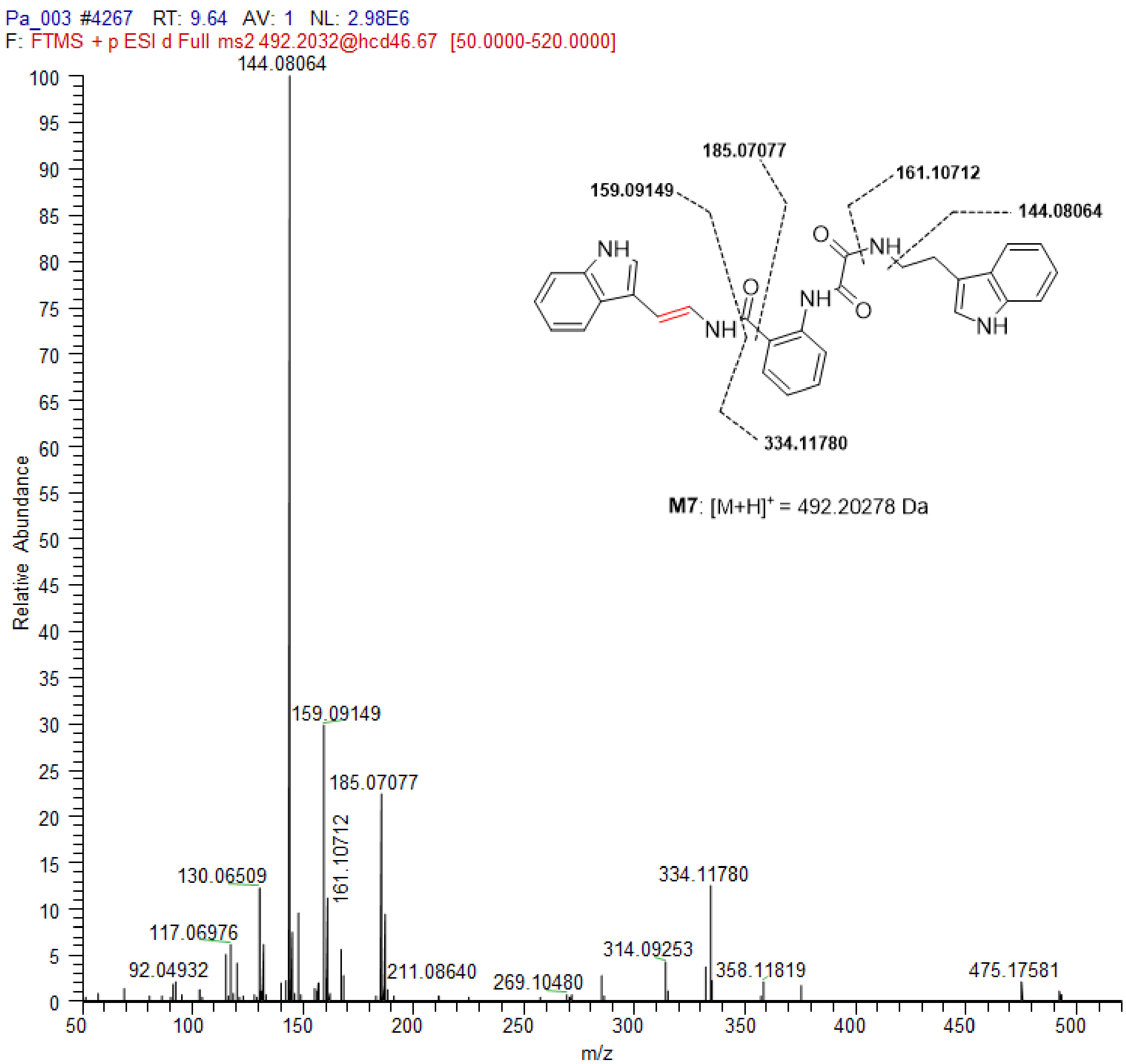

I. Metabolite **M8**

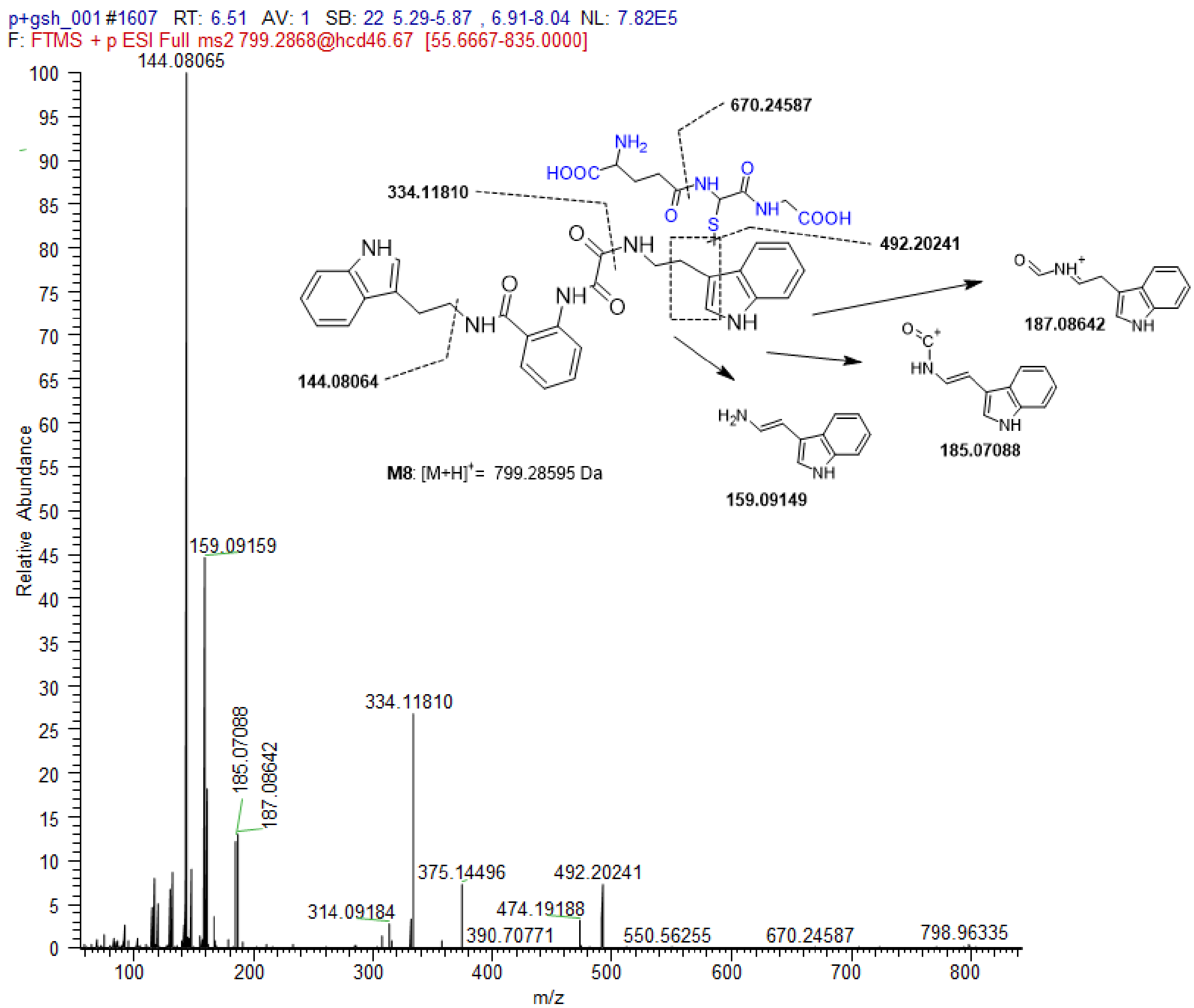

Figure S1. Compound VS-15 incubation with MLM: proposed structures, mass spectra (MS^2) data, and fragment ions interpretation of the detected metabolites **M1-M8**.

	**Retention time (min)**	**Theoretical $[M+H]^+$**	**Measured $[M+H]^+$**	**Δ Da ppm**
**66**	13.71	466.22375	466.22350	0.54
**M1**	10.73	482.21867	482.21942	1.56
**M2**	11.72	482.21867	482.21924	1.18
**M3**	12.08	482.21867	482.21884	0.35
**M4**	8.34	498.21358	498.21390	0.64
**M5**	9.57	498.21358	498.21393	0.70
**M6**	11.05	464.20810	464.20840	0.65

Table S2. Phase I metabolites of **66** formed upon incubation with MLM and detected by LC-HRMS analysis.

A. Compound **66**

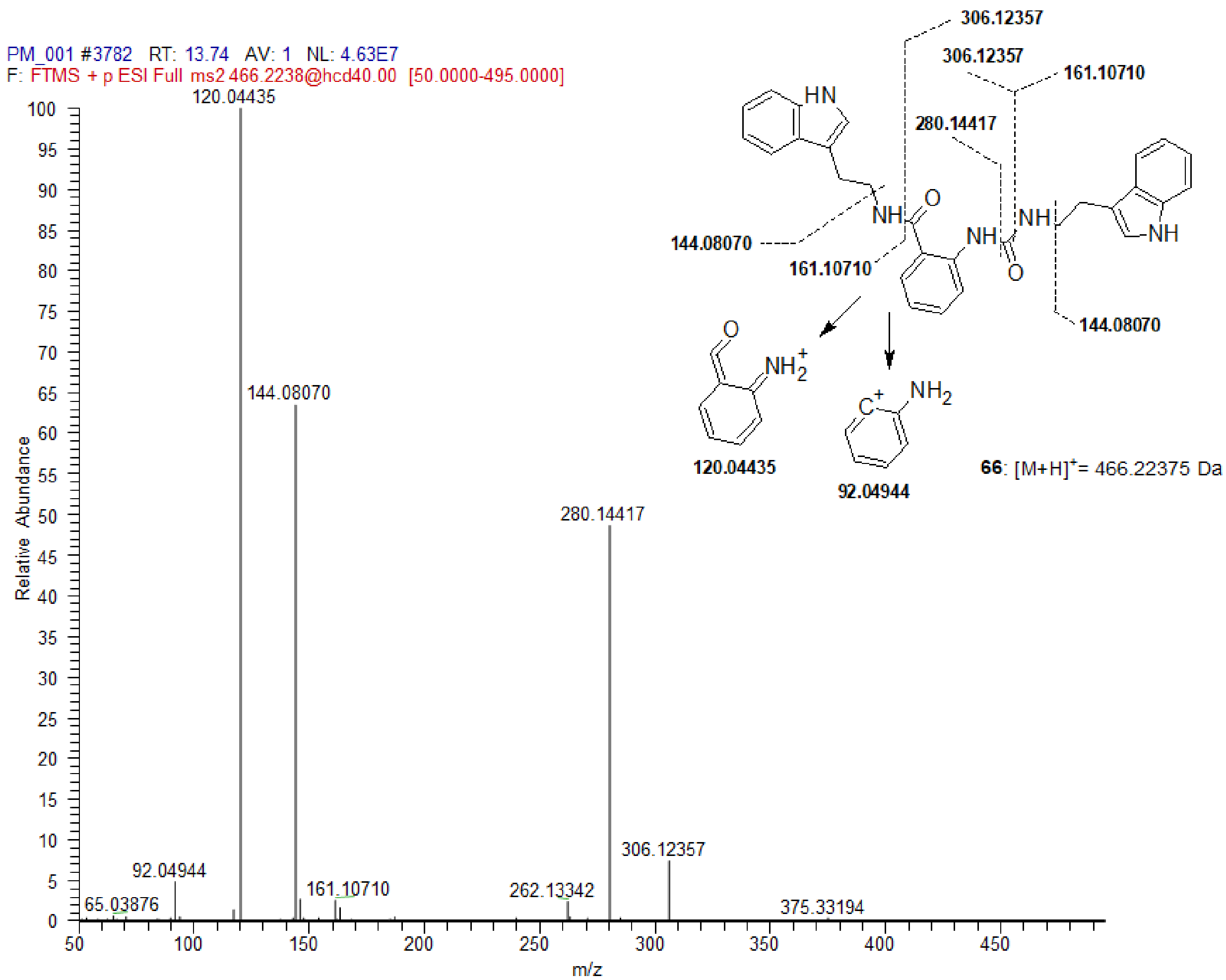

B. Metabolite **M1**

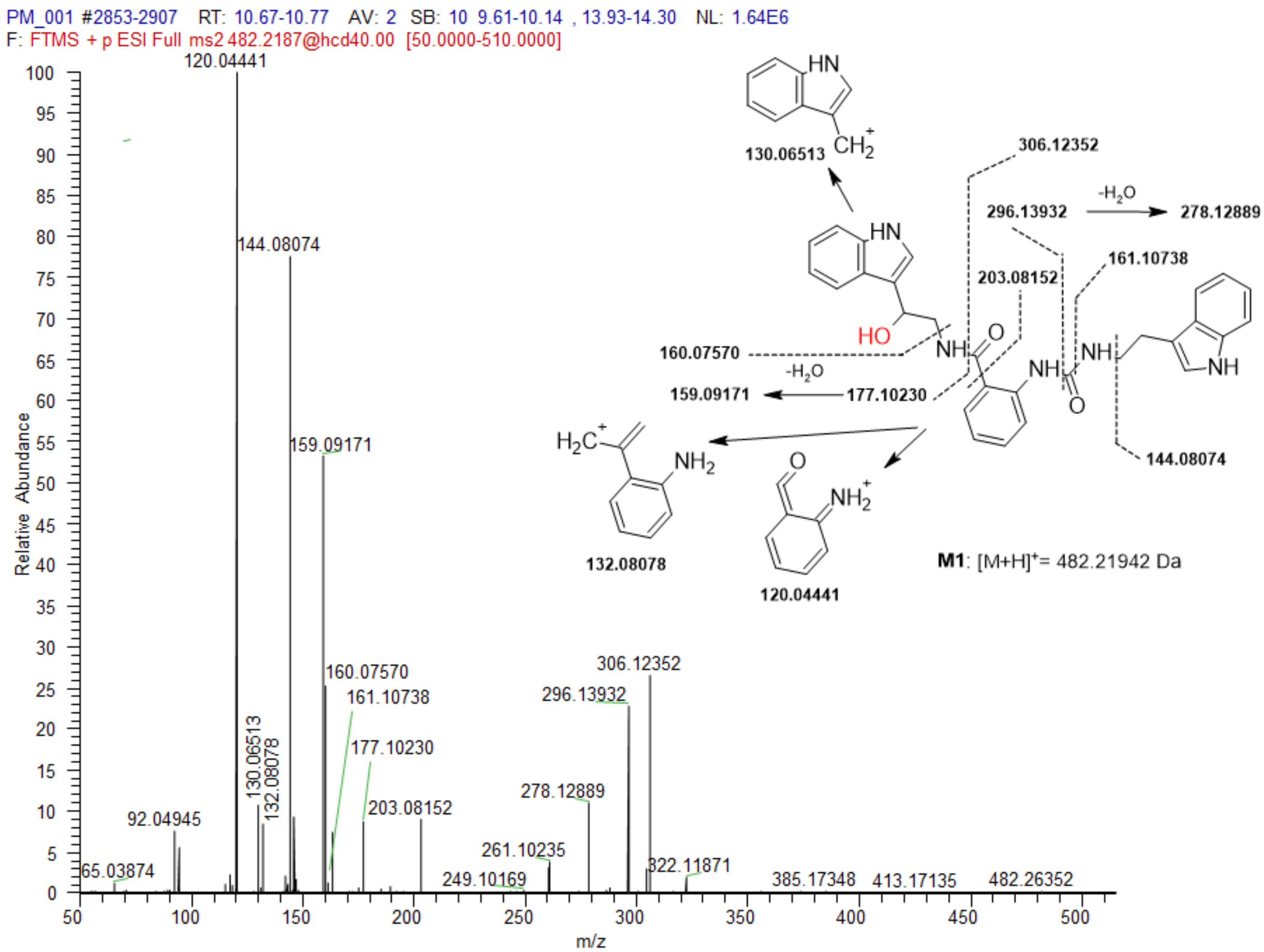

C. Metabolite M2

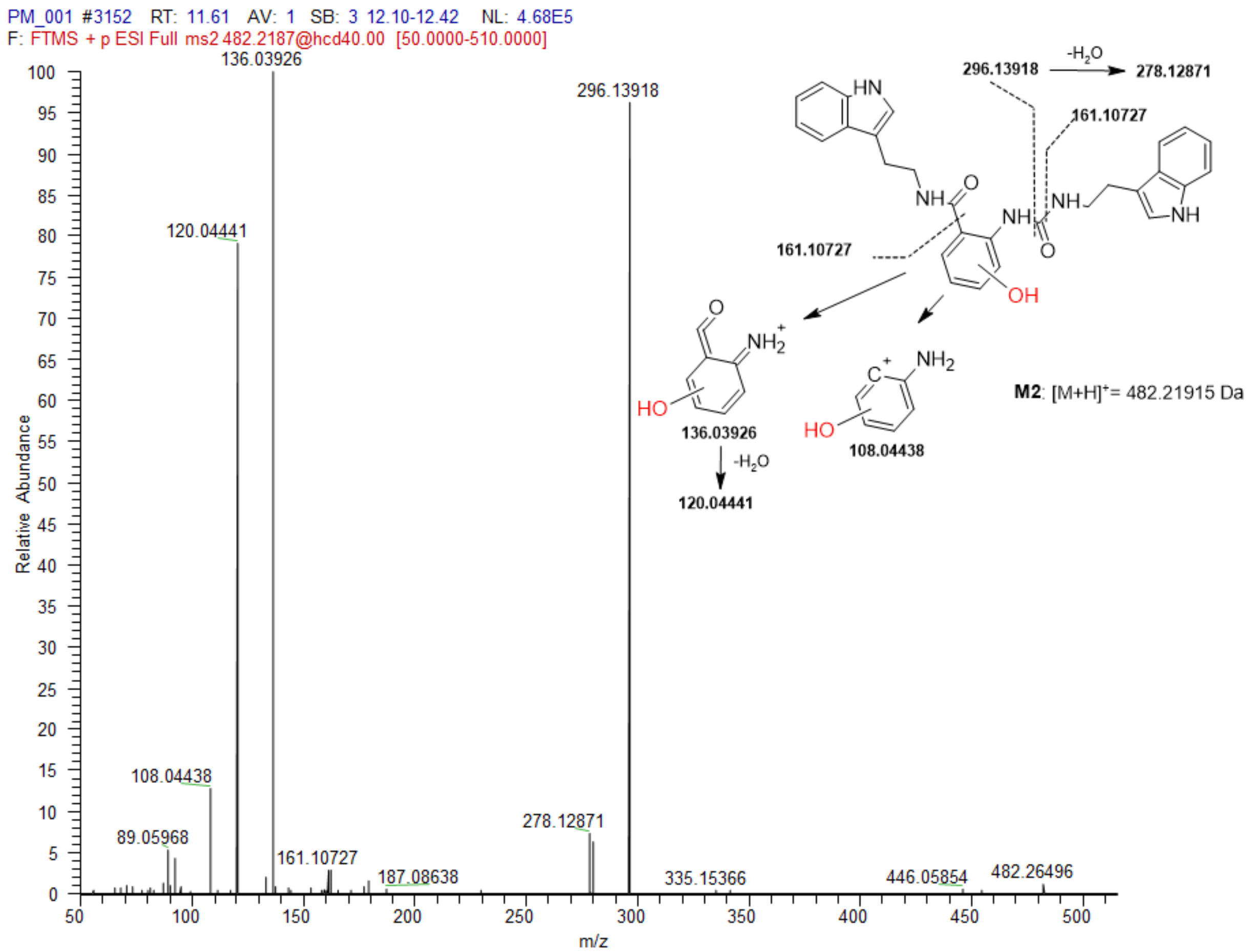

D. Metabolite **M3**

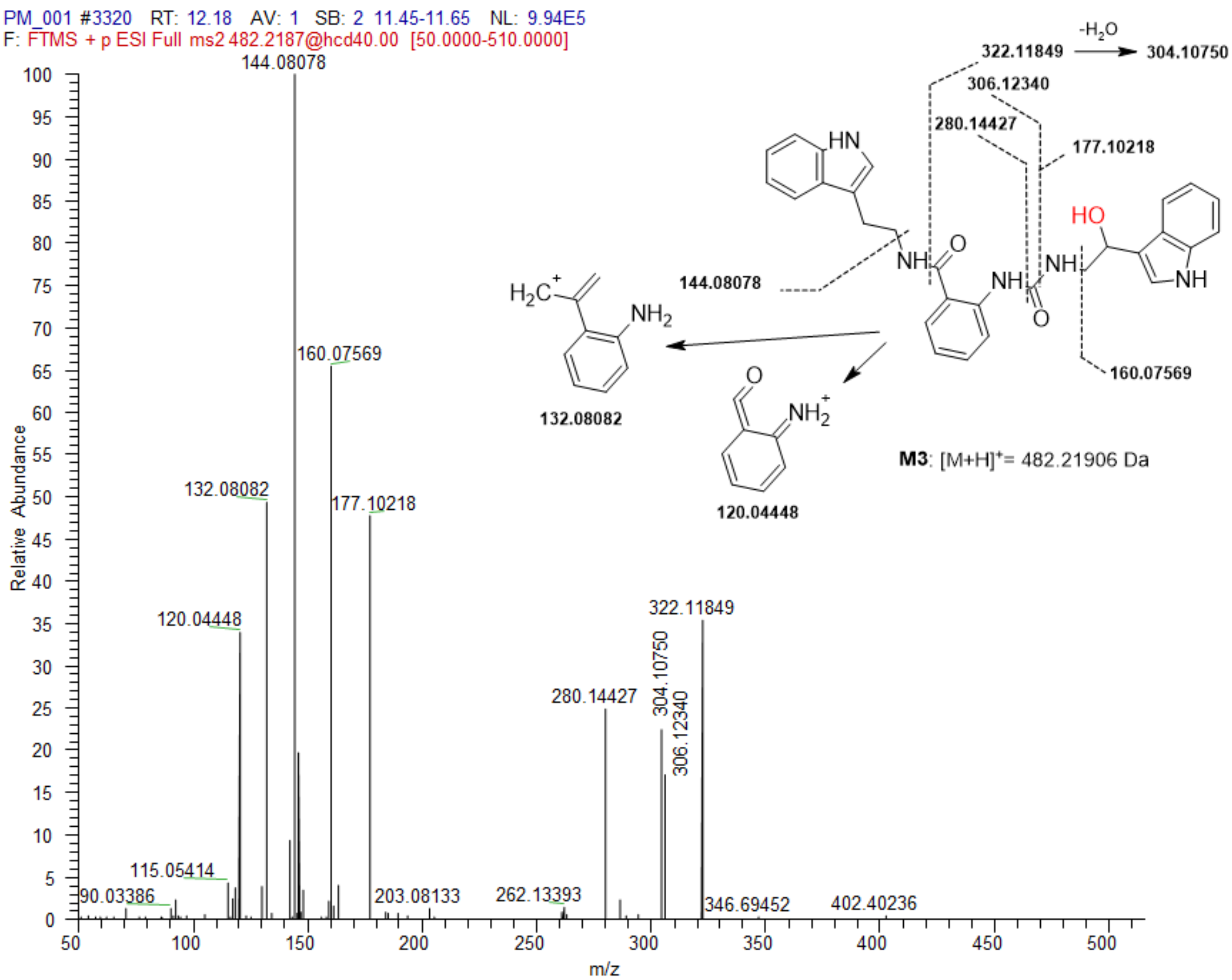

E. Metabolite **M4**

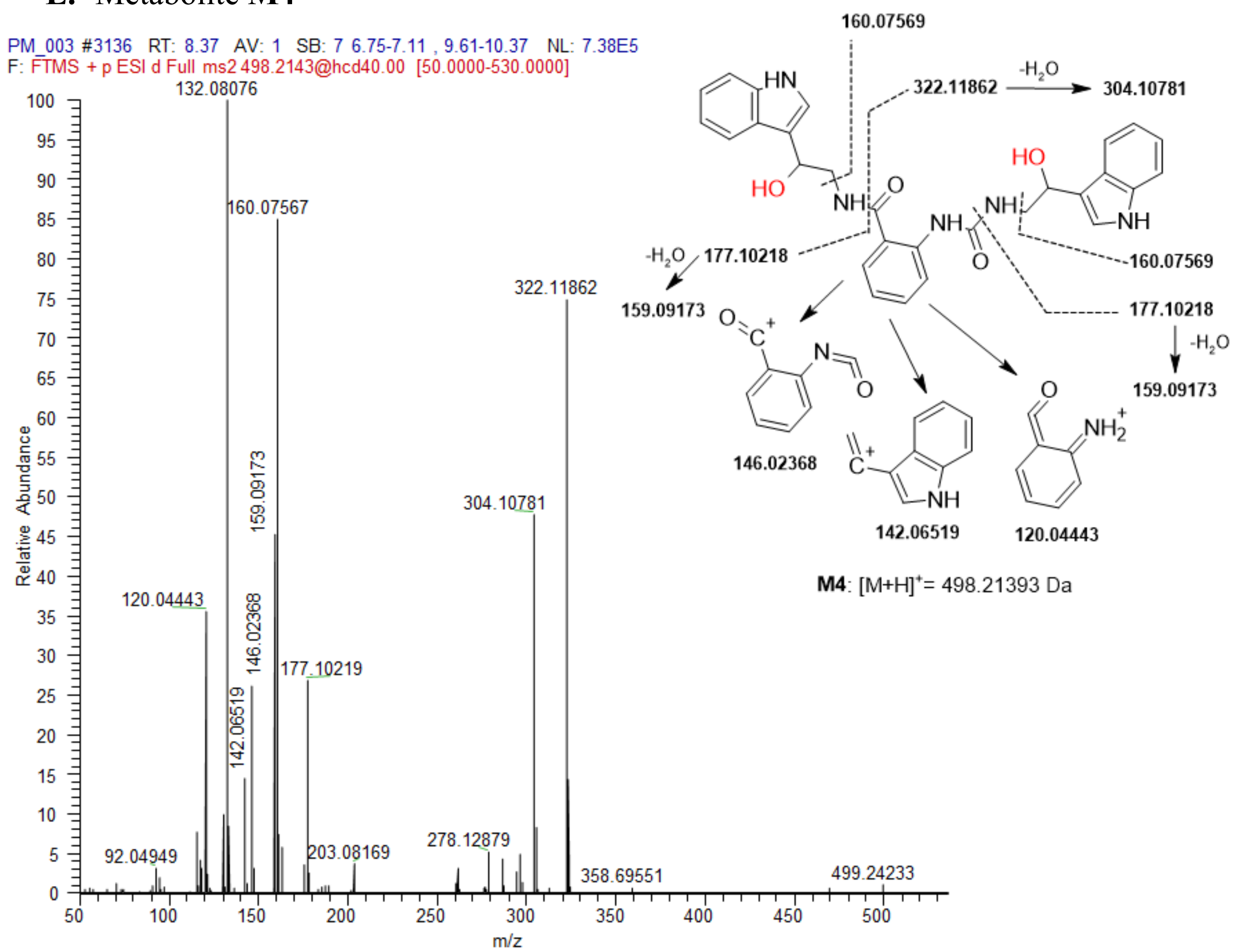

F. Metabolite **M5**

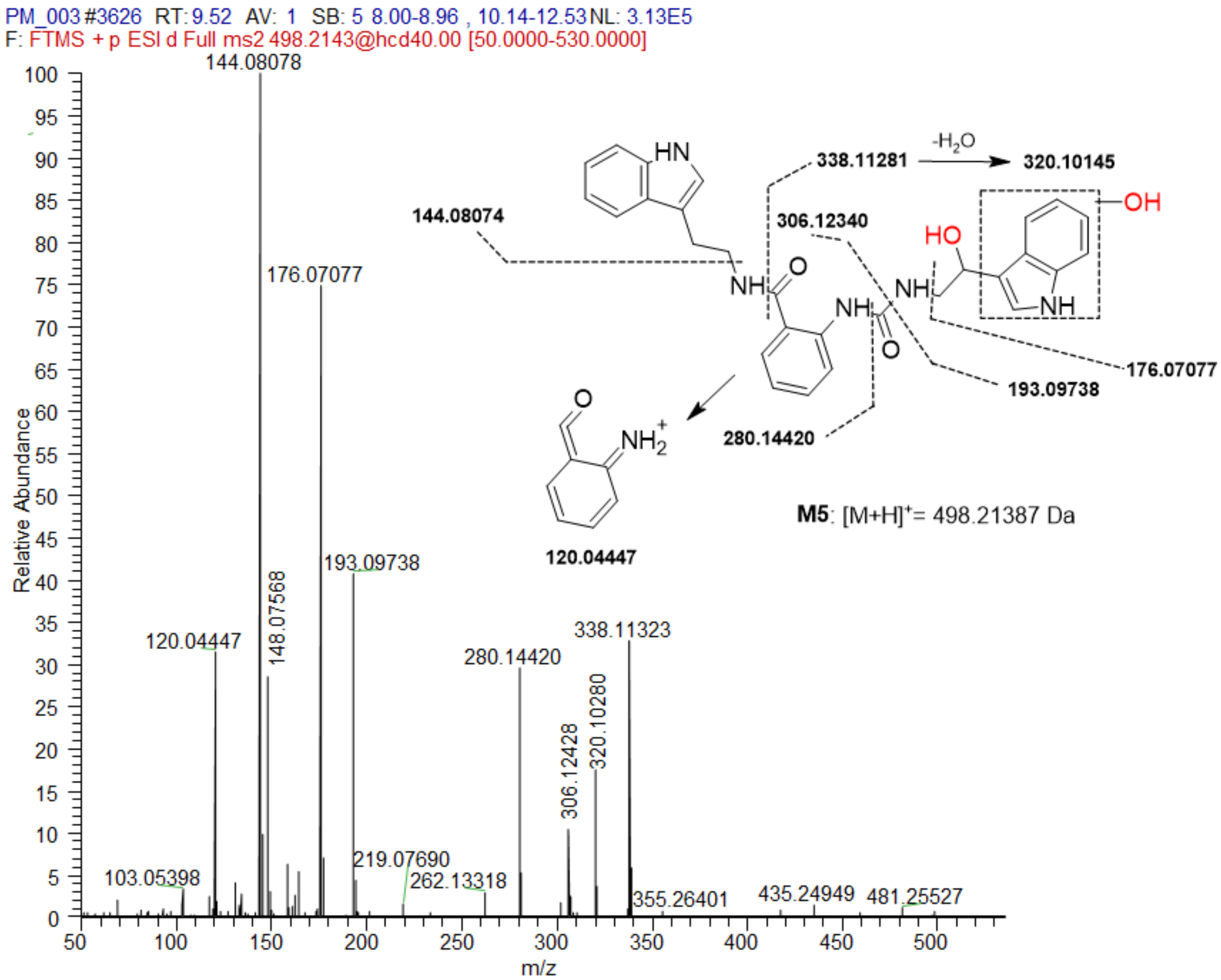

G. Metabolite **M6**

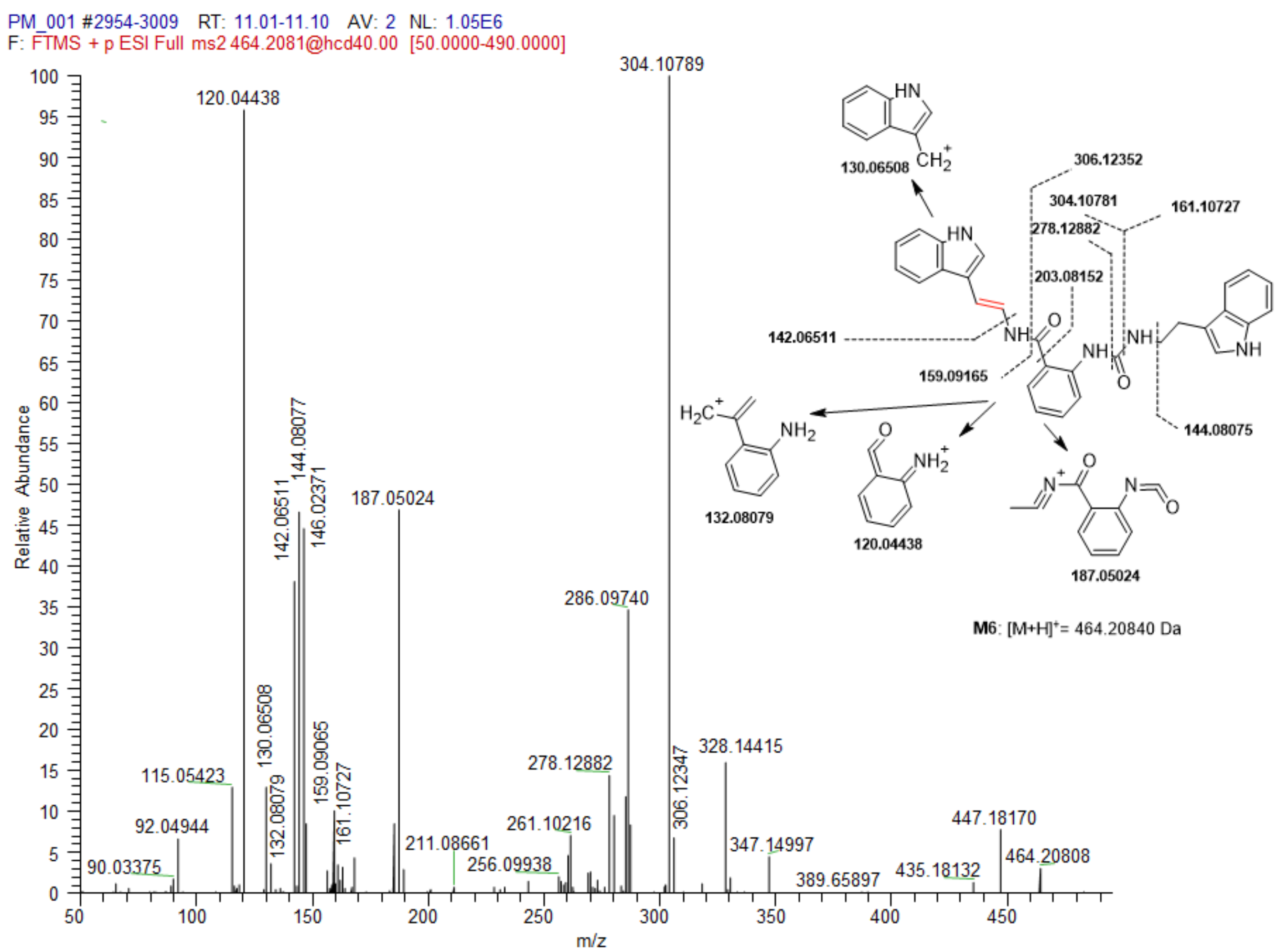

Figure S2. Compound **66** incubation with MLM: proposed structures, mass spectra (MS^2) data, and fragment ions interpretation of the detected metabolites **M1-M6**.

CYP inhibition

The inhibitory activity of **66** was evaluated on liver CYP using the aminopyrine *N*-demethylase activity assay, determining the amount of formaldehyde produced according to the previously reported procedure with slight modifications[1].

Briefly, 1 mM aminopyrine was incubated in liver microsomes (2 mg/mL, corresponding to 144 or 64 pmol total CYP for RLM or HLM respectively) dissolved in Tris/HCl buffer (50 mM, pH 7.4) and supplemented with 150 mM KCI, 10 mM $MgCl_2$. Samples were preincubated in the presence of increasing concentrations of **66** (0.01, 1, 10, 100, 1000 μM), then activated by 1 mM β-NADPH. After protein precipitation (equal volume of TCA 5%) the protein free supernatant was treated with Nash reagent and incubated at 37°C for 40 min.

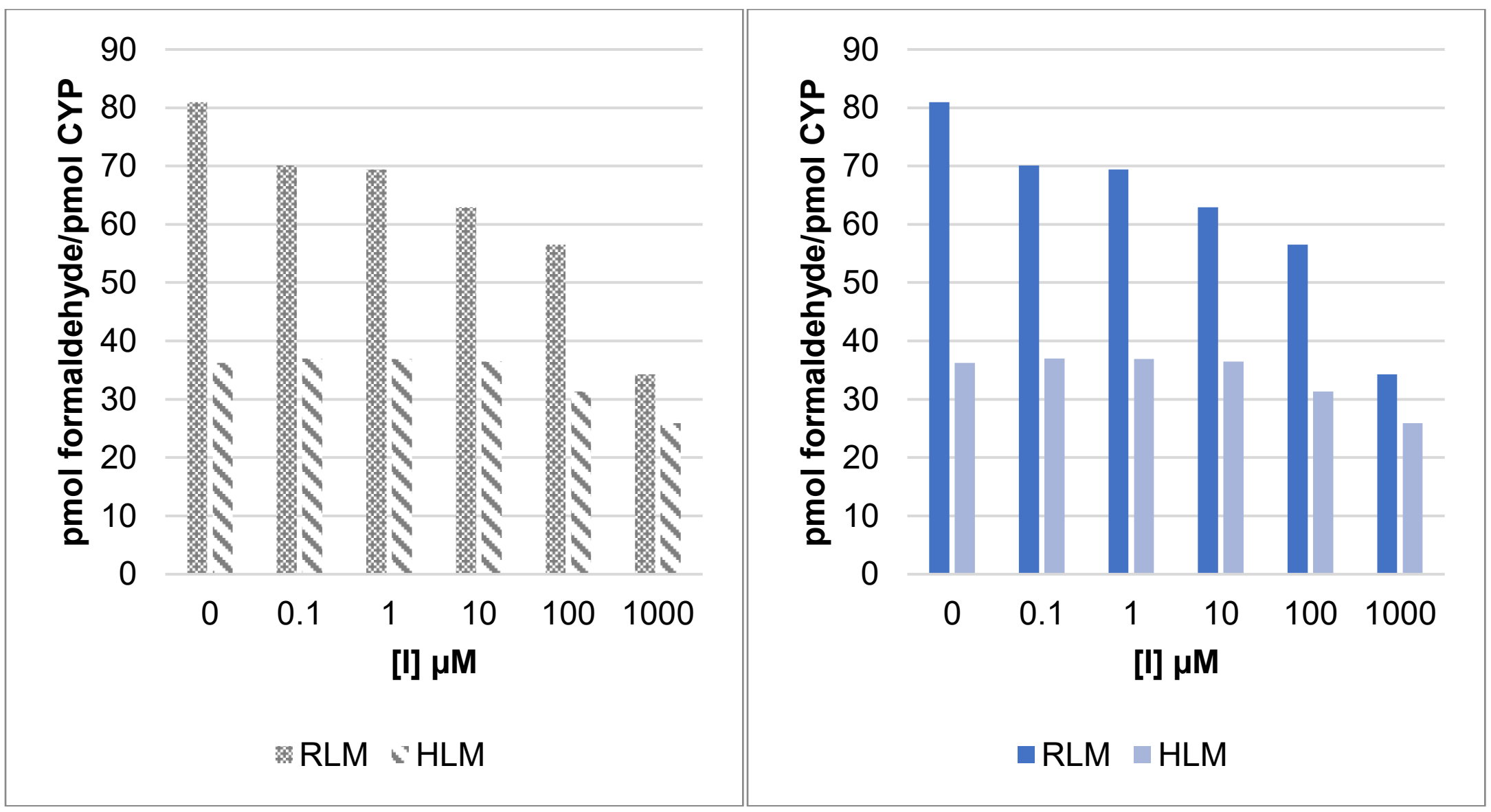

Figure S3. Inhibition of aminopyrine *N*-demethylase activity by compound **66** in RLM and HLM.

IDO1 protein levels by western blot

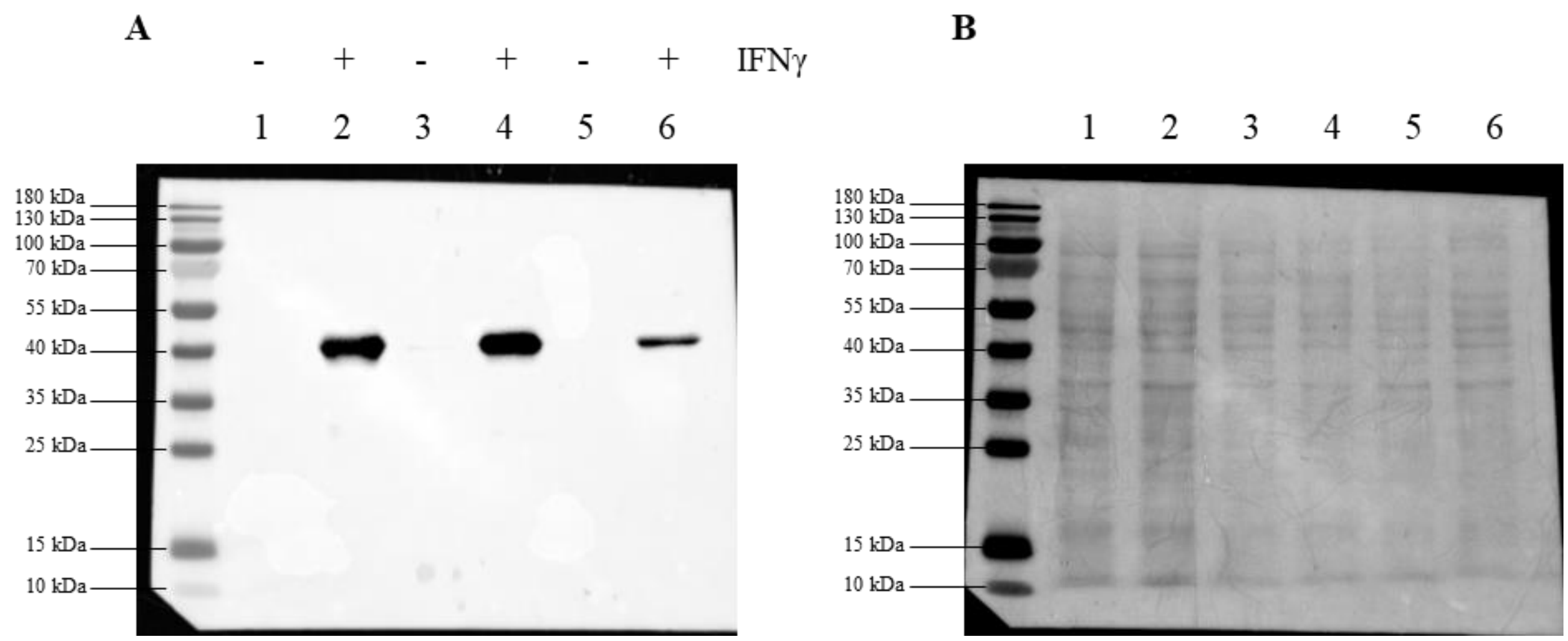

Figure S4. Western blot analysis of IDO1 expression following IFN-γ stimulation. (A) Immunoblot showing IDO1 protein levels after stimulation with IFN-γ (1000 U/mL) for 48h. Lane 1–2: A375 cells; lane 3–4: HepG2 cells; lane 5–6: U87 cells. For each cell line, the first lane represents untreated control cells and the second lane represents IFN-γ-treated cells. (B) Ponceau S staining of the membrane showing total protein loading as a loading control.

TDO protein levels by western blot

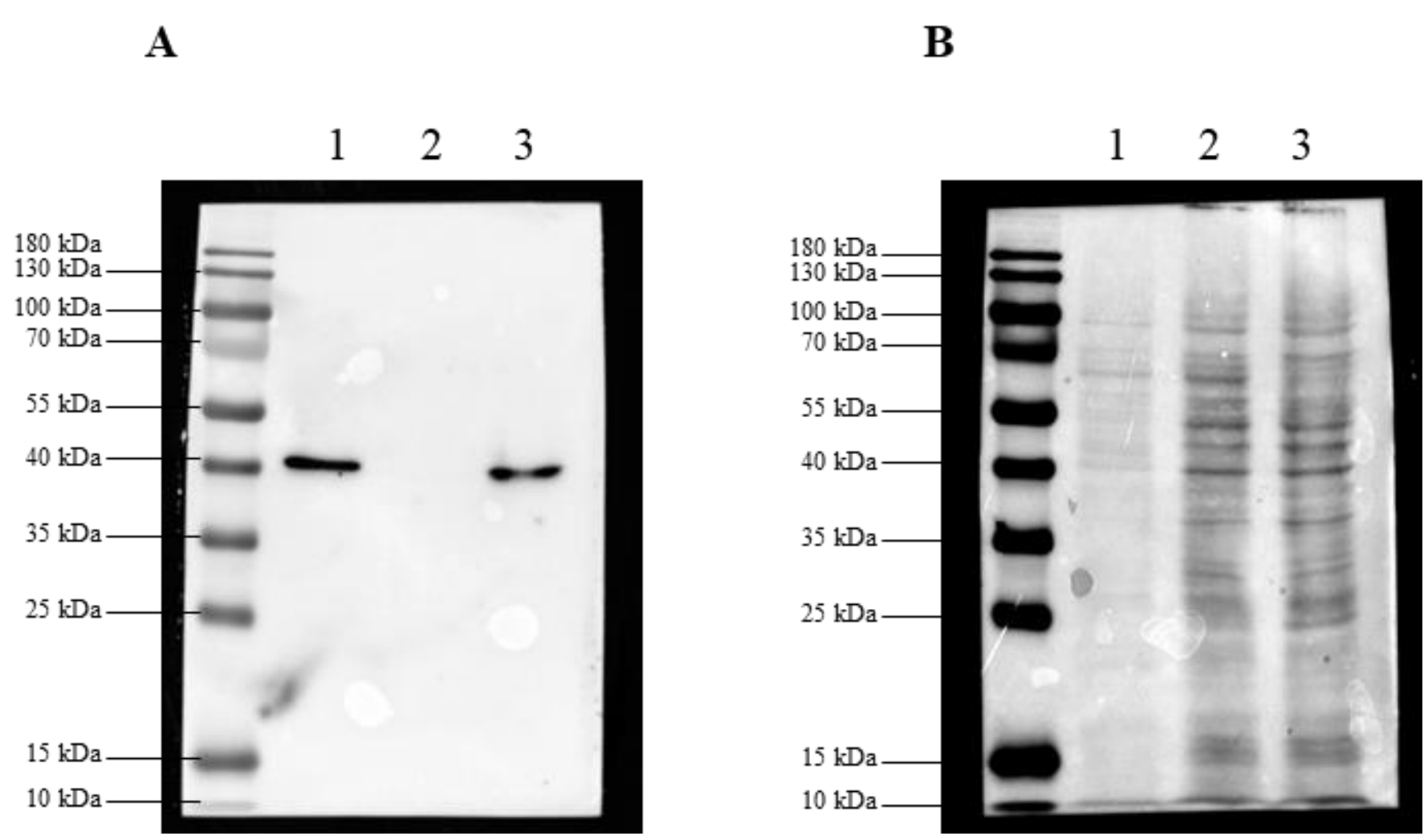

Figure S5. Western blot analysis of TDO expression. (A) Representative immunoblot showing TDO protein expression in SW-48 (lane 1), A375 (lane 2), and U87 (lane 3) cells. SW-48 and A375 cells were used as positive and negative controls for TDO expression, respectively. (B) Ponceau S staining of the membrane showing total protein loading as a loading control.

Quantitative targeted-metabolomics in A375, U87 and HepG2 cell lines

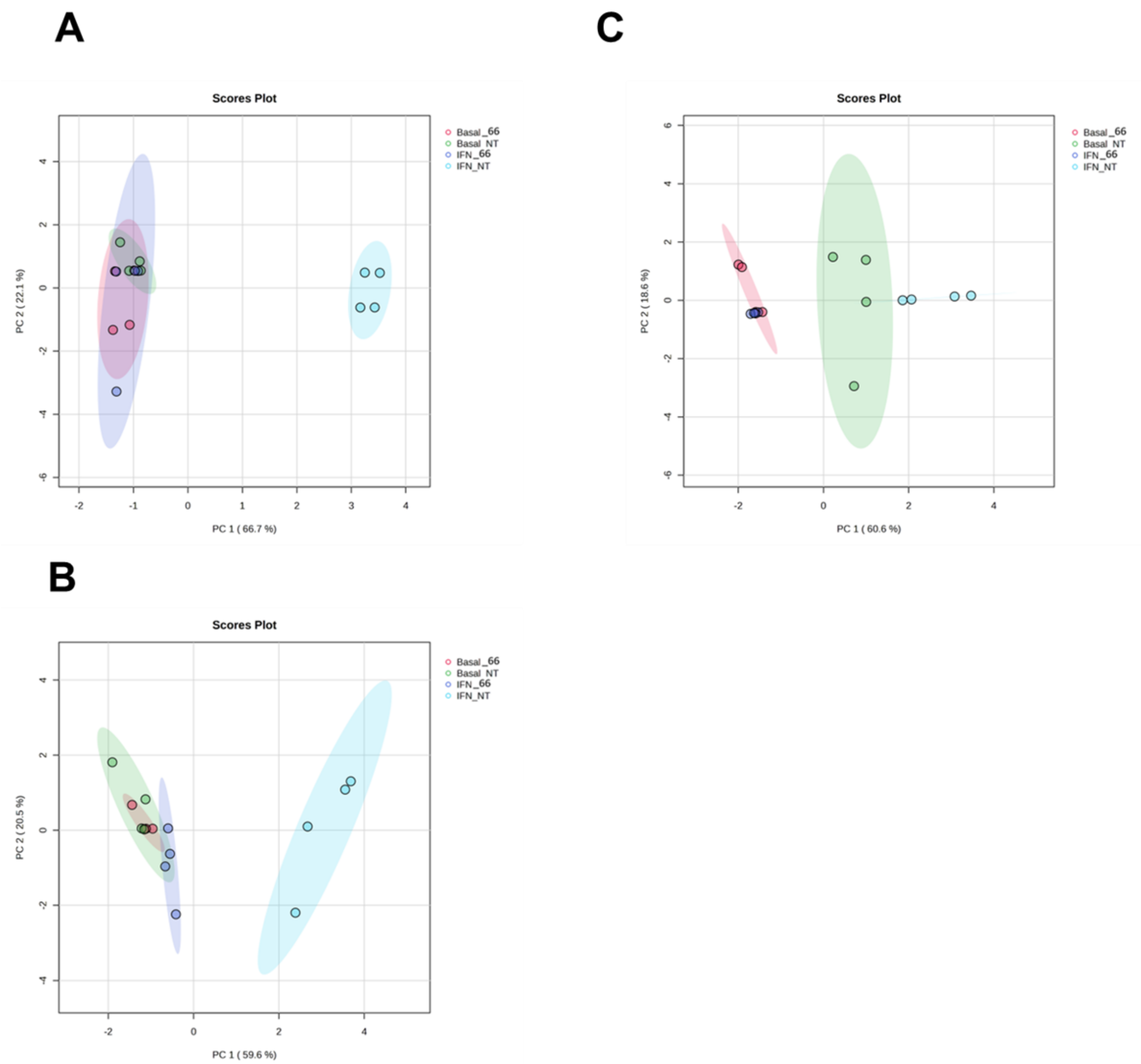

Figure S6. Principal Component Analysis (PCA) of A375 (A), HepG2 (B), and U87 (C) cell lines. In all three cell lines, IFNγ-stimulated samples are clearly distinguishable along PC1 (right side), indicating effective IDO1 induction and increased kynurenine production. In panel A (A375), the Basal, Basal_**66**, and IFN_**66** groups cluster together on the left side of PC1, suggesting efficient inhibition of IDO1 activity. In panel B (HepG2), the IFN_**66** group is located near the Basal condition, where kynurenine levels are minimal, again indicating effective IDO1/TDO inhibition. In panel C (U87), the overlap among Basal, Basal_**66**, and IFN_**66** groups is even more pronounced, confirming robust inhibition of IDO1/TDO. Legend: Basal_**66**: Uninduced cell line treated with compound **66**; Basal_NT: Uninduced, untreated cell line; IFN_**66**: IFNγ-induced cell line treated with compound **66**; IFN_NT: IFNγ-induced, untreated cell line.

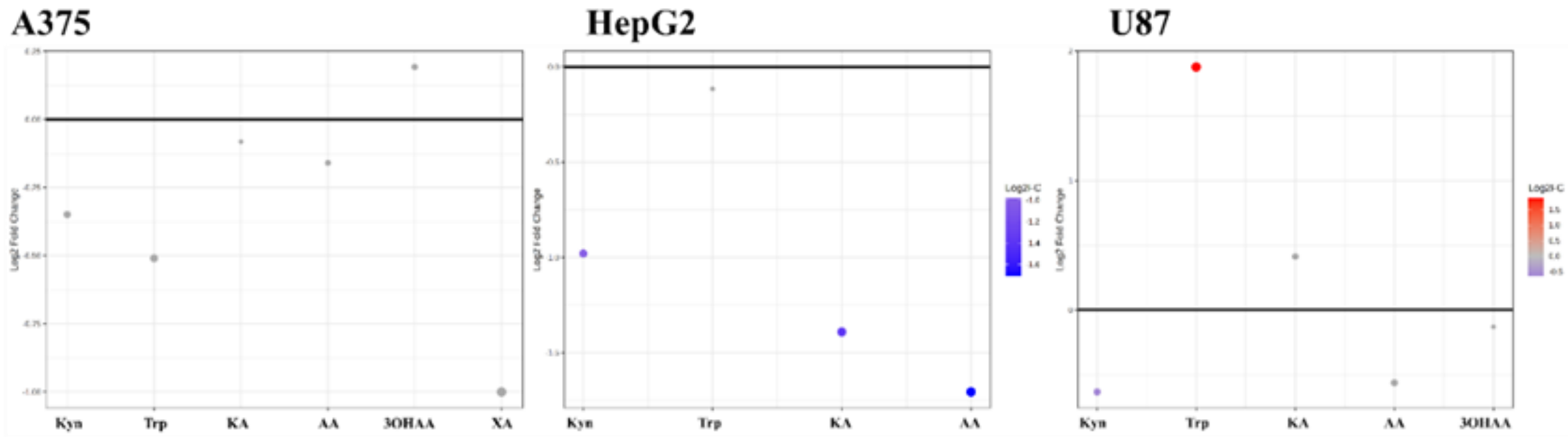

Figure S7. Fold Change (FC) Analysis. The goal of fold change analysis is to compare the absolute values of change between two group means before the application of column-wise data normalization. Significant features are those whose FCs exceed the given FC threshold (either up or down, in this case above or below 1.5). The comparisons were performed between inhibited basal cells and basal controls to highlight FC differences after the inhibitor's effect. The A375 cell line is not affected by **66** under basal conditions, as no FC differences were observed. In contrast, the HepG2 and U87 cell lines clearly show a reduction trend in kyn, which is attributable to TDO inhibition.

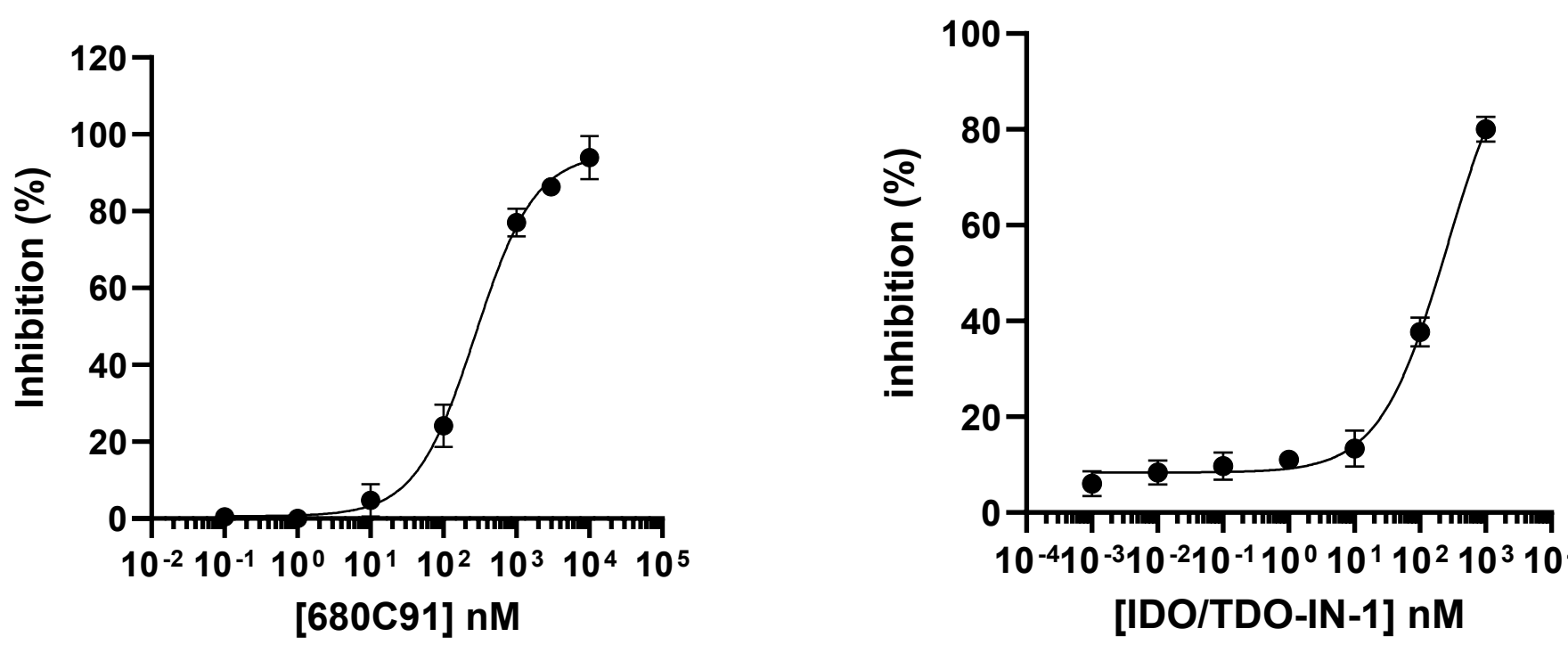

Figure S8. Concentration response curves of the TDO inhibitor 680C91 in U87 cells under basal conditions (left) and of the dual IDO1/TDO inhibitor IDO/TDO-IN-1 in IFNγ-stimulated U87 cells expressing IDO1 (right).

Apo-IDO1 and *apo*-TDO inhibition

Cell-based assay in P1.HTR

Cells of P1.HTR, a highly transfectable clonal variant of mouse mastocytoma P815[2, 3] were transfected by electroporation with a plasmid constructs coding for mouse (P1.mIDO1) or human (P1.hIDO1) IDO1, mouse (P1.mTDO) or human (P1.hTDO) TDO as described in previous work[3, 4]. The stable transfectant cell line was obtained by puromycin selection and cultured in Iscove's

Modified Dulbecco's Medium (Gibco, Invitrogen CA, USA) supplemented with 10% FCS (Gibco, Invitrogen CA, USA), 1 mM glutamine (Gibco, Invitrogen CA, USA) and penicillin/streptomycin (Gibco, Invitrogen CA, USA) at 37°C in a humidified 7% CO_2 incubator.

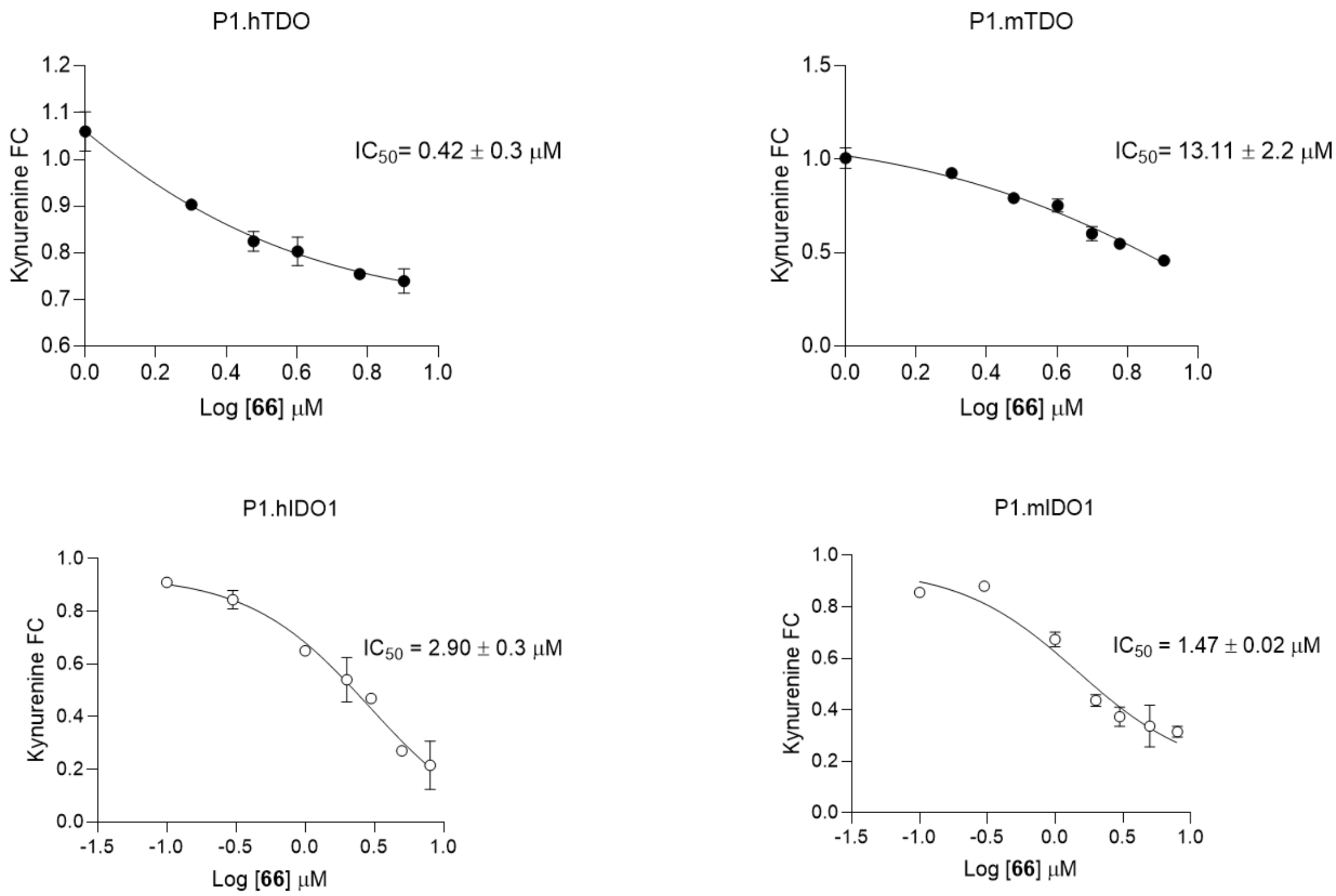

Figure S9. Inhibitory activity on TDO and IDO1. Cellular IC_{50} value of **66** obtained from a concentration-response curve in P1.HTR tumor cells stably transfected with human (left) or mouse (right) TDO or IDO1. Results are the mean ± S.D. of four independent experiments, each performed in triplicate.

Spectrophotometric characterization of *holo*-IDO1 and TDO

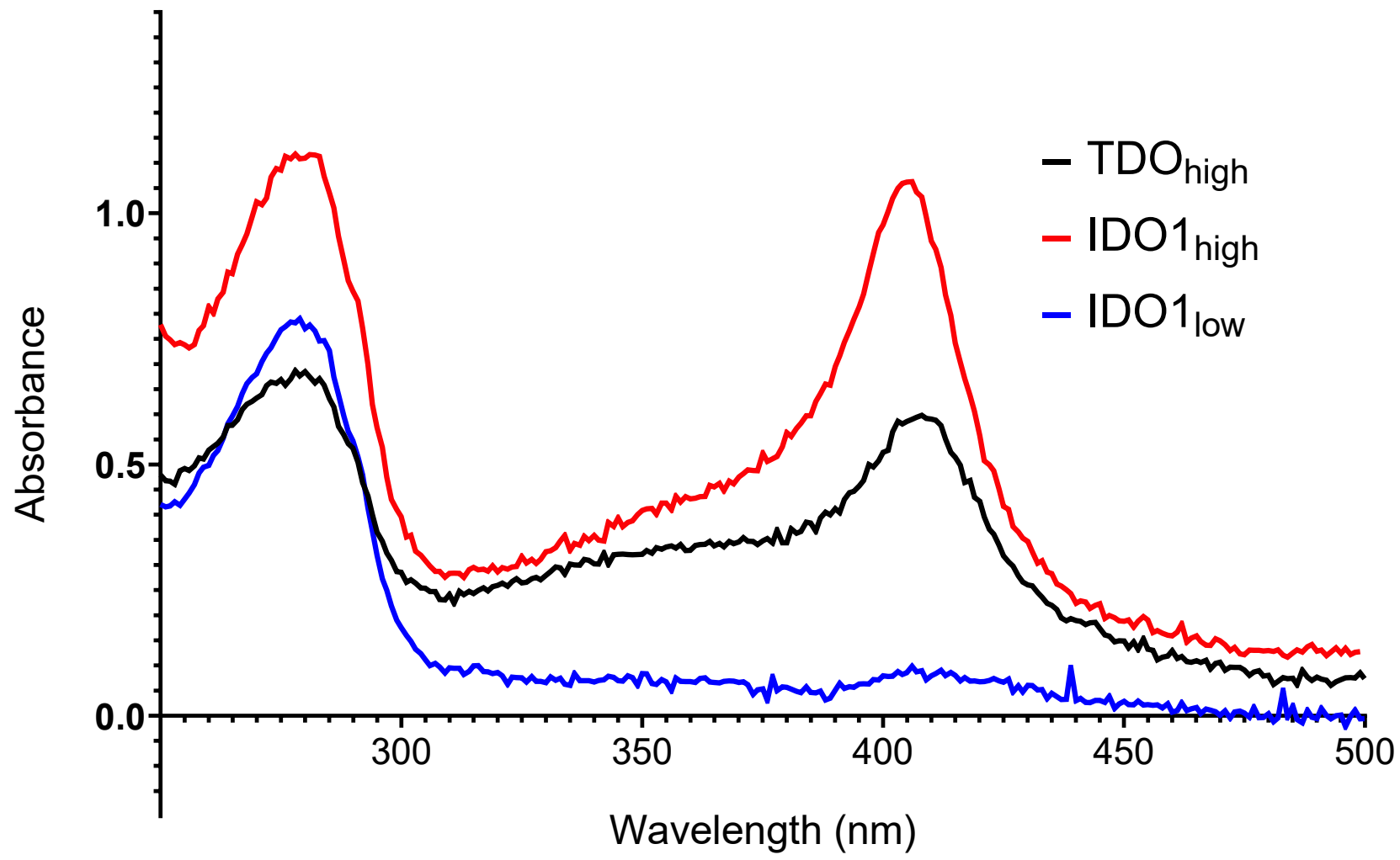

Figure S10. UV-Vis absorption spectrum of IDO1$_{high}$ (red) and TDO$_{high}$ (black) was recorded with a Varian Cary 50 spectrophotometer in the 250-500 nm range. The protein sample concentration was 10 µM in 50 mM potassium phosphate buffer (pH 7.4) 50 mM, EDTA 1 mM, DMSO 5%.

Concentration-dependent kinetic analysis of inhibitor-induced changes in the hIDO1 Soret band

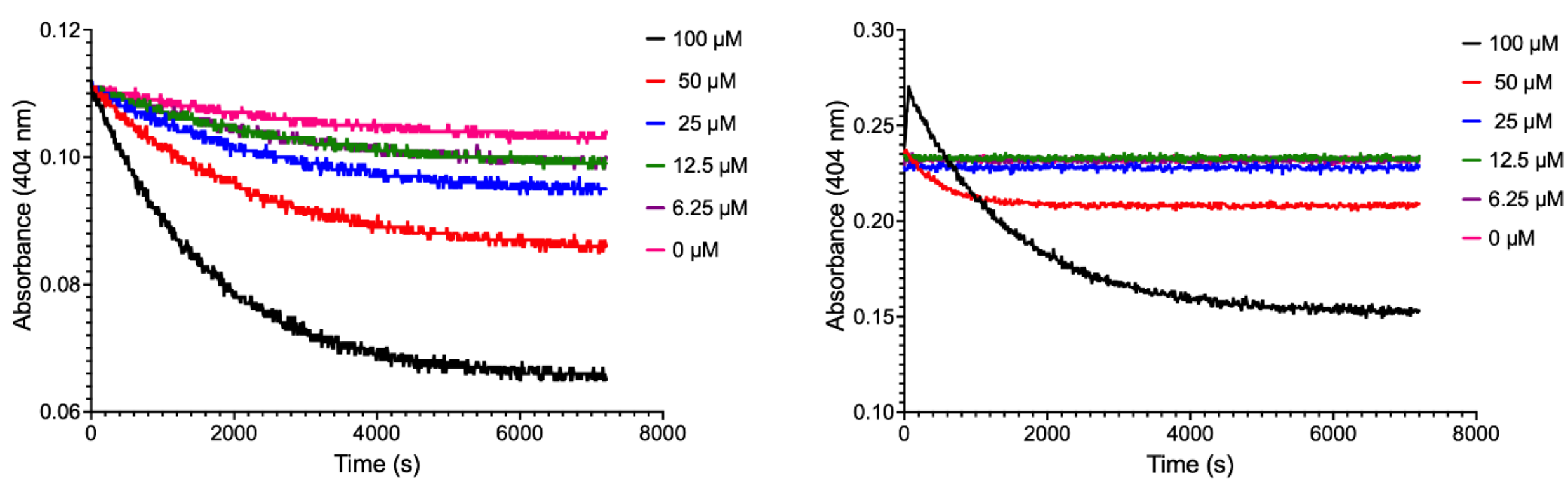

Figure S11. Concentration-dependent kinetic profiles of heme displacement from hIDO1 monitored by UV-visible spectroscopy. Absorbance at 404 nm was recorded over 7000 s (n=2, each concentration tested in technical triplicates) in a reaction mixture containing 3 µM hIDO1, 50 mM phosphate buffer (pH 7.0), 1 mM EDTA, and 5% DMSO, under an inhibitor concentration gradient of 0 to 100 µM. Linrodostat (left) induces a progressive concentration-dependent decrease in absorbance across the tested range. Compound **66** (right) also produced a concentration-dependent decrease in absorbance at the higher concentrations tested, whereas no clearly detectable change was observed below a defined concentration range.

Enzymatic assays

IDO1 activity at 25 and 37 °C

Michaelis-Menten constants (Km and Vmax) for the recombinant enzyme were determined using Trp as a substrate. A 120-minute incubation at 37°C was performed to evaluate enzyme kinetics under forced conditions (Figure S6). The results showed minimal variation in Km and Vmax between the two temperatures, indicating the functional stability of the enzyme. At 25°C, the Km was 0.301 mM, while at 37°C it was 0.414 mM. Similarly, Vmax was 0.006 mM/min at 25°C and 0.008 mM/min at 37°C. These values align closely with data reported in the BRENDA enzyme database.

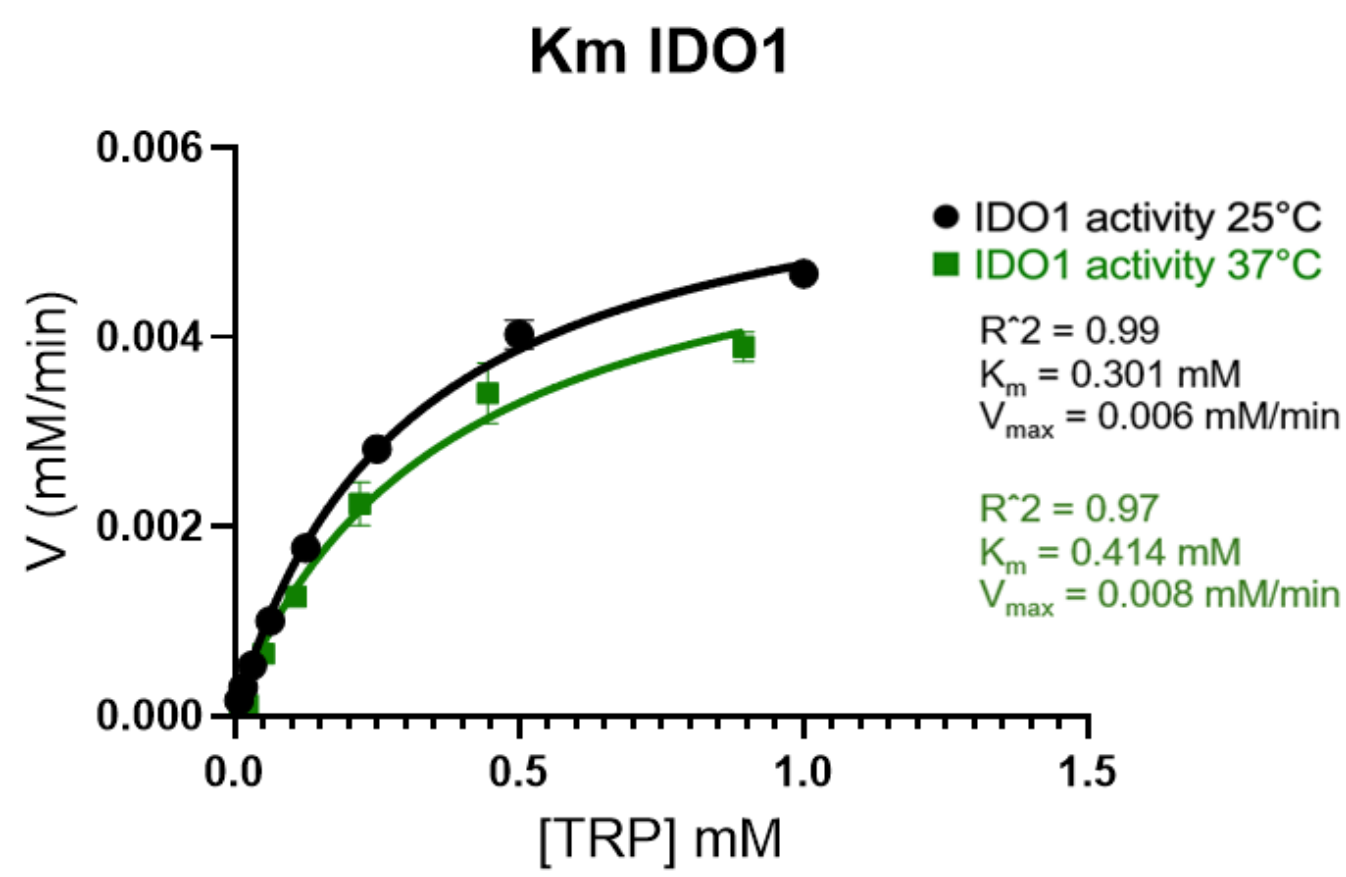

Figure S12. Michaelis-Menten curves for IDO1 at 25°C (black) and 37°C (green), with corresponding Km, Vmax, and R^2 values.

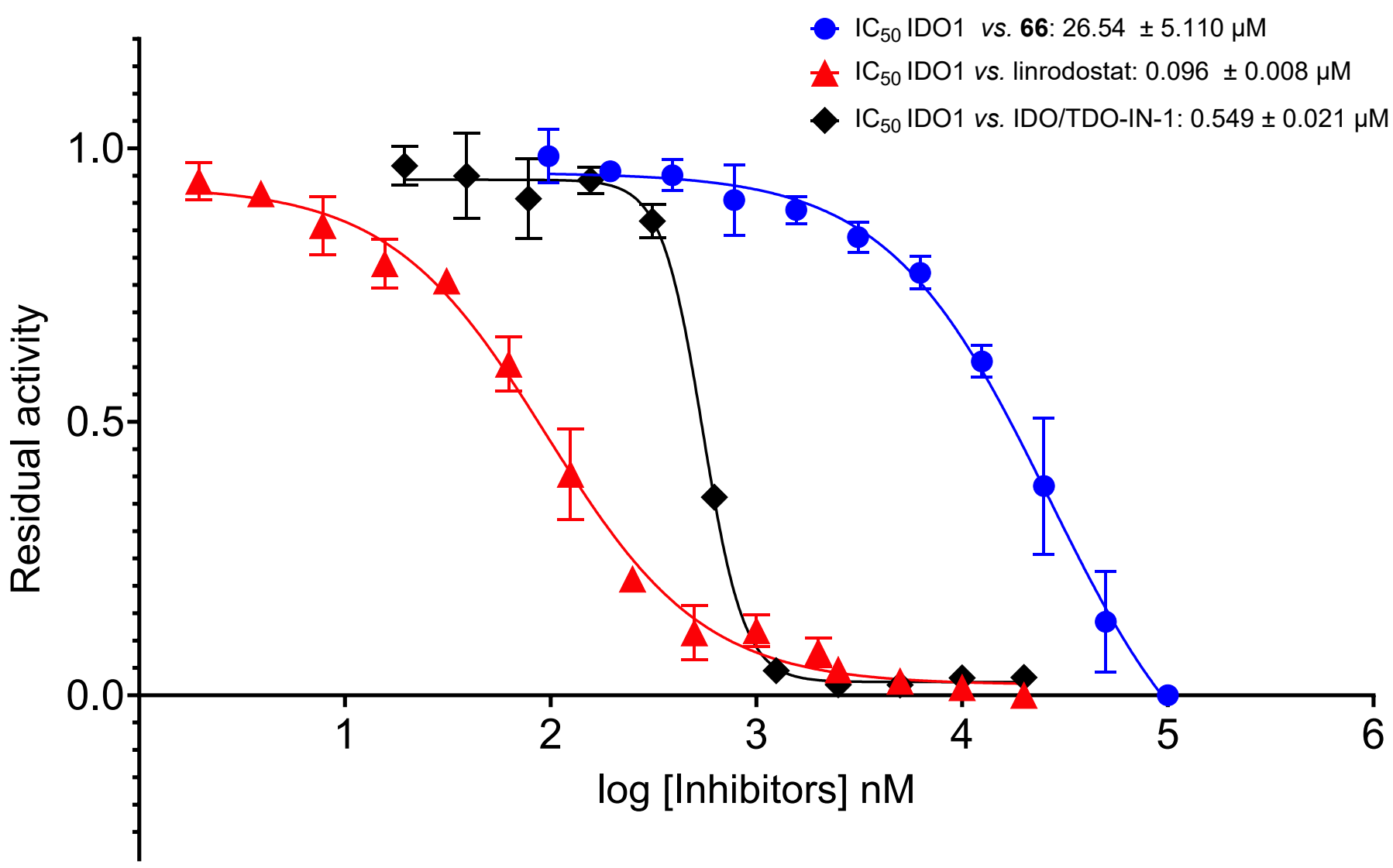

Figure S13. IDO1 inhibition curves in the presence of compound **66** (in blue), *apo*-IDO1 inhibitor linrodostat (in red), and dual *holo*-IDO1/TDO inhibitor IDO/TDO-IN-1 (in black) with corresponding IC_{50} and ± S.D. from two independent experiments. IDO1 activity was monitored at 37°C.

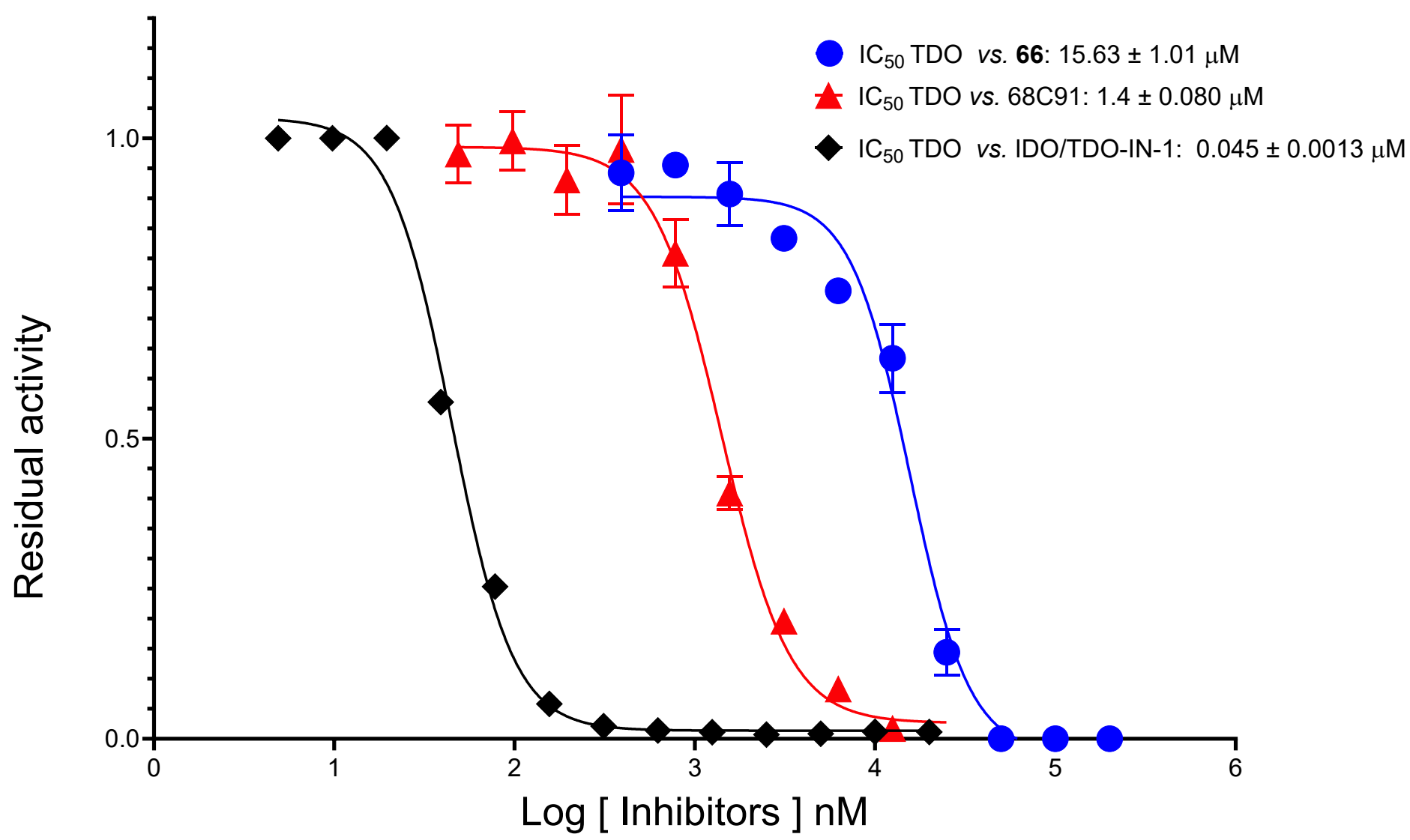

Figure S14. TDO inhibition curves in the presence of compound **66** (in blue), TDO inhibitor 680C91 (in red) and dual *holo*-IDO1/TDO inhibitor IDO/TDO-IN-1 (in black) with corresponding IC_{50} and ± S.D. from two independent experiments. TDO activity was monitored at 37°C.

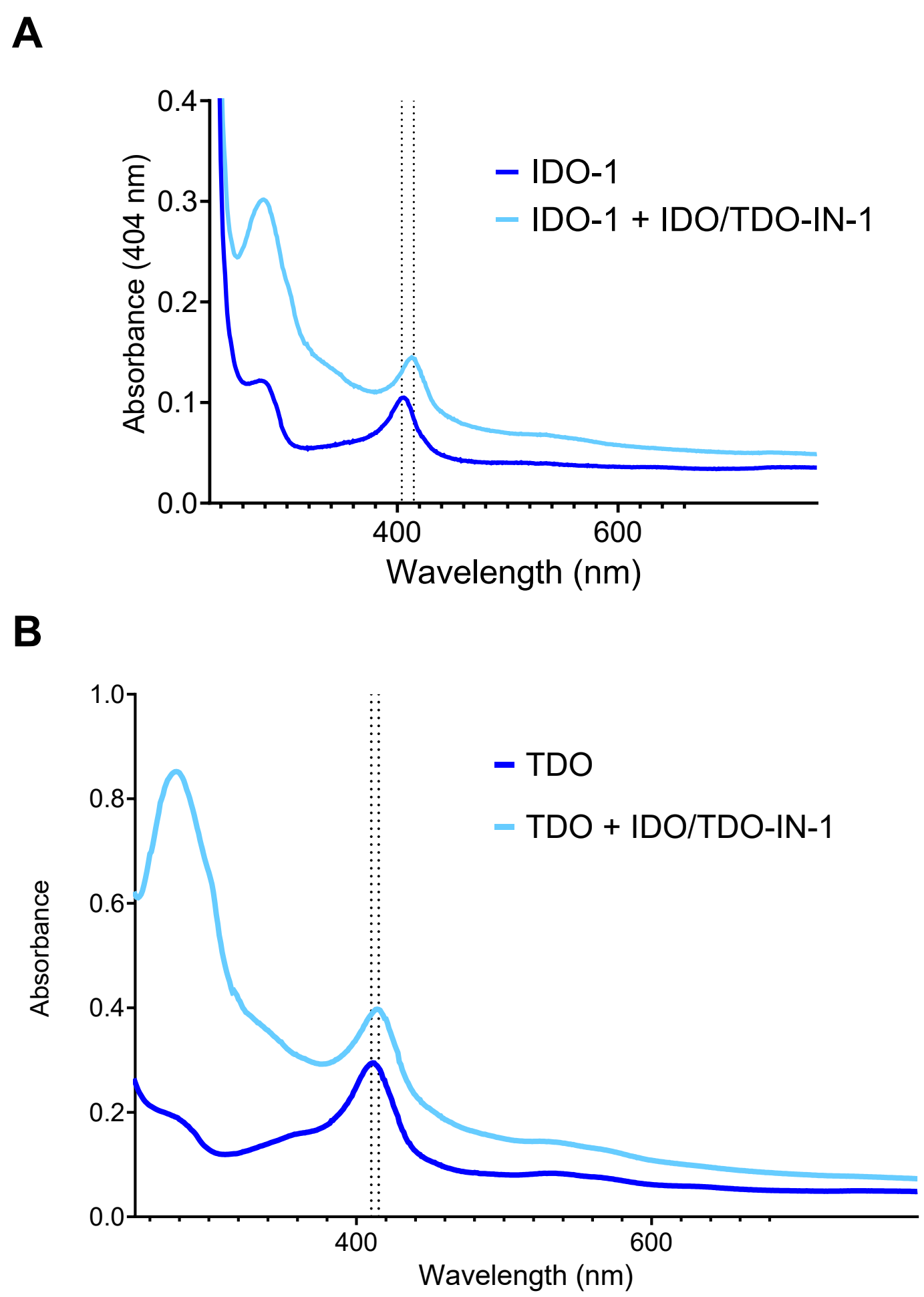

Figure S15. a) UV-vis absorption spectra of IDO1 (250–780 nm) in the absence (in blue) and in presence of compound IDO/TDO-IN-1 (in sky blue) at 50 µM. b) UV-vis absorption spectra of TDO (250–780 nm) in the absence (in blue), presence of compound IDO/TDO-IN-1 (in sky blue) at 50 µM.

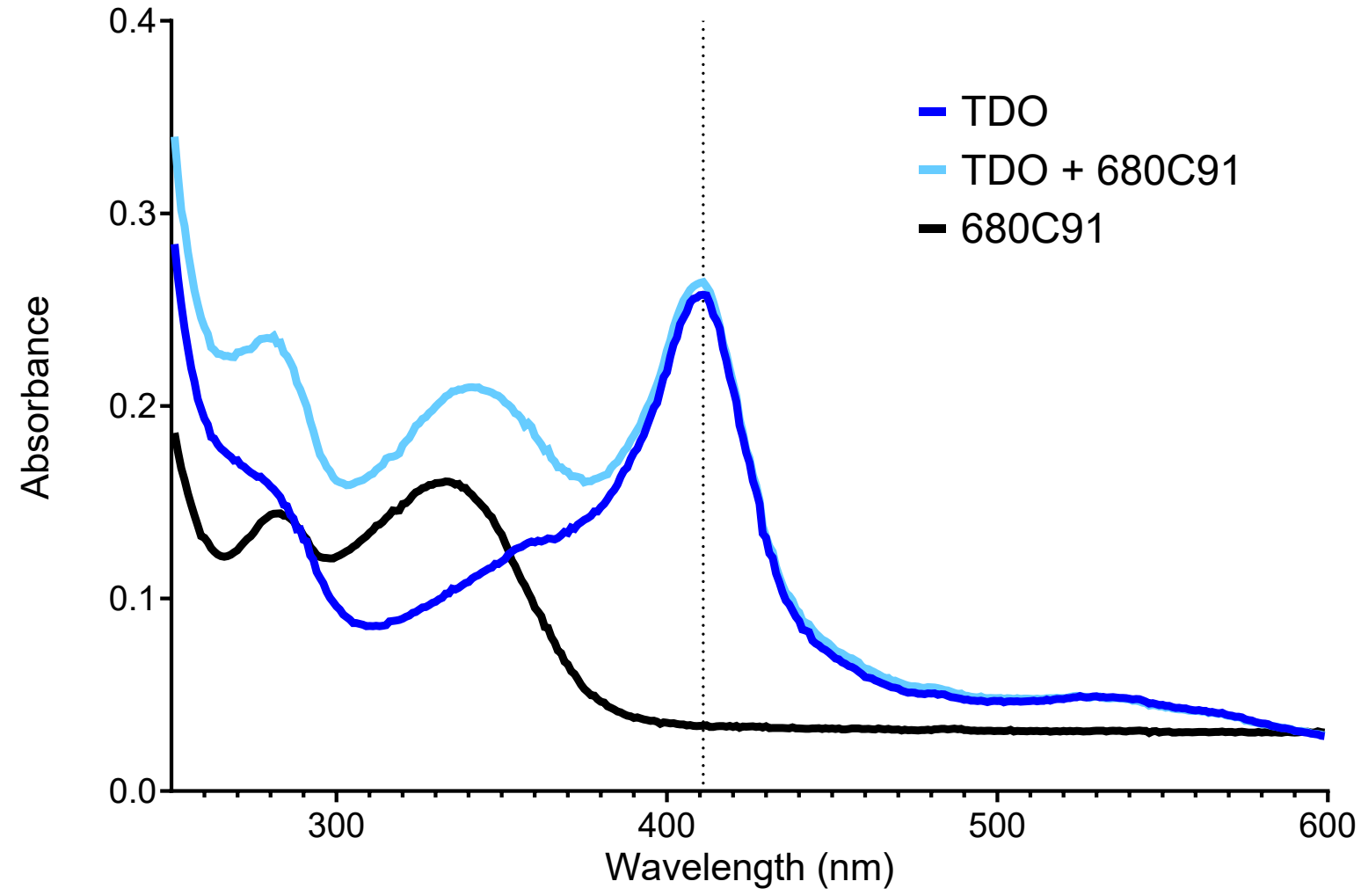

Figure S16. UV-vis absorption spectra of TDO (250–600 nm) in the absence (in blue), presence of compound 680C91 (sky blue) and with only 680C91 (in black) at 50 µM.

FPocket-based shape analysis of *apo*-IDO1 and *apo*-TDO

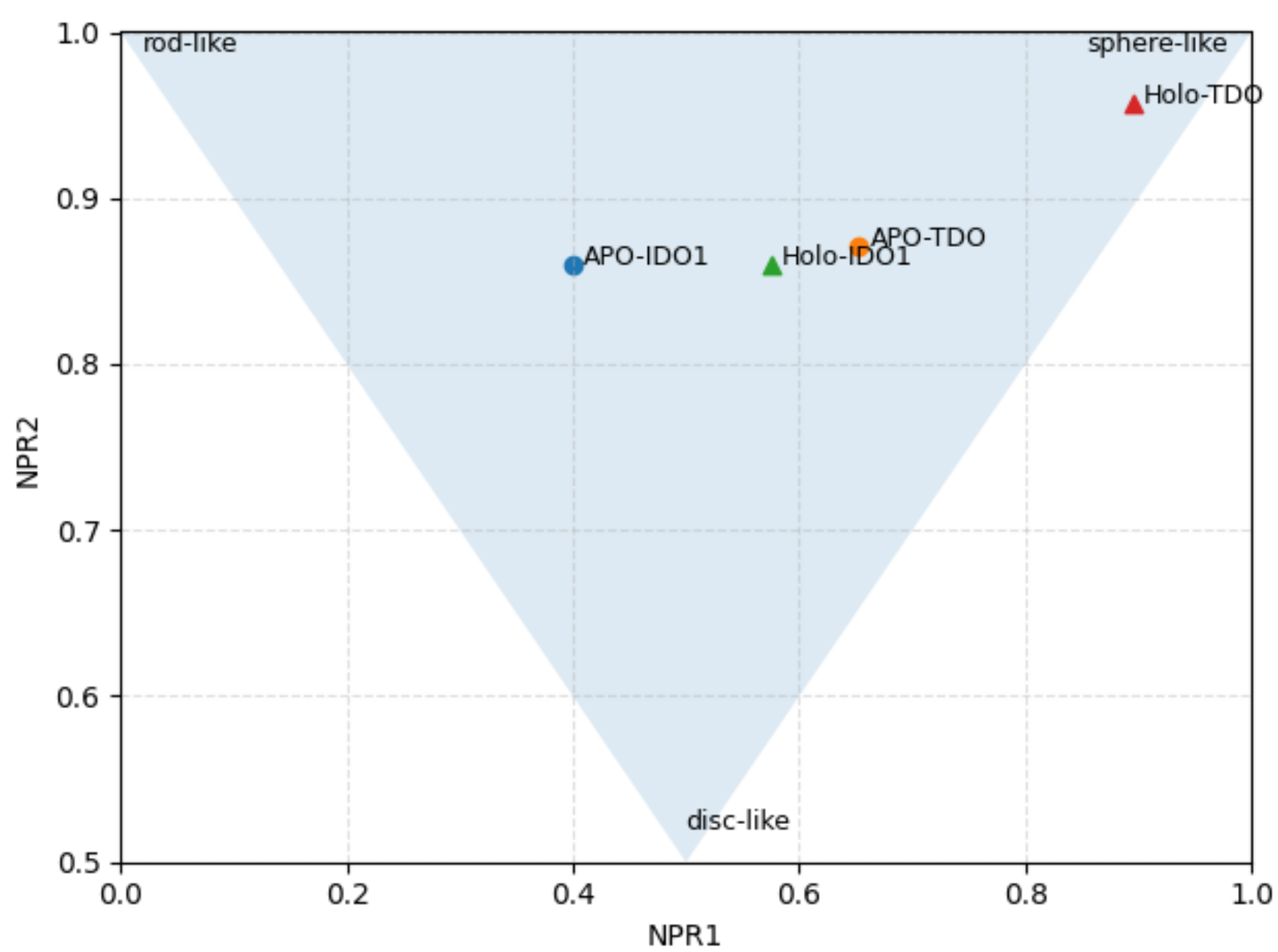

Figure S17. NPR plot derived from FPocket analysis showing the shape distribution of IDO1 and TDO binding pockets (*apo* and *holo* forms). Pocket geometry is described using the normalized principal moment ratios NPR1 and NPR2, distinguishing rod-like, disc-like, and sphere-like shapes.

Interference with murine IDO1 signaling

Immunoprecipitation and phosphatase assay

P1.IDO1 cells were pre-incubated for 4 hours at 37°C with compound **66** 1 µM or DMSO as control, before assessing the phosphatase activity associated with the IDO1 protein. For each condition, the IDO1 protein was immunoprecipitated from 6 x 10^6 cells lysed in 400 µL of Lysis Buffer (50 mM Tris-HCl pH 7.5, 150 mM NaCl, 1% Nonidet P-40, and a cocktail of protease inhibitors). The IDO1 protein was immunoprecipitated overnight at 4°C by a specific monoclonal rabbit antibody raised in our lab, followed by the addition of Protein G-Sepharose (Sigma-Aldrich, MO, USA) for 2 hours. The phosphatase activity associated with immunoprecipitated IDO1 was assessed by a specific kit purchased from Sigma-Aldrich (MO, USA), according to the manufacturer's instructions. The resulting immune-complexes were analyzed by immunoblotting, as described below.

Endosome isolation

Endosomes were isolated using a Minute Endosome Isolation and Cell Fractionation Kit (Invent Biotechnologies, Inc., MN, USA) according to the manufacturer's instructions and as previously reported[5]. The assay allows the separation of isolated endosomes from the cytosolic fraction. Protein concentrations were quantified using the Bradford assay prior to electrophoresis, and equal amounts of protein were loaded for each condition (8 µg for EE fractions and 5 µg for cytosolic fractions). Both fractions were analyzed by immunoblotting, using the antibodies described below.

Immunoblot analysis

The following antibodies were used for immunoblot analysis: mouse mAb anti-SH-PTP2 (clone B-1, Cat #sc-7384, Santa Cruz Biotechnology, Dallas, TX, USA), mouse mAb anti-IDO1 (clone 8G11, Cat #MABS485, Sigma-Aldrich, Merck, Darmstadt, Germany), rabbit mAb anti-Rab5 (clone C8B1, Cat# 3547, Cell Signaling Technology, Danvers, MA, USA), and rabbit mAb anti-GAPDH (clone 14C10, Cat #2118, Cell Signaling Technology, Danvers, MA, USA).

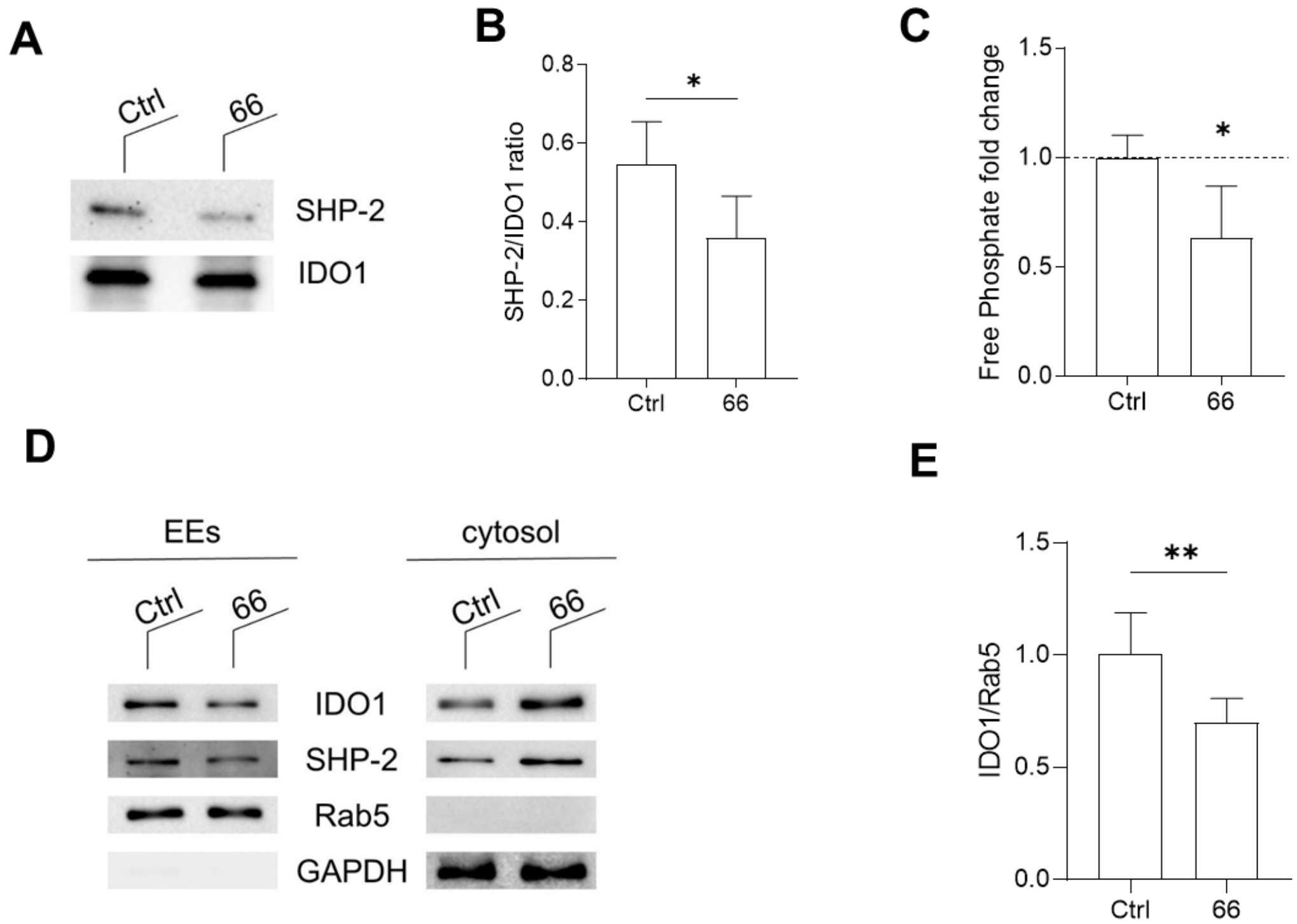

Figure S18. Compound **66** interferes with IDO1-SHP2 interaction and with IDO1 localization in murine cells. (A) Immunoblot analysis of co-immunoprecipitates from P1.HTR tumor cells stably transfected with mouse IDO1 and treated with **66** or the vehicle alone as a control (Ctrl). (B) Quantitative analysis of six independent immunoblot experiments, one of which is represented in (A). (C) Phosphatase activity produced by IDO1 immunoprecipitated from P1.IDO1 cells treated as in (A). Levels of free phosphate (pmol) generated during the incubation of a tyrosine-phosphorylated peptide with the co-immunoprecipitated IDO1 proteins from each condition were presented as a fold change relative to untreated cells (dotted line, 1-fold). (D) Immunoblot analysis of early endosomes (EEs) and cytosol isolated from **66**-treated P1.IDO1 cells. Cells incubated with the vehicle DMSO were used as a control (Ctrl). (E) Quantitative analysis of six independent immunoblot experiments, one of which is represented in (D). The IDO1:Rab5 ratio was calculated using densitometric quantification of the specific bands. In (A) and (D), one experiment representative of six is shown. In (B), (C) and (E), the results are representative of six independent experiments (mean ± S.D.), each performed in triplicate. *p< 0.05, **p<0.01 (Unpaired Student's *t*-test).

Purity evaluation of selected compounds by HPLC-UV analysis

LC-UV method for purity evaluation

Instrumentation:	Shimadzu HPLC system LC-10AD series
Column:	Synergi Polar (150 × 4.6 mm, 4 µm d_p) (Phenomenex).
Mobile Phase	Phase A: 0.2% formic acid in water HPLC grade

Phase B: 0.2% formic acid in acetonitrile HPLC grade

Analysis mode: Gradient of concentration.

Time (min)	% B
0.0	30
13.0	90
14.5	90
15.0	30
20.0	30

Detection: λ= 280 nm (for metabolic stability experiments); 220 and 268 nm (for purity)

Flow rate: 1 mL/min

Injected volume: 20 µL

Chemical purity of selected compounds

Each compound was dissolved in pure DMSO at a concentration of 1 mg/mL. Furthermore, a 1:10 dilution in water-acetonitrile (1:1) was performed prior HPLC analysis.

Compound **16**: purity $\lambda_{282\ nm}$>99%, $\lambda_{220\ nm}$>99%

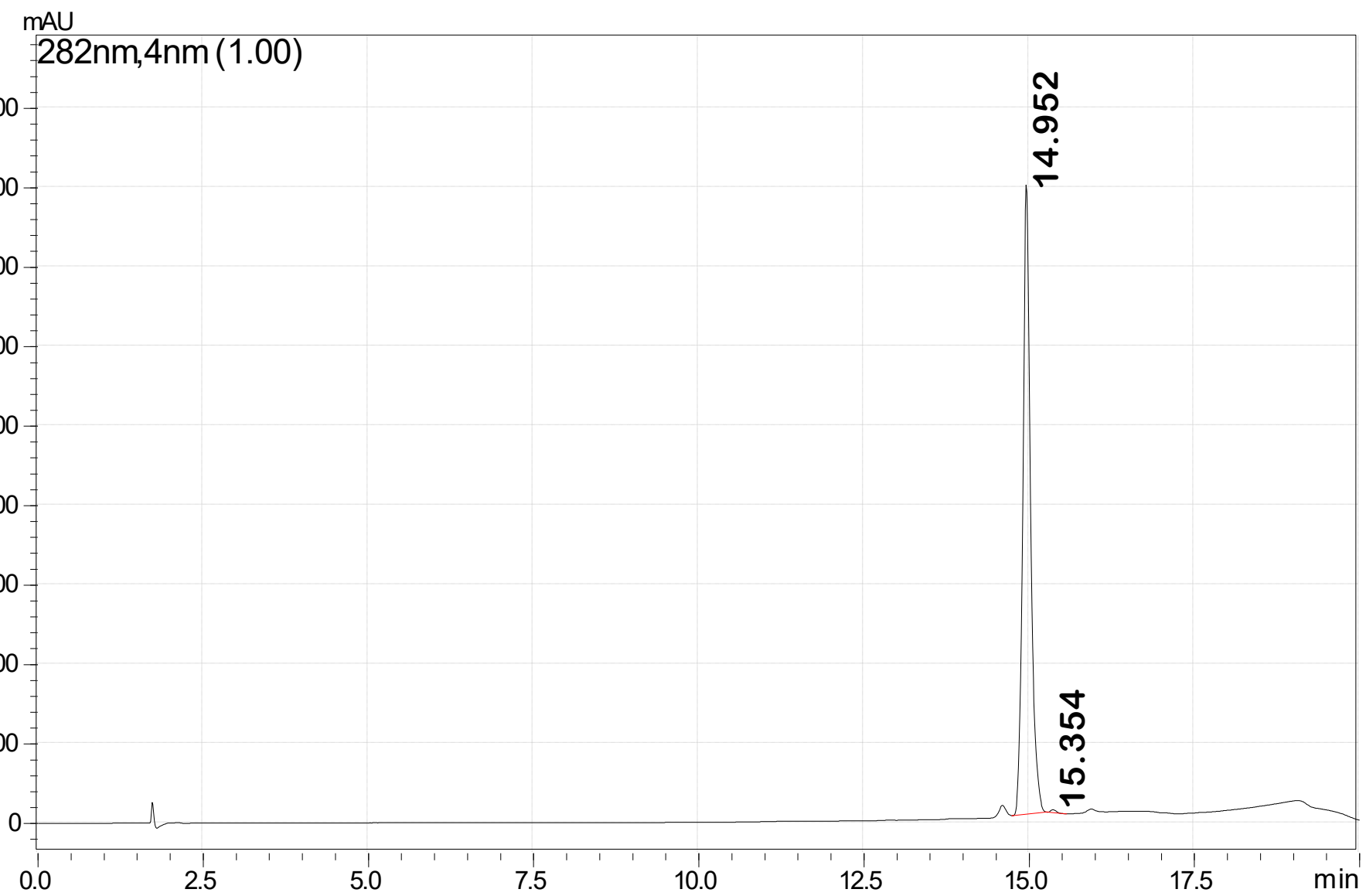

Compound **17**: purity $\lambda_{282\ nm}$=95.0%, $\lambda_{220\ nm}$>88.5%

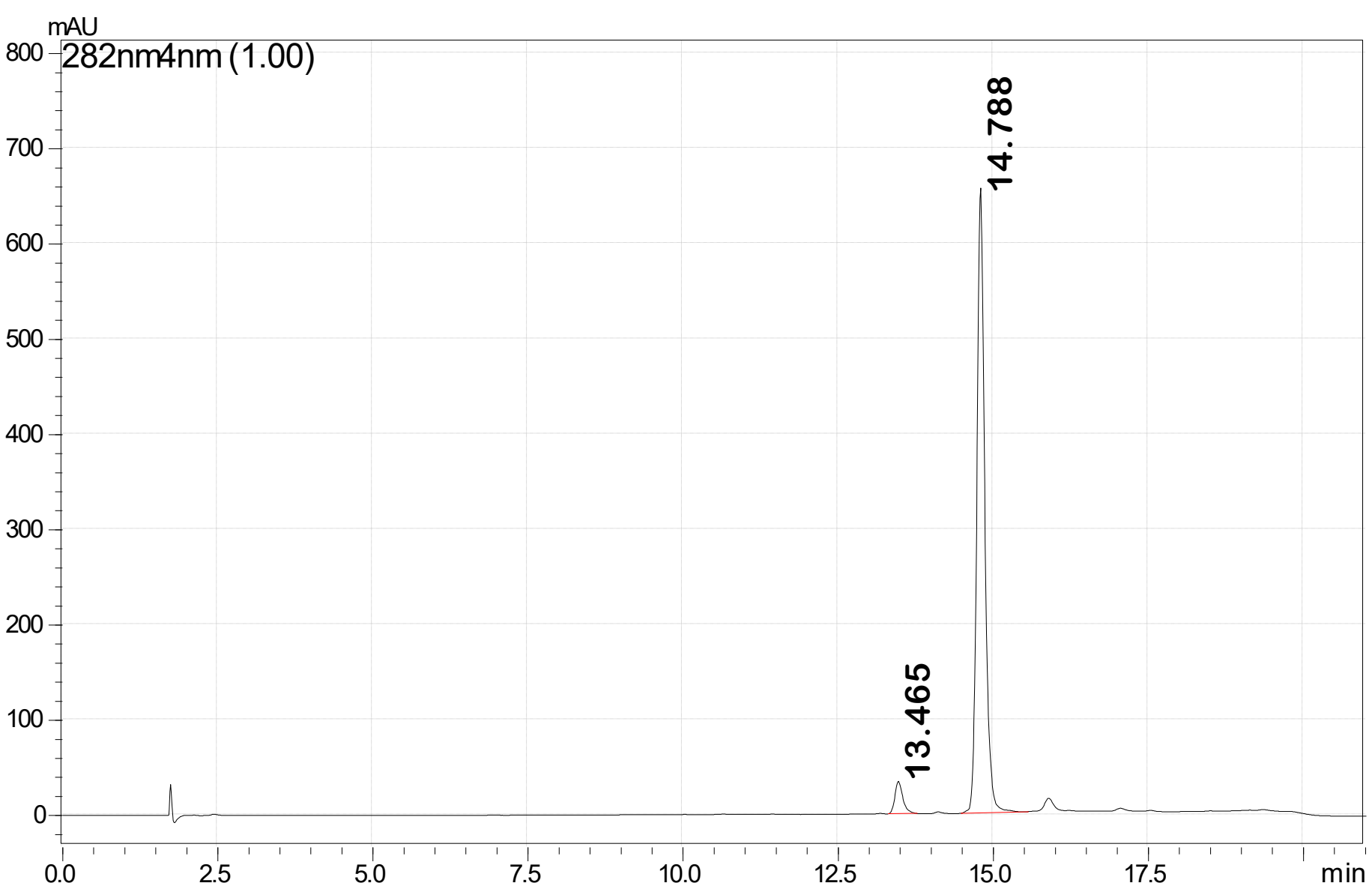

Compound **18**: purity $\lambda_{282\ nm}$=98.6%, $\lambda_{220\ nm}$>99%

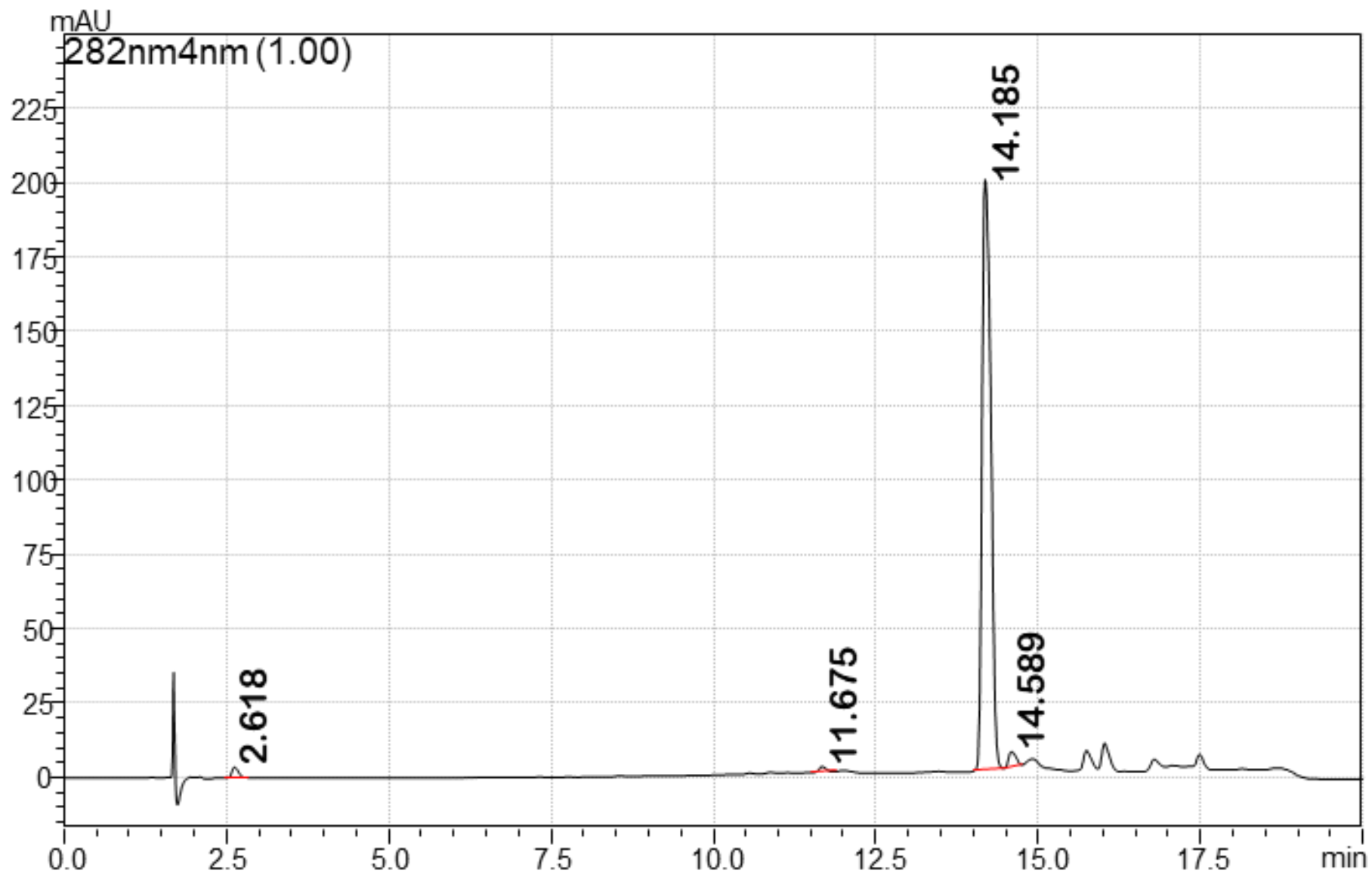

Compound **19**: purity λ_{282nm}=98.4%, $\lambda_{220\ nm}$>99%

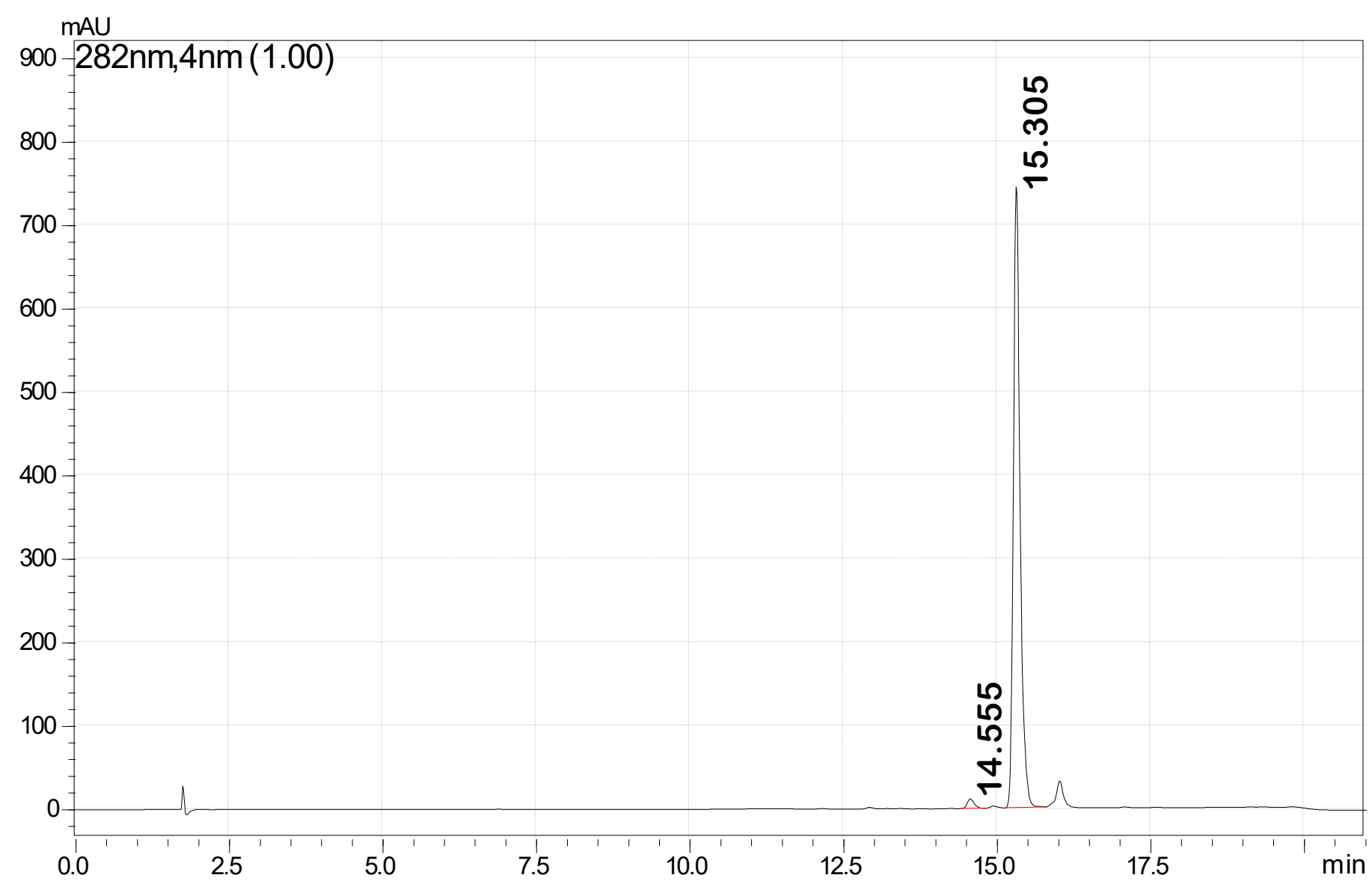

Compound **21**: purity λ_{282nm}>99%, $\lambda_{220\ nm}$>99%

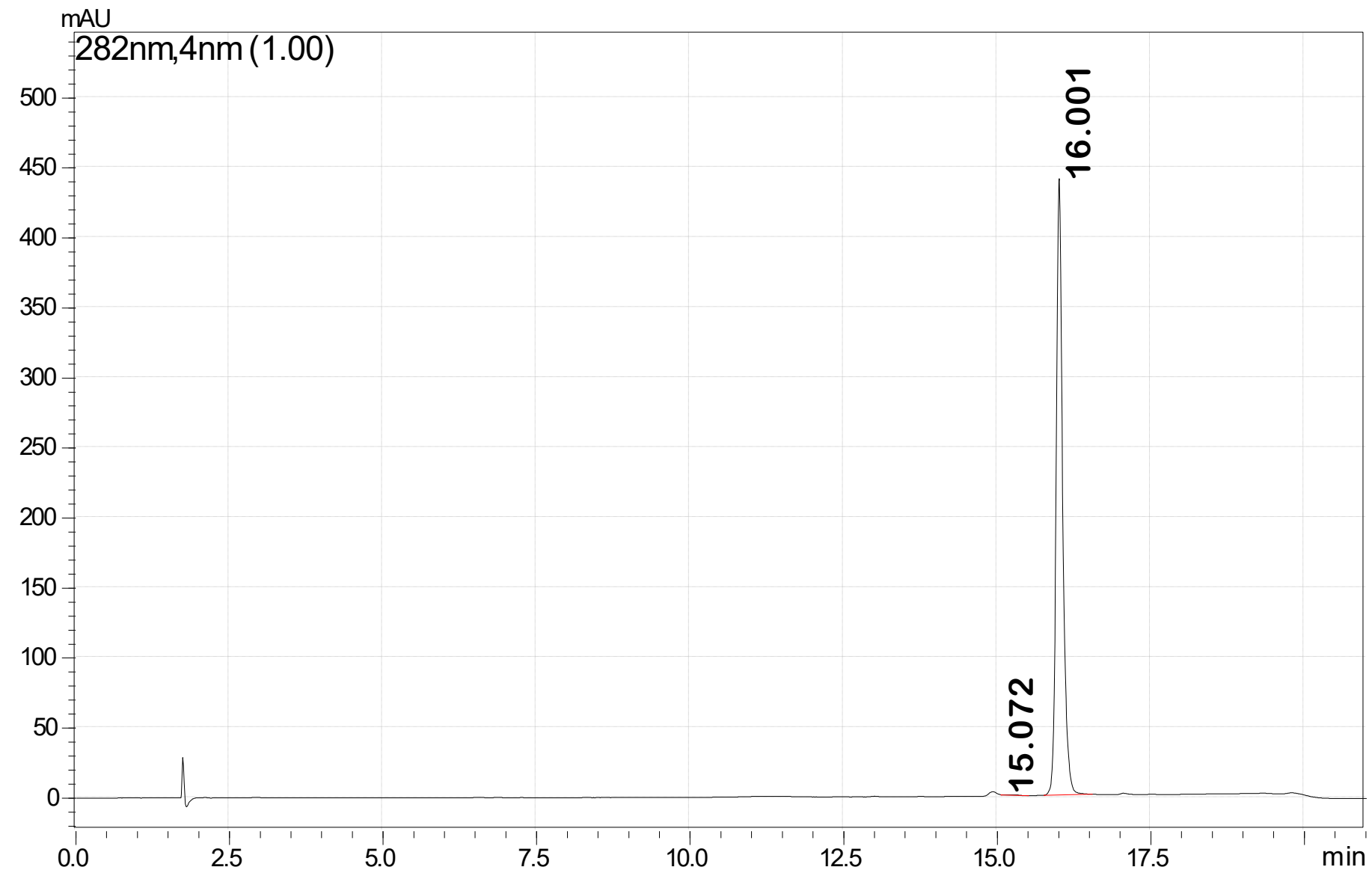

Compound **39**: purity λ_{282nm}>99%, $\lambda_{220\ nm}$>99%

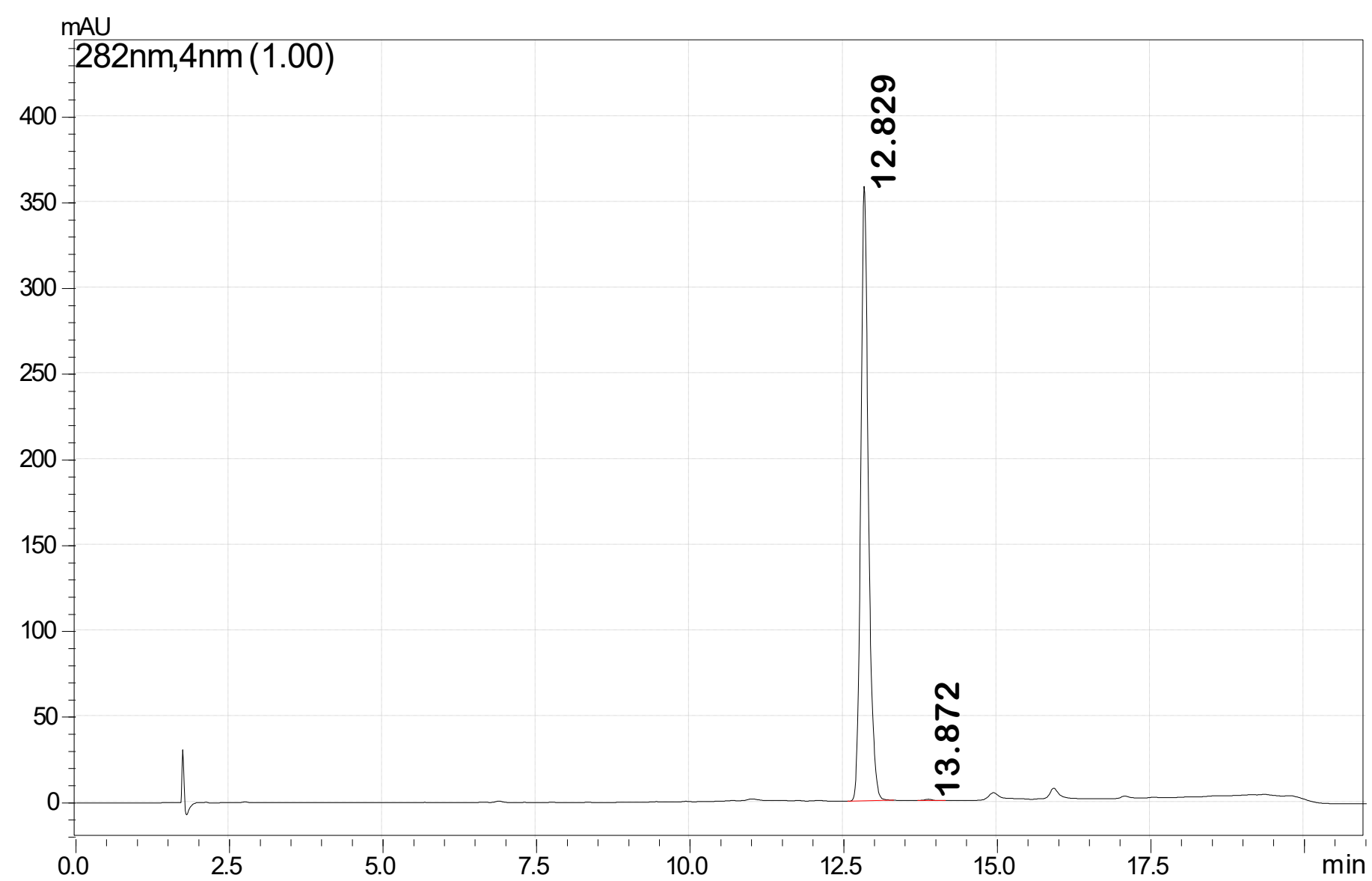

Compound **40**: purity λ_{282nm}=95.9%, λ_{220nm}=95.1%

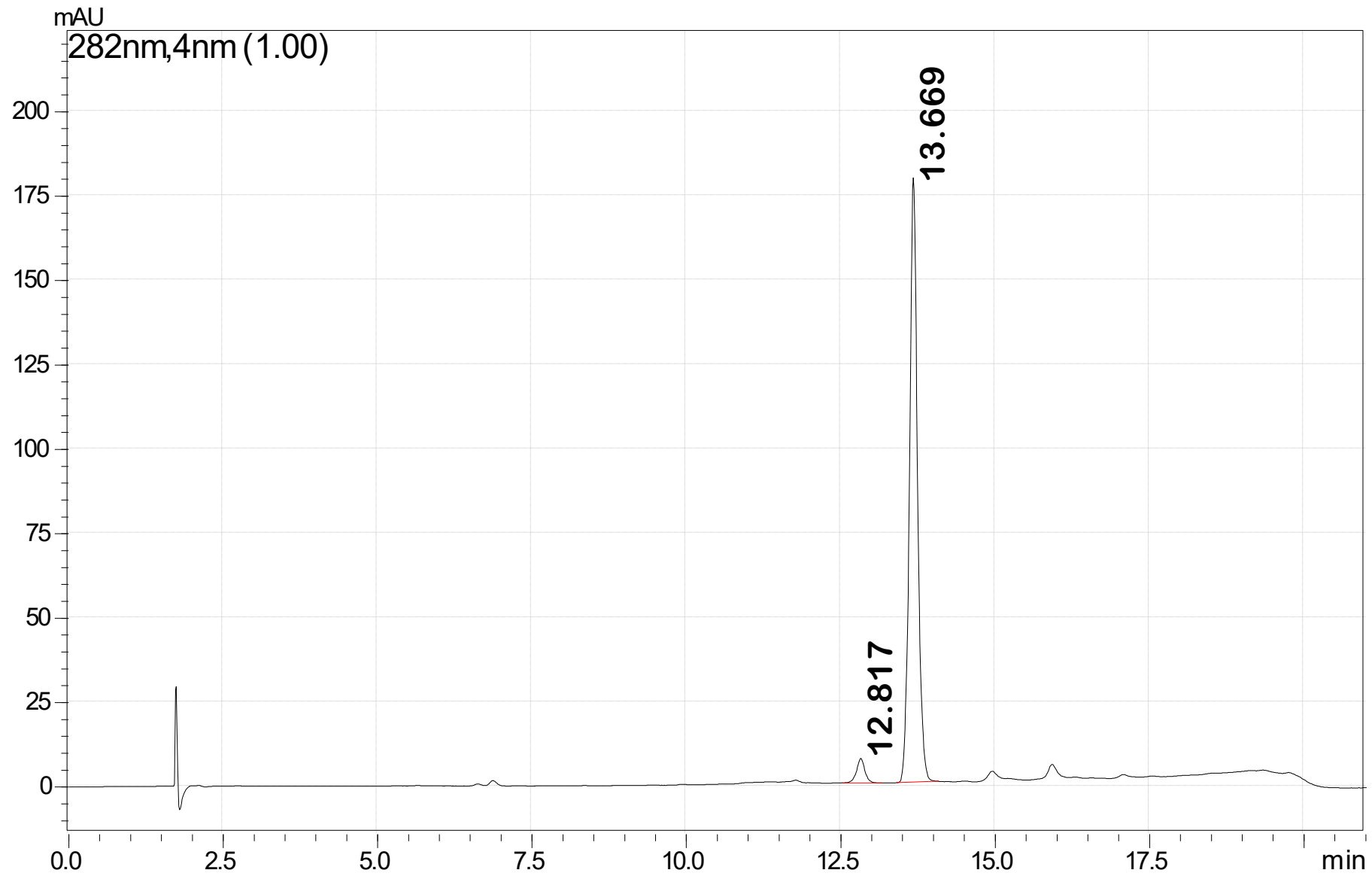

Compound **41**: purity $\lambda_{254\ nm}$>98.1%, $\lambda_{220\ nm}$>99%

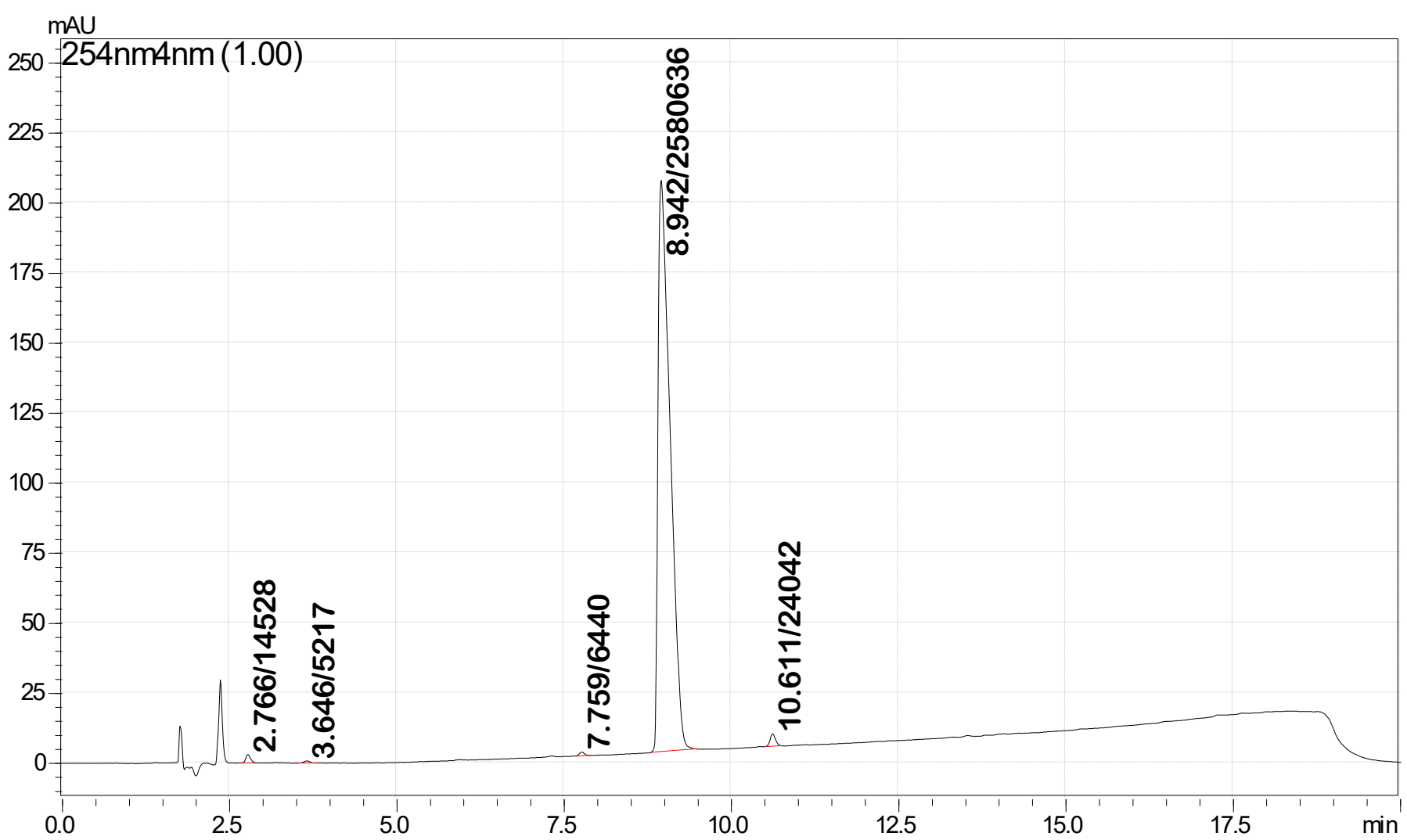

Compound **66**: purity λ_{282nm}=96.8%, λ_{220nm}=98.5%

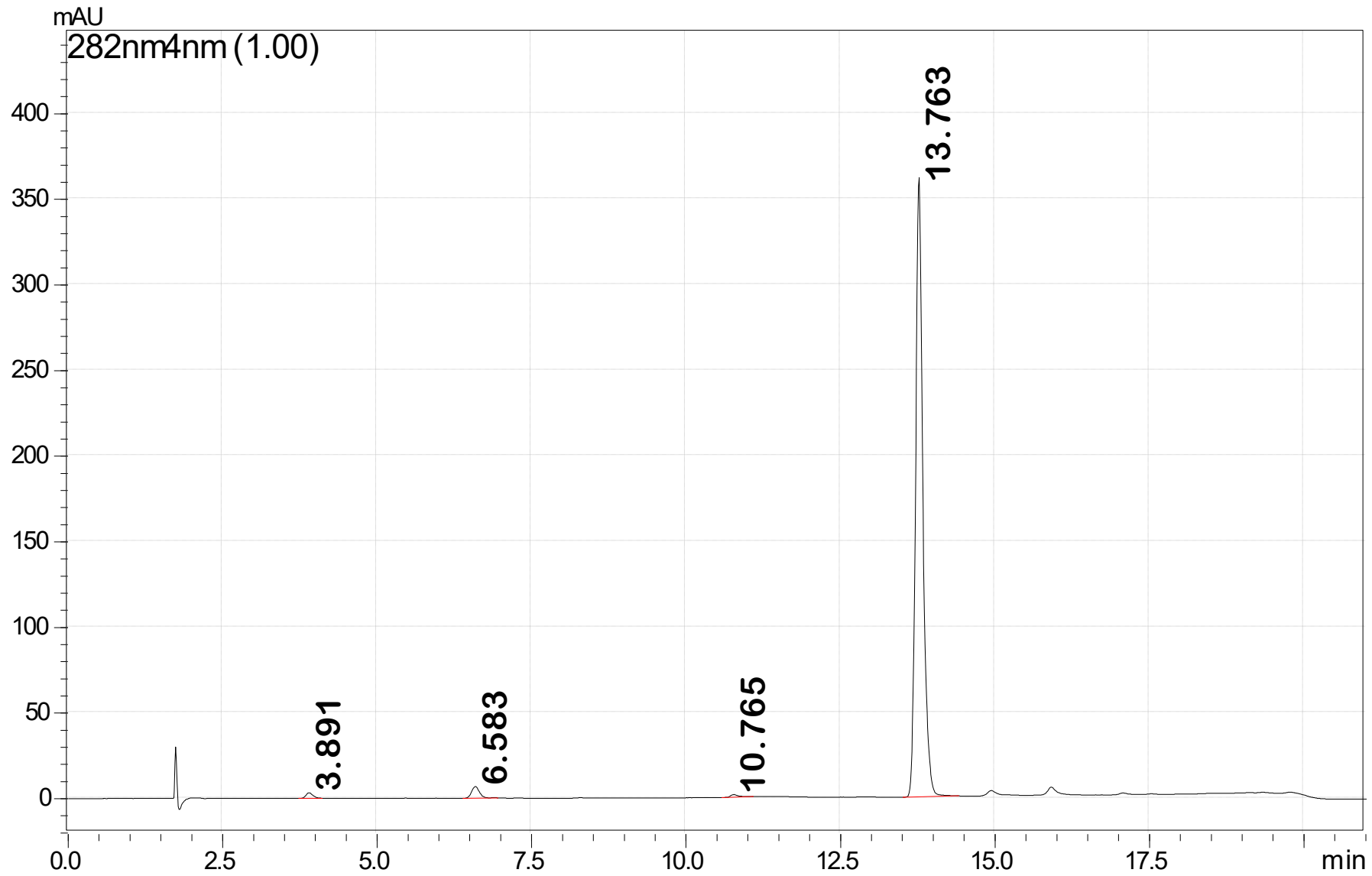

Compound **67**: purity λ_{268nm}=95.5%, λ_{220nm}=96.2%

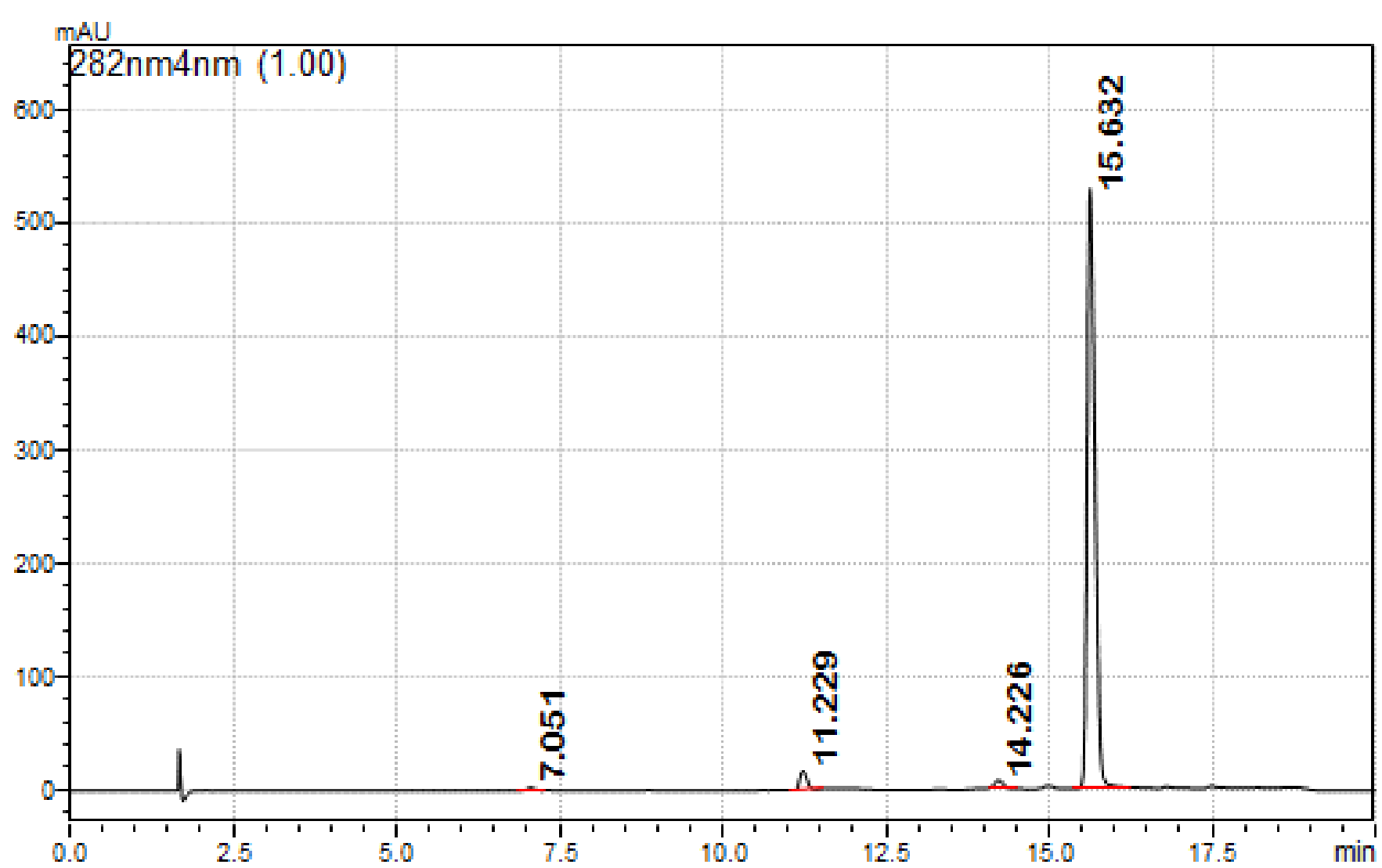

16, ^{1}H-NMR (400 MHz, chloroform-*d*)

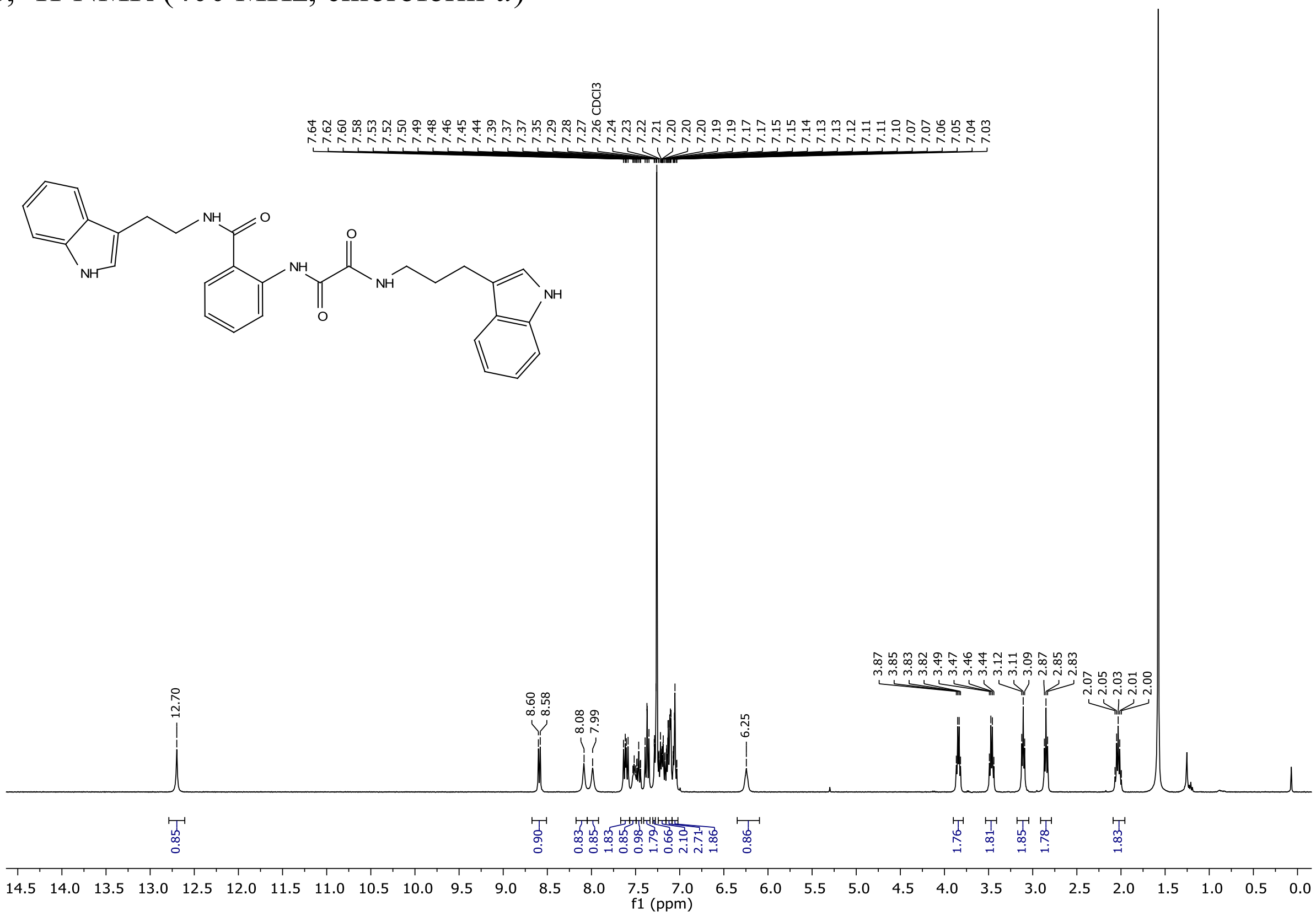

16, ^{13}C-NMR (101 MHz, acetone-d_6)

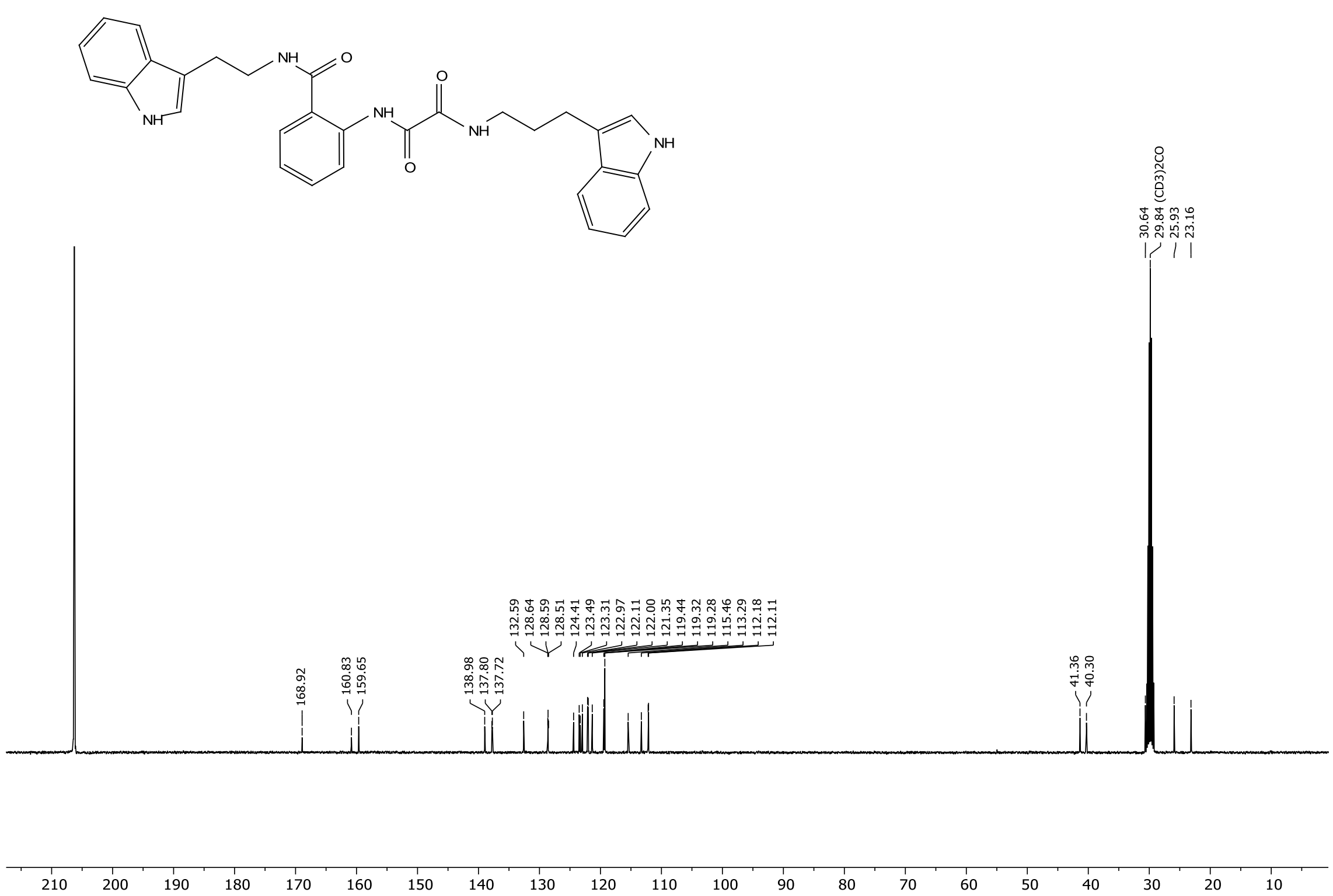

17, ^{1}H-NMR (400 MHz, acetone-d_6)

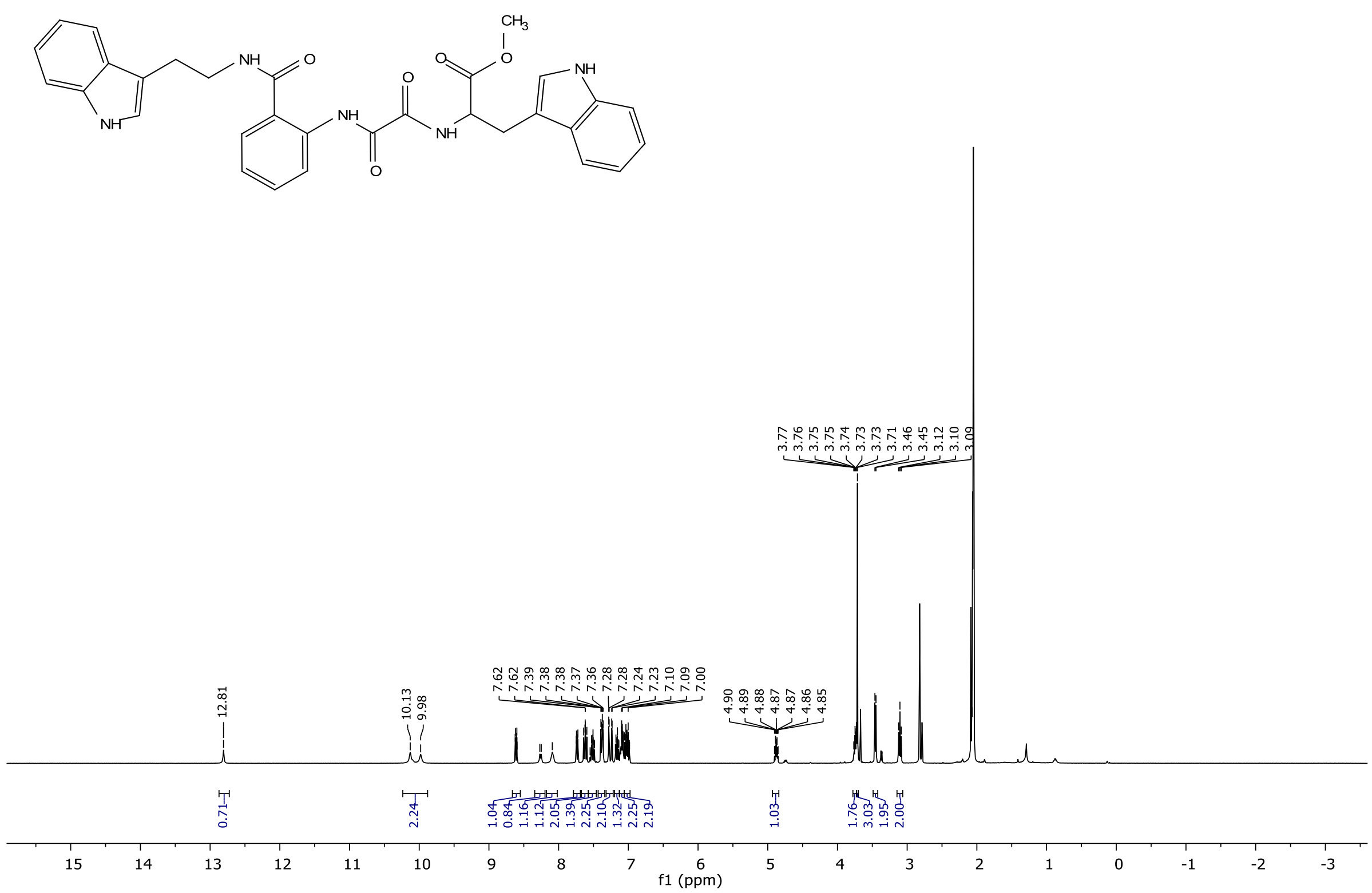

17, ^{13}C-NMR (101 MHz, acetone-d_6)

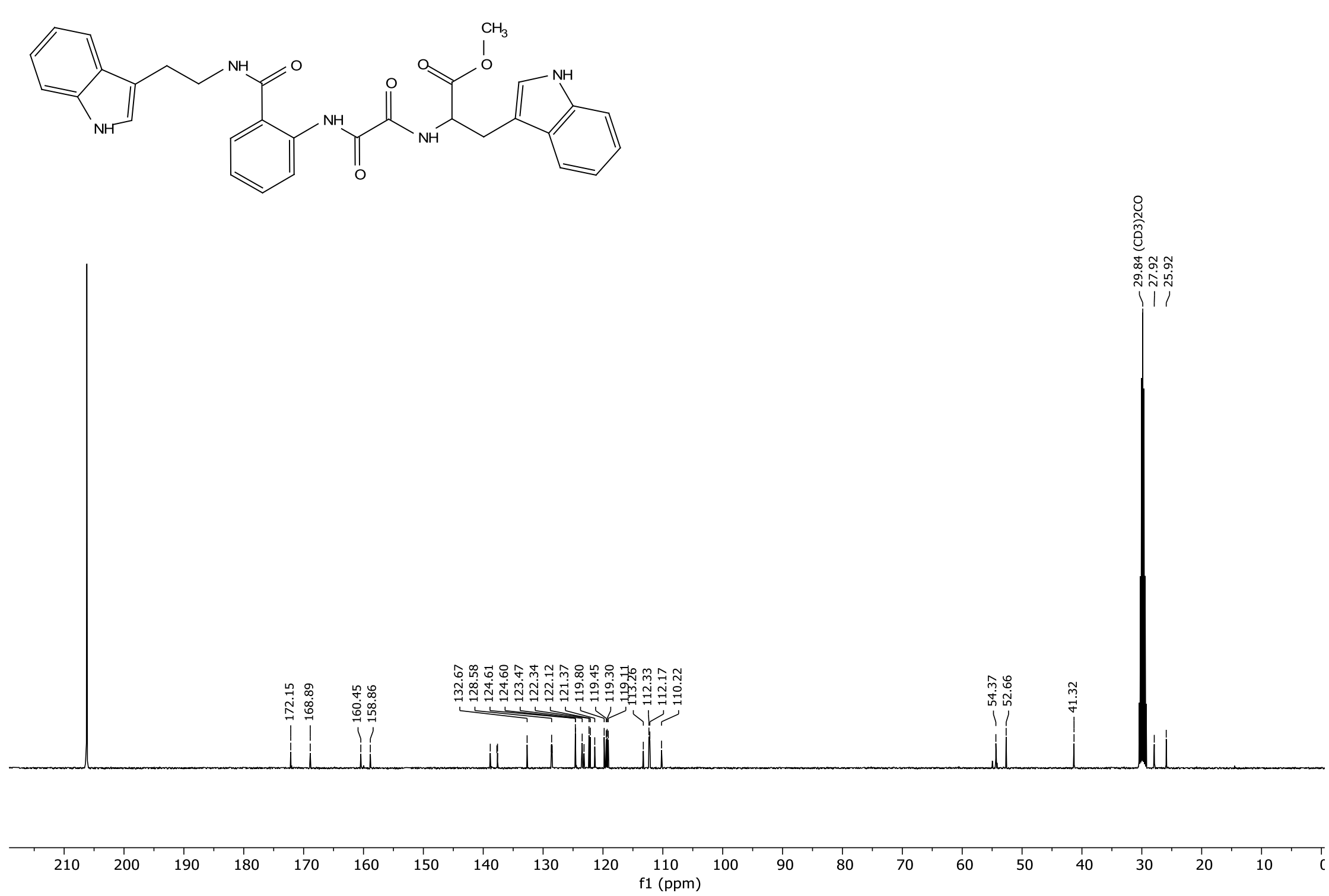

18, ^{1}H-NMR (400 MHz, acetone-d_6)

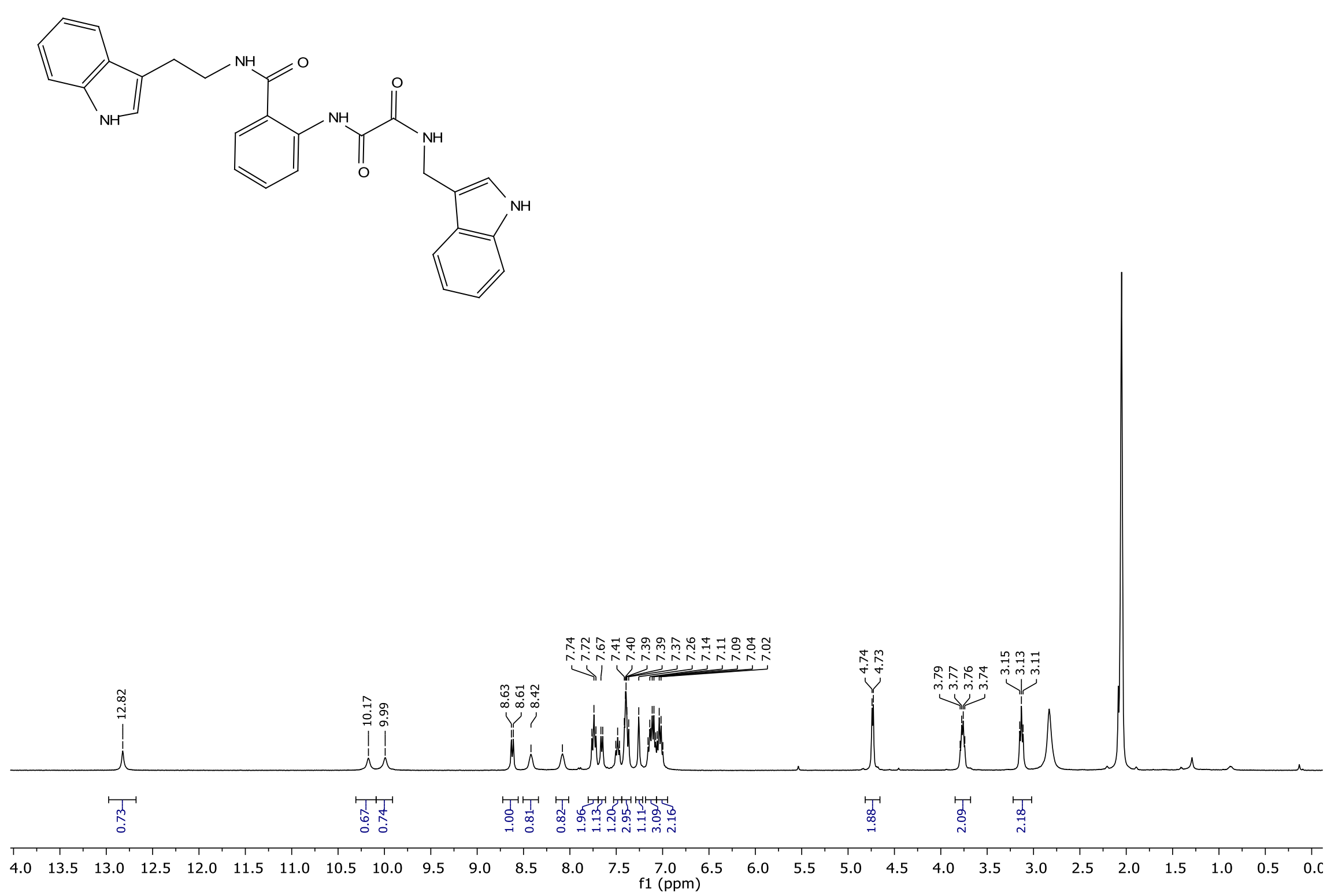

18, ^{13}C-NMR (101 MHz, acetone-d_6)

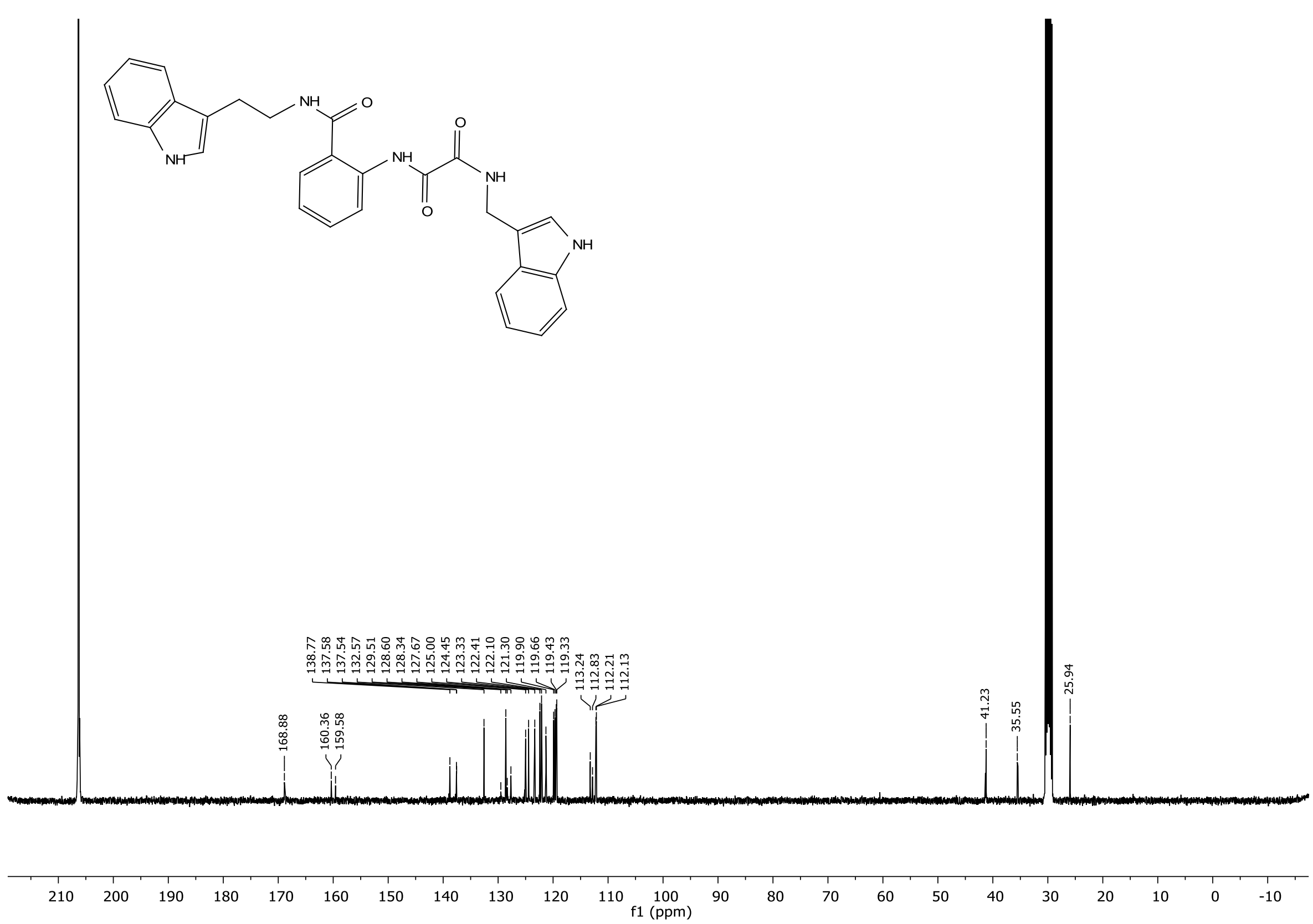

19, ^{1}H-NMR (400 MHz, acetone-d_6)

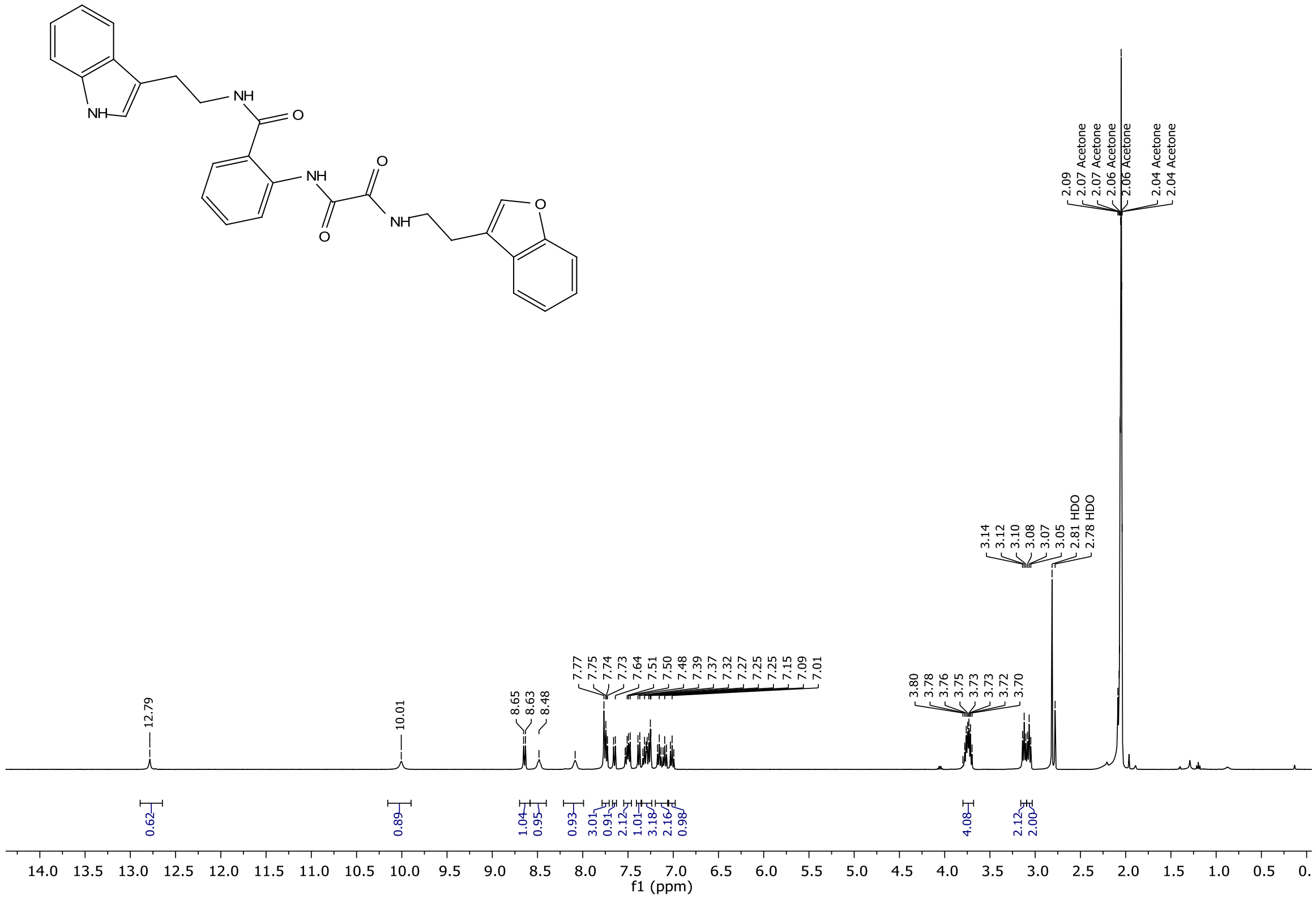

19, ^{13}C-NMR (101 MHz, acetone-d_6)

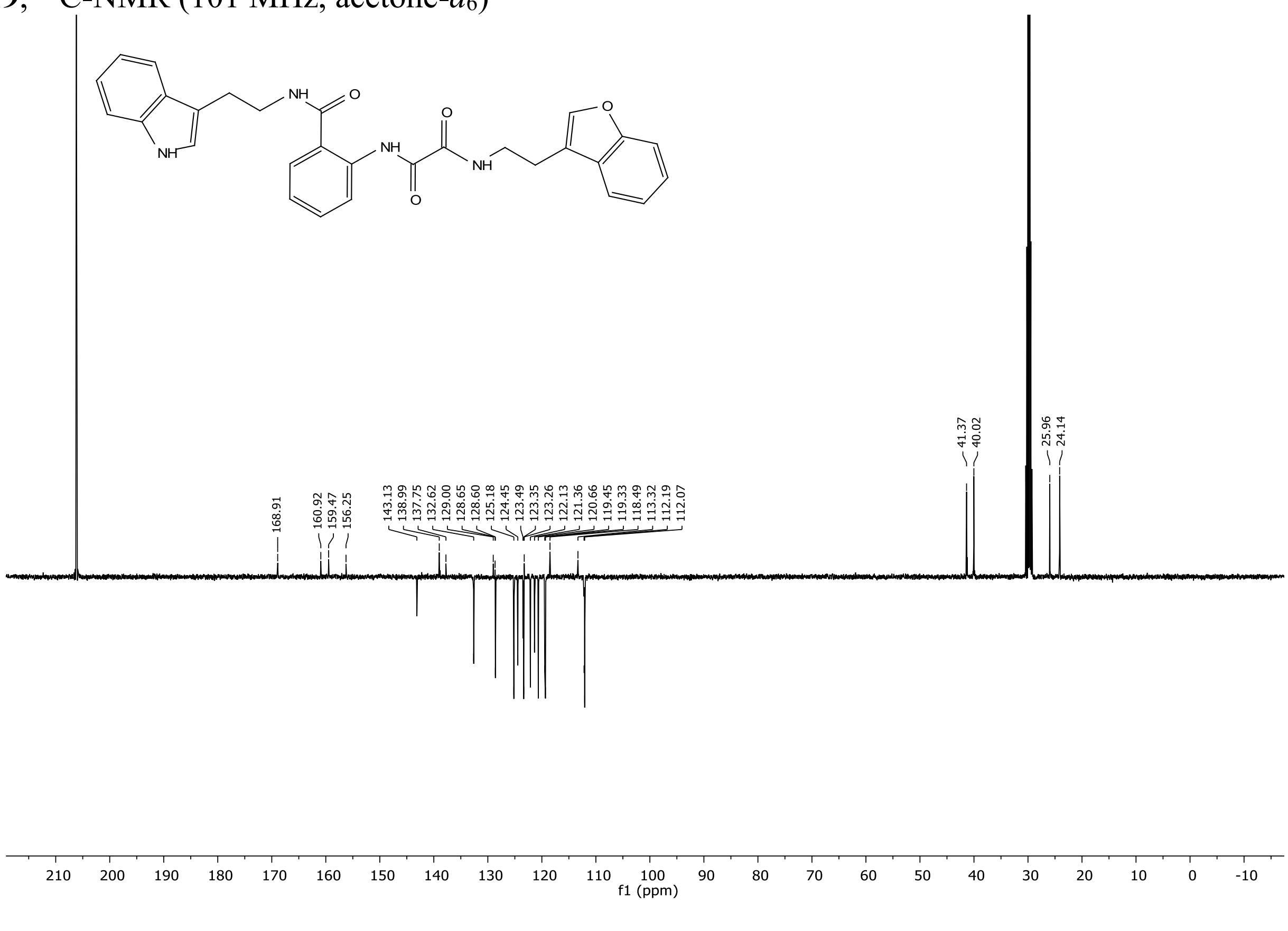

21, ^{1}H-NMR (400 MHz, acetone-d_6)

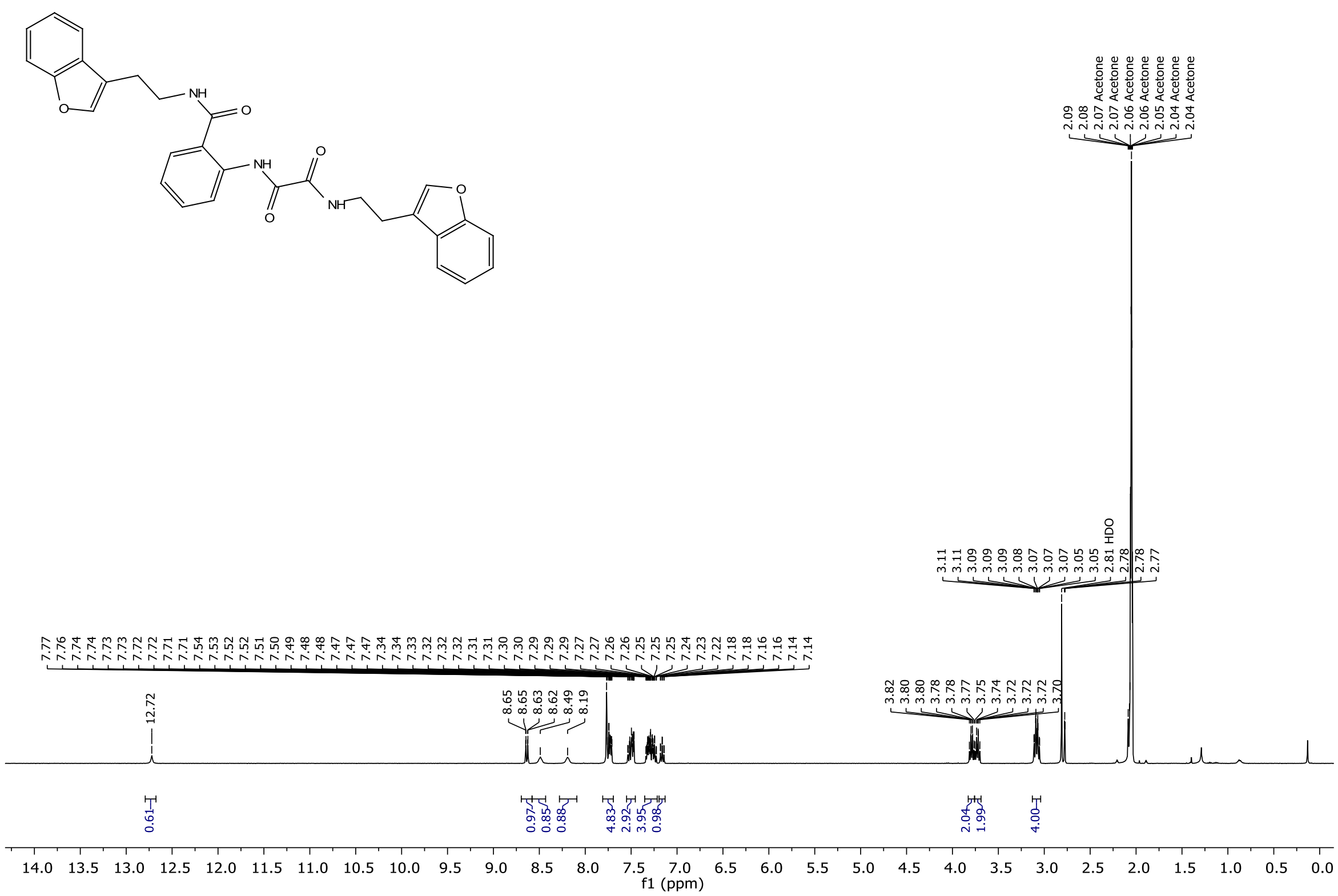

21, ^{13}C-NMR (101 MHz, Chloroform-d)

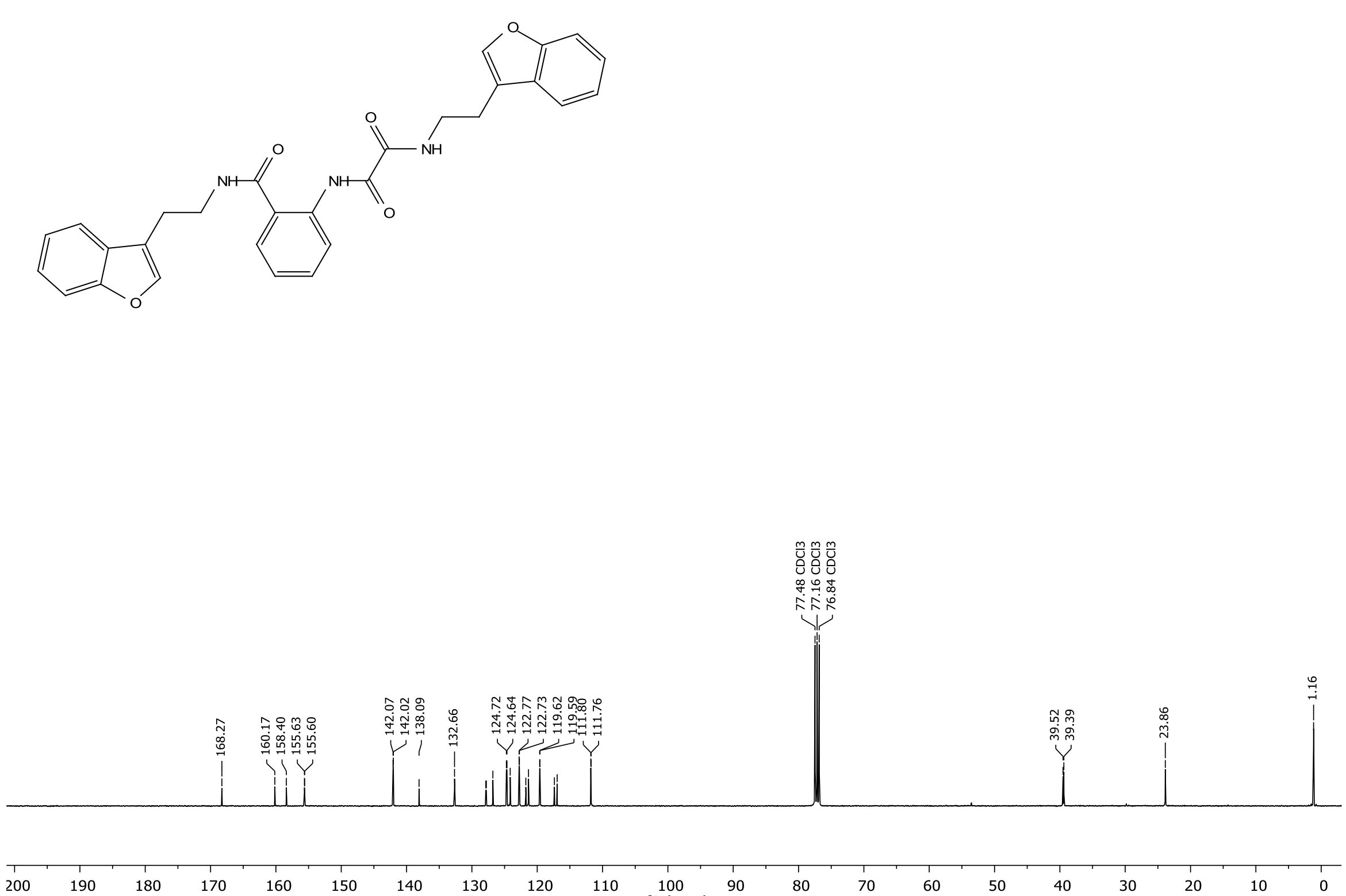

39, ^{1}H-NMR (400 MHz, acetone-d_6)

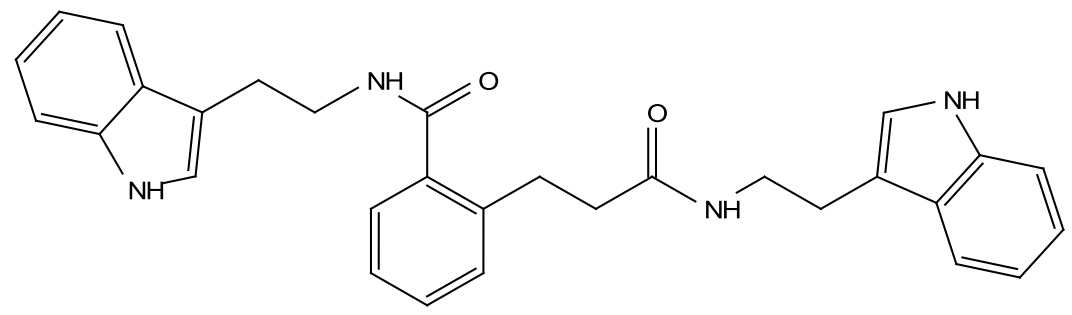

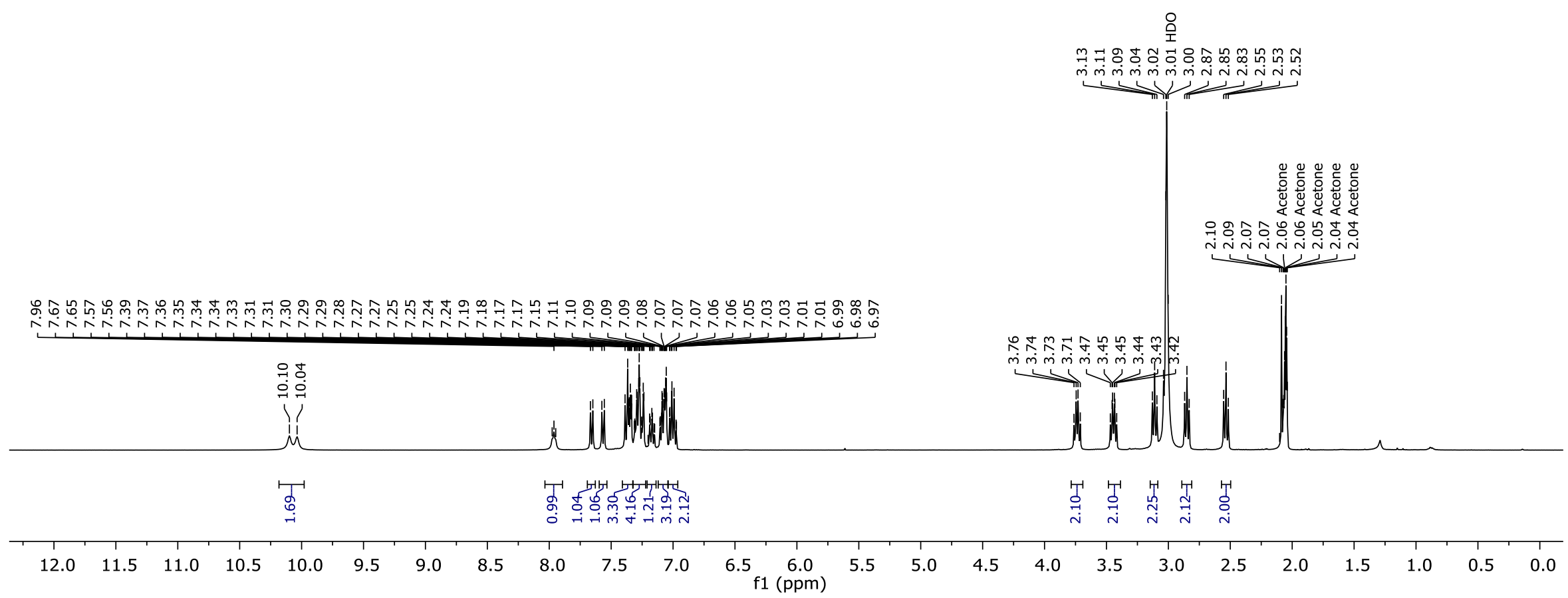

39, ^{13}C-NMR (101 MHz, acetone-d_6)

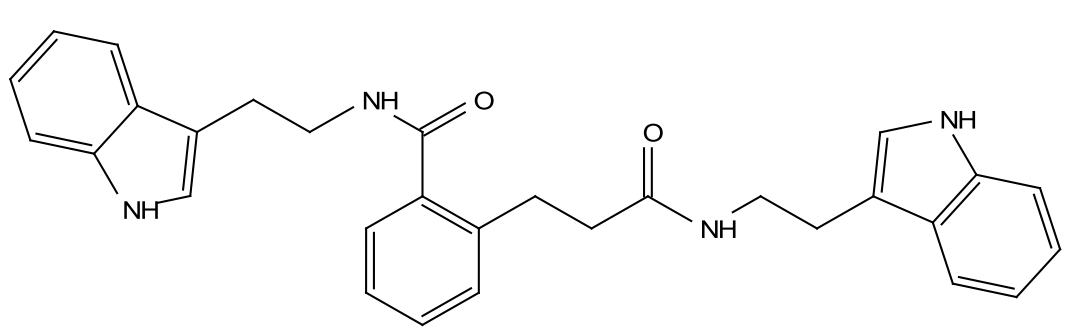

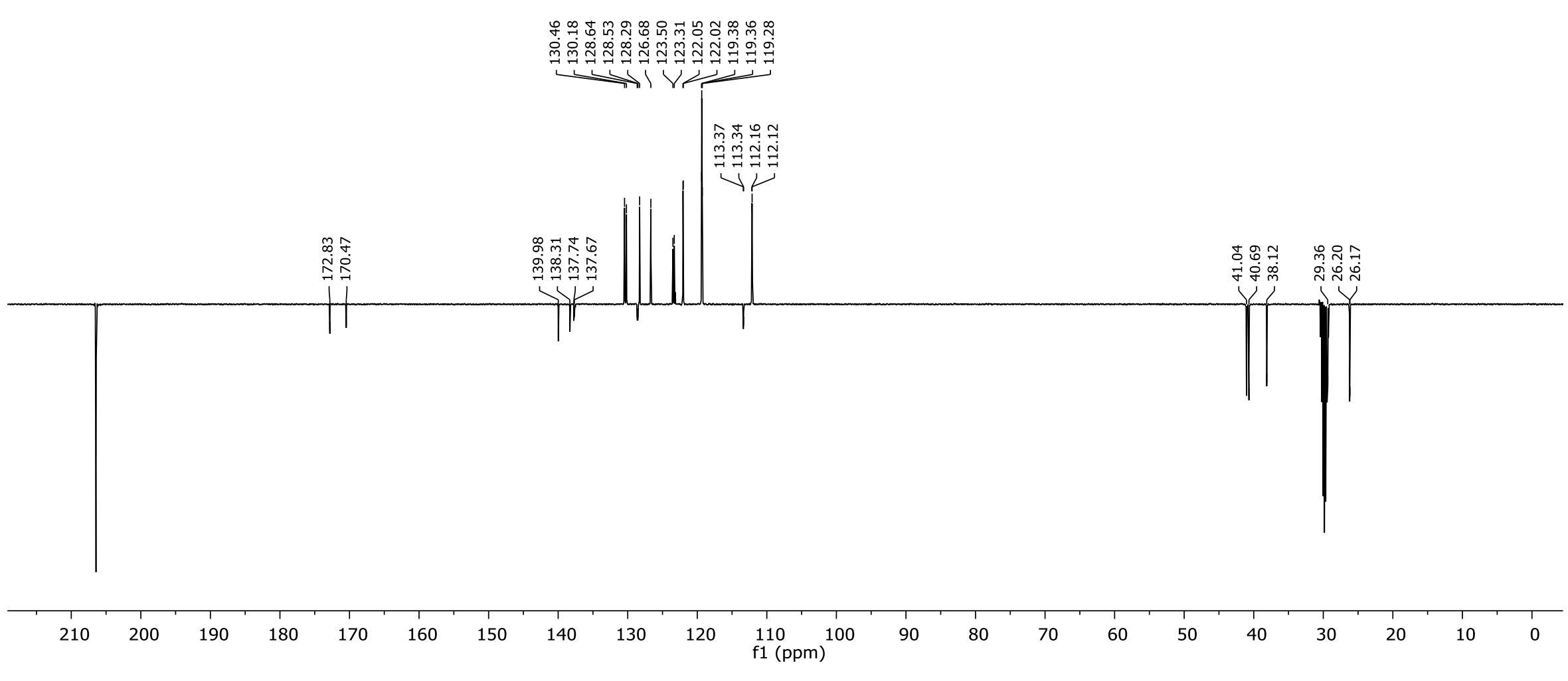

40, ^{1}H-NMR (400 MHz, Chloroform-*d*)

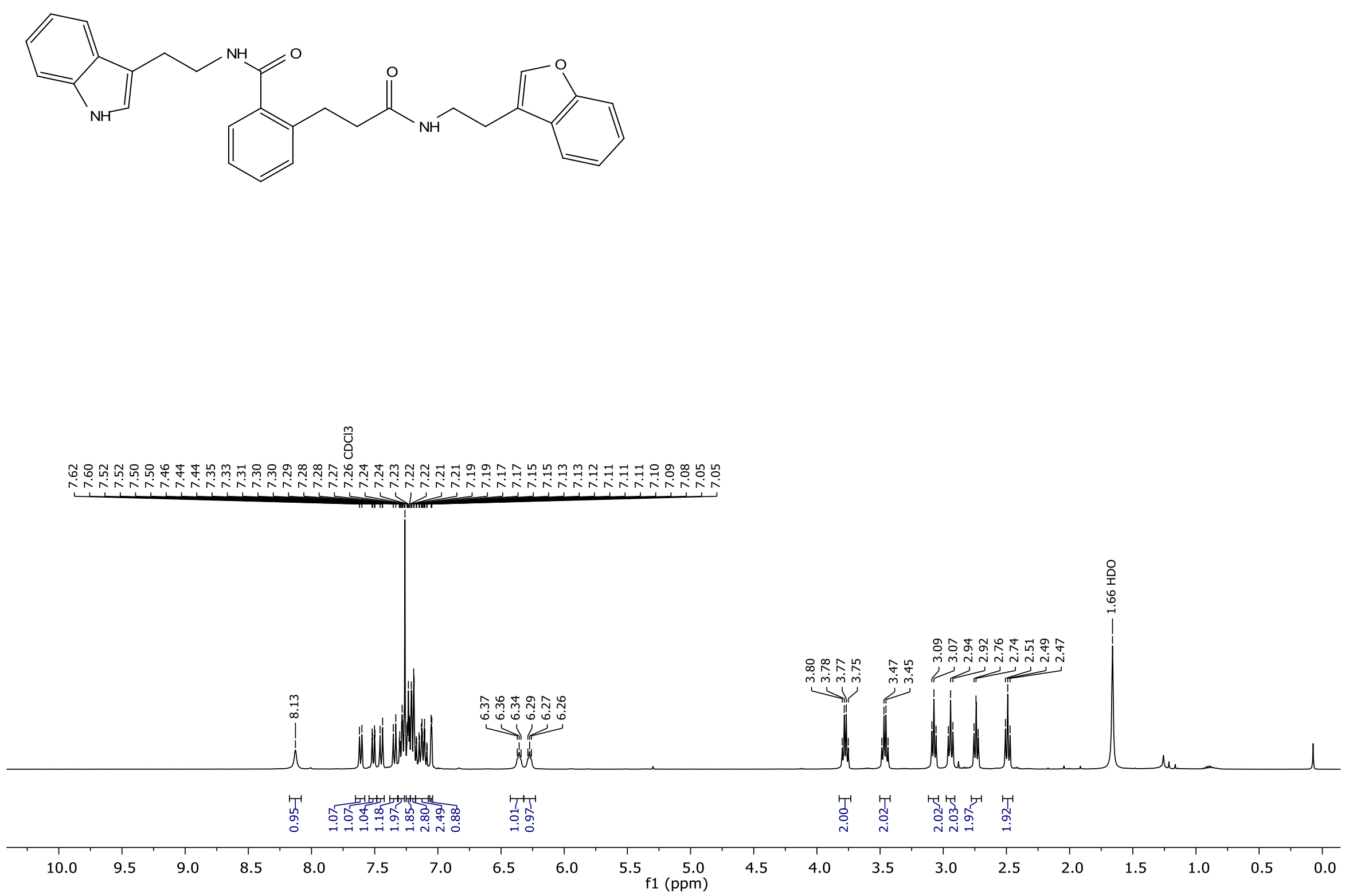

40, ^{13}C-NMR (101 MHz, Chloroform-*d*)

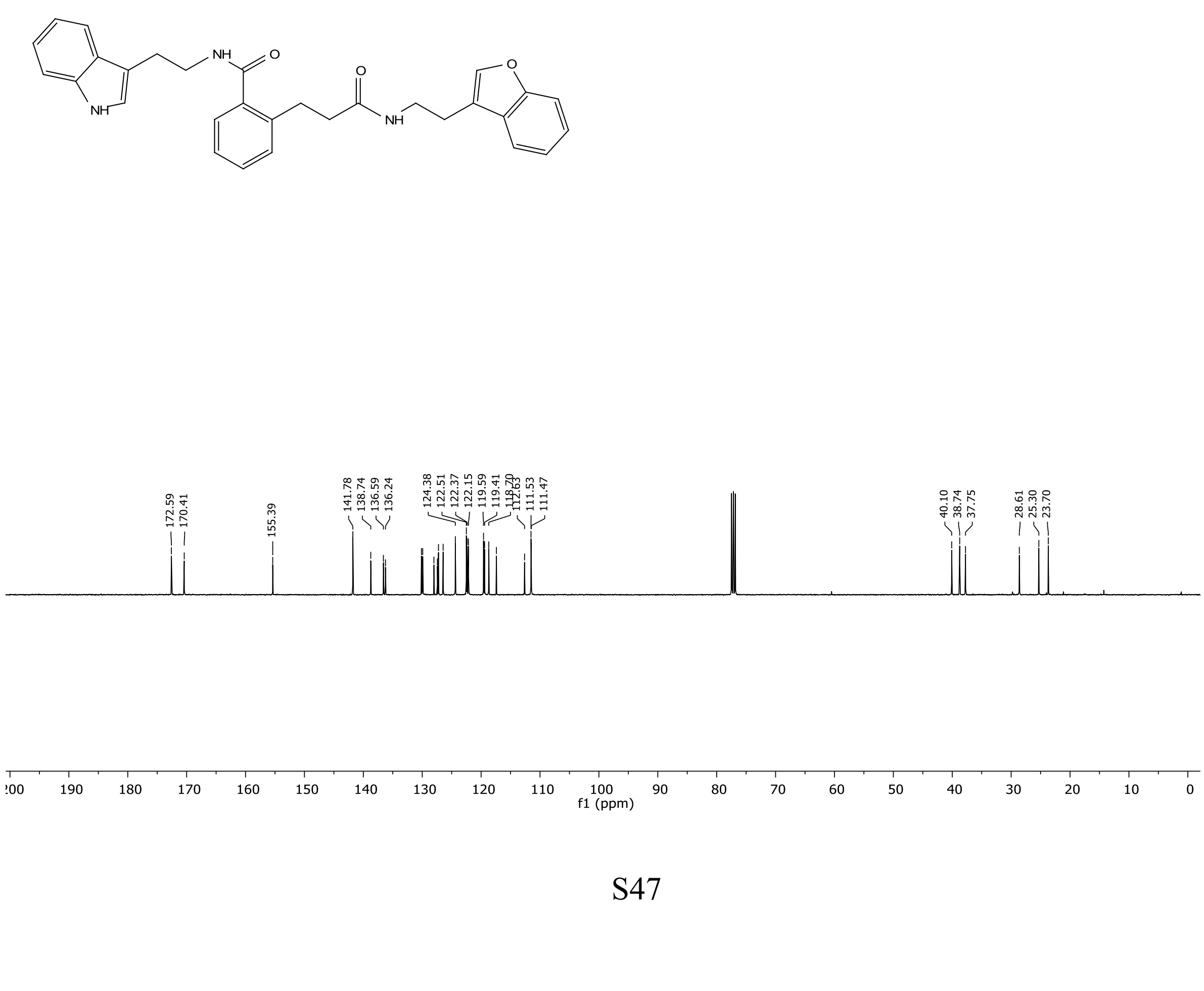

41, ^{1}H-NMR (400 MHz, Chloroform-*d*)

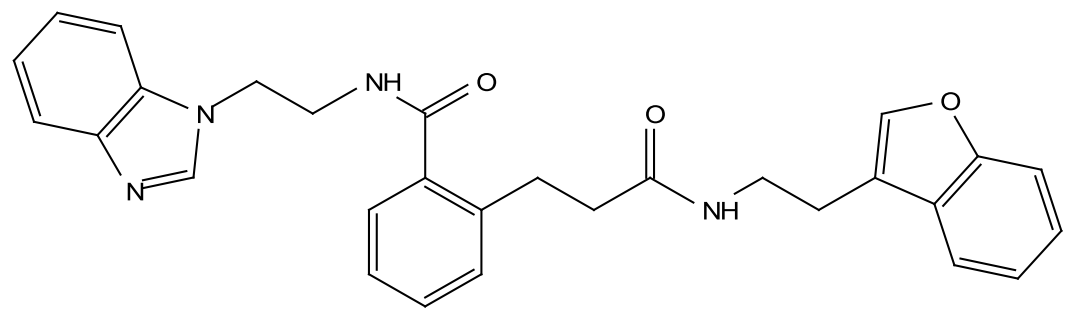

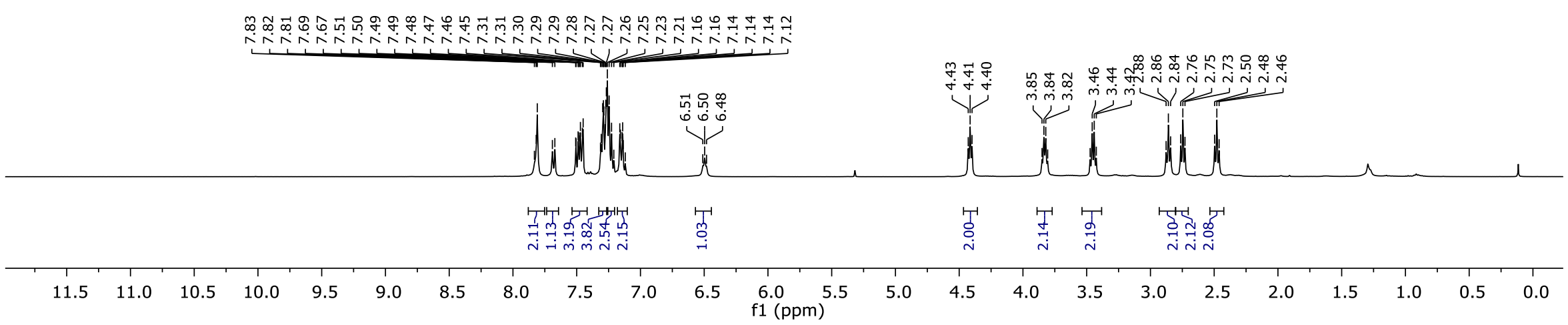

41, ^{13}C-NMR (101 MHz, Chloroform-*d*)

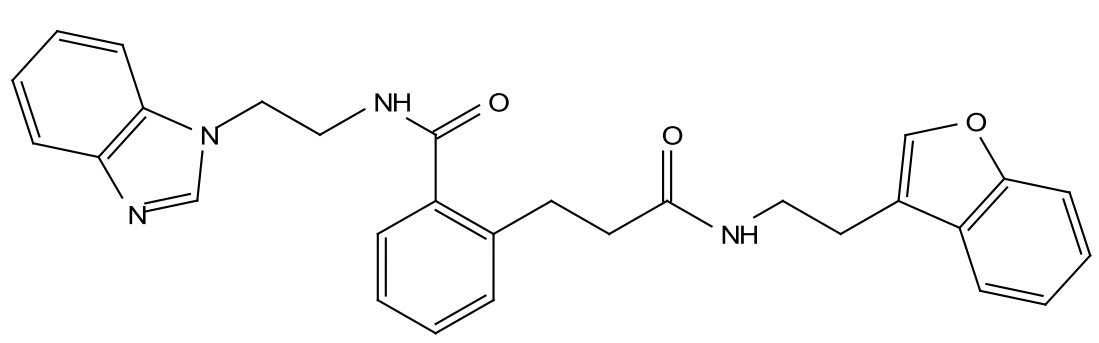

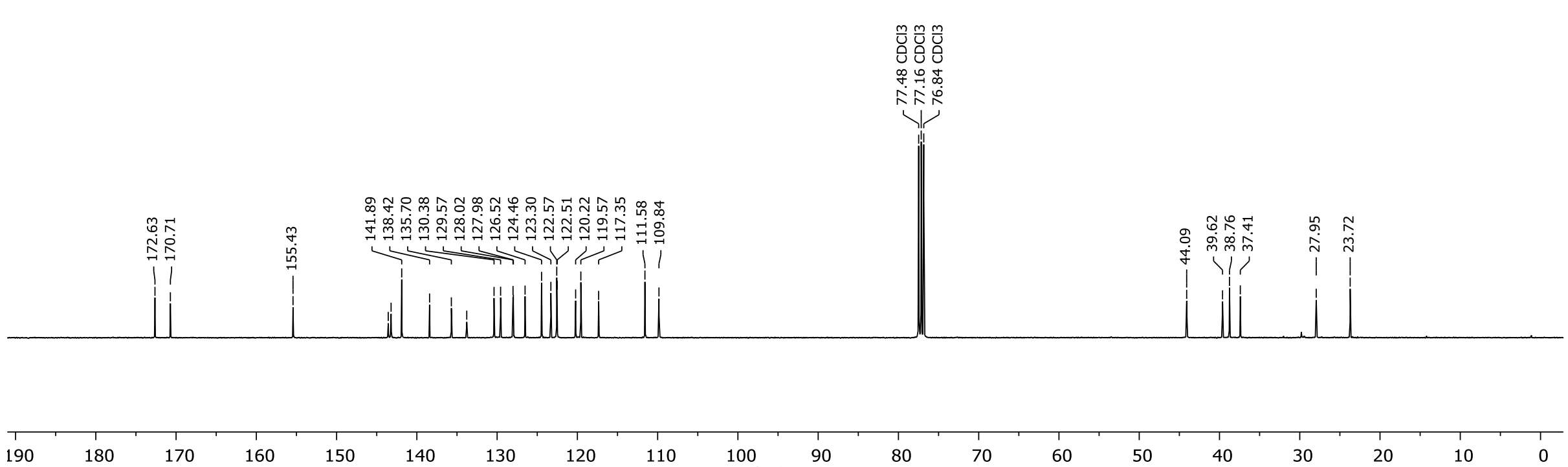

66, ^{1}H-NMR (400 MHz, acetone-d_6)

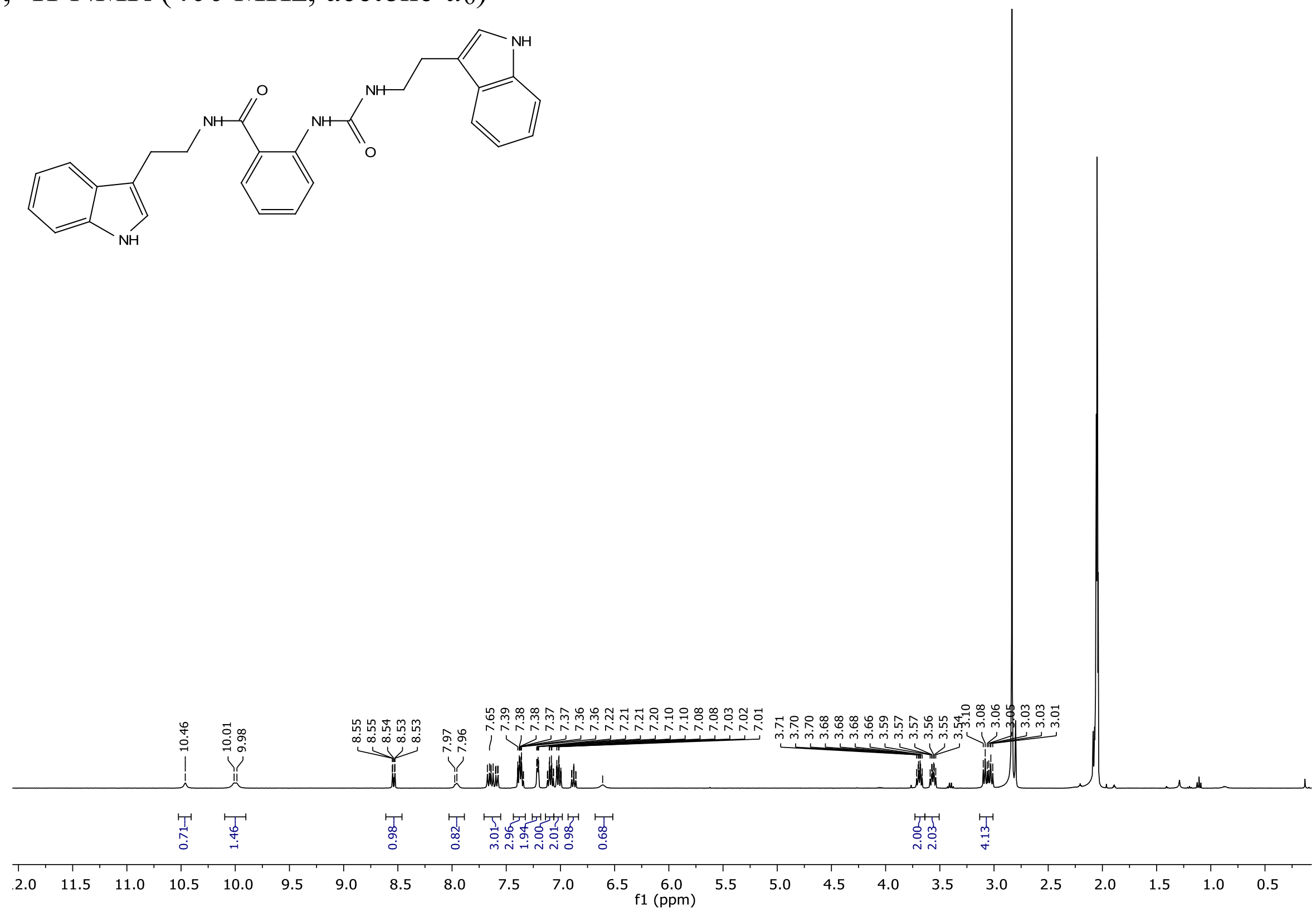

66, ^{13}C-NMR (101 MHz, acetone-d_6)

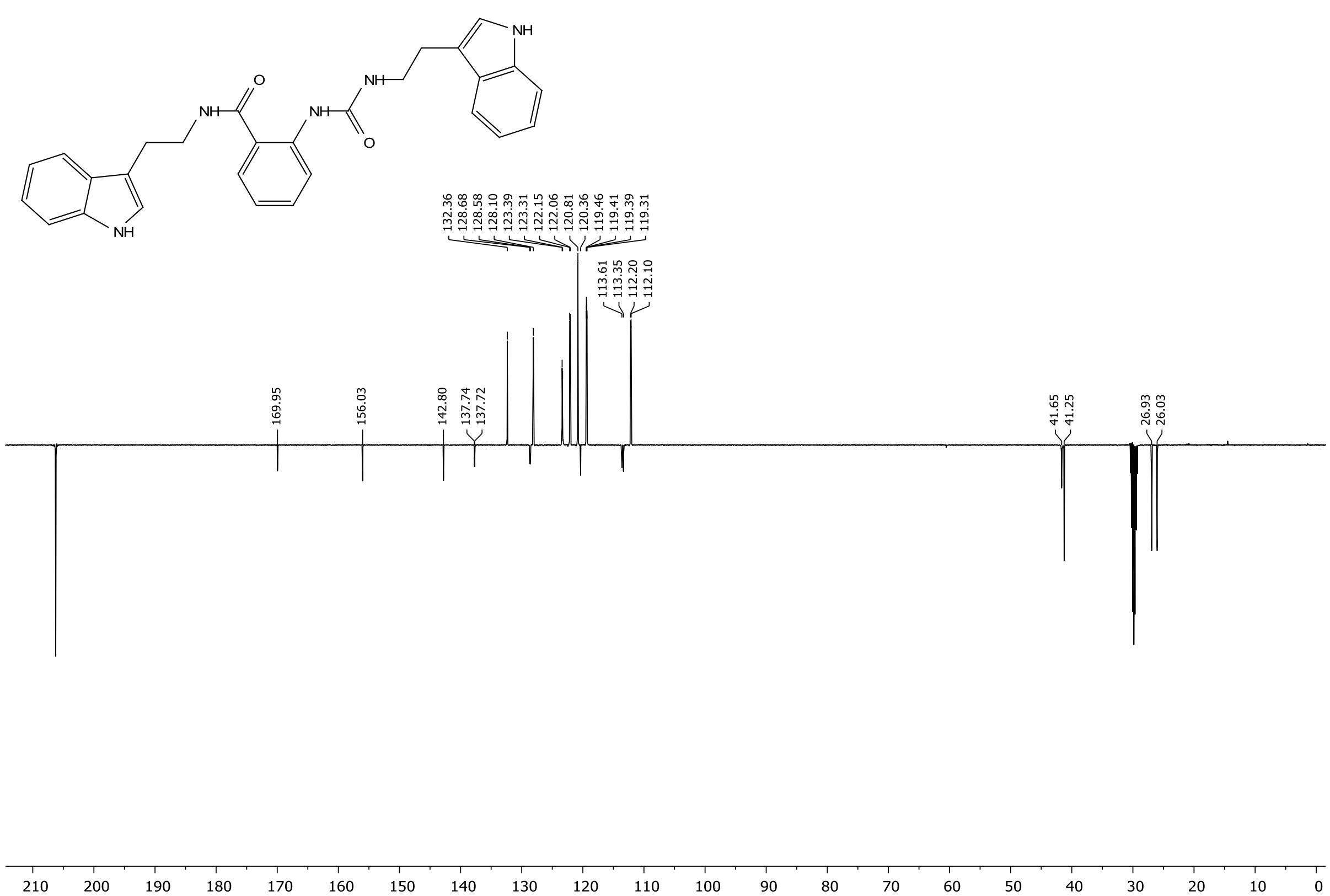

67, ^{1}H-NMR (400 MHz, acetone-d_6)

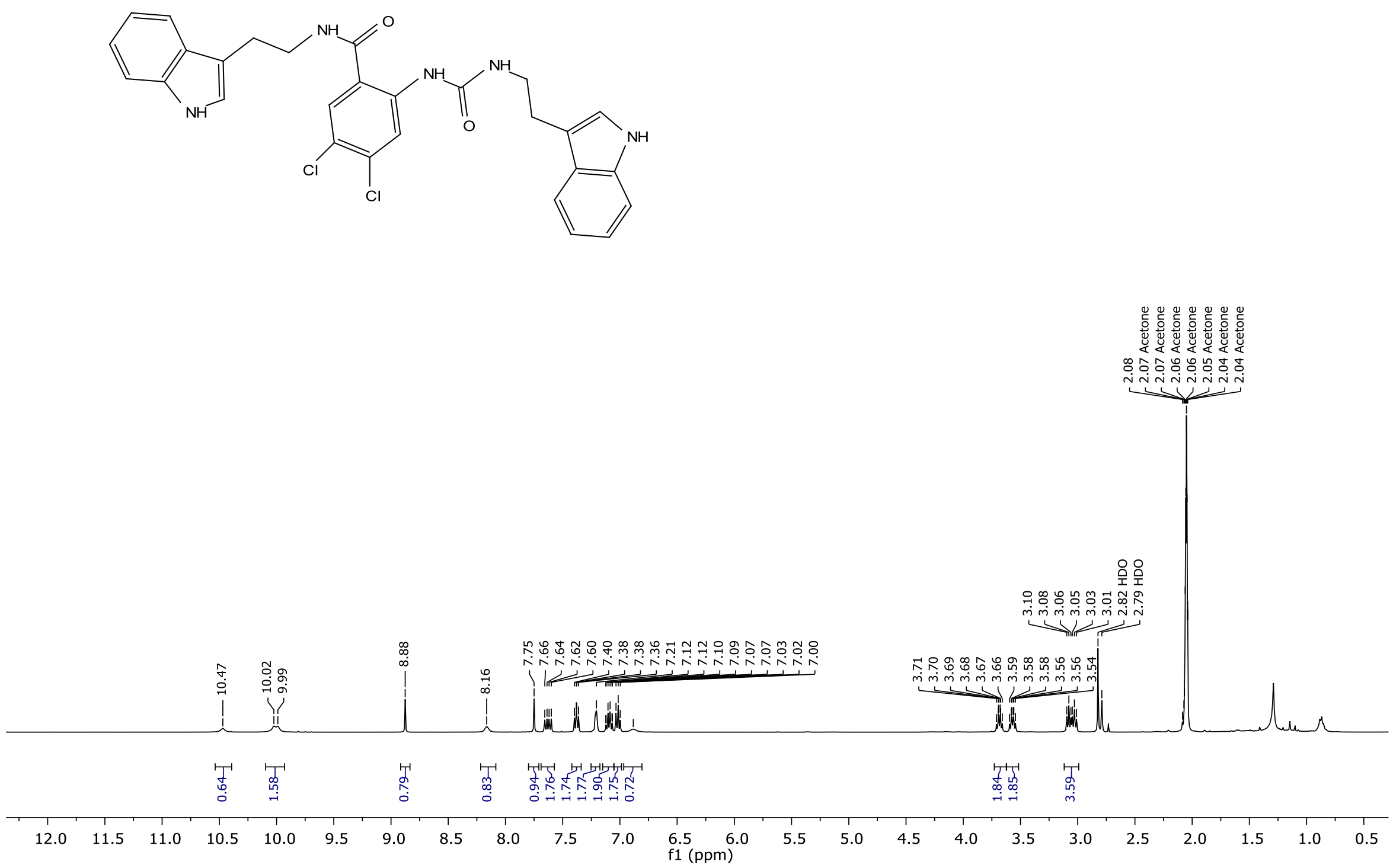

67, ^{13}C-NMR (101 MHz, acetone-d_6)

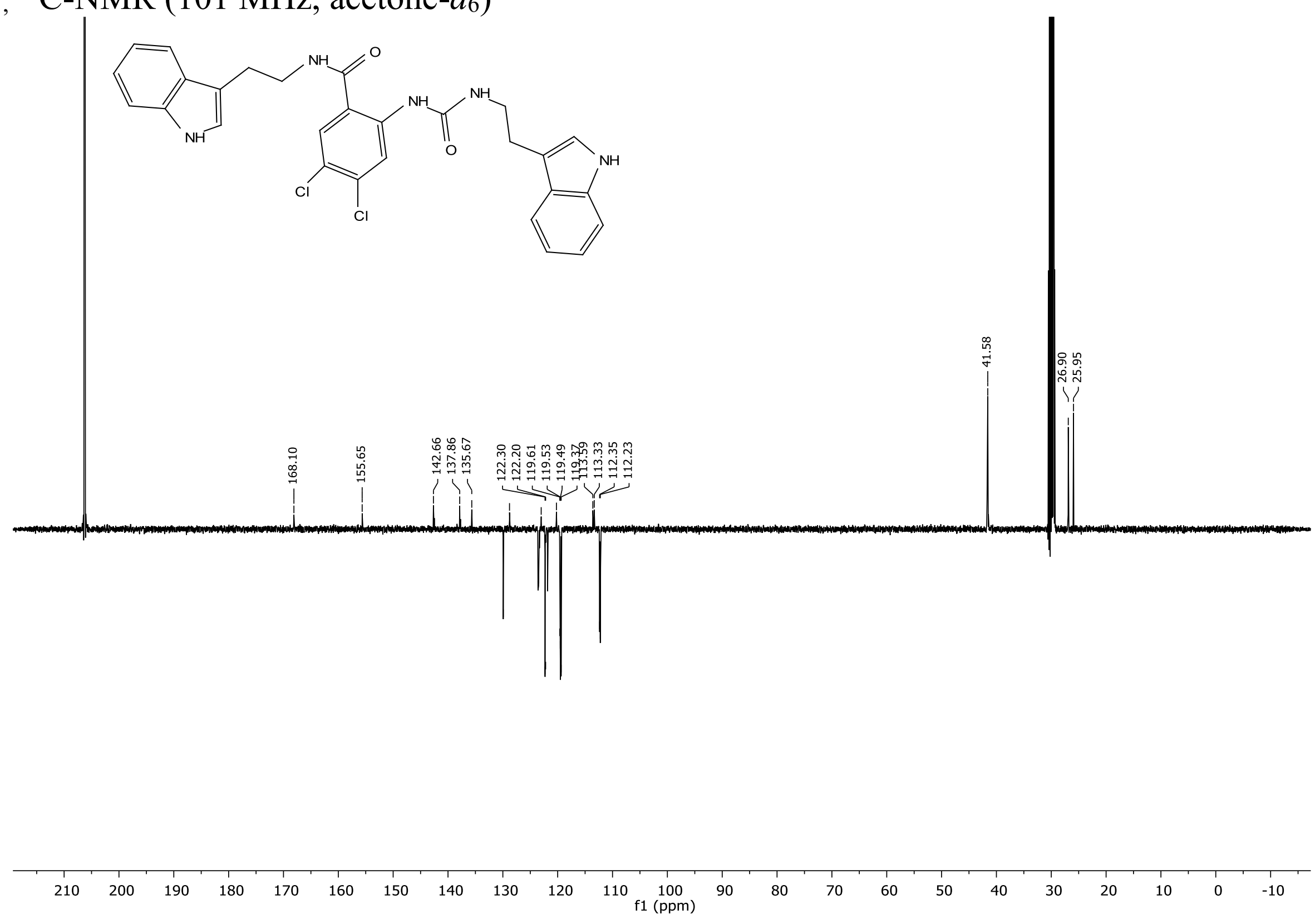

References

1. Galli, U.; Tron, G. C.; Purghè, B.; Grosa, G.; Aprile, S., Metabolic Fate of the Isocyanide Moiety: Are Isocyanides Pharmacophore Groups Neglected by Medicinal Chemists? *Chem. Res. Toxicol.* **2020**, *33* (4), 955-966.
2. Fallarino, F.; Uyttenhove, C.; Boon, T.; Gajewski, T. F., Endogenous IL-12 is necessary for rejection of P815 tumor variants in vivo. *J. Immunol.* **1996**, *156* (3), 1095-100.
3. Albini, E.; Rosini, V.; Gargaro, M.; Mondanelli, G.; Belladonna, M. L.; Pallotta, M. T.; Volpi, C.; Fallarino, F.; Macchiarulo, A.; Antognelli, C.; Bianchi, R.; Vacca, C.; Puccetti, P.; Grohmann, U.; Orabona, C., Distinct roles of immunoreceptor tyrosine-based motifs in immunosuppressive indoleamine 2,3-dioxygenase 1. *J. Cell. Mol. Med.* **2017**, *21* (1), 165-176.
4. Pallotta, M. T.; Orabona, C.; Volpi, C.; Vacca, C.; Belladonna, M. L.; Bianchi, R.; Servillo, G.; Brunacci, C.; Calvitti, M.; Bicciato, S.; Mazza, E. M. C.; Boon, L.; Grassi, F.; Fioretti, M. C.; Fallarino, F.; Puccetti, P.; Grohmann, U., Indoleamine 2,3-dioxygenase is a signaling protein in long-term tolerance by dendritic cells. *Nat. Immunol.* **2011**, *12* (9), 870-878.
5. Panfili, E.; Rezzi, S. J.; Adamo, A.; Mazzoletti, D.; Massarotti, A.; Miggiano, R.; Fallarini, S.; Ambrosino, S.; Coletti, A.; Molinaro, P.; Milella, M.; Paiella, S.; Macchiarulo, A.; Ugel, S.; Pirali, T.; Pallotta, M. T., Identification of a Compound Inhibiting Both the Enzymatic and Nonenzymatic Functions of Indoleamine 2,3-Dioxygenase 1. *ACS Pharmacol. Transl. Sci.* **2024**, *7* (10), 3056-3070.
